# Enantioselective Cobaltaphotoredox-Catalyzed C–H Activation

**DOI:** 10.1021/jacs.4c08459

**Published:** 2024-08-15

**Authors:** Yang Xu, Ye Lin, Simon L. Homölle, João C. A. Oliveira, Lutz Ackermann

**Affiliations:** Wöhler-Research Institute for Sustainable Chemistry (WISCh), Georg-August-Universität Göttingen Tammannstraße 2, Göttingen 37077, Germany

## Abstract

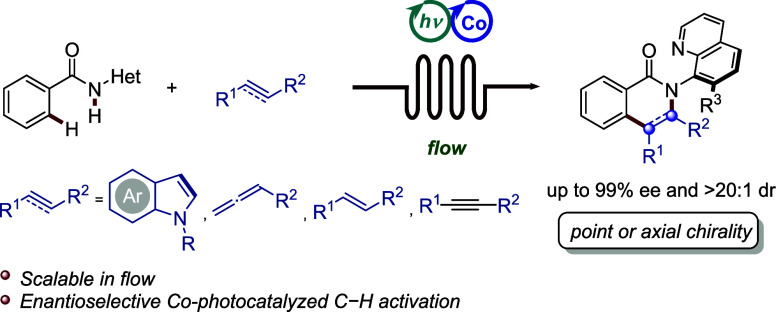

The quest for sustainable strategies in molecular synthesis has spurred the emergence of photocatalysis as a particularly powerful technique. In recent years, the application of photocatalysis in this context has greatly promoted the development of asymmetric catalysis. Despite the impressive advances, enantioselective photoinduced strong arene C–H activations by cobalt catalysis remain unexplored. Herein, we report a strategy that merges organic photoredox catalysis and enantioselective cobalt-catalyzed C–H activation, enabling the regio- and stereoselective dual functionalization of indoles in an enantioselective fashion. Thereby, the assembly of various chiral indolo[2,3-*c*]isoquinolin-5-ones was realized with high enantioselectivities of up to 99%. The robustness of the cobaltaphotoredox catalysis was demonstrated through enantioselective C–H activation and annulations in a continuous flow to provide straightforward access to central and axially chiral molecules.

## Introduction

Over the past decades, photochemistry has been identified as an environment-friendly platform for molecular synthesis.^1−9^ The merger of photoinduced catalysis with enantioselective catalysis,^10−12^ such as organocatalysis,^13−16^ enzymatic catalysis,^17^ and transition metal catalysis,^18,19^ provides a powerful strategy to satisfy the growing demand for enantiomerically pure small molecules. In contrast to traditional enantioselective methods, the introduction of photoredox catalysis has enabled mild and green reaction conditions. Since the pioneering enantioselective cross-coupling reaction through synergistic photocatalysis and nickel catalysis by Molander et al.,^20^ enantioselective photoinduced metal-catalysis has become a popular strategy for expanding the synthetic utility of visible-light photocatalysis, largely employing prefunctionalized substrates.^21^

**Figure 1:**
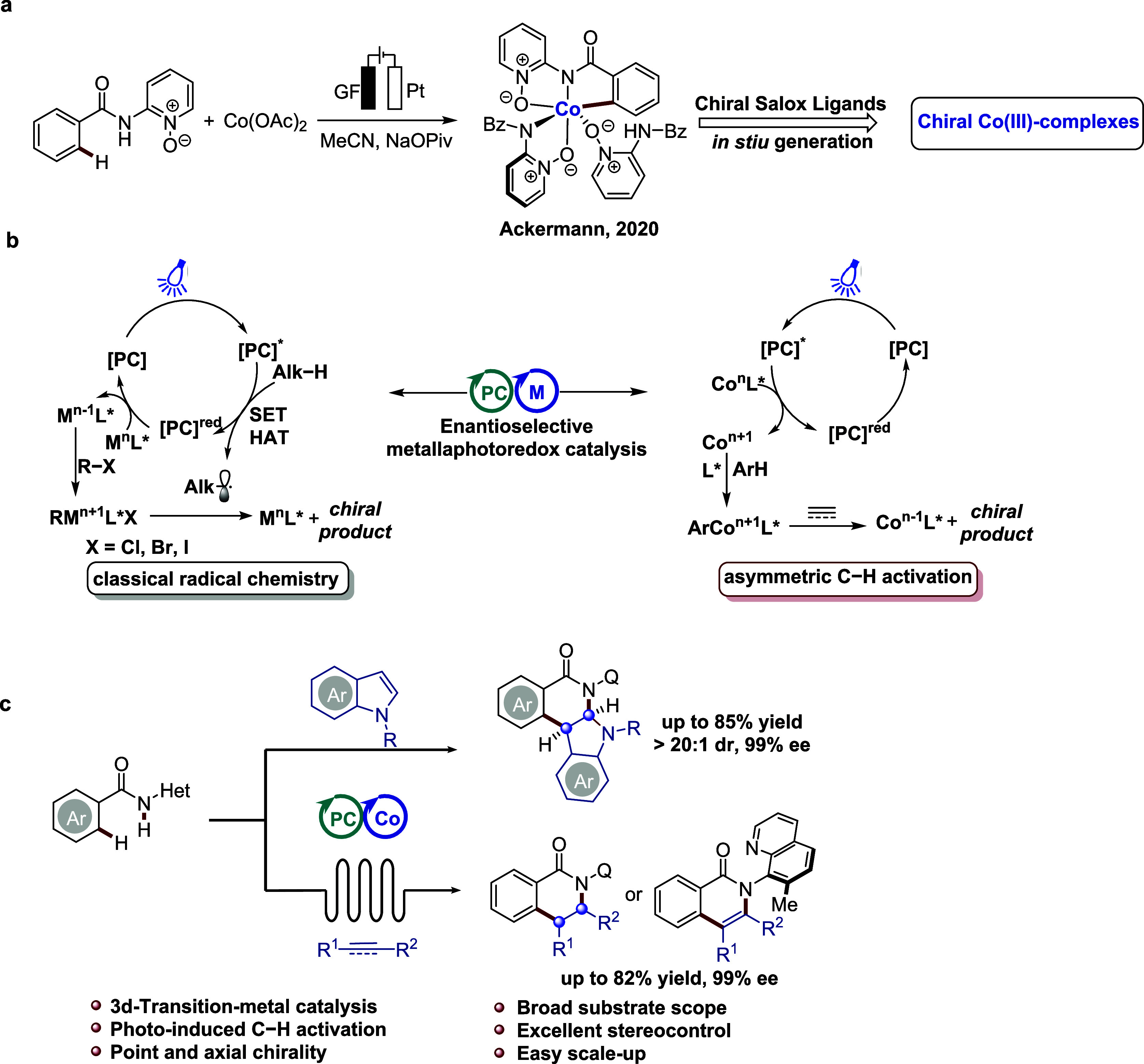
Design blueprint for enantioselective photoinduced cobalt-catalyzed C–H activation. (a) Cobalt(III) complex featuring bidentate L,X-type ligands. (b) Photoinduced metal-catalyzed enantioselective C–H activation. (c) Enantioselective photoinduced cobalt-catalyzed activation of strong C–H bonds.

Transition-metal-catalyzed enantioselective C–H activation has emerged as an efficient methodology for constructing C–C and C–Het bonds.^22−25^ With a focus on developing sustainable strategies that offer economic and environmental benefits, recent momentum has shifted toward Earth-abundant, less toxic metals, particularly 3d transition metals, for C–H activation.^26−31^ In 2017, as a proof of concept, we reported on the first enantioinduction in high valent cobalt(III) catalysis exploiting a monoprotected amino acid (MPAA) ligand, albeit with low enantiomeric excess.^32a^ The design of a C_2_-symmetric carboxylic acid enabled the first highly enantioselective high valent cobalt-catalyzed C–H activation.^32b^ Detailed mechanistic studies, including the isolation of key cyclometalated cobalt(III) complexes with two benzamide substrates as ligands (Figure 1a),^33^ showed the potential of chiral LX-type ligands for enantiocontrol. Thus, enantioselectivity control in cobalt-catalyzed C–H activations can be enabled by replacing one of the benzamide substrates with a bidentate chiral ligand, such as MPAAs.^33b^ Especially chiral salicyloxazoline ligands, originally developed by Bolm in 1991,^34^ have recently been identified as a powerful tool in cobalt catalysis.^31b^ In addition, this strategy has induced significant advances in catalytic enantioselective aryl C–H annulations with alkenes, allenes, alkynes, and isonitriles.^35^ In this regard, we have recently employed chiral salox preligands for electrooxidative annulation of strained bicyclic alkenes^35j^ as well as enantio- and diastereo-selective alkyne annulations by electrocatalysis^35i^ through a C–N reductive elimination as the proposed enantio-determining step.^35j^ Mechanistic studies were supportive of the enantioselective alkene annulation to proceed via a cobalt(III/I) manifold.^33a,35j^ Despite of this indisputable progress in electrocatalysis, enantioselective cobalt-photocatalyzed C–H activations had thus far unfortunately proven elusive.^51^ Fused polycyclic indoline motifs bearing contiguous stereogenic centers are essential *N*-heterocycles in various bioactive molecules and natural products, which exhibit diverse bioactivities.^36−41^ Of particular note, catalytic asymmetric dearomatization (CADA) of indoles is considered as one of the most powerful strategies to assemble such chiral indoline structures.^42^ However, cobalt-catalyzed asymmetric C–H annulations with indoles remain elusive.

The merger of enantioselective 3d transition-metal catalysis with photoredoxcatalysis has only very recently been realized. These methods were operative by single electron transfer (SET) or hydrogen atom transfer (HAT) processes via homolytic C–H cleavage and were thus limited to weak and activated C–H bonds (Figure 1b, left).^18,21^ In contrast, achieving enantioselective activation of strong arene bonds under visible light is underdeveloped because of suitable ligands that can simultaneously reduce the metal’s redox potential and control full enantioselectivity (Figure 1b, right), were only available with toxic and precious palladium.^43^ Hence, enantioselective photoinduced C–H activation by Earth-abundant 3d metals is highly desirable. To this end, we report here the first enantioselective cobaltaphotoredox-catalyzed direct C–H activation (Figure 1c). Salient features of our findings include: (1) enantioselective photoinduced cobalt-catalyzed C–H activation for enantioselective dearomatization of indoles, (2) no need for sacrificial chemical

oxidants, (3) organic dyes in lieu of precious metal complexes as photocatalysts, (4) efficient cobaltaphotoredox enantioselective catalysis in a continuous flow, and (5) broad substrate scope including the construction of point chirality and axial chirality, with high catalytic efficiency, and excellent stereoselectivities (up to 85% yield, >20:1 dr, and 99% ee).

## Results and Discussion

**Figure 2:**
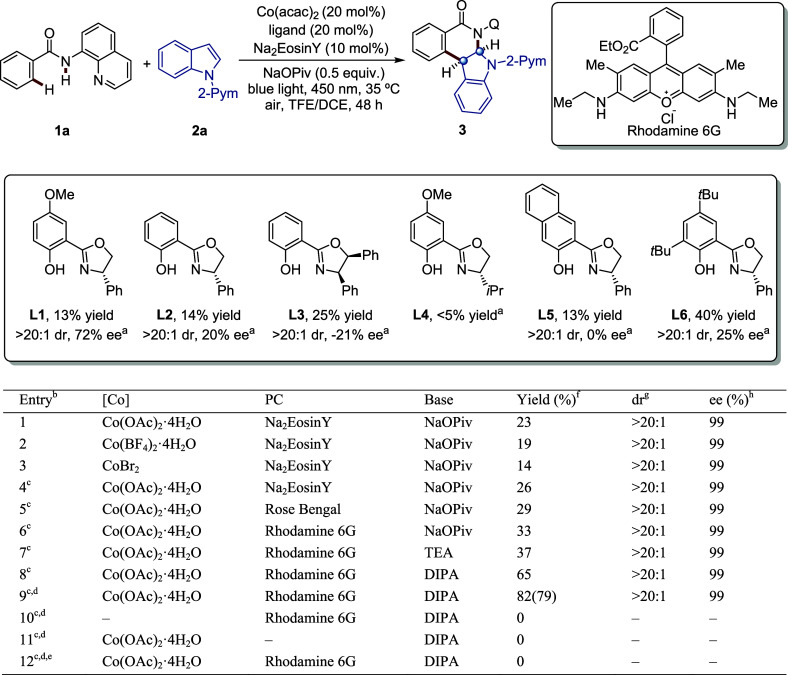
Optimization of the reaction parameters: ^a^Reaction conditions: **1a** (0.15 mmol, 1.0 equiv), **2a** (0.15 mmol, 1.0 equiv), PC (0.015 mmol, 10 mol %), [Co] (0.03 mmol, 20 mol %), ligand (0.03 mmol, 20 mol %), base (0.075 mmol, 0.5 equiv), TFE (2.0 mL), DCE (0.5 mL), 35 °C, 48 h. ^b^Reaction conditions: **1a** (0.15 mmol, 1.0 equiv), **2a** (0.15 mmol, 1.0 equiv), PC (0.015 mmol, 10 mol %), [Co] (0.03 mmol, 20 mol %), **L6** (0.03 mmol, 20 mol %), base (0.075 mmol, 0.5 equiv), TFE (2.0 mL), DCE (0.5 mL), 35 °C, 48 h. ^c^Base (0.195 mmol, 1.3 equiv). ^d^TFE (1.0 mL) and DCE (0.25 mL). ^e^Without blue light. ^f^Yields were determined by ^1^H NMR using 1,3,5-trimethoxybenzene as the internal standard; isolated yields after column chromatography are shown in parentheses. ^g^The dr value was determined by^1^H NMR analysis. ^h^The ee value was determined by chiral high-performance liquid chromatography (HPLC) analysis. Pym = pyrimidine. Q = 8-quinolinyl. PC = photocatalyst. TFE = 2,2,2-trifluoroethanol. DCE = 1,2-dichloroethane. TEA = triethylamine. DIPA = diisopropylamine.

Thus, we were intrigued by constructing chiral indolines through enantioselective photoinduced cobalt-catalyzed dearomative cyclizations of indoles. Initially, we probed the use of Co(acac)_2_ as the precatalyst and salicyloxazoline **L1** as the chiral ligand for the C–H activation of benzamide **1a** with *N*-pyrimidyl indole **2a**. Performing the reaction under blue light (450 nm) irradiation at ambient temperature under an atmosphere of ambient air. With Na_2_EosinY as the photocatalyst, and NaOPiv as the base, the desired annulation product 3 was obtained in 13% yield with >20:1 dr and 72% ee (Figure 2). Next, a variety of chiral ligands, including phosphoric and carboxylic acids, were evaluated, but none of them provided the desired product with satisfactory enantioselectivity. To our delight, the desired product **3** was formed in an encouraging yield of 40% using **L6**, albeit with a low enantioselectivity of 25%. Further exploration of different cobalt salts and organic dyes revealed Co(OAc)_2_·4H_2_O with rhodamine 6G as a suitable catalyst combination in this transformation (Figure 2, entry 6). Indeed, the best result was obtained with the organic base diisopropylamine (DIPA) and a higher concentration (Figure 2, entry 9). Furthermore, control experiments confirmed the crucial roles of Co(OAc)_2_·4H_2_O, ligand **L6**, rhodamine 6G, blue light, and DIPA in the formation of **3** (for detailed optimization, see the Supporting Information).

**Figure 3:**
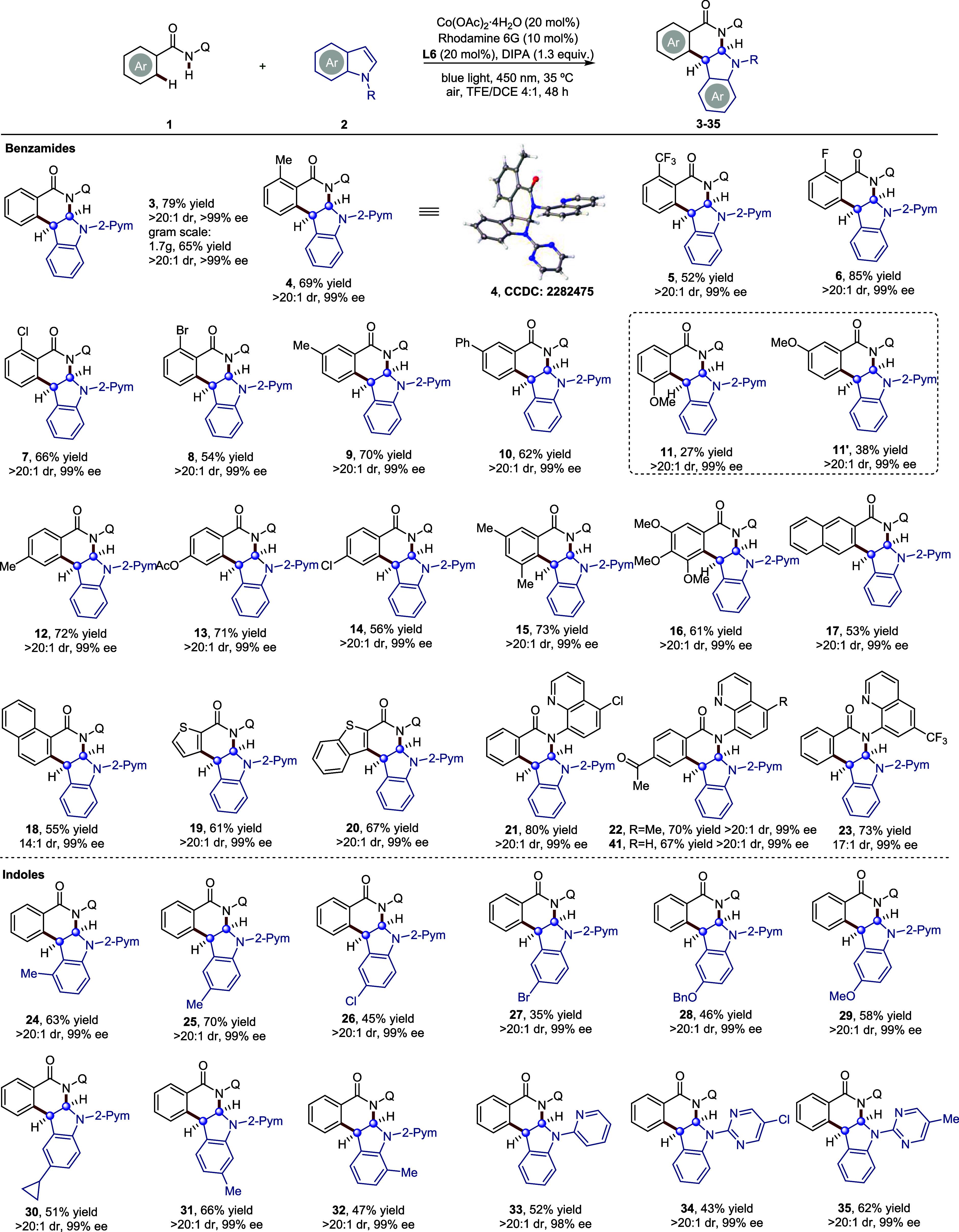
Scope of the benzamides with indoles. Reaction conditions: **1** (0.15 mmol, 1.0 equiv), **2** (0.15 mmol, 1.0 equiv), PC (0.015 mmol, 10 mol %), Co(OAc)_2_·4H_2_O (0.03 mmol, 20 mol %), **L6** (0.03 mmol, 20 mol %), DIPA (0.2 mmol, 1.3 equiv), TFE (1.0 mL), DCE (0.25 mL), 35 °C, air, 48 h.

With the optimized conditions in hand, we explored the versatility of ambient cobaltaphotoredox catalysis. As shown in Figure 3, the photoinduced cobalt-catalyzed enantioselective dearomative C–H activation was applicable to a range of N-quinolyl benzamides **1** and indoles **2**. Various benzamides **1**, including those with ortho, meta, and para substituents, both electron-donating and electron-withdrawing groups, proved to be compatible, and the desired products **4** to **14** were obtained as single diastereoisomers in moderate to good yields with excellent enantioselectivities. The absolute configuration and connectivity of product **4** were unambiguously confirmed by X-ray crystallography (CCDC: 2282475). For the meta-methoxy substrate **1i**, a regioisomeric mixture (**11**, 27% yield, 99% ee, and **11′**, 38% yield, >99% ee) was obtained due to the strong electron-donating effect of methoxy group and the weak directing ability of the methoxy group, which allowed

C–H activation occurs at the congested ortho position. In contrast, meta-methyl and meta-phenyl substituents provided good access to the products **9** and **10**. Di- and trisubstituted N-quinolyl benzamides, as well as heteroaromatics with a potentially coordinating sulfur, were also efficiently activated, leading to the desired products **15** to **20** with 99% ee. Additionally, aminoquinolines substituted with 5-Cl, 5-Me, and 6-CF_3_ groups were also tolerated, affording the desired products **21** to **23** with excellent stereoselectivities. To further demonstrate the utility of our strategy, we investigated the scope of indole derivatives **2**. Various electron-donating groups at different positions (**24**-**25**, **28**-**32**) were amenable. In addition, halogen-substituents (5-Cl and 5-Br) were well tolerated. Notably, N-pyridine indole and other N-pyrimidine derivatives with methyl or chloro substituents proved to be viable as well. Furthermore, we performed a gram-scale synthesis of **3** using our standard reaction conditions in a batch reactor, affording a yield of 65%, >20:1 dr, and 99% ee (Figure 3).

### Mechanistic Studies

**Figure 4:**
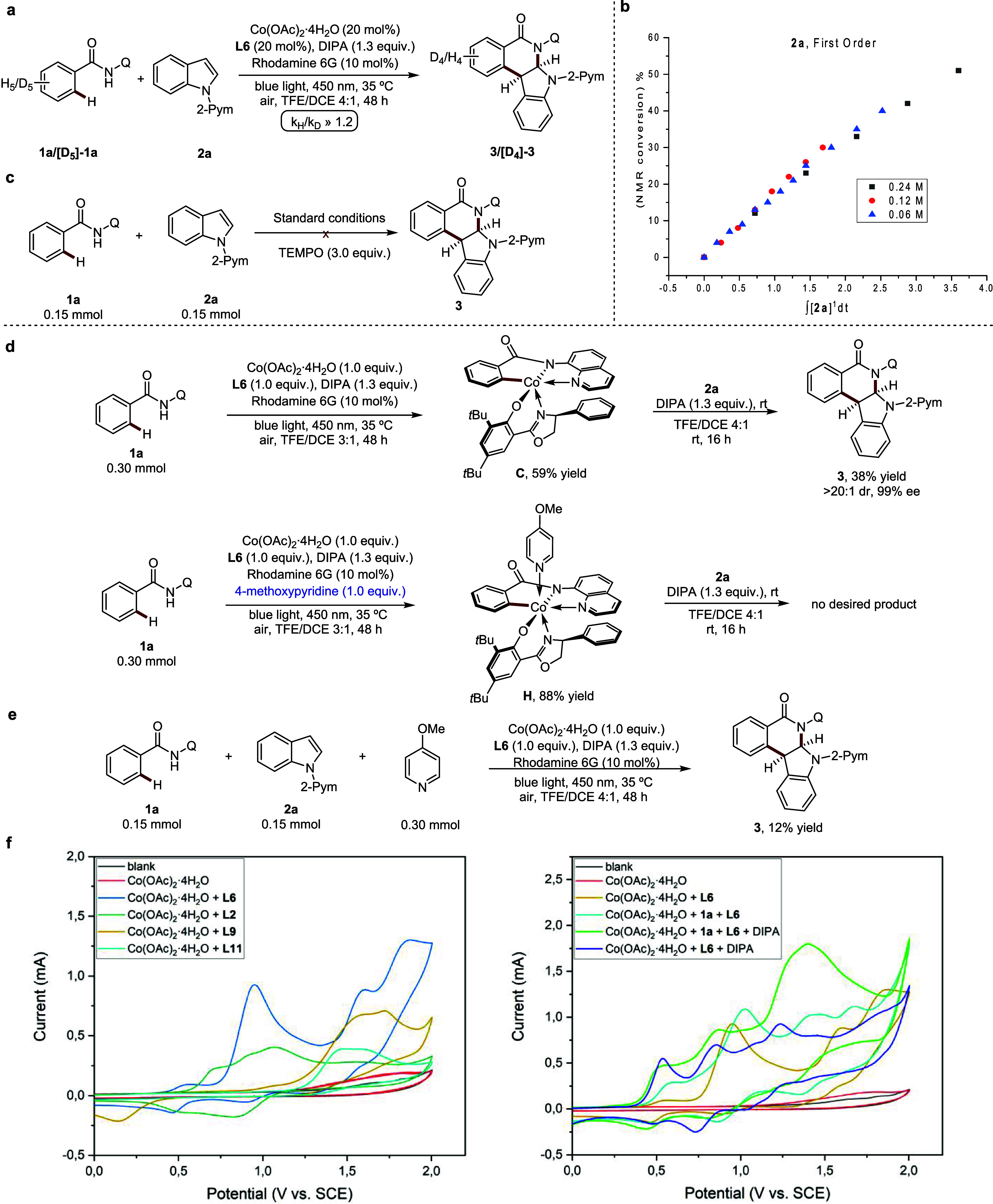
Mechanistic studies. (a) KIE experiment. (b) The reaction order of indole (**2a**). (c) Radicaltrap experiment. (d) The synthesis of cobaltacycle intermediates and stoichiometric reactions of cobaltacycle intermediate. (e) Control experiments. (f) Cyclic voltammetry measurements.

To understand the mechanism of the cobaltaphotoredox-catalyzed enantioselective C–H activation, we performed intermolecular competition experiments between substrates **1a** and **[D5]-1a** to determine the kinetic isotope effect (KIE), indicating that the C–H cleavage was not involved in the rate-determining step (Figure 4a). Next, we studied the kinetic profile using variable-time normalization analysis (VTNA).^44,45^ Thus, we observed a first-order dependence on indole **2a**, being indicative of insertion or reductive elimination involved in the rate-determining step (Figure 4b). We found an inverse-first-order dependence on benzamide **1a**, and a fractional order (0.5) for Co(OAc)_2_·4H_2_O and **L6**. We hypothesize that this is due to the coordination of the benzamide **1a** with cobalt to generate a catalytically ineffective intermediate (see the Supporting Information). Subsequently, the addition of the radical inhibitor 2,2,6,6-tetramethylpiperidine-1-oxyl (TEMPO) to the reaction completely suppressed the formation of **3**, suggesting the

formation of radical intermediates (Figure 4c). Then, cobalt(III) intermediates were synthesized to investigate the coordination mode at cobalt and stereoinduction (Figure 4d). The reaction of substrate **1a** with 1.0 equiv of Co(OAc)_2_·4H_2_O, **L6**, and 10 mol % rhodamine 6G under blue light irradiations yielded the penta-coordinated cobaltacycle **C** which was fairly characterized by 1H NMR spectroscopy and high-resolution mass spectrometry. The intermediate **C** could then react with indole **2a** to provide **3** in 38% yield with >20:1 dr and 99% ee, which suggests the involvement of intermediate **C** in the catalytic cycle. Moreover, using 4-methoxypyridine to stabilize the intermediate **C** resulted in the formation of **H** with 88% yield, which did not yield **3**, when being reacted with indole **2a**. Additionally, when pyridine was added to the standard reaction, only a 12% yield was obtained, pointing toward its competitive coordination (Figure 4e). A comparison of the oxidation potentials of Co(OAc)_2_·4H_2_O modified with different ligands revealed no oxidation peak for cobalt with **L9** or **L11**. However, compared to the oxidation potential of cobalt with **L2** at 0.60 V, the cobalt complex associated with ligand **L6** exhibited an oxidation potential of 0.50 V, indicating that **L6** enhances the susceptibility for a cobalt(II/III) regime (Figure 4f).

Additionally, in order to assess the enantio-determining step DFT calculations were carried out at the ωB97X-D/def2-TZVPP+SMD(TFE)//TPSS-D3(BJ)/def2-SVP level of theory (Figure S11).^46^ These were carried out between migratory insertion and reductive elementary steps for three possible spin states, namely, singlet, triplet, and quintet, for the major enantiomer pathway. We observed a spin-crossover for both elementary steps. An assessment of the energy barriers revealed that migratory insertion is in fact the enantio-determining step with an energy barrier of 19.4 kcal mol^–1^ at the singlet surface.

### Proposed Catalytic Cycle

**Figure 5:**
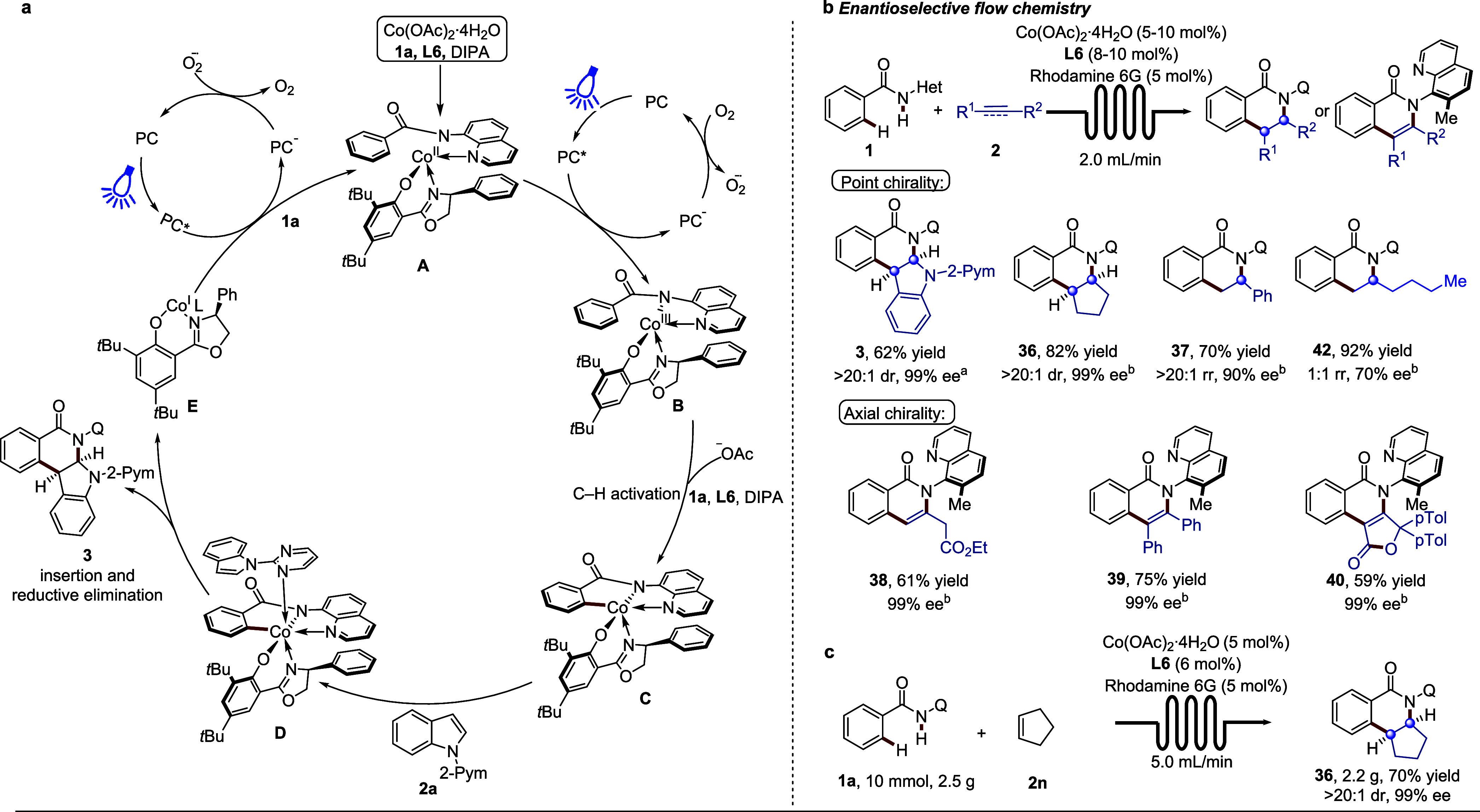
Flow photochemistry, scale-up reaction, and catalytic cycle. (a) Proposed catalytic cycle. (b) Flow photochemistry ^a^**1** (0.30 mmol, 1.0 equiv), **2** (0.45 mmol, 1.5 equiv), rhodamine 6G (0.03 mmol, 10 mol %), Co(OAc)_2_•4H_2_O (0.03 mmol, 10 mol %), **L6** (0.03 mmol, 10 mol %), DIPA (0.4 mmol, 1.3 equiv), TFE (2.0 mL), DCE (0.5 mL), rt, air, 48 h, Substrate **1a** is added to the reaction in three separate additions at 8 h intervals. ^b^**1** (0.30 mmol, 1.0 equiv), **2** (0.45 mmol, 1.5 equiv), rhodamine 6G (0.03 mmol, 10 mol %), Co(OAc)_2_•4H_2_O (0.015 mmol, 5 mol %), **L6** (0.024 mmol, 8 mol %), NaOTf (0.15 mmol, 0.5 equiv), TFE (2.0 mL), DCE (0.5 mL), rt, air, 16–48 h. (c) Gram-scale by flow photochemistry. **1a** (10 mmol, 1.0 equiv), **2** (15 mmol, 1.5 equiv), rhodamine 6G (5 mol %), Co(OAc)_2_•4H_2_O (5 mol %), **L6** (6 mol %), NaOTf (5 mmol, 0.5 equiv), TFE (60 mL), DCE (15 mL), rt, air, 24 h.

Based on our mechanistic investigations and previous strategies,^47,48^ a plausible mechanism is proposed in Figure 5a. Initially, the photoexcited rhodamine 6G oxidizes cobalt(II) in the presence of **1a**, **L6**, and DIPA, leading to the formation of the chiral octahedral cobalt(III) complex **B**. Subsequent an enantio-determining base-assisted C–H activation furnishes cobaltacycle intermediate **C**. Indole **2a** then undergoes a regioselective migratory insertion with the chiral cobalt(III) complex **D** with the assistance of pyrimidine guidance, followed by a reductive elimination step to afford the desired product **3** and cobalt(I) species **E**. The cobalt(I) species is reoxidized to cobalt(II) by rhodamine 6G under irradiation in the presence of aerial oxygen, thereby regenerating the active cobalt(II) catalyst.

### Practicality Studies

To demonstrate the synthetic utility and address the challenges often met during scale-up, we explored the applicability of this photochemical transformation in flow.^49,50^ The reaction was successfully carried out under continuous flow conditions, resulting in the formation of **3** with a yield of 62%, >20:1 dr, and 99% ee (Figure 5b). We extended our investigations to other alkenes, including cyclopentene and styrene, which yielded the corresponding products **36** and **37** in good yields and excellent enantioselectivities. Importantly, the efficacy of the cobalt catalyst was mirrored by a TON of 16.4 for **36** with a reduced cobalt catalyst loading of 5 mol %. It is worth noting that this continuous flow condition was also effective for constructing C–N axial chirality using allenes and alkynes (**38**, 61% yield, 99% ee; **39**, 75% yield, 99% ee; and **40**, 59% yield, 99% ee). Unactivated alkenes proved to be viable as well, albeit with somewhat reduced enantioselectivity (**42**). To further demonstrate the practicality of the continuous flow strategy, a gram-scale reaction was performed with a reduced loading of rhodamine 6G, Co(OAc)_2_·4H_2_O, and **L6**. This resulted in the successful synthesis of **36** in good yield and excellent enantioselectivity (Figure 5c), highlighting the scalability and robustness of the continuous flow cobaltaphotoredox catalysis.

## Conclusion

We have developed the first enantioselective cobaltaphotoredox-catalyzed C–H activation. This dearomatization of indoles enabled regioselective and stereoselective C–2 and C–3 dual functionalization and provided an efficient, straightforward, and highly enantioselective route for the assembly of chiral indolo[2,3-*c*]isoquinolin-5-ones. The success of our strategy highlights the potential of photochemical 3d-transition metal-catalyzed enantioselective C–H activation. The robustness was demonstrated through enantioselective annulations of alkenes, allenes, and alkynes under continuous photoflow conditions, resulting in the synthesis of compounds with central and axial chirality. We anticipate that this strategy sets the stage for photoinduced 3d-transition metal-catalyzed enantioselective C–H activation in its broadest sense.

## Supporting Information Available

The authors declare that the data supporting the findings of this study are available within the paper and the Supporting Information files. Source data are provided in this paper. All other requests for materials and information should be addressed to the corresponding authors. Crystallographic data for the structures reported in this article have been deposited at the Cambridge Crystallographic Data Centre, under deposition numbers CCDC 2282475 (**4**) and 2282491 (**35**). Copies of the data can be obtained free of charge via https://www.ccdc.cam.ac.uk/structures/. Additional experimental details, materials, and methods, including photographs of the experimental setup, computational details, crystallographic data, NMR spectra, and HPLC spectra (PDF)

## Abbreviations

Pym: pyrimidine
Q: 8-quinolinyl
PC: photocatalyst
TFE: 2,2,2-trifluoroethanol
DCE: 1,2-dichloroethane
TEA: triethylamine
DIPA: diisopropylamine
KIE: kinetic isotope effect
CV: cyclic voltammogram
HRMS: high-resolution mass spectrometry
NMR: nuclear magnetic resonance
CDA: cross-dehydrogenative arylation
VTNA: variable time normalization analysis
