# Supplementary material for: Enantioselective Cobaltaphotoredox-Catalyzed C–H Activation
Source: J Am Chem Soc. 2024 Aug 15;146(34):24105–13. doi: 10.1021/jacs.4c08459 (PMC11363020; doi:10.1021/jacs.4c08459)
Supplement: Supplementary file 1 — ja4c08459_si_001.pdf [file ja4c08459_si_001.pdf]

**Electronic Supporting Information**

# Enantioselective Cobaltaphotoredox Catalyzed C−H Activation

*Yang Xu, Ye Lin, Simon L. Homölle, João C. A. Oliveira and Lutz Ackermann**

Wöhler-Research Institute for Sustainable Chemistry (WISCh), Georg-August-Universität Göttingen, Tammannstraße 2, 37077 Göttingen, Germany.

Lutz.Ackermann@chemie.uni-goettingen.de

# Contents

1 General Remarks ....................................3

2 Synthesis of Substrates ....................................4

3 Optimization of Reaction Conditions ....................................5

4 Experimental ....................................14

5 Characterization data ....................................18

6 Mechanistic Studies ....................................58

References ....................................119

X-Ray Crystallographic Data ....................................121

NMR Spectra ....................................125

## General Remarks

Catalytic reactions were performed in 10 mL vial using a Kessil PR160L photoreactor with 450 nm irradiation. The reaction temperature was measured by digital thermometer PCE-T 390, which was in the range of 30 to 32 °C. Solvents for column chromatography and extraction (EtOAc, *n*-hexane, DCM) were distilled prior to their use. Routine TLC analysis was carried out on aluminium sheets coated with silica gel 60 F254, 0.2 mm thickness. Plates were analyzed using a 254 nm UV lamp. Chromatography was carried out on Merck silica gel 60 (40–63 µm). The substrates were either purchased directly from commercial suppliers or prepared according to previously reported procedures, if not noted otherwise. All other reagents and solvents used in this study were purchased from commercial sources and used as received. NMR spectra were recorded on a Varian Mercury VX 300 or Bruker Avance III 400 in the solvent indicated; chemical shifts (δ) are given in ppm relative to the residual solvent peak. All IR spectra were recorded on a Bruker FT-IR Alpha-P device. EI-MS spectra were recorded on Jeol AccuTOF at 70eV, ESI-MS spectra on Bruker MicrOTOF and maXis. HPLC chromatograms were recorded on an Agilent 1290 Infinity using CHIRALPAK® IA-3, IB-3, IC-3, ID-3, IE-3, IF-3, AD-3, OD-3 and OJ-3 columns (3.0 µm particle size; Ø: 4.6 mm and 250 mm length). Optical rotations were measured with Anton Paar MCP 150 at 20 °C under a Na/Hg lamp, λ = 589 nm (c in g/100 mL). Values were denoted as specific rotations: $[\alpha]_D^{20}$. X-ray diffraction experiments for the compounds analyzed were carried out at 100(2) K on a Bruker D8 Venture four-circle-diffractometer from Bruker AXS GmbH. M. p.: Stuart melting point apparatus SMP3, Barloworld Scientific, values are uncorrected. Absorption spectra were measured on a Jasco V-770 spectrophotometer. CV studies were performed using a Metrohm Autolab PGSTAT204 workstation and Nova 2.1 software. Headspace analysis of the S4 reaction mixture was performed on an Agilent 7890B GC System using a Thermal Conductivity Detector and a 5Å MS column.

# Synthesis of Substrates

The amides **1a-1u**[1] were synthesized according to literature procedure.

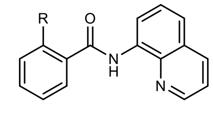

**1a**, R = H
**1b**, R = Me
**1c**, R = $CF_3$
**1d**, R = F
**1e**, R = Cl
**1f**, R = Br

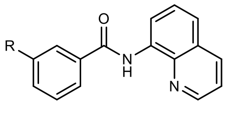

**1g**, R = Me
**1h**, R = Ph
**1i**, R = OMe

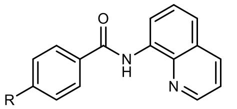

**1j**, R = Me
**1k**, R = OAc
**1l**, R = Cl

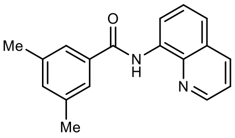

**1m**

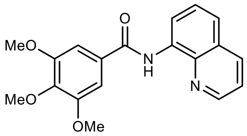

**1n**

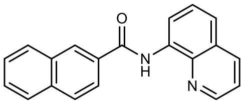

**1o**

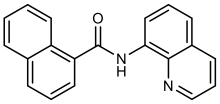

**1p**

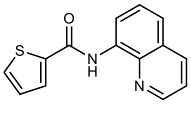

**1q**

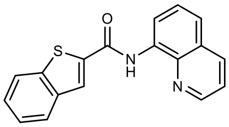

**1r**

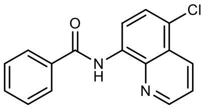

**1s**

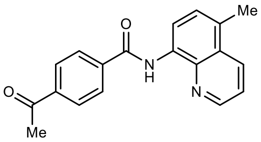

**1t**

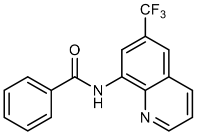

**1u**

The indoles **2a-2m**,[2-3] allene **2p**[4] and alkyne **2r**[5] were synthesized according to literature procedure.

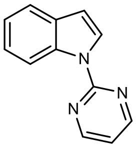

**2a**

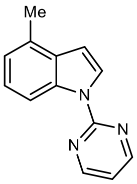

**2b**

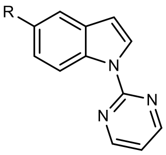

**2c**, R = Me
**2d**, R = Cl
**2e**, R = Br
**2f**, R = OBn
**2g**, R = OMe

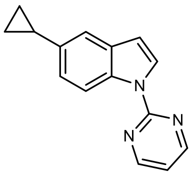

**2h**

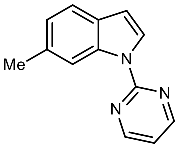

**2i**

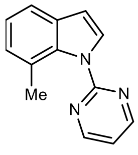

**2j**

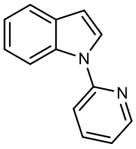

**2k**

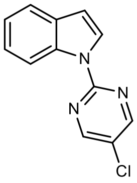

**2l**

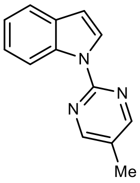

**2m**

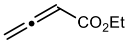

**2p**

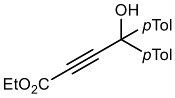

**2r**

# Optimization of Reaction Conditions

**Table S1.** Screening of chiral ligands.

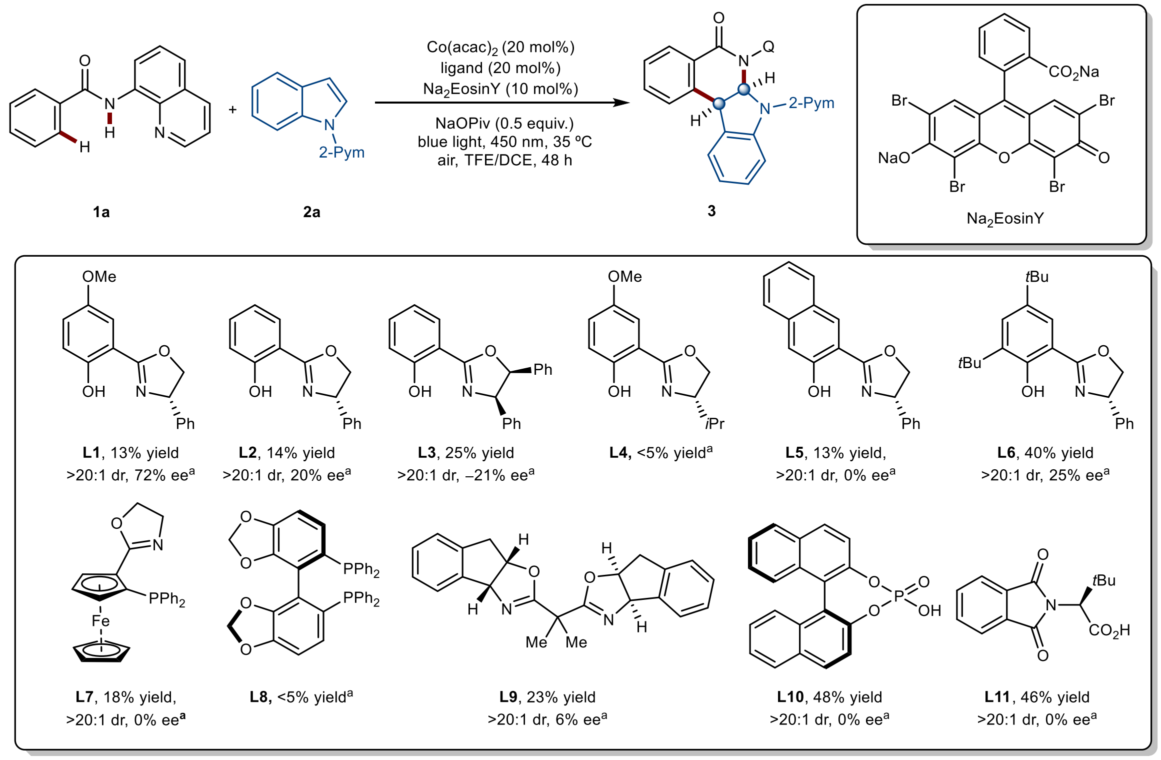


Reaction conditions: **1a** (0.15 mmol, 1.0 equiv.), **2a** (0.15 mmol, 1.0 equiv.), $Na_2$EosinY (0.015 mmol, 10 mol%), $Co(acac)_2$ (0.03 mmol, 20 mol%), ligand (0.03 mmol, 20 mol%), NaOPiv (0.075 mmol, 0.5 equiv.), TFE (2.0 mL), DCE (0.5 mL), 35 °C, 48 h. Yields were determined by $^1$H NMR using 1,3,5-trimethoxybenzene as the internal standard; isolated yields after column chromatography are shown in parentheses. The dr value was determined by $^1$H NMR analysis. The ee value was determined by chiral high-performance liquid chromatography (HPLC) analysis.

**Table S2.** Screening of cobalt salts.

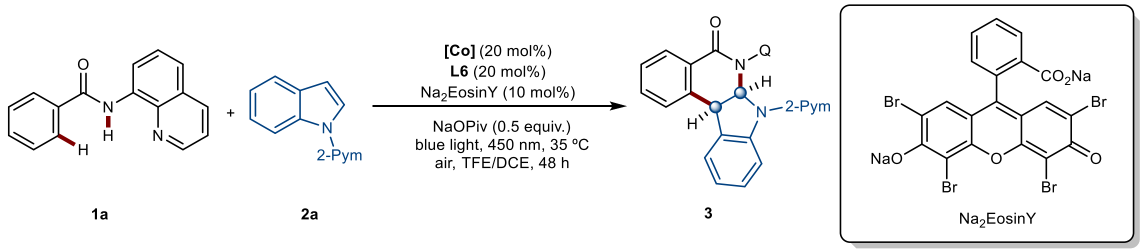


| Entry | [Co] | Yield(%) | dr | ee(%) |
|---|---|---|---|---|
| 1 | $Co(acac)_2$ | 40 | >20:1 | 25 |
| 2 | $Co(OAc)_2 \cdot 4H_2O$ | 23 | >20:1 | 99 |
| 3 | $Co(BF_4)_2 \cdot 4H_2O$ | 19 | >20:1 | 99 |
| 4 | $CoBr_2$ | 14 | >20:1 | 99 |

Reaction conditions: **1a** (0.15mmol, 1.0 equiv.), **2a** (0.15 mmol, 1.0 equiv.), $Na_2$EosinY (0.015 mmol, 10 mol%), [Co] (0.03 mmol, 20 mol%), **L6** (0.03 mmol, 20 mol%), NaOPiv (0.075 mmol, 0.5 equiv.), TFE (2.0 mL), DCE (0.5 mL), 35 °C, 48 h. Yields were determined by $^1$H NMR using 1,3,5-trimethoxybenzene as the internal standard; isolated yields after column chromatography are shown in parentheses. The dr value was determined by $^1$H NMR analysis. The ee value was determined by HPLC analysis.

**Table S3.** Screening of chiral ligands (based on $Co(OAc)_2 \cdot 4H_2O$).

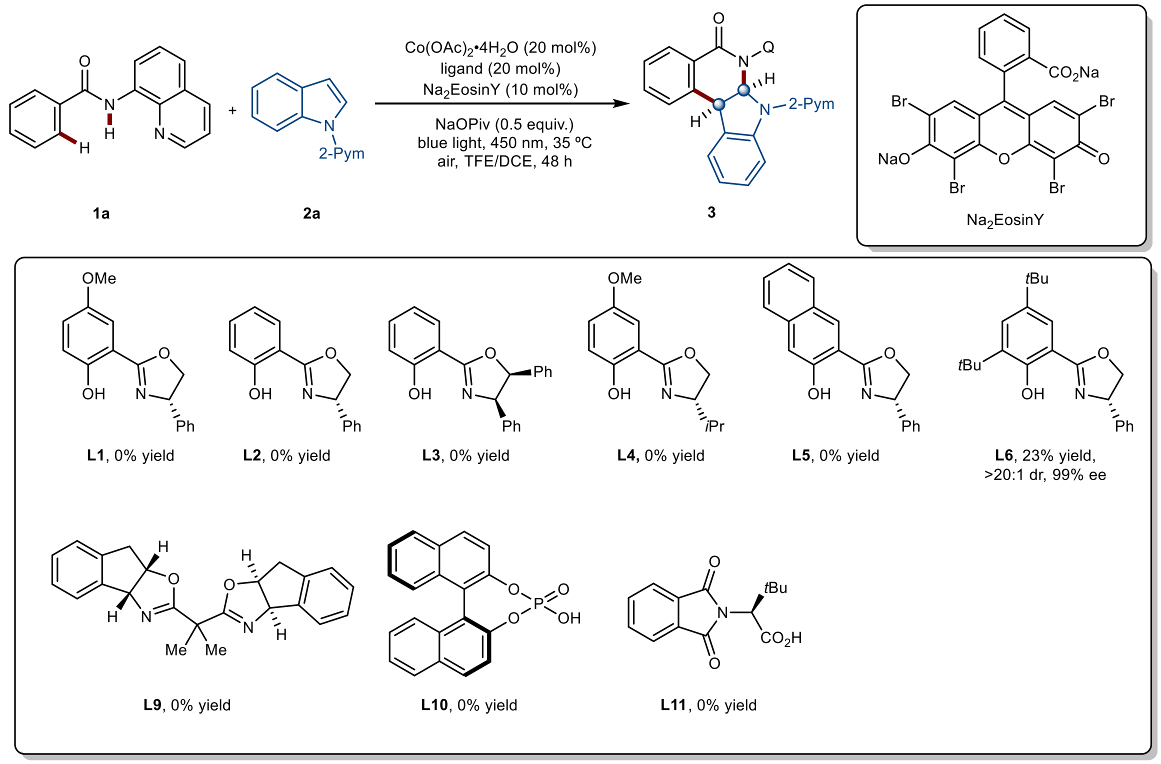


Reaction conditions: **1a** (0.15 mmol, 1.0 equiv.), **2a** (0.15 mmol, 1.0 equiv.), Na2EosinY (0.015 mmol, 10 mol%), $Co(OAc)_2 \cdot 4H_2O$ (0.03 mmol, 20 mol%), ligand (0.03 mmol, 20 mol%), NaOPiv (0.075 mmol, 0.5 equiv.), TFE (2.0 mL), DCE (0.5 mL), 35 °C, 48 h. Yields were determined by $^1H$ NMR using 1,3,5-trimethoxybenzene as the internal standard; isolated yields after column chromatography are shown in parentheses. The dr value was determined by $^1H$ NMR analysis. The ee value was determined by chiral high-performance liquid chromatography (HPLC) analysis.

**Table S4.** Screening of photocatalysts and the loading of NaOPiv.

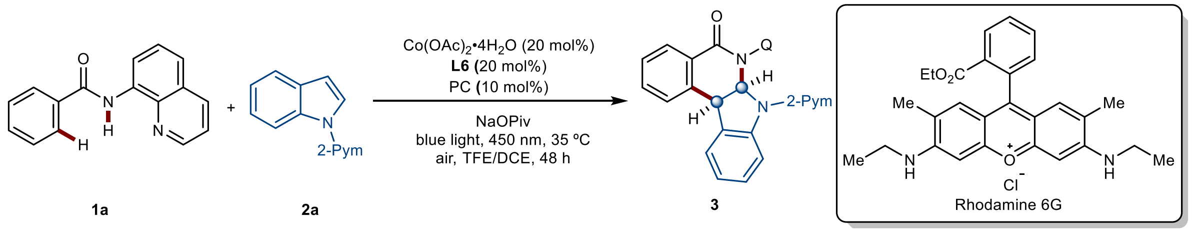


| Entry | NaOPiv | PC | Yield (%) | dr | ee (%) |
|---|---|---|---|---|---|
| 1 | 0.5 equiv. | $Na_2$EosinY | 23 | >20:1 | 99 |
| 2 | 1.3 equiv. | $Na_2$EosinY | 26 | >20:1 | 99 |
| 3 | 1.3 equiv. | Rose Bengal | 29 | >20:1 | 99 |
| 4 | 1.3 equiv. | Rhodamine 6G | 33 | >20:1 | 99 |

Reaction conditions: **1a** (0.15 mmol, 1.0 equiv.), **2a** (0.15 mmol, 1.0 equiv.), PC (0.015 mmol, 10 mol%), $Co(OAc)_2{\cdot}4H_2O$ (0.03 mmol, 20 mol%), **L6** (0.03 mmol, 20 mol%), NaOPiv (0.5-1.3 equiv.), TFE (2.0 mL), DCE (0.5 mL), 35 °C, 48 h. Yields were determined by $^1$H NMR using 1,3,5-trimethoxybenzene as the internal standard; isolated yields after column chromatography are shown in parentheses. The dr value was determined by $^1$H NMR analysis. The ee value was determined by HPLC analysis.

**Table S5.** Screening of base.[a]

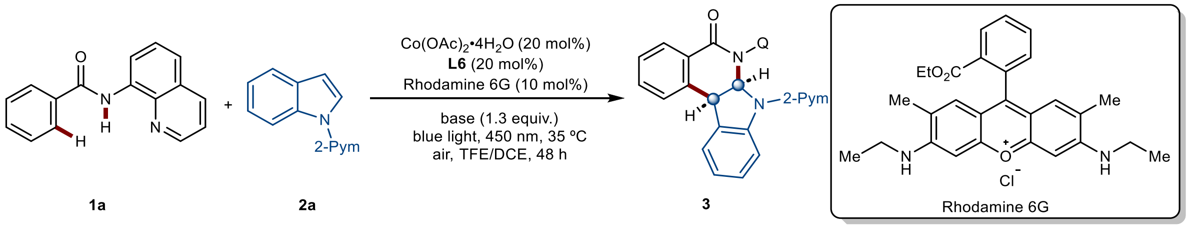


| Entry | Base | Yield (%) | dr | ee (%) |
|---|---|---|---|---|
| 1 | NaOPiv | 33 | >20:1 | 99 |
| 2 | TEA | 37 | >20:1 | 99 |
| 3 | DIPA | 65 | >20:1 | 99 |
| 4 | DIPEA | 27 | >20:1 | 99 |
| 5 | TMG | 35 | >20:1 | 99 |
| 6 | DBN | 42 | >20:1 | 99 |
| 7[b] | DIPA | 28 | >20:1 | 99 |
| 8[c] | DIPA | 82 | >20:1 | 99 |

Reaction conditions: [a]**1a** (0.15 mmol, 1.0 equiv.), **2a** (0.15 mmol, 1.0 equiv.), Rhodamine 6G (0.015 mmol, 10 mol%), $Co(OAc)_2 \cdot 4H_2O$ (0.03 mmol, 20 mol%), **L6** (0.03 mmol, 20 mol%), base (1.3 equiv.), TFE (2.0 mL), DCE (0.5 mL), 35 °C, 48 h. Yields were determined by $^1$H NMR using 1,3,5-trimethoxybenzene as the internal standard; isolated yields after column chromatography are shown in parentheses. The dr value was determined by $^1$H NMR analysis. The ee value was determined by HPLC analysis. [b]TFE (4.0 mL), DCE (1.0 mL). [c]TFE (1.0 mL), DCE (0.25 mL). TEA = triethylamine. DIPA = diisopropylamine. DIPEA = *N*,*N*-diisopropylethylamin. TMG = 1,1,3,3-Tetramethylguanidine. DBN = 1,5-diazabicyclo-[4.3.0]-non-5-en.

**Table S6.** Screening of Salox ligands.[a]

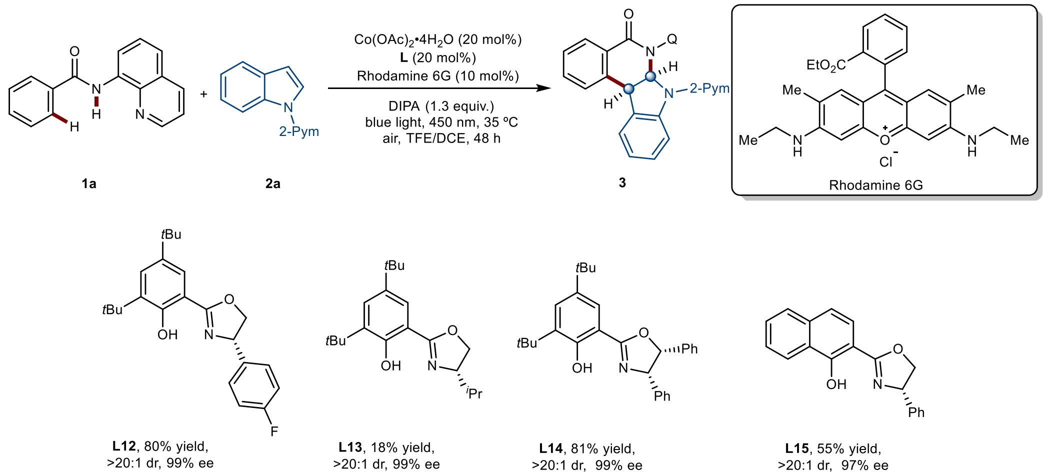


Reaction conditions: [a]**1a** (0.15 mmol, 1.0 equiv.), **2a** (0.15 mmol, 1.0 equiv.), Rhodamine 6G (0.015 mmol, 10 mol%), $Co(OAc)_2 \cdot 4H_2O$ (0.03 mmol, 20 mol%), **L6** (0.03 mmol, 20 mol%), DIPA (1.3 equiv.), TFE (2.0 mL), DCE (0.5 mL), 35 °C, 48 h. Yields were determined by $^1H$ NMR using 1,3,5-trimethoxybenzene as the internal standard; The dr value was determined by $^1H$ NMR analysis. The ee value was determined by HPLC analysis.

**Table S7.** Screening of metallic photocatalysts.[a]

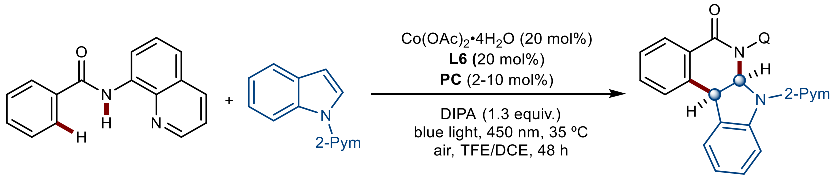


| Entry | Variation from standard conditions | Yield (%) | dr | ee (%) |
|---|---|---|---|---|
| 1 | none | 82 | >20:1 | 99 |
| 2 | Rhodamine 6G (5 mol%) | 72 | >20:1 | 99 |
| 3[b] | $Ru(bpy)_3Cl_3 \cdot 6H_2O$ | 22 | >20:1 | 95 |
| 4[b] | $[Ru(bpz)_3][PF_6]_2$ | <5 | >20:1 | - |
| 5[b] | $[Ir\{dFCF_3ppy\}_2(bpy)]PF_6$ | 24 | >20:1 | 94 |
| 6[b] | $Ir(ppy)_3$ | 20 | >20:1 | 95 |

Reaction conditions: [a]**1a** (0.15 mmol, 1.0 equiv.), **2a** (0.15 mmol, 1.0 equiv.), Rhodamine 6G (0.015 mmol, 10 mol%), $Co(OAc)_2 \cdot 4H_2O$ (0.03 mmol, 20 mol%), **L6** (0.03 mmol, 20 mol%), DIPA (1.3 equiv.), TFE (2.0 mL), DCE (0.5 mL), 35 °C, 48 h. [b]PC (2 mol%). Yields were determined by $^1H$ NMR using 1,3,5-trimethoxybenzene as the internal standard; The dr value was determined by $^1H$ NMR analysis. The ee value was determined by HPLC analysis.

**Table S8.** Screening the loading of the catalyst.[a]

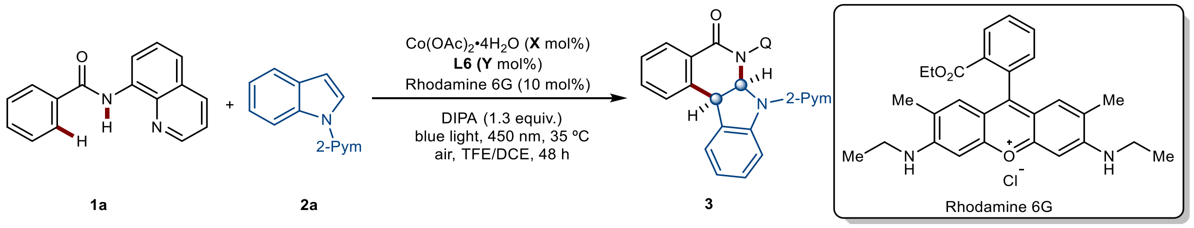


| Entry | Variation from standard conditions | Yield (%) | dr | ee (%) |
|---|---|---|---|---|
| 1 | none (X = 20, Y = 20) | 82 | >20:1 | 99 |
| 2 | X = 10, Y = 20 | 56 | >20:1 | 99 |
| 3 | X = 10, Y = 10 | 53 | >20:1 | 99 |
| 4 | X = 5, Y = 10 | 33 | >20:1 | 99 |
| 5 | X = 5, Y = 10, 70 °C | 25 | >20:1 | 99 |
| 6 | X = 10, Y = 10, **2a** (1.5 equiv.) | 68 | >20:1 | 99 |
| 8 | X = 5, Y = 10, **2a** (1.5 equiv.) | 35 | >20:1 | 99 |
| 9 | X = 5, Y = 10, **2a** (1.5 equiv.), blue light (440 nm) | 32 | >20:1 | 99 |
| 10 | X = 5, Y = 10, **2a** (1.5 equiv.), blue light (427 nm) | 28 | >20:1 | 99 |
| 11[b] | X = 10, Y = 10, **2a** (1.5 equiv.) | 75 | >20:1 | 99 |
| 12[b] | X = 5, Y = 10, **2a** (1.5 equiv.) | 57 | >20:1 | 99 |

Reaction conditions: [a]**1a** (0.15 mmol, 1.0 equiv.), **2a** (0.15 mmol, 1.0 equiv.), Rhodamine 6G (0.015 mmol, 10 mol%), $Co(OAc)_2 \cdot 4H_2O$ (0.03 mmol, 20 mol%), **L6** (0.03 mmol, 20 mol%), DIPA (1.3 equiv.), TFE (2.0 mL), DCE (0.5 mL), 35 °C, 48 h. [b]Substrate **1a** is introduced to the reaction in three separate additions at eight-hour intervals. Yields were determined by $^1H$ NMR using 1,3,5-trimethoxybenzene as the internal standard; The dr value was determined by $^1H$ NMR analysis. The ee value was determined by HPLC analysis.

**Table S9.** Control experiments.

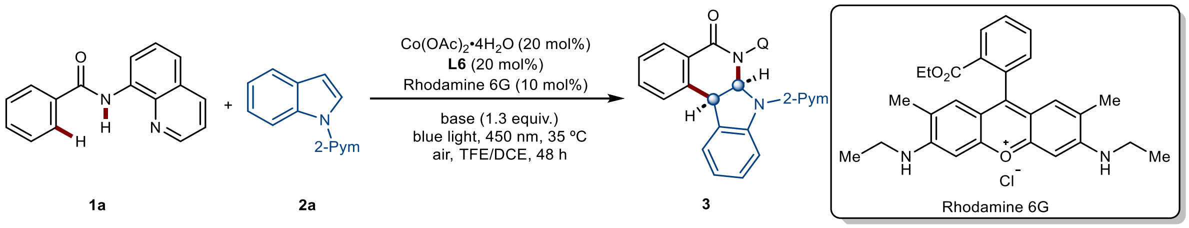


**1a** **2a** **3**

| **Entry** | Variation from standard conditions | **Yield (%)** | **dr** | **ee (%)** |
|---|---|---|---|---|
| 1 | none | 82 | >20:1 | 99 |
| 2 | without **L6** | 0 | - | - |
| 3 | without DIPA | 12 | >20:1 | 99 |
| 4 | $N_2$ instead of air | 0 | - | - |
| 5 | $O_2$ instead of air | 81 | >20:1 | 99 |

Standard conditions: **1a** (0.15 mmol, 1.0 equiv.), **2a** (0.15 mmol, 1.0 equiv.), Rhodamine 6G (0.015 mmol, 10 mol%), $Co(OAc)_2 \cdot 4H_2O$ (0.03 mmol, 20 mol%), **L6** (0.03 mmol, 20 mol%), DIPA (1.3 equiv.), TFE (1.0 mL), DCE (0.25 mL), 35 °C, 48 h. Yields were determined by $^1$H NMR using 1,3,5-trimethoxybenzene as the internal standard; isolated yields after column chromatography are shown in parentheses. The dr value was determined by $^1$H NMR analysis. The ee value was determined by HPLC analysis.

# Experimental

## 4.1 General Procedure A for the Synthesis of Products 3-35

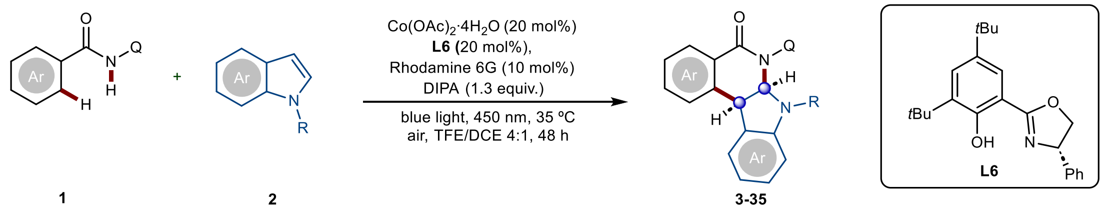


A 10 mL vial was charged with the amide **1** (0.15 mmol, 1.0 equiv.), indole **2** (0.15 mmol, 1.0 equiv.), $Co(OAc)_2·4H_2O$ (7.5 mg, 20 mol%), **L6** (10.6 mg, 20 mol%), Rhodamine 6G (7.2 mg, 10 mol%), DIPA (27 µL, 0.20 mmol, 1.3 equiv.) and a teflon-coated magnetic stirring bar. Then DCE (0.25 mL) and TFE (1 mL) were added. The vial was stirred at room temperature under blue LEDs (450 nm) for 48 h (**Fig. S1**). After completion of the reaction, the solvent was then removed under vacuum and the residue was purified by column chromatography on silica gel with *n*-hexane /ethyl acetate (3:1-1:1) as eluent to give corresponding product **3-35**. Racemic samples of compounds **3** were prepared with racemic **L6**.

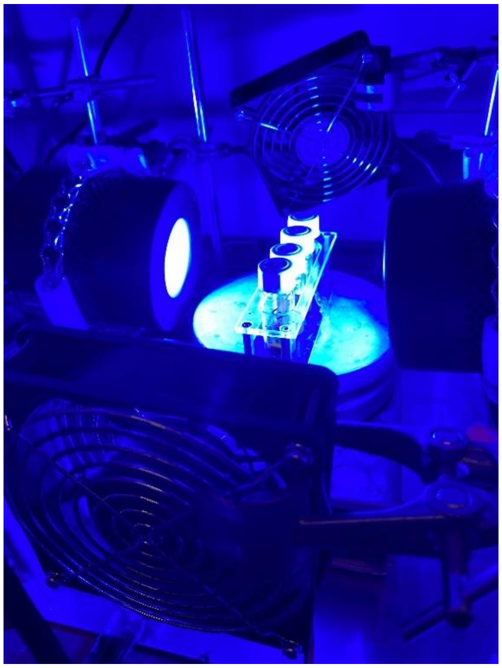

**Figure S1.** Pictures of the photochemical set up used.

## 4.2 Gram-Scale Reaction for the Synthesis 3

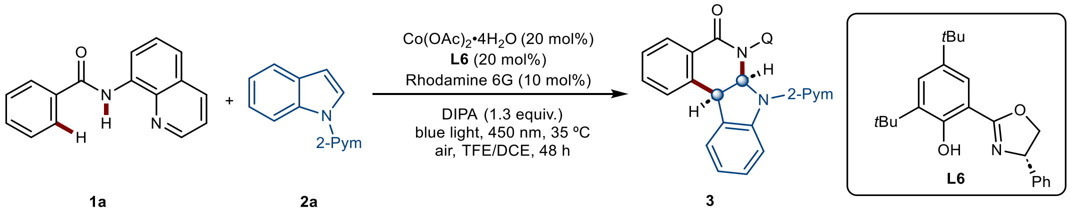


A 250 mL flask was charged with the amide **1a** (1.24g, 5.0 mmol, 1.0 equiv.), indole **2a** (0.975g, 5.0 mmol, 1.0 equiv.), $Co(OAc)_2{\cdot}4H_2O$ (249 mg, 20 mol%), **L6** (351 mg, 20 mol%), Rhodamine 6G (240 mg, 10 mol%), DIPA (911 μL, 6.5 mmol, 1.3 equiv.) and a teflon-coated magnetic stirring bar. Then DCE (8 mL) and TFE (32 mL) were added. The vial was stirred at room temperature under blue LEDs (450 nm) for 48 h. After completion of the reaction, the solvent was then removed under vacuum and the residue was purified by column chromatography on silica gel with *n*-hexane /ethyl acetate (1:1) as eluent to give corresponding product **3** as a buff yellow solid (1.7g, 65% yield, >20:1 dr, 99% ee).

## 4.3 General Procedure B for the Synthesis of 3 in Continuous Photoflow

All flow experiments were performed using a self-assembled flow platform: take a 1m long piece of PTFE tubing (outer diameter 1/16”, inner diameter 1/32”) and wrap it around an 18 mL test tube 25 times.

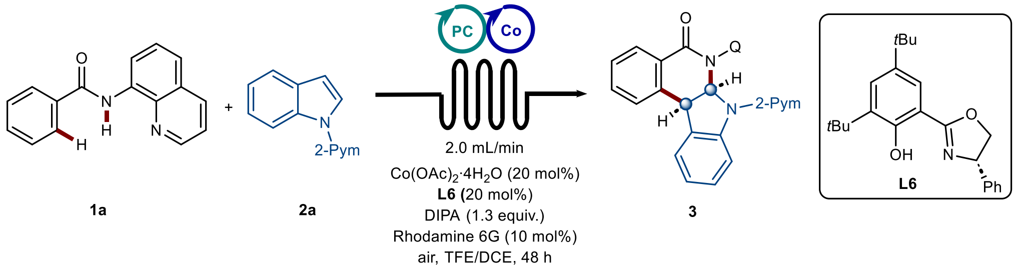


A 25 mL flask was charged with the indole **2a** (0.45 mmol, 1.5 equiv.), $Co(OAc)_2{\cdot}4H_2O$ (15 mg, 20 mol%), **L6** (21.2 mg, 20 mol%), Rhodamine 6G (14.4 mg, 10 mol%), DIPA (54 μL, 0.39 mmol, 1.3 equiv.) and a teflon-coated magnetic stirring bar. Then DCE (0.5 mL) and TFE (2 mL) were added. Substrate **1a** (0.30 mmol) was added to the reaction in three separate additions at eight-hour intervals. The solution was pumped to the flow reactor by a peristaltic pump with a flow speed of 2.0 mL/min. The reaction was performed at room temperature with blue LEDs (450 nm) for 48 h

(**Fig. S2**) After completion of the reaction, the solvent was then removed under vacuum and the residue was purified by column chromatography on silica gel with *n*-hexane /ethyl acetate (1:1) as eluent to give **3** as a buff yellow solid (62% yield, >20:1 dr, 99% ee).

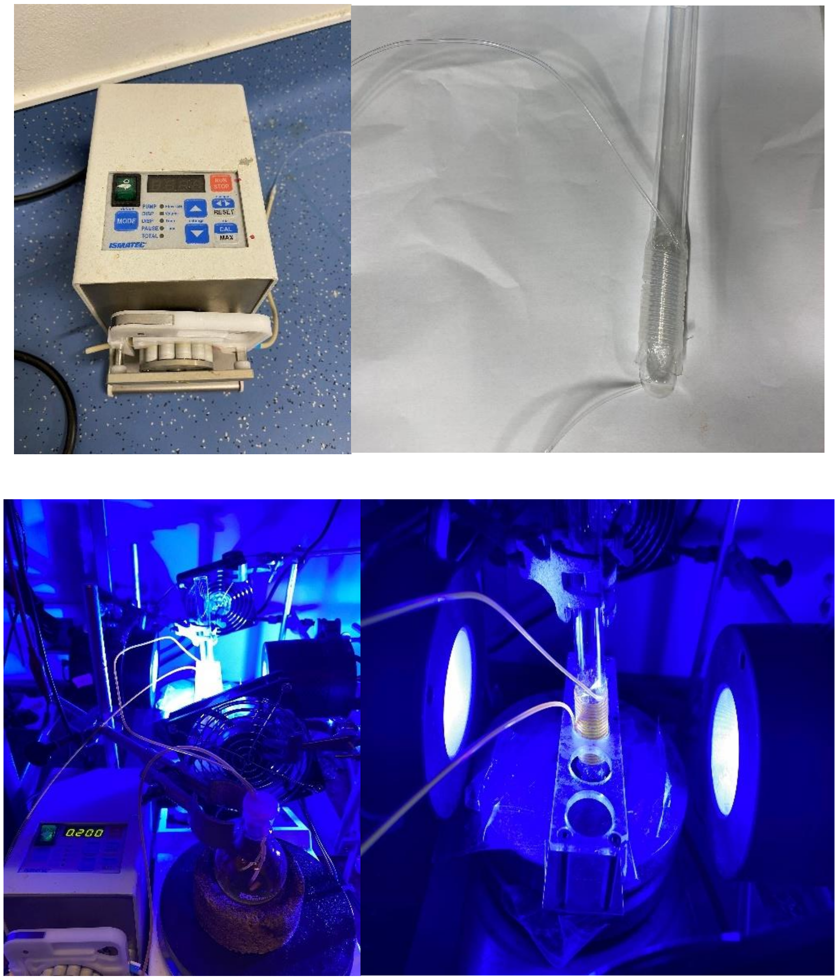

**Figure S2.** Pictures of the flow photochemical set up used.

### 4.4 General Procedure C for the Synthesis 36-40 in Continuous Flow Photochemistry

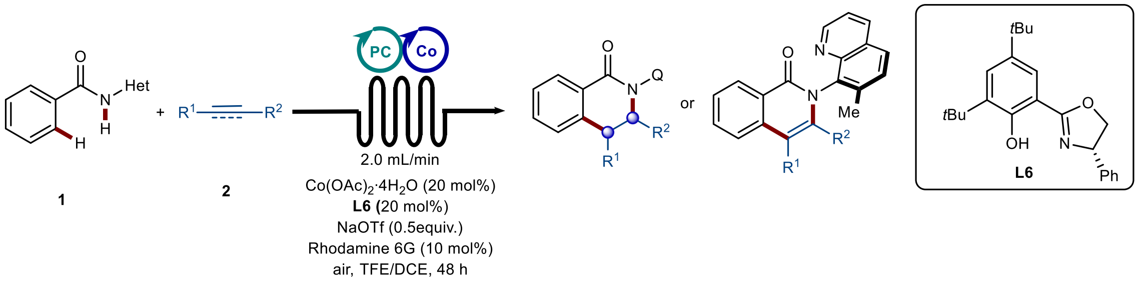

We tried to make **36** using DIPA as a base, but the yield was only <5%. So next we used NaOTf instead of DIPA. A 25 mL flask was charged with the amide **1** (0.30 mmol, 1.0 equiv.), alkene/alkyne/allene **2** (0.45 mmol, 1.5 equiv.), $Co(OAc)_2{\cdot}4H_2O$ (3.8 mg, 5 mol%), **L6** (8.5 mg, 8 mol%), Rhodamine 6G (14.4 mg, 10 mol%), NaOTf (25.8 mg, 0.15 mmol, 0.5 equiv.) and a teflon-coated magnetic stirring bar. Then DCE (0.5 mL) and TFE (2 mL) were added. The solution was pumped to the flow reactor by a peristaltic pump with a flow speed of 2.0 mL/min. The reaction was performed at room temperature with blue LEDs (450 nm) for 16-48 h (**Fig. S2**). After completion of the reaction, the solvent was then removed under vacuum and the residue was purified by column chromatography on silica gel with *n*-hexane /ethyl acetate (3:1-1:1) as eluent to give corresponding product **36-40**.

### 4.5 Gram-Scale Continuous Photoflow Synthesis of 36

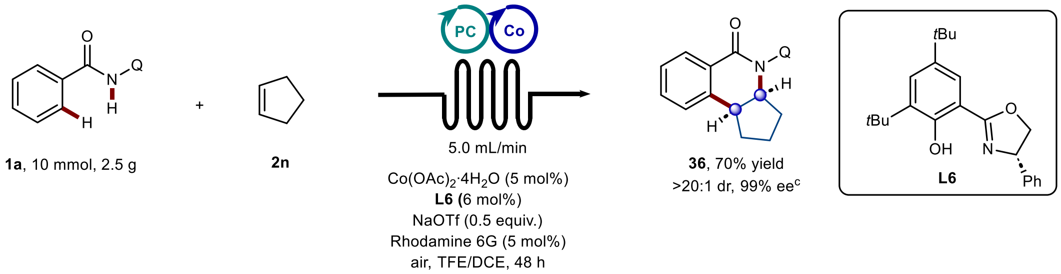


A 25 mL flask was charged with the amide **1** (10.0 mmol, 1.0 equiv.), **2n** (15.0 mmol, 1.5 equiv.), $Co(OAc)_2{\cdot}4H_2O$ (124 mg, 5 mol%), **L6** (211 mg, 6 mol%), Rhodamine 6G (240 mg, 5 mol%), NaOTf (860 mg, 5.0 mmol, 0.5 equiv.) and a teflon-coated magnetic stirring bar. Then DCE (15 mL) and TFE (60 mL) were added. The solution was pumped to the flow reactor by a peristaltic pump with a flow speed of 5.0 mL/min. The reaction was performed at room temperature with blue LEDs (450 nm) for 24 h (**Fig. S2**). After completion of the reaction, the solvent was then removed under vacuum and the residue was purified by column chromatography on silica gel with *n*-hexane/ethyl acetate (3:1) as eluent to give corresponding product **36** as a buff yellow solid (2.2 g, 70% yield, >20:1 dr, 99% ee).

## Characterization data

**(6a*R*,11b*R*)-7-(Pyrimidin-2-yl)-6-(quinolin-8-yl)-6,6a,7,11b-tetrahydro-5*H*-indolo[2,3-*c*]isoquinolin-5-one (3)**

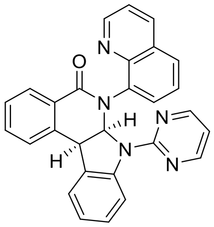

Prepared according to general procedure **A** on a 0.15 mmol scale, column chromatography (ethyl acetate/*n*-hexane = 2:1) afforded the title compound as a pale yellow sticky liquid (52 mg, 0.12 mmol, 79%), with >20:1 dr, 99% ee.

**$^{1}$H NMR** (400 MHz, $CDCl_3$) δ 8.97 (d, *J* = 3.0 Hz, 1H), 8.35 – 8.23 (m, 2H), 8.10 (d, *J* = 7.8 Hz, 1H), 7.75 – 7.61 (m, 3H), 7.62 – 7.47 (m, 4H), 7.45 – 7.32 (m, 2H), 7.21 – 7.12 (m, 2H), 7.07 – 6.98 (m, 2H), 6.23 (t, *J* = 4.7 Hz, 1H), 4.84 (d, *J* = 6.4 Hz, 1H) ppm;

**$^{13}$C NMR** (101 MHz, $CDCl_3$) δ 164.2 ($C_q$), 159.0 ($C_q$), 156.5 (CH), 150.4 (CH), 145.7 ($C_q$), 141.3 ($C_q$), 137.0 ($C_q$), 135.8 (CH), 134.9 ($C_q$), 133.9 ($C_q$), 132.5 (CH), 130.9 (CH), 129.6 (CH), 128.9 ($C_q$), 128.5 (CH), 128.4 (CH), 128.1 (CH), 128.0 (CH), 127.3 ($C_q$), 125.6 (CH), 123.9 (CH), 123.0 (CH), 121.1 (CH), 117.3 (CH), 111.8 (CH), 76.4 (CH), 44.4 (CH) ppm;

**IR** (ATR): $\tilde{\nu}$ = 1658, 1580, 1554, 1482, 1460, 1436, 1290, 795, 748, 698cm$^{-1}$;

**HRMS (ESI):** *m/z* $[M+H]^+$ calcd for $C_{28}H_{20}N_5O$: 442.1662; found: 442.1657;

**m.p.:** 137-138 °C;

**$[\alpha]^{D}_{20}$** = –218.6 (c = 0.50, $CHCl_3$);

**$R_t$** (ID-3 column, *n*-hexane/*i*-PrOH 50/50, 1.0 mL/min, 250.4 nm): tr(major) = 44.5 min, tr(minor) = 12.0 min, 99% ee.

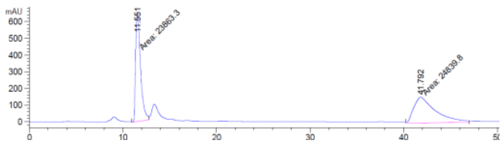

| Peak # | RetTime [min] | Type | Width [min] | Area [mAU*s] | Height [mAU] | Area % |
|---|---|---|---|---|---|---|
| 1 | 11.551 | MM | 0.6094 | 2.38633e4 | 652.59302 | 48.9975 |
| 2 | 41.792 | MM | 2.6782 | 2.48398e4 | 154.57999 | 51.0025 |

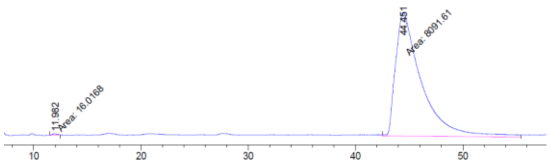

| Peak # | RetTime [min] | Type | Width [min] | Area [mAU*s] | Height [mAU] | Area % |
|---|---|---|---|---|---|---|
| 1 | 11.982 | MM | 0.5597 | 16.01684 | 4.76944e-1 | 0.1976 |
| 2 | 44.451 | MM | 2.7863 | 8091.61279 | 48.40052 | 99.8024 |

**(6a*R*,11b*R*)-4-Methyl-7-(pyrimidin-2-yl)-6-(quinolin-8-yl)-6,6a,7,11b-tetrahydro-5*H*-indolo[2,3-*c*]isoquinolin-5-one (4)**

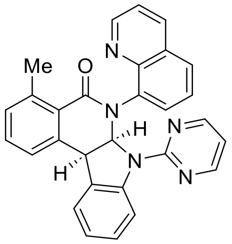

Prepared according to general procedure **A** on a 0.15 mmol scale, column chromatography (ethyl acetate/*n*-hexane = 2:1) afforded the title compound as a pale yellow sticky liquid (47 mg, 0.10 mmol, 69%), with >20:1 dr, 99% ee.

**$^1$H NMR** (400 MHz, $CDCl_3$) δ 9.02 (s, 1H), 8.36 (d, *J* = 7.9 Hz, 1H), 8.14 (d, *J* = 8.2 Hz, 1H), 7.72 (s, 2H), 7.60 (q, *J* = 7.2 Hz, 2H), 7.54 – 7.33 (m, 5H), 7.21 (s, 2H), 7.07 (s, 2H), 6.27 (s, 1H), 4.84 (d, *J* = 6.3 Hz, 1H), 2.76 (s, 3H) ppm;

**$^{13}$C NMR** (101 MHz, $CDCl_3$) δ 165.3 ($C_q$), 158.9 ($C_q$), 156.4 (CH), 150.3 (CH), 145.6 ($C_q$), 143.1 ($C_q$), 141.1 ($C_q$), 137.3 ($C_q$), 136.0 ($C_q$), 135.8 (CH), 134.4 ($C_q$), 132.3 (CH), 131.6 (CH), 131.2 (CH), 129.0 ($C_q$), 128.3 (CH), 127.9 (CH), 127.1 (CH), 125.6 (CH), 125.2 ($C_q$), 124.0 (CH), 123.0 (CH), 121.1 (CH), 117.1 (CH), 111.7 (CH), 75.7 (CH), 45.2 (CH), 23.6 ($CH_3$) ppm;

**IR** (ATR): $\tilde{\nu}$ = 1656, 1554, 1483, 1439, 1238, 1215, 1168, 1062, 794, 749 $cm^{-1}$;

**HRMS (ESI):** *m/z* $[M+H]^+$ calcd for $C_{29}H_{22}N_5O$: 456.1819; found: 456.1817;

**$[\alpha]^D_{20}$** = –290.4 (c = 0.50, $CHCl_3$);

**$R_t$** (ID-3 column, *n*-hexane/*i*-PrOH 70/30, 1.0 mL/min, 250.4 nm): tr(major) = 31.6 min, tr(minor) = 10.0 min, 99% ee.

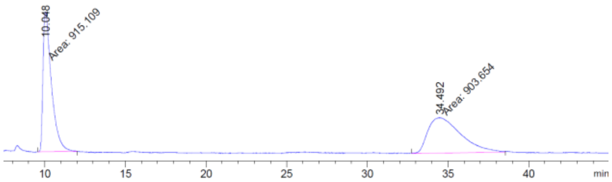


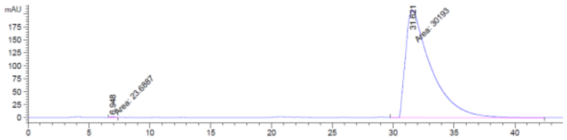


| Peak # | RetTime [min] | Type | Width [min] | Area [mAU*s] | Height [mAU] | Area % |
|---|---|---|---|---|---|---|
| 1 | 10.048 | MM | 0.6110 | 915.10876 | 24.96256 | 50.3149 |
| 2 | 34.492 | MM | 2.3880 | 903.65411 | 6.30683 | 49.6851 |

| Peak # | RetTime [min] | Type | Width [min] | Area [mAU*s] | Height [mAU] | Area % |
|---|---|---|---|---|---|---|
| 1 | 6.948 | MM | 0.3733 | 23.68869 | 1.05775 | 0.0784 |
| 2 | 31.621 | MM | 2.4280 | 3.01930e4 | 207.25674 | 99.9216 |

**(6a*R*,11b*R*)-7-(Pyrimidin-2-yl)-6-(quinolin-8-yl)-4-(trifluoromethyl)-6,6a,7,11b-tetrahydro-5*H*-indolo[2,3-*c*]isoquinolin-5-one (5)**

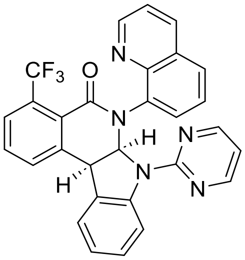


Prepared according to general procedure **A** on a 0.15 mmol scale, column chromatography (ethyl acetate/*n*-hexane = 2:1) afforded the title compound as a pale yellow sticky liquid (40 mg, 0.08 mmol, 52%), with >20:1 dr, 99% ee.

**$^{1}$H NMR** (400 MHz, $CDCl_3$) δ 8.99 (d, *J* = 4.2 Hz, 1H), 8.38 (d, *J* = 8.0 Hz, 1H), 8.12 (d, *J* = 8.2 Hz, 1H), 7.99 (t, *J* = 4.8 Hz, 1H), 7.82 (d, *J* = 4.7 Hz, 2H), 7.74 (d, *J* = 4.7 Hz, 2H), 7.60 (dd, *J* = 6.3, 3.4 Hz, 1H), 7.51 (d, *J* = 6.1 Hz, 1H), 7.44 (dd, *J* = 8.2, 5.5 Hz, 2H), 7.22 – 7.15 (m, 2H), 7.15 – 7.00 (m, 2H), 6.29 (t, *J* = 4.8 Hz, 1H), 4.90 (d, *J* = 6.1 Hz, 1H) ppm;

**$^{13}$C NMR** (101 MHz, $CDCl_3$) δ 161.5 ($C_q$), 158.8 ($C_q$), 156.5 (CH), 150.3 (CH), 145.4 ($C_q$), 141.1 ($C_q$), 137.3 ($C_q$), 136.4 ($C_q$), 135.8 (CH), 133.0 ($C_q$), 132.9 (CH), 131.8 (CH), 131.3 (q, $J_{CF}$ = 33.3 Hz, $C_q$), 131.1 (CH), 129.0 ($C_q$), 128.8 (CH), 128.2 (CH), 127.9 (q, $J_{CF}$ = 7.1 Hz, CH), 125.4 (CH), 123.7 (CH), 123.6 (q, $J_{CF}$ = 274.7 Hz, $C_q$), 123.1 (CH), 121.2 (CH), 117.2 (CH), 112.0 (CH), 75.4 (CH), 45.2 (CH) ppm;

**$^{19}$F NMR** (377 MHz, $CDCl_3$) δ –58.29 ppm;

**IR** (ATR): $\tilde{\nu}$ = 1680, 1580, 1484, 1434, 1383, 1157, 1133, 1080, 792, 750 $cm^{-1}$;

**HRMS (ESI):** *m/z* $[M+H]^+$ calcd for $C_{29}H_{19}F_3N_5O$: 510.1536; found: 510.1539;

**$[\alpha]^{D}_{20}$** = –192.0 (c = 0.50, $CHCl_3$);

**$R_t$** (ID-3 column, *n*-hexane/*i*-PrOH 70/30, 1.0 mL/min, 250.4 nm): tr(major) = 25.9 min, tr(minor) = 8.3 min, 99% ee.

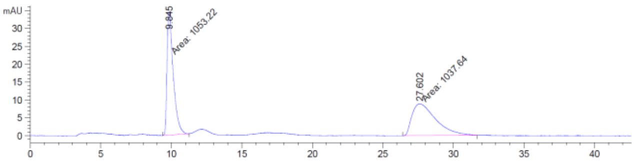


| Peak # | RetTime [min] | Type | Width [min] | Area [mAU*s] | Height [mAU] | Area % |
|---|---|---|---|---|---|---|
| 1 | 9.845 | MM | 0.5091 | 1053.22339 | 34.47668 | 50.3727 |
| 2 | 27.602 | MM | 1.9486 | 1037.63635 | 8.87520 | 49.6273 |

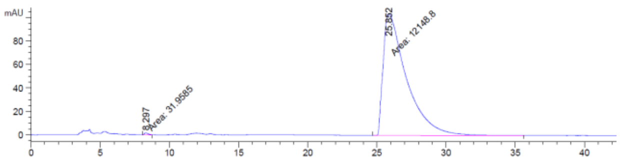


| Peak # | RetTime [min] | Type | Width [min] | Area [mAU*s] | Height [mAU] | Area % |
|---|---|---|---|---|---|---|
| 1 | 8.297 | MM | 0.3282 | 31.95855 | 1.62281 | 0.2624 |
| 2 | 25.852 | MM | 1.9536 | 1.21488e4 | 103.64334 | 99.7376 |

**(6a*R*,11b*R*)-4-Fluoro-7-(pyrimidin-2-yl)-6-(quinolin-8-yl)-6,6a,7,11b-tetrahydro-5*H*-indolo[2,3-*c*]isoquinolin-5-one (6)**

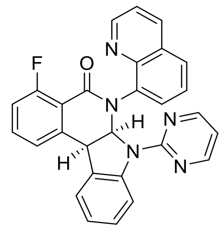

Prepared according to general procedure **A** on a 0.15 mmol scale, column chromatography (ethyl acetate/*n*-hexane = 2:1) afforded the title compound as a pale yellow sticky liquid (59 mg, 0.13 mmol, 85%), with >20:1 dr, 99% ee.

**$^{1}$H NMR** (400 MHz, $CDCl_3$) δ 8.99 (d, $J$ = 3.9 Hz, 1H), 8.32 (d, $J$ = 8.0 Hz, 1H), 8.10 (d, $J$ = 8.0 Hz, 1H), 7.67 (dd, $J$ = 8.6, 4.8 Hz, 3H), 7.58 (d, $J$ = 7.6 Hz, 1H), 7.50 (d, $J$ = 6.1 Hz, 1H), 7.46 – 7.33 (m, 3H), 7.25 – 7.11 (m, 3H), 7.09 – 6.98 (m, 2H), 6.25 (t, $J$ = 4.8 Hz, 1H), 4.84 (d, $J$ = 6.2 Hz, 1H) ppm;

**$^{13}$C NMR** (101 MHz, $CDCl_3$) δ 164.6 ($C_q$), 161.9 ($C_q$), 161.3 (d, $J_{CF}$ = 4.7 Hz, $C_q$), 158.9 ($C_q$), 156.5 (CH), 150.4 (CH), 145.6 ($C_q$), 141.1 ($C_q$), 137.7 ($C_q$), 136.6 ($C_q$), 135.8 (CH), 133.7 (d, $J_{CF}$ = 10.1 Hz, CH), 133.4 ($C_q$), 131.1 (CH), 128.9 ($C_q$), 128.6 (CH), 128.0 (CH), 125.5 (CH), 124.5 (d, $J_{CF}$ = 4.0 Hz, $C_q$), 123.8 (CH), 123.1 (CH), 121.1 (CH), 117.3 (CH), 117.0 (CH), 115.6 (d, $J_{CF}$ = 4.3 Hz, CH), 111.9 (CH), 75.9 (CH), 44.7 (d, $J_{CF}$ = 2.5 Hz, CH) ppm;

**$^{19}$F NMR** (377 MHz, $CDCl_3$) δ –108.47 ppm;

**IR** (ATR): $\tilde{\nu}$ = 1665, 1580, 1555, 1483, 1435, 1384, 1118, 1063, 795, 749 $cm^{-1}$;

**HRMS (ESI):** *m/z* $[M+H]^+$ calcd for $C_{28}H_{19}FN_5O$: 460.1568; found: 460.1563;

**$[\alpha]^{D}_{20}$** = –270.2 (c = 0.50, $CHCl_3$);

**$R_t$** (ID-3 column, *n*-hexane/*i*-PrOH 50/50, 1.0 mL/min, 250.4 nm): tr(major) = 44.6 min, tr(minor) = 11.3 min, 99% ee.

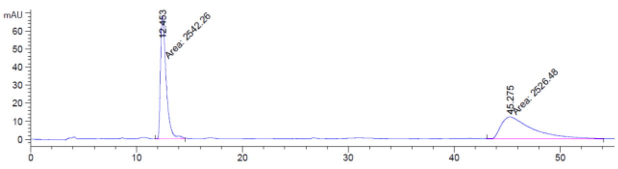


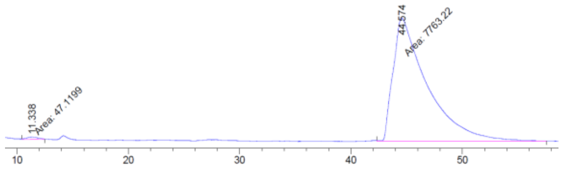


| Peak # | RetTime [min] | Type | Width [min] | Area [mAU*s] | Height [mAU] | Area % |
|---|---|---|---|---|---|---|
| 1 | 12.453 | MM | 0.6211 | 2542.25537 | 68.21837 | 50.1556 |
| 2 | 45.275 | MM | 3.4660 | 2526.47998 | 12.14893 | 49.8444 |

| Peak # | RetTime [min] | Type | Width [min] | Area [mAU*s] | Height [mAU] | Area % |
|---|---|---|---|---|---|---|
| 1 | 11.338 | MM | 1.1859 | 47.11985 | 6.62248e-1 | 0.6033 |
| 2 | 44.574 | MM | 3.5291 | 7763.21826 | 36.66262 | 99.3967 |

**(6a*R*,11b*R*)-4-Chloro-7-(pyrimidin-2-yl)-6-(quinolin-8-yl)-6,6a,7,11b-tetrahydro-5*H*-indolo[2,3-*c*]isoquinolin-5-one (7)**

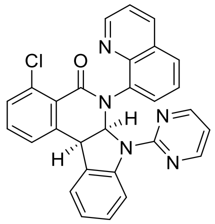

Prepared according to general procedure **A** on a 0.15 mmol scale, column chromatography (ethyl acetate/*n*-hexane = 2:1) afforded the title compound as a pale yellow sticky liquid (47 mg, 0.10 mmol, 66%), with >20:1 dr, 99% ee.

**$^1$H NMR** (400 MHz, $CDCl_3$) δ 8.97 (d, *J* = 4.3 Hz, 1H), 8.31 (d, *J* = 8.0 Hz, 1H), 8.08 (d, *J* = 8.0 Hz, 1H), 7.67 (d, *J* = 4.7 Hz, 2H), 7.60 – 7.51 (m, 3H), 7.47 (t, *J* = 5.3 Hz, 2H), 7.44 – 7.33 (m, 2H), 7.21 – 7.09 (m, 2H), 7.04 (t, *J* = 7.4 Hz, 1H), 6.99 (d, *J* = 7.5 Hz, 1H), 6.23 (t, *J* = 3.9 Hz, 1H), 4.82 (d, *J* = 6.2 Hz, 1H) ppm;

**$^{13}$C NMR** (101 MHz, $CDCl_3$) δ 162.1 ($C_q$), 158.8 ($C_q$), 156.5 (CH), 150.4 (CH), 145.5 ($C_q$), 141.1 ($C_q$), 137.9 ($C_q$), 136.9 ($C_q$), 136.6 ($C_q$), 135.8 (CH), 133.5 ($C_q$), 132.3 (CH), 132.3 (CH), 131.2 (CH), 128.9 ($C_q$), 128.7 (CH), 128.0 (CH), 127.8 (CH), 125.5 (CH), 124.1 ($C_q$), 123.9 (CH), 123.1 (CH), 121.2 (CH), 117.2 (CH), 111.9 (CH), 75.4 (CH), 45.1 (CH) ppm;

**IR** (ATR): $\tilde{\nu}$ = 1665, 1580, 1555, 1523, 1482, 1433, 1383, 1273, 795, 749 $cm^{-1}$;

**HRMS (ESI):** *m/z* $[M+H]^+$ calcd for $C_{28}H_{19}ClN_5O$: 476.1273; found: 476.1269;

**$[\alpha]^D_{20}$** = –220.8 (c = 0.25, $CHCl_3$);

**$R_t$** (ID-3 column, *n*-hexane/*i*-PrOH 50/50, 1.0 mL/min, 250.4 nm): tr(major) = 45.3 min, tr(minor) = 10.5 min, 99% ee.

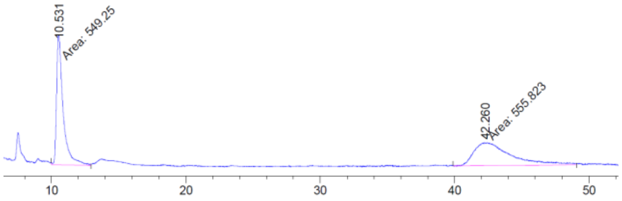


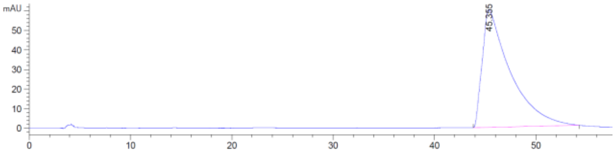


| Peak # | RetTime [min] | Type | Width [min] | Area [mAU*s] | Height [mAU] | Area % |
|---|---|---|---|---|---|---|
| 1 | 10.531 | MM | 0.5628 | 549.25006 | 16.26402 | 49.7026 |
| 2 | 42.260 | MM | 3.1971 | 555.82318 | 2.89752 | 50.2974 |

| Peak # | RetTime [min] | Type | Width [min] | Area [mAU*s] | Height [mAU] | Area % |
|---|---|---|---|---|---|---|
| 1 | 45.355 | BB | 2.2517 | 1.16100e4 | 60.34344 | 100.0000 |

**(6a*R*,11b*R*)-4-Bromo-7-(pyrimidin-2-yl)-6-(quinolin-8-yl)-6,6a,7,11b-tetrahydro-5*H*-indolo[2,3-*c*]isoquinolin-5-one (8)**

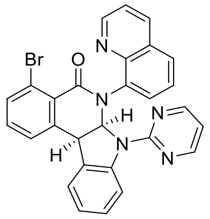

Prepared according to general procedure **A** on a 0.15 mmol scale, column chromatography (ethyl acetate/*n*-hexane = 2:1) afforded the title compound as a pale yellow sticky liquid (42 mg, 0.08 mmol, 54%), with >20:1 dr, 99% ee.

**$^{1}$H NMR** (400 MHz, $CDCl_3$) δ 8.99 (d, $J$ = 2.2 Hz, 1H), 8.30 (d, $J$ = 8.1 Hz, 1H), 8.09 (d, $J$ = 8.3 Hz, 1H), 7.82 (d, $J$ = 7.9 Hz, 1H), 7.68 (d, $J$ = 4.8 Hz, 2H), 7.57 (d, $J$ = 8.0 Hz, 1H), 7.55 – 7.45 (m, 3H), 7.44 – 7.33 (m, 2H), 7.21 – 7.11 (m, 2H), 7.05 (t, $J$ = 7.5 Hz, 1H), 6.99 (d, $J$ = 7.5 Hz, 1H), 6.24 (td, $J$ = 4.8, 1.4 Hz, 1H), 4.84 (d, $J$ = 6.2 Hz, 1H) ppm;

**$^{13}$C NMR** (101 MHz, $CDCl_3$) δ 162.1 ($C_q$), 158.8 ($C_q$), 156.5 (CH), 150.4 (CH), 145.5 ($C_q$), 141.0 ($C_q$), 137.9 ($C_q$), 136.6 ($C_q$), 136.0 (CH), 135.8 (CH), 133.6 ($C_q$), 132.4 (CH), 131.2 (CH), 129.0 ($C_q$), 128.7 (CH), 128.5 (CH), 128.0 (CH), 125.5 (CH), 125.2 ($C_q$), 124.7 ($C_q$), 123.9 (CH), 123.1 (CH), 121.2 (CH), 117.2 (CH), 111.9 (CH), 75.3 (CH), 45.2 (CH) ppm;

**IR** (ATR): $\tilde{\nu}$ = 1665, 1581, 1555, 1483, 1444, 1433, 1273, 1170, 796, 749 $cm^{-1}$;

**HRMS (ESI):** *m/z* $[M+H]^+$ calcd for $C_{28}H_{19}BrN_5O$: 520.0767; found: 520.0762;

**$[\alpha]^{D}_{20}$** = –182.8 (c = 0.50, $CHCl_3$);

**$R_t$** (ID-3 column, *n*-hexane/*i*-PrOH 50/50, 1.0 mL/min, 250.4 nm): tr(major) = 41.2 min, tr(minor) = 10.1 min, 99 ee.

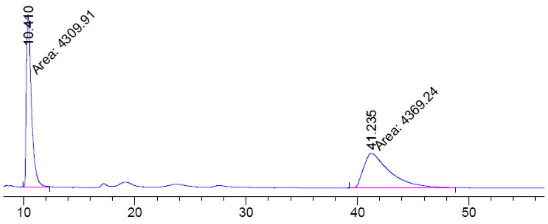


| Peak # | RetTime [min] | Type | Width [min] | Area [mAU*s] | Height [mAU] | Area % |
|---|---|---|---|---|---|---|
| 1 | 10.410 | MM | 0.5276 | 4309.91211 | 136.15276 | 49.6582 |
| 2 | 41.235 | MM | 2.6841 | 4369.24121 | 27.13068 | 50.3418 |

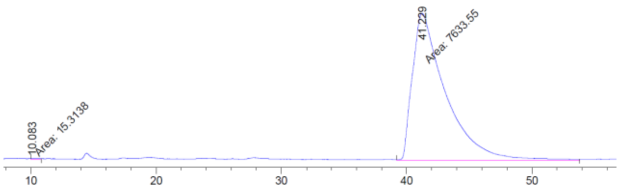


| Peak # | RetTime [min] | Type | Width [min] | Area [mAU*s] | Height [mAU] | Area % |
|---|---|---|---|---|---|---|
| 1 | 10.083 | MM | 0.7429 | 15.31380 | 3.43551e-1 | 0.2002 |
| 2 | 41.229 | MM | 3.0769 | 7633.54932 | 41.34925 | 99.7998 |

**(6a*R*,11b*R*)-3-Methyl-7-(pyrimidin-2-yl)-6-(quinolin-8-yl)-6,6a,7,11b-tetrahydro-5*H*-indolo[2,3-*c*]isoquinolin-5-one (9)**

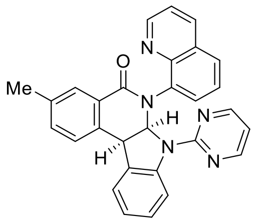


Prepared according to general procedure **A** on a 0.15 mmol scale, column chromatography (ethyl acetate/*n*-hexane = 2:1) afforded the title compound as a pale yellow sticky liquid (48 mg, 0.11 mmol, 70%), with >20:1 dr, 99% ee.

**$^{1}$H NMR** (400 MHz, $CDCl_3$) δ 8.97 (d, *J* = 4.0 Hz, 1H), 8.31 (d, *J* = 8.0 Hz, 1H), 8.15 – 8.04 (m, 2H), 7.66 (d, *J* = 4.8 Hz, 2H), 7.58 (t, *J* = 5.0 Hz, 1H), 7.54 – 7.43 (m, 3H), 7.44 – 7.32 (m, 2H), 7.17 (d, *J* = 4.7 Hz, 2H), 7.03 (d, *J* = 4.4 Hz, 2H), 6.23 (t, *J* = 4.7 Hz, 1H), 4.81 (d, *J* = 6.4 Hz, 1H), 2.46 (s, 3H) ppm;

**$^{13}$C NMR** (101 MHz, $CDCl_3$) δ 164.4 ($C_q$), 159.0 ($C_q$), 156.5 (CH), 150.4 (CH), 145.7 ($C_q$), 141.3 ($C_q$), 137.8 ($C_q$), 137.2 ($C_q$), 135.8 (CH), 134.2 ($C_q$), 133.3 (CH), 131.9 ($C_q$), 130.9 (CH), 130.0 (CH), 128.9 ($C_q$), 128.4 (CH), 128.3 (CH), 128.0 (CH), 127.0 ($C_q$), 125.6 (CH), 123.9 (CH), 123.0 (CH), 121.1 (CH), 117.2 (CH), 111.8 (CH), 76.4 (CH), 44.1 (CH), 21.3 ($CH_3$) ppm;

IR (ATR): $\tilde{\nu}$ = 1656, 1555, 1483, 1433, 1238, 1215, 1168, 1062, 796, 749 $cm^{-1}$;

**HRMS (ESI):** *m/z* $[M+H]^+$ calcd for $C_{29}H_{22}N_5O$: 456.1819; found: 456.1817;

**$[\alpha]^{D}_{20}$** = –262.8 (c = 0.50, $CHCl_3$);

**$R_t$** (OD-3 column, *n*-hexane/*i*-PrOH 80/20, 1.0 mL/min, 250.4 nm): tr(major) = 20.5 min, tr(minor) = 18.3 min, 99% ee.

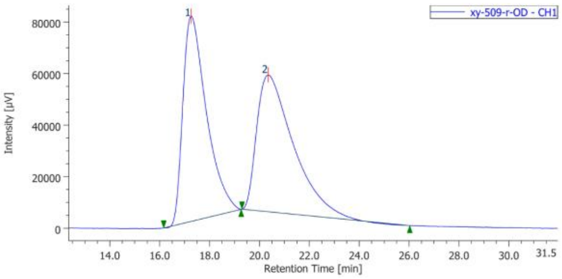


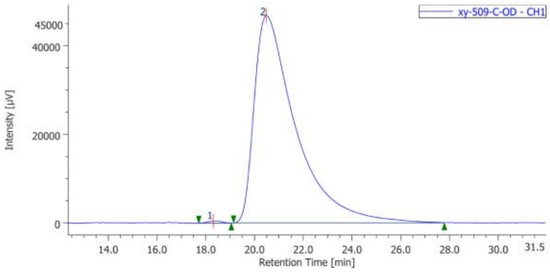


| # | Peak Name | CH | tR [min] | Area [µV·sec] | Height [µV] | Area% | Height% |
|---|---|---|---|---|---|---|---|
| 1 | Unknown | 1 | 17.260 | 5235588 | 79632 | 49.002 | 60.010 |
| 2 | Unknown | 1 | 20.350 | 5448940 | 53066 | 50.998 | 39.990 |
| Total | | | | 10684528 | 132698 | | |

| # | Peak Name | CH | tR [min] | Area [µV·sec] | Height [µV] | Area% | Height% |
|---|---|---|---|---|---|---|---|
| 1 | Unknown | 1 | 18.303 | 18769 | 479 | 0.338 | 1.012 |
| 2 | Unknown | 1 | 20.483 | 5531484 | 46806 | 99.662 | 98.988 |
| Total | | | | 5550253 | 47285 | | |

**(6a*R*,11bR)-3-Phenyl-7-(pyrimidin-2-yl)-6-(quinolin-8-yl)-6,6a,7,11b-tetrahydro-5*H*-indolo[2,3-*c*]isoquinolin-5-one (10)**

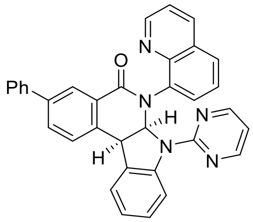

Prepared according to general procedure **A** on a 0.15 mmol scale, column chromatography (ethyl acetate/*n*-hexane = 2:1) afforded the title compound as a pale yellow sticky liquid (42 mg, 0.09 mmol, 62%), with >20:1 dr, 99% ee.

**$^{1}$H NMR** (400 MHz, $CDCl_3$) δ 9.00 (d, *J* = 4.3 Hz, 1H), 8.55 (s, 1H), 8.35 (d, *J* = 8.0 Hz, 1H), 8.12 (d, *J* = 8.3 Hz, 1H), 7.94 (d, *J* = 7.8 Hz, 1H), 7.77 – 7.64 (m, 5H), 7.60 (d, *J* = 7.1 Hz, 1H), 7.55 (d, *J* = 6.5 Hz, 1H), 7.52 – 7.34 (m, 5H), 7.24 – 7.14 (m, 2H), 7.14 – 7.03 (m, 2H), 6.25 (t, *J* = 4.7 Hz, 1H), 4.90 (d, *J* = 6.4 Hz, 1H) ppm;

**$^{13}$C NMR** (101 MHz, $CDCl_3$) δ 164.2 ($C_q$), 159.0 ($C_q$), 156.5 (CH), 150.5 (CH), 145.6 ($C_q$), 141.3 ($C_q$), 141.0 ($C_q$), 140.2 ($C_q$), 137.1 ($C_q$), 135.8 (CH), 133.9 ($C_q$), 133.8 ($C_q$), 131.0 (CH), 130.9 (CH), 129.1 (CH), 128.9 (CH), 128.9 ($C_q$), 128.4 (CH), 128.1 (CH), 127.7 (CH), 127.6 ($C_q$), 127.2 (CH), 125.6 (CH), 123.9 (CH), 123.1 (CH), 121.2 (CH), 117.3 (CH), 111.9 (CH), 76.4 (CH), 44.2 (CH) ppm;

**IR** (ATR): $\tilde{\nu}$ = 1659, 1581, 1554, 1482, 1429, 1387, 1272, 1232, 793, 751 $cm^{-1}$;

**HRMS (ESI):** *m/z* $[M+H]^+$ calcd for $C_{34}H_{24}N_5O$: 518.1975; found: 518.1972;

**$[\alpha]^{D}_{20}$** = –302.8 (c = 0.50, $CHCl_3$);

**$R_t$** (OD-3 column, *n*-hexane/*i*-PrOH 80/20, 1.0 mL/min, 250.4 nm): tr(major) = 21.8 min, tr(minor) = 33.1 min, 99% ee.

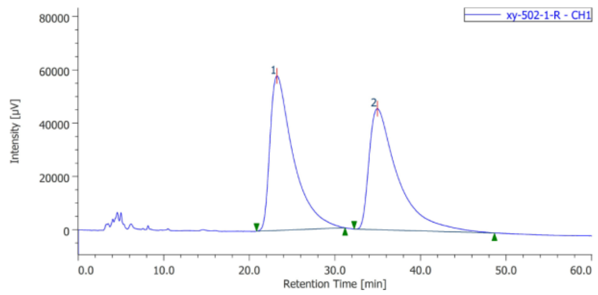


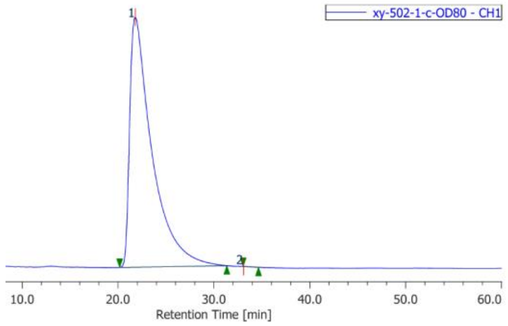


| # | Peak Name | CH | tR [min] | Area [µV·sec] | Height [µV] | Area% | Height% |
|---|---|---|---|---|---|---|---|
| 1 | Unknown | 1 | 23.207 | 10960369 | 58002 | 50.973 | 56.071 |
| 2 | Unknown | 1 | 34.947 | 10541898 | 45441 | 49.027 | 43.929 |
| Total | | | | 21502267 | 103443 | | |

| # | Peak Name | CH | tR [min] | Area [µV·sec] | Height [µV] | Area% | Height% |
|---|---|---|---|---|---|---|---|
| 1 | Unknown | 1 | 21.787 | 28476866 | 176633 | 99.983 | 99.976 |
| 2 | Unknown | 1 | 33.080 | 4846 | 42 | 0.017 | 0.024 |
| Total | | | | 28481712 | 176675 | | |

**(6a*R*,11b*S*)-1-Methoxy-7-(pyrimidin-2-yl)-6-(quinolin-8-yl)-6,6a,7,11b-tetrahydro-5*H*-indolo[2,3-*c*]isoquinolin-5-one (11)**

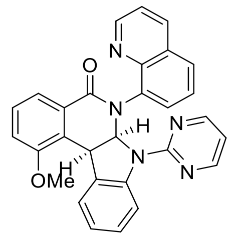

Prepared according to general procedure **A** on a 0.15 mmol scale, column chromatography (ethyl acetate/*n*-hexane = 2:1) afforded the title compound as a pale yellow sticky liquid (19 mg, 0.04mmol, 27%), with >20:1 dr, 99% ee.

**$^{1}$H NMR** (400 MHz, $CDCl_3$) δ 8.89 (d, $J$ = 3.4 Hz, 1H), 8.22 (d, $J$ = 8.0 Hz, 1H), 8.02 (d, $J$ = 8.2 Hz, 1H), 7.82 (d, $J$ = 7.7 Hz, 1H), 7.61 – 7.53 (m, 1H), 7.54 – 7.47 (m, 1H), 7.43 – 7.30 (m, 3H), 7.29 – 7.24 (m, 1H), 7.22 – 7.14 (m, 2H), 7.12 – 7.07 (m, 2H), 6.99 – 6.90 (m, 2H), 6.14 (t, $J$ = 4.9 Hz, 1H), 5.15 (d, $J$ = 6.6 Hz, 1H), 3.93 (s, 3H) ppm;

**$^{13}$C NMR** (101 MHz, $CDCl_3$) δ 164.3 ($C_q$), 159.0 ($C_q$), 156.7 ($C_q$), 156.4 (CH), 150.4 (CH), 145.7 ($C_q$), 141.6 ($C_q$), 137.2 ($C_q$), 135.7 (CH), 133.7 ($C_q$), 130.8 (CH), 128.9 ($C_q$), 128.5 ($C_q$), 128.3 (CH), 128.0 (CH), 128.0 (CH), 125.6 (CH), 124.5 (CH), 124.3 ($C_q$), 123.0 (CH), 121.2 (CH), 121.1 (CH), 117.2 (CH), 114.1 (CH), 111.7 (CH), 76.3 (CH), 56.0 ($CH_3$), 38.9 (CH) ppm;

**IR** (ATR): $\tilde{\nu}$ = 1660, 1580, 1555, 1482, 1435, 1401, 1295, 1270, 796, 748 $cm^{-1}$;

**HRMS (ESI):** *m/z* $[M+H]^+$ calcd for $C_{29}H_{22}N_5O_2$: 472.1768; found: 472.1761;

**$[\alpha]^{D}_{20}$** = –71.3 (c = 1.00, $CHCl_3$);

**$R_t$** (AD-3 column, *n*-hexane/*i*-PrOH 60/40, 1.0 mL/min, 250.4 nm): tr(major) = 10.9 min, tr(minor) = 5.5 min, 99% ee.

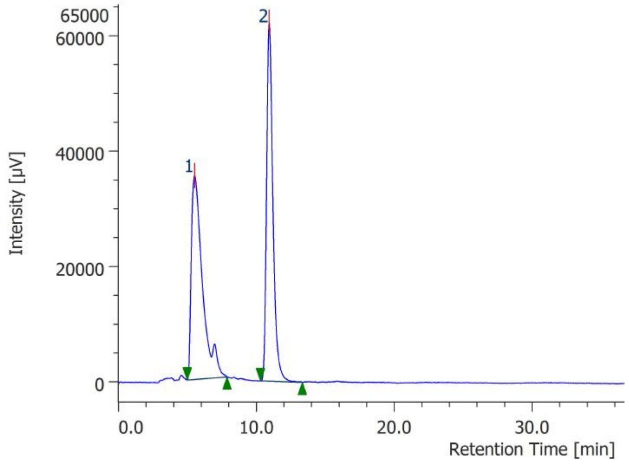


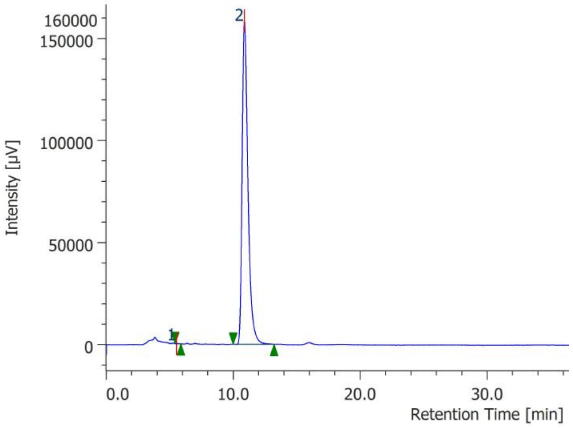


| # | Peak Name | CH | tR [min] | Area [µV·sec] | Height [µV] | Area% | Height% |
|---|---|---|---|---|---|---|---|
| 1 | Unknown | 1 | 5.510 | 1989412 | 35329 | 50.117 | 36.246 |
| 2 | Unknown | 1 | 10.923 | 1980099 | 62140 | 49.883 | 63.754 |
| Total | | | | 3969511 | 97469 | | |

| # | Peak Name | CH | tR [min] | Area [µV·sec] | Height [µV] | Area% | Height% |
|---|---|---|---|---|---|---|---|
| 1 | Unknown | 1 | 5.523 | 1881 | 116 | 0.037 | 0.073 |
| 2 | Unknown | 1 | 10.883 | 5070848 | 158373 | 99.963 | 99.927 |
| Total | | | | 5072729 | 158489 | | |

**(6a*R*,11b*R*)-3-Methoxy-7-(pyrimidin-2-yl)-6-(quinolin-8-yl)-6,6a,7,11b-tetrahydro-5*H*-indolo[2,3-*c*]isoquinolin-5-one (11')**

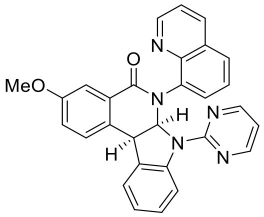


Prepared according to general procedure **A** on a 0.15 mmol scale, column chromatography (ethyl acetate/*n*-hexane = 2:1) afforded the title compound as a pale yellow sticky liquid (27 mg, 0.06 mmol, 38%), with >20:1 dr, 99% ee.

**$^1$H NMR** (400 MHz, $CDCl_3$) δ 8.90 (d, *J* = 3.9 Hz, 1H), 8.23 (d, *J* = 8.0 Hz, 1H), 8.03 (d, *J* = 8.2 Hz, 1H), 7.74 (s, 1H), 7.58 (d, *J* = 4.7 Hz, 2H), 7.51 (t, *J* = 5.0 Hz, 1H), 7.45 – 7.37 (m, 2H), 7.37 – 7.24 (m, 2H), 7.18 (s, 1H), 7.09 (d, *J* = 4.9 Hz, 2H), 6.96 (d, *J* = 4.6 Hz, 2H), 6.15 (t, *J* = 4.6 Hz, 1H), 4.72 (d, *J* = 6.4 Hz, 1H), 3.81 (s, 3H) ppm;

**$^{13}$C NMR** (101 MHz, $CDCl_3$) δ 164.2 ($C_q$), 159.5 ($C_q$), 159.0 ($C_q$), 156.5 (CH), 150.5 (CH), 145.6 ($C_q$), 141.2 ($C_q$), 137.1 ($C_q$), 135.8 (CH), 134.3 ($C_q$), 130.8 (CH), 129.7 (CH), 128.9 ($C_q$), 128.3 ($C_q$), 128.3 (CH), 128.1 (CH), 127.1 ($C_q$), 125.6 (CH), 123.9 (CH), 123.1 (CH), 121.1 (CH), 120.2 (CH), 117.2 (CH), 112.7 (CH), 111.8 (CH), 76.5 (CH), 55.6 ($CH_3$), 43.7 (CH) ppm;

**IR** (ATR): $\tilde{\nu}$ = 1658, 1579, 1554, 1482, 1432, 1386, 1280, 1032, 795, 753 $cm^{-1}$;

**HRMS (ESI):** *m/z* $[M+H]^+$ calcd for $C_{29}H_{22}N_5O_2$: 472.1768; found: 472.1761;

**$[\alpha]^D_{20}$** = –325.2 (c = 0.50, $CHCl_3$);

**$R_t$** (AD-3 column, *n*-hexane/*i*-PrOH 60/40, 1.0 mL/min, 250.4 nm): tr(major) = 47.1 min, tr(minor) = 6.9 min, 99% ee.

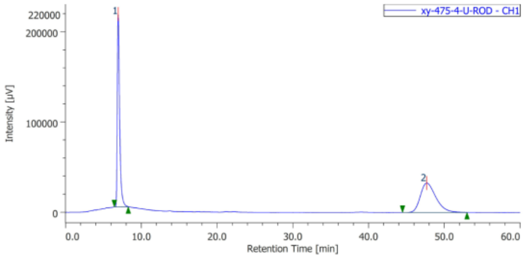


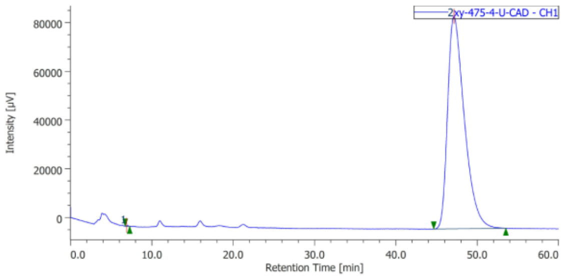


| # | Peak Name | CH | tR [min] | Area [µV·sec] | Height [µV] | Area% | Height% |
|---|---|---|---|---|---|---|---|
| 1 | Unknown | 1 | 6.943 | 4663881 | 213531 | 50.336 | 86.766 |
| 2 | Unknown | 1 | 47.650 | 4601661 | 32569 | 49.664 | 13.234 |
| Total | | | | 9265542 | 246100 | | |

| # | Peak Name | CH | tR [min] | Area [µV·sec] | Height [µV] | Area% | Height% |
|---|---|---|---|---|---|---|---|
| 1 | Unknown | 1 | 6.917 | 4956 | 348 | 0.040 | 0.398 |
| 2 | Unknown | 1 | 47.120 | 12445041 | 87170 | 99.960 | 99.602 |
| Total | | | | 12449997 | 87518 | | |

**(6a*R*,11b*R*)-2-Methyl-7-(pyrimidin-2-yl)-6-(quinolin-8-yl)-6,6a,7,11b-tetrahydro-5*H*-indolo[2,3-*c*]isoquinolin-5-one (12)**

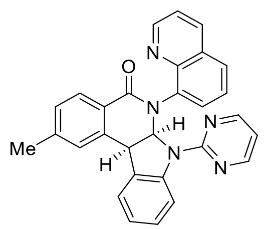


Prepared according to general procedure **A** on a 0.15 mmol scale, column chromatography (ethyl acetate/*n*-hexane = 2:1) afforded the title compound as a pale yellow sticky liquid (49 mg, 0.11 mmol, 72%), with >20:1 dr, 99% ee.

**$^1$H NMR** (400 MHz, $CDCl_3$) δ 8.88 (d, *J* = 3.4 Hz, 1H), 8.23 (d, *J* = 8.0 Hz, 1H), 8.08 (d, *J* = 7.9 Hz, 1H), 8.01 (d, *J* = 8.2 Hz, 1H), 7.57 (d, *J* = 4.8 Hz, 2H), 7.49 (dd, *J* = 6.2, 3.6 Hz, 1H), 7.41 (d, *J* = 6.3 Hz, 1H), 7.36 – 7.23 (m, 2H), 7.23 (d, *J* = 8.0 Hz, 1H), 7.18 (s, 1H), 7.08 (d, *J* = 4.1 Hz, 2H), 6.96 (d, *J* = 4.5 Hz, 2H), 6.14 (t, *J* = 4.7 Hz, 1H), 4.70 (d, *J* = 6.4 Hz, 1H), 2.48 (s, 3H) ppm;

**$^{13}$C NMR** (101 MHz, $CDCl_3$) δ 164.4 ($C_q$), 159.0 ($C_q$), 156.5 (CH), 150.4 (CH), 145.7 ($C_q$), 143.1 ($C_q$), 141.3 ($C_q$), 137.2 ($C_q$), 135.8 (CH), 134.9 ($C_q$), 134.0 ($C_q$), 130.9 (CH), 129.6 (CH), 129.0 (CH), 128.9 (CH), 128.3 (CH), 128.0 (CH), 125.6 (CH), 124.7 ($C_q$), 123.9 (CH), 123.0 (CH), 121.1 (CH), 117.2 (CH), 111.8 (CH), 76.4 (CH), 44.4 (CH), 21.8 ($CH_3$) ppm;

**IR** (ATR): $\tilde{\nu}$ = 1658, 1614, 1580, 1554, 1482, 1433, 1386, 1292, 794, 751 $cm^{-1}$;

**HRMS (ESI):** *m/z* $[M+H]^+$ calcd for $C_{29}H_{22}N_5O$: 456.1819; found: 456.1819;

**$[\alpha]^D_{20}$** = –204.9 (c = 1.00, $CHCl_3$);

**$R_t$** (OD-3 column, *n*-hexane/*i*-PrOH 70/30, 1.0 mL/min, 250.4 nm): tr(major) = 20.0 min, tr(minor) = 8.7 min, 99% ee.

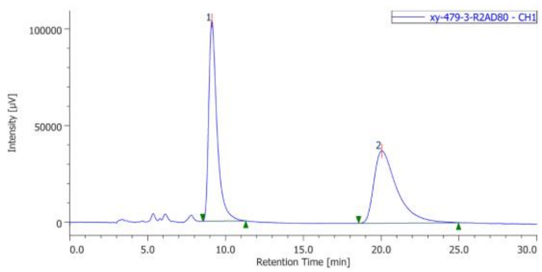


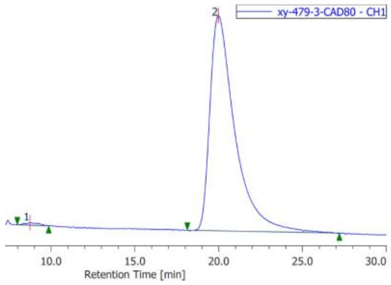


| # | Peak Name | CH | tR [min] | Area [μV·sec] | Height [μV] | Area% | Height% |
|---|---|---|---|---|---|---|---|
| 1 | Unknown | 1 | 9.120 | 3837672 | 103611 | 50.067 | 73.441 |
| 2 | Unknown | 1 | 20.033 | 3827362 | 37469 | 49.933 | 26.559 |
| Total | | | | 7665034 | 141080 | | |

| # | Peak Name | CH | tR [min] | Area [μV·sec] | Height [μV] | Area% | Height% |
|---|---|---|---|---|---|---|---|
| 1 | Unknown | 1 | 8.737 | 25535 | 459 | 0.627 | 1.161 |
| 2 | Unknown | 1 | 19.967 | 4046631 | 39059 | 99.373 | 98.839 |
| Total | | | | 4072166 | 39518 | | |

**(6a*R*,11b*R*)-5-Oxo-7-(pyrimidin-2-yl)-6-(quinolin-8-yl)-6,6a,7,11b-tetrahydro-5*H*-indolo[2,3-*c*]isoquinolin-2-yl acetate (13)**

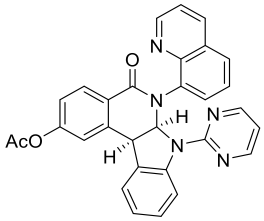


Prepared according to general procedure **A** on a 0.15 mmol scale, column chromatography (ethyl acetate/*n*-hexane = 2:1) afforded the title compound as a pale yellow sticky liquid (53 mg, 0.11 mmol, 71%), with >20:1 dr, 99% ee.

**$^{1}$H NMR** (400 MHz, $CDCl_3$) δ 8.97 (s, 1H), 8.34 (dd, *J* = 12.5, 8.0 Hz, 2H), 8.27 (s, 1H), 8.20 – 8.07 (m, 2H), 7.73 – 7.56 (m, 3H), 7.54 (d, *J* = 6.5 Hz, 1H), 7.46 – 7.30 (m, 2H), 7.18 (d, *J* = 4.9 Hz, 2H), 7.04 (d, *J* = 8.7 Hz, 2H), 6.25 (s, 1H), 4.91 (d, *J* = 6.4 Hz, 1H), 4.04 (s, 3H) ppm;

**$^{13}$C NMR** (101 MHz, $CDCl_3$) δ 166.5 ($C_q$), 163.3 ($C_q$), 158.9 ($C_q$), 156.5 (CH), 150.5 (CH), 145.4 ($C_q$), 141.2 ($C_q$), 136.7 ($C_q$), 135.8 (CH), 135.2 ($C_q$), 133.6 ($C_q$), 133.3 ($C_q$), 130.9 ($C_q$), 130.8 (CH), 129.8 (CH), 129.7 (CH), 129.0 (CH), 128.9 ($C_q$), 128.6 (CH), 128.2 (CH), 125.6 (CH), 123.9 (CH), 123.2 (CH), 121.2 (CH), 117.4 (CH), 112.0 (CH), 76.3 (CH), 52.6 ($CH_3$), 44.2 (CH) ppm;

**IR** (ATR): $\tilde{\nu}$ = 1721, 1664, 1581, 1555, 1483, 1432, 1277, 1200, 795, 749 $cm^{-1}$;

**HRMS (ESI):** *m/z* $[M+H]^+$ calcd for $C_{30}H_{22}N_5O_3$: 500.1717; found: 500.1711;

**$[\alpha]^{D}_{20}$** = –239.6 (c = 0.50, $CHCl_3$);

**$R_t$** (ID-3 column, *n*-hexane/*i*-PrOH 50/50, 1.0 mL/min, 250.4 nm): tr(major) = 37.0 min, tr(minor) = 15.8 min, 99% ee.

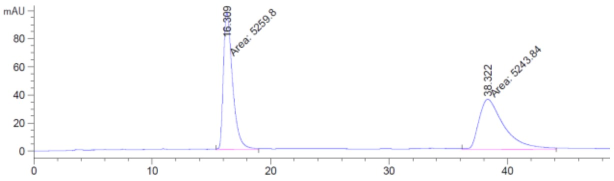


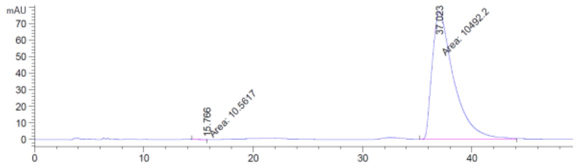


| Peak # | RetTime [min] | Type | Width [min] | Area [mAU*s] | Height [mAU] | Area % |
|---|---|---|---|---|---|---|
| 1 | 16.309 | MM | 0.8980 | 5259.80420 | 97.62533 | 50.0760 |
| 2 | 38.322 | MM | 2.4550 | 5243.83984 | 35.60002 | 49.9240 |

| Peak # | RetTime [min] | Type | Width [min] | Area [mAU*s] | Height [mAU] | Area % |
|---|---|---|---|---|---|---|
| 1 | 15.766 | MM | 0.5208 | 10.56165 | 3.37977e-1 | 0.1006 |
| 2 | 37.023 | MM | 2.2710 | 1.04922e4 | 77.00117 | 99.8994 |

**(6a*R*,11b*R*)-2-Chloro-7-(pyrimidin-2-yl)-6-(quinolin-8-yl)-6,6a,7,11b-tetrahydro-5*H*-indolo[2,3-*c*]isoquinolin-5-one (14)**

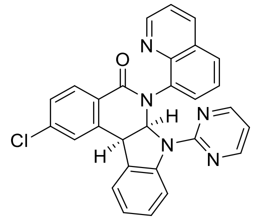


Prepared according to general procedure **A** on a 0.15 mmol scale, column chromatography (ethyl acetate/*n*-hexane = 2:1) afforded the title compound as a pale yellow sticky liquid (40 mg, 0.08 mmol, 56%), with >20:1 dr, 99% ee.

**$^1$H NMR** (400 MHz, $CDCl_3$) δ 9.00 – 8.94 (m, 1H), 8.33 (d, *J* = 8.0 Hz, 1H), 8.22 (d, *J* = 8.3 Hz, 1H), 8.12 (d, *J* = 8.4 Hz, 1H), 7.68 (d, *J* = 4.8 Hz, 2H), 7.63 – 7.56 (m, 2H), 7.55 – 7.35 (m, 4H), 7.22 – 7.13 (m, 2H), 7.11 – 7.00 (m, 2H), 6.26 (t, *J* = 4.7 Hz, 1H), 4.81 (d, *J* = 6.4 Hz, 1H) ppm;

**$^{13}$C NMR** (101 MHz, $CDCl_3$) δ 163.4 ($C_q$), 158.9 ($C_q$), 156.5 (CH), 150.5 (CH), 145.5 ($C_q$), 141.2 ($C_q$), 138.6 ($C_q$), 136.8 ($C_q$), 136.7 ($C_q$), 135.8 (CH), 133.1 ($C_q$), 131.2 (CH), 130.8 (CH), 128.9 ($C_q$), 128.6 (CH), 128.4 (CH), 128.3 (CH), 128.2 (CH), 125.8 ($C_q$), 125.6 (CH), 123.8 (CH), 123.2 (CH), 121.2 (CH), 117.4 (CH), 112.0 (CH), 76.3 (CH), 44.2 (CH) ppm;

**IR** (ATR): $\tilde{\nu}$ = 1665, 1614, 1580, 1555, 1482, 1433, 1386, 1292, 794, 749 $cm^{-1}$;

**HRMS (ESI):** *m/z* $[M+H]^+$ calcd for $C_{28}H_{19}ClN_5O$: 476.1273; found: 476.1271;

**$[\alpha]^{D}_{20}$** = –221.6 (c = 0.50, $CHCl_3$);

**$R_t$** (ID-3 column, *n*-hexane/*i*-PrOH 50/50, 1.0 mL/min, 250.4 nm): tr(major) = 34.5 min, tr(minor) = 11.7 min, 99% ee.

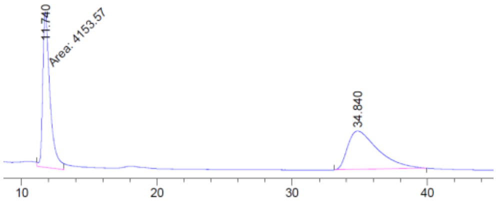


| Peak # | RetTime [min] | Type | Width [min] | Area [mAU*s] | Height [mAU] | Area % |
|---|---|---|---|---|---|---|
| 1 | 11.740 | MM | 0.6225 | 4153.56738 | 111.21391 | 49.3332 |
| 2 | 34.840 | BB | 1.8201 | 4265.85596 | 27.43449 | 50.6668 |

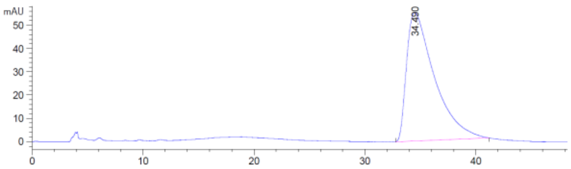


| Peak # | RetTime [min] | Type | Width [min] | Area [mAU*s] | Height [mAU] | Area % |
|---|---|---|---|---|---|---|
| 1 | 34.490 | BB | 2.0438 | 9654.00977 | 55.29741 | 100.0000 |

**(6a*R*,11b*R*)-1,3-Dimethyl-7-(pyrimidin-2-yl)-6-(quinolin-8-yl)-6,6a,7,11b-tetrahydro-5*H*-indolo[2,3-*c*]isoquinolin-5-one (15)**

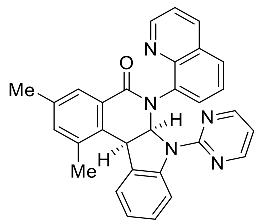


Prepared according to general procedure **A** on a 0.15 mmol scale, column chromatography (ethyl acetate/*n*-hexane = 2:1) afforded the title compound as a pale yellow sticky liquid (51 mg, 0.11 mmol, 73%), with >20:1 dr, 99% ee.

$^{1}$**H NMR** (400 MHz, $CDCl_3$) δ 8.95 (d, *J* = 4.3 Hz, 1H), 8.32 (t, *J* = 5.8 Hz, 1H), 8.08 (d, *J* = 8.1 Hz, 1H), 7.97 (s, 1H), 7.83 – 7.50 (m, 3H), 7.48 – 7.31 (m, 4H), 7.21 – 7.11 (m, 2H), 7.01 (t, *J* = 7.2 Hz, 1H), 6.92 (d, *J* = 7.5 Hz, 1H), 6.28 – 6.15 (m, 1H), 4.94 (d, *J* = 6.4 Hz, 1H), 2.54 (s, 3H), 2.42 (s, 3H) ppm;

$^{13}$**C NMR** (101 MHz, $CDCl_3$) δ 164.8 ($C_q$), 159.0 ($C_q$), 156.5 (CH), 150.4 (CH), 145.6, 141.6 ($C_q$), 137.3 ($C_q$), 137.2 ($C_q$), 135.8 (CH), 135.5 ($C_q$), 135.3 ($C_q$), 135.3 (CH), 133.4 ($C_q$), 130.8 (CH), 130.7 ($C_q$), 128.9 ($C_q$), 128.2 (CH), 128.0 (CH), 127.9 (CH), 127.4 ($C_q$), 125.6 (CH), 124.0 (CH), 123.0 (CH), 121.1 (CH), 117.3 (CH), 111.8 (CH), 76.7 (CH), 41.6 (CH), 21.2 ($CH_3$), 18.8 ($CH_3$) ppm;

**IR** (ATR): $\tilde{\nu}$ = 1660, 1578, 1554, 1482, 1439, 1388, 1304, 1270, 794, 751 $cm^{-1}$;

**HRMS (ESI):** *m/z* $[M+H]^+$ calcd for $C_{30}H_{24}N_5O$: 470.1975; found: 470.1979;

**[α]$^{D}$$_{20}$** = –319.9 (c = 1.00, $CHCl_3$);

**R$_t$** (IA-3 column, *n*-hexane/*i*-PrOH 50/50, 1.0 mL/min, 250.4 nm): tr(major) = 16.5 min, tr(minor) = 5.4 min, 99% ee.

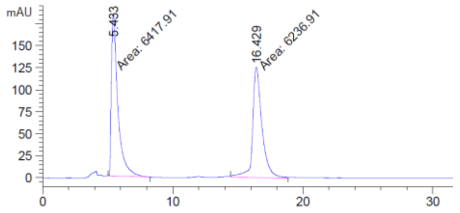


| Peak # | RetTime [min] | Type | Width [min] | Area [mAU*s] | Height [mAU] | Area % |
|---|---|---|---|---|---|---|
| 1 | 5.433 | MM | 0.5812 | 6417.90674 | 184.04677 | 50.7151 |
| 2 | 16.429 | MM | 0.8294 | 6236.91016 | 125.33669 | 49.2849 |

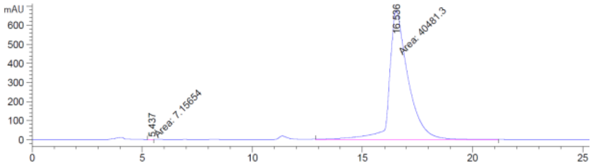


| Peak # | RetTime [min] | Type | Width [min] | Area [mAU*s] | Height [mAU] | Area % |
|---|---|---|---|---|---|---|
| 1 | 5.437 | MM | 0.2880 | 7.15654 | 2.96377e-1 | 0.0177 |
| 2 | 16.536 | MM | 0.9951 | 4.04813e4 | 678.01935 | 99.9823 |

**(6a*R*,11b*S*)-1,2,3-Trimethoxy-7-(pyrimidin-2-yl)-6-(quinolin-8-yl)-6,6a,7,11b-tetrahydro-5*H*-indolo[2,3-*c*]isoquinolin-5-one (16)**

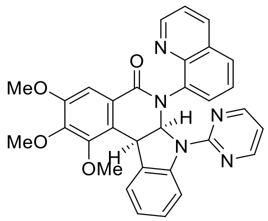


Prepared according to general procedure **A** on a 0.15 mmol scale, column chromatography (ethyl acetate/*n*-hexane = 2:1) afforded the title compound as a pale yellow sticky liquid (49 mg, 0.09 mmol, 61%), with >20:1 dr, 99% ee.

**$^{1}$H NMR** (400 MHz, $CDCl_3$) δ 9.00 (dd, *J* = 4.1, 2.1 Hz, 1H), 8.30 (d, *J* = 8.0 Hz, 1H), 8.11 (d, *J* = 8.2 Hz, 1H), 7.64 – 7.58(m, 4H), 7.43 (m, 2H), 7.35 (t, *J* = 7.7 Hz, 1H), 7.22 – 7.12 (m, 2H), 7.12 – 7.00 (m, 2H), 6.22 (t, *J* = 4.8 Hz, 1H), 5.07 (d, *J* = 6.5 Hz, 1H), 4.08 (s, 6H), 3.93 (s, 3H) ppm;

**$^{13}$C NMR** (101 MHz, $CDCl_3$) δ 164.0 ($C_q$), 159.0 ($C_q$), 156.4 (CH), 153.1 ($C_q$), 150.7 ($C_q$), 150.4 (CH), 145.7 ($C_q$), 141.4 ($C_q$), 137.1 ($C_q$), 135.8 (CH), 133.9 ($C_q$), 130.8 (CH), 128.8 ($C_q$), 128.0 (CH), 128.0 (CH), 125.6 (CH), 124.5 (CH), 123.1 (CH), 122.8 ($C_q$), 121.7 ($C_q$), 121.1 (CH), 117.1 (CH), 111.7 (CH), 107.8 (CH), 76.6 (CH), 61.4 ($CH_3$), 61.0 ($CH_3$), 56.2 ($CH_3$), 39.3 (CH) ppm;

**IR** (ATR): $\tilde{\nu}$ = 1660, 1579, 1554, 1484, 1442, 1384, 1342, 1116, 795, 750 $cm^{-1}$;

**HRMS (ESI):** *m/z* $[M+H]^+$ calcd for $C_{31}H_{26}N_5O_4$: 532.1979; found: 532.1972;

**$[\alpha]^{D}_{20}$** = –263.4 (c = 0.50, $CHCl_3$);

**$R_t$** (AD-3 column, *n*-hexane/*i*-PrOH 80/20, 1.0 mL/min, 250.4 nm): tr(major) = 33.7 min, tr(minor) = 9.2 min, 99% ee.

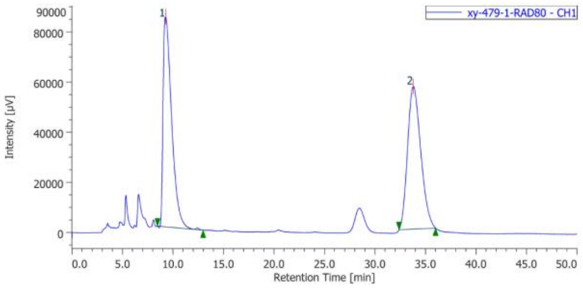


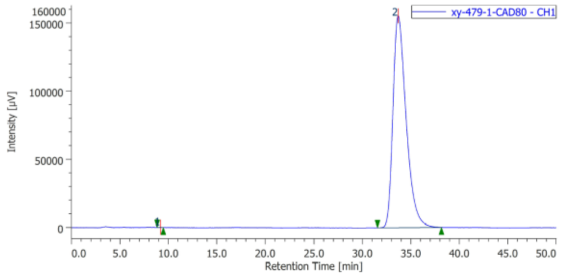


| # | Peak Name | CH | tR [min] | Area [µV·sec] | Height [µV] | Area% | Height% |
|---|---|---|---|---|---|---|---|
| 1 | Unknown | 1 | 9.260 | 4950371 | 83789 | 48.957 | 59.575 |
| 2 | Unknown | 1 | 33.790 | 5161268 | 56854 | 51.043 | 40.425 |
| Total | | | | 10111639 | 140643 | | |

| # | Peak Name | CH | tR [min] | Area [µV·sec] | Height [µV] | Area% | Height% |
|---|---|---|---|---|---|---|---|
| 1 | Unknown | 1 | 9.207 | 1207 | 103 | 0.008 | 0.066 |
| 2 | Unknown | 1 | 33.680 | 14704850 | 155280 | 99.992 | 99.934 |
| Total | | | | 14706057 | 155383 | | |

**(5a*R*,13b*R*)-5-(Pyrimidin-2-yl)-6-(quinolin-8-yl)-5,5a,6,13b-tetrahydro-7*H*-benzo[*g*]indolo[2,3-*c*]isoquinolin-7-one (17)**

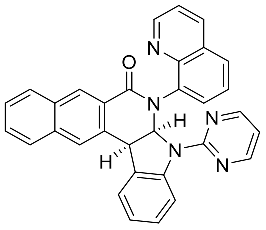

Prepared according to general procedure **A** on a 0.15 mmol scale, column chromatography (ethyl acetate/*n*-hexane = 2:1) afforded the title compound as a pale yellow sticky liquid (39 mg, 0.08 mmol, 53%), with 14:1 dr, 99% ee.

**$^{1}$H NMR** (400 MHz, $CDCl_3$) δ 8.95 (d, *J* = 3.8 Hz, 1H), 8.85 (s, 1H), 8.34 (d, *J* = 8.1 Hz, 1H), 8.12 (d, *J* = 8.4 Hz, 1H), 8.06 – 7.98 (m, 3H), 7.75 – 7.50 (m, 6H), 7.45 – 7.33 (m, 2H), 7.20 (d, *J* = 9.6 Hz, 2H), 7.02 (d, *J* = 6.0 Hz, 2H), 6.26 (t, *J* = 4.3 Hz, 1H), 5.03 (d, *J* = 6.2 Hz, 1H).

**$^{13}$C NMR** (101 MHz, $CDCl_3$) δ 164.3 ($C_q$), 159.0 ($C_q$), 156.5 (CH), 150.5 (CH), 145.6 ($C_q$), 141.1 ($C_q$), 137.1 ($C_q$), 135.8 (CH), 135.4 ($C_q$), 134.2 ($C_q$), 132.7 ($C_q$), 131.1 ($C_q$), 130.9 (CH), 129.7 (CH), 128.9 ($C_q$), 128.5 (CH), 128.3 (CH), 128.1 (CH), 127.4 (CH), 127.3 (CH), 126.5 (CH), 125.6 (CH), 125.0 ($C_q$), 124.1 (CH), 123.1 (CH), 121.1 (CH), 117.3 (CH), 111.8 (CH), 76.4 (CH), 44.9 (CH) ppm;

**IR** (ATR): $\tilde{\nu}$ = 1658, 1629, 1580, 1554, 1483, 1435, 1289, 1210, 794, 749 $cm^{-1}$;

**HRMS (ESI):** *m/z* $[M+H]^+$ calcd for $C_{32}H_{22}N_5O$: 492.1819; found: 492.1811;

**$[\alpha]^D_{20}$** = –336.8 (c = 1.00, $CHCl_3$);

**$R_t$** (IB-3 column, *n*-hexane/*i*-PrOH 70/30, 1.0 mL/min, 250.4 nm): tr(major) = 20.6 min, tr(minor) = 23.5 min, 99% ee.

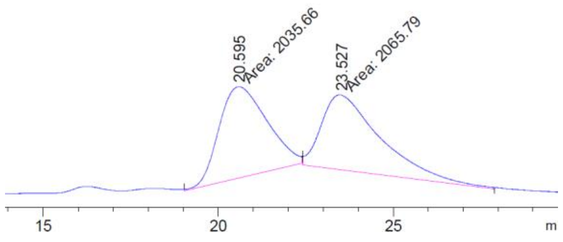


| Peak # | RetTime [min] | Type | Width [min] | Area [mAU*s] | Height [mAU] | Area % |
|---|---|---|---|---|---|---|
| 1 | 20.595 | MM | 1.5814 | 2035.66162 | 21.45479 | 49.6327 |
| 2 | 23.527 | MM | 1.9666 | 2065.79297 | 17.50739 | 50.3673 |

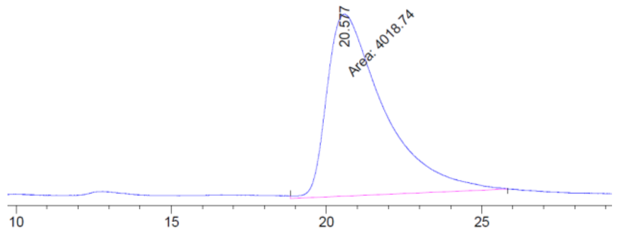


| Peak # | RetTime [min] | Type | Width [min] | Area [mAU*s] | Height [mAU] | Area % |
|---|---|---|---|---|---|---|
| 1 | 20.577 | MM | 2.1231 | 4018.74023 | 31.54847 | 100.0000 |

**(6a*R*,11b*R*)-7-(Pyrimidin-2-yl)-6-(quinolin-8-yl)-6,6a,7,11b-tetrahydro-5*H*-benzo[*h*]indolo[2,3-*c*]isoquinolin-5-one (18)**

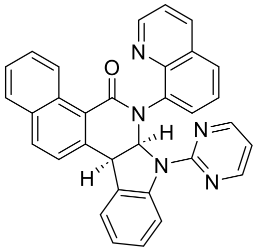


Prepared according to general procedure **A** on a 0.15 mmol scale, column chromatography (*n*-hexane/ethyl acetate = 1:2) afforded the title compound a pale yellow sticky liquid (41 mg, 0.08 mmol, 55%), with >20:1 dr, 99% ee.

**$^{1}$H NMR** (400 MHz, $CDCl_3$) δ 8.98 (d, *J* = 4.1 Hz, 1H), 8.91 (s, 1H), 8.41 (d, *J* = 8.0 Hz, 1H), 8.15 (d, *J* = 8.2 Hz, 1H), 8.10 – 8.00 (m, 3H), 7.76 – 7.56 (m, 6H), 7.43 (m, 2H), 7.33 – 7.21 (m, 2H), 7.07 (m, 2H), 6.28 (t, *J* = 4.7 Hz, 1H), 5.05 (d, *J* = 6.1 Hz, 1H) ppm;

**$^{13}$C NMR** (101 MHz, $CDCl_3$) δ 164.2 ($C_q$), 158.8 ($C_q$), 156.4 (CH), 150.3 (CH), 145.4 ($C_q$), 141.0 ($C_q$), 137.0 ($C_q$), 135.7 (CH), 135.2 ($C_q$), 134.1 ($C_q$), 132.6 ($C_q$), 130.9 ($C_q$), 130.8 (CH), 130.8 (CH), 129.5 (CH), 128.7 ($C_q$), 128.3 (CH), 128.1 (CH), 128.0 (CH), 127.3 (CH), 127.2 (CH), 126.3 (CH), 125.4 (CH), 124.8 ($C_q$), 123.9 (CH), 122.9 (CH), 121.0 (CH), 117.1 (CH), 111.7 (CH), 76.3 (CH), 44.7 (CH) ppm.

**IR** (ATR): $\tilde{\nu}$ = 3047, 1657, 1580, 1482, 1433, 1383, 1288, 794, 750, 480 $cm^{-1}$;

**HRMS (ESI):** *m/z* $[M+H]^+$ calcd for $C_{32}H_{22}N_5O$: 492.1819; found: 492.1816;

**$[\alpha]^{D}_{20}$** = –384.2 (c = 0.50, $CHCl_3$);

**$R_t$** (OD-3 column, *n*-hexane/*i*-PrOH 80/20, 1.0 mL/min, 250.4 nm): tr(major) = 39.8 min, tr(minor) = 32.5 min, 99% ee.

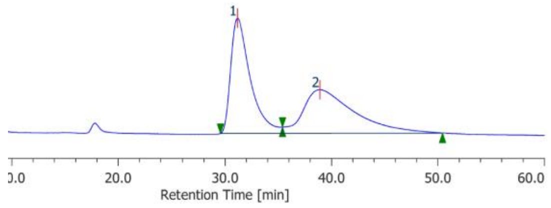


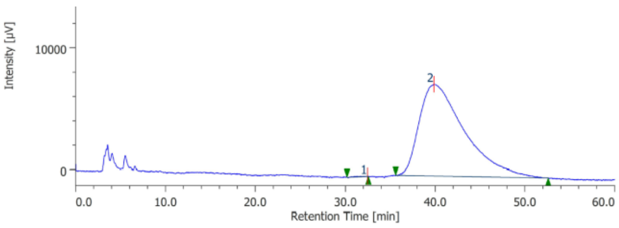


| # | Peak Name | CH | tR [min] | Area [µV·sec] | Height [µV] | Area% | Height% |
|---|---|---|---|---|---|---|---|
| 1 | Unknown | 1 | 31.150 | 7994183 | 62485 | 50.397 | 72.529 |
| 2 | Unknown | 1 | 38.900 | 7868275 | 23667 | 49.603 | 27.471 |
| Total | | | | 15862458 | 86152 | | |

| # | Peak Name | CH | tR [min] | Area [µV·sec] | Height [µV] | Area% | Height% |
|---|---|---|---|---|---|---|---|
| 1 | Unknown | 1 | 32.457 | 2374 | 18 | 0.091 | 0.238 |
| 2 | Unknown | 1 | 39.837 | 2608811 | 7517 | 99.909 | 99.762 |
| Total | | | | 2611185 | 7535 | | |

**(5a*R*,10b*S*)-6-(Pyrimidin-2-yl)-5-(quinolin-8-yl)-5,5a,6,10b-tetrahydro-4*H*-thieno[3',2':4,5]pyrido[2,3-*b*]indol-4-one (19)**

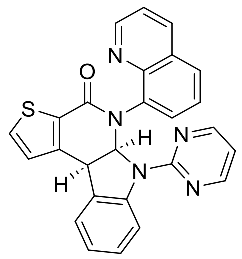

Prepared according to general procedure **A** on a 0.15 mmol scale, column chromatography (ethyl acetate/*n*-hexane = 2:1) afforded the title compound as a pale yellow sticky liquid (41 mg, 0.09 mmol, 61%), with >20:1 dr, 99% ee.

**$^{1}$H NMR** (400 MHz, $CDCl_3$) δ 9.01 (d, *J* = 4.1 Hz, 1H), 8.32 (d, *J* = 8.2 Hz, 1H), 8.11 (d, *J* = 8.2 Hz, 1H), 7.71 (d, *J* = 5.0 Hz, 1H), 7.70 – 7.52 (m, 4H), 7.48 – 7.30 (m, 3H), 7.23 – 7.00 (m, 4H), 6.23 (t, *J* = 4.8 Hz, 1H), 4.95 (d, *J* = 6.8 Hz, 1H) ppm;

**$^{13}$C NMR** (101 MHz, $CDCl_3$) δ 160.9 ($C_q$), 158.9 ($C_q$), 156.4 (CH), 150.4 (CH), 145.9 ($C_q$), 141.7 ($C_q$), 140.0 ($C_q$), 136.6 ($C_q$), 135.8 (CH), 132.8 ($C_q$), 132.3 (CH), 131.1 (CH), 128.9 ($C_q$), 128.3 (CH), 128.2 (CH), 127.1 (CH), 125.6 (CH), 123.8 (CH), 123.2 (CH), 121.1 (CH), 117.5 (CH), 111.9 (CH), 78.3 (CH), 42.1 (CH) ppm;

**IR** (ATR): $\tilde{\nu}$ = 1665, 1581, 1555, 1483, 1433, 1383, 1273, 1150, 795, 749 $cm^{-1}$;

**HRMS (ESI):** *m/z* $[M+H]^+$ calcd for $C_{26}H_{18}N_5OS$: 448.1227; found: 448.1224;

**$[\alpha]^D_{20}$** = –310.9 (c = 1.00, $CHCl_3$);

**$R_t$** (ID-3 column, *n*-hexane/*i*-PrOH 50/500, 1.0 mL/min, 250.4 nm): tr(major) = 30.0 min, tr(minor) = 10.4 min, 99% ee.

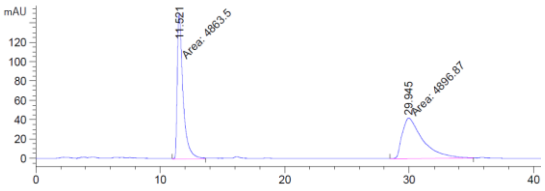


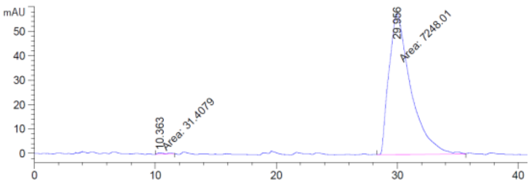


| Peak # | RetTime [min] | Type | Width [min] | Area [mAU*s] | Height [mAU] | Area % |
|---|---|---|---|---|---|---|
| 1 | 11.521 | MM | 0.5366 | 4863.49658 | 151.06131 | 49.8290 |
| 2 | 29.945 | MM | 1.9419 | 4896.87305 | 42.02719 | 50.1710 |

| Peak # | RetTime [min] | Type | Width [min] | Area [mAU*s] | Height [mAU] | Area % |
|---|---|---|---|---|---|---|
| 1 | 10.363 | MM | 0.8257 | 31.40790 | 6.33941e-1 | 0.4315 |
| 2 | 29.956 | MM | 2.0913 | 7248.00781 | 57.76211 | 99.5685 |

**(5a*R*,12c*S*)-5-(Pyrimidin-2-yl)-6-(quinolin-8-yl)-5,5a,6,12c-tetrahydro-7*H*-benzo[4',5']thieno[3',2':4,5]pyrido[2,3-*b*]indol-7-one (20)**

Prepared according to general procedure **A** on a 0.15 mmol scale, column chromatography (ethyl acetate/*n*-hexane = 2:1) afforded the title compound as a pale yellow sticky liquid (50 mg, 0.10 mmol, 67%), with >20:1 dr, 99% ee.

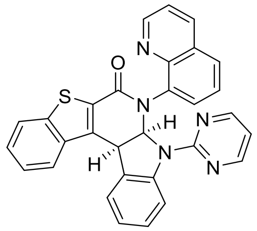

**$^{1}$H NMR** (400 MHz, $CDCl_3$) δ 8.99 (d, *J* = 4.1 Hz, 1H), 8.36 (d, *J* = 8.0 Hz, 1H), 8.12 (d, *J* = 8.2 Hz, 1H), 8.07 – 7.97 (m, 2H), 7.73 – 7.52 (m, 6H), 7.49 – 7.33 (m, 2H), 7.24 (d, *J* = 7.2 Hz, 1H), 7.17 (t, *J* = 7.7 Hz, 1H), 7.09 (d, *J* = 7.4 Hz, 1H), 6.99 (t, *J* = 7.5 Hz, 1H), 6.25 (t, *J* = 4.8 Hz, 1H), 5.25 (d, *J* = 6.9 Hz, 1H) ppm;

**$^{13}$C NMR** (101 MHz, $CDCl_3$) δ 161.6 ($C_q$), 158.9 ($C_q$), 156.5 (CH), 150.5 (CH), 145.8 ($C_q$), 142.7 ($C_q$), 141.9 ($C_q$), 138.3 ($C_q$), 136.6 ($C_q$), 135.8 (CH), 135.3 ($C_q$), 132.6 ($C_q$), 131.7 ($C_q$), 131.0 (CH), 128.9 ($C_q$), 128.5 (CH), 128.3 (CH), 127.0 (CH), 125.6 (CH), 125.2 (CH), 124.0 (CH), 123.7 (CH), 123.3 (CH), 123.2 (CH), 121.2 (CH), 117.6 (CH), 112.0 (CH), 78.5 (CH), 40.9 (CH) ppm;

**IR** (ATR): $\tilde{\nu}$ = 1656, 1581, 1555, 1483, 1432, 1414, 1287, 1150, 795, 748 $cm^{-1}$;

**HRMS (ESI):** *m/z* $[M+H]^+$ calcd for $C_{30}H_{20}N_5OS$: 498.1383; found: 498.1382;

**$[\alpha]^{D}_{20}$** = –452.6 (c = 1.00, $CHCl_3$);

**$R_t$** (ID-3 column, *n*-hexane/*i*-PrOH 50/50, 1.0 mL/min, 250.4 nm): tr(major) = 34.1 min, tr(minor) = 16.5 min, 99% ee.

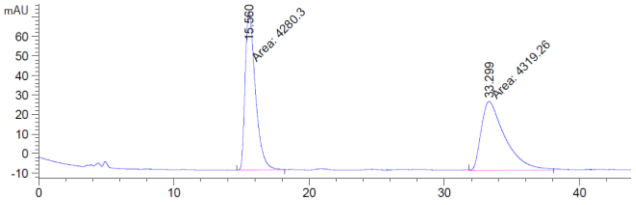


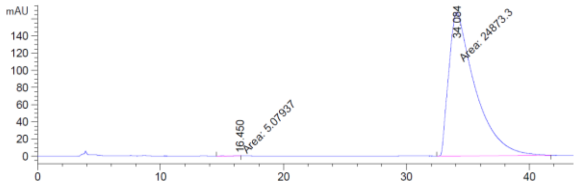


| Peak # | RetTime [min] | Type | Width [min] | Area [mAU*s] | Height [mAU] | Area % |
|---|---|---|---|---|---|---|
| 1 | 15.560 | MM | 0.8787 | 4280.29688 | 81.18289 | 49.7735 |
| 2 | 33.299 | MM | 2.0416 | 4319.25586 | 35.26049 | 50.2265 |

| Peak # | RetTime [min] | Type | Width [min] | Area [mAU*s] | Height [mAU] | Area % |
|---|---|---|---|---|---|---|
| 1 | 16.450 | MM | 0.5292 | 5.07937 | 1.59976e-1 | 0.0204 |
| 2 | 34.084 | MM | 2.4730 | 2.48733e4 | 167.63420 | 99.9796 |

**(6a*R*,11b*R*)-6-(5-Chloroquinolin-8-yl)-7-(pyrimidin-2-yl)-6,6a,7,11b-tetrahydro-5*H*-indolo[2,3-*c*]isoquinolin-5-one (21)**

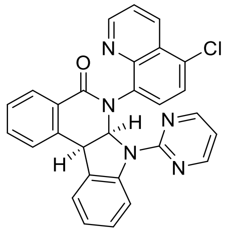

Prepared according to general procedure **A** on a 0.15 mmol scale, column chromatography (ethyl acetate/*n*-hexane = 2:1) afforded the title compound as a pale yellow sticky liquid (57 mg, 0.12 mmol, 80%), with >20:1 dr, 99% ee.

**$^1$H NMR** (400 MHz, $CDCl_3$) δ 9.00 (d, $J$ = 3.9 Hz, 1H), 8.51 (d, $J$ = 8.5 Hz, 1H), 8.34 – 8.23 (m, 2H), 7.74 – 7.64 (m, 3H), 7.57 (d, $J$ = 7.5 Hz, 1H), 7.55 – 7.47 (m, 2H), 7.45 (d, $J$ = 5.1 Hz, 1H), 7.36 (t, $J$ = 7.1 Hz, 1H), 7.25 (s, 1H), 7.08 (d, $J$ = 7.9 Hz, 1H), 7.03 (d, $J$ = 6.2 Hz, 2H), 6.29 (t, $J$ = 4.7 Hz, 1H), 4.83 (d, $J$ = 6.4 Hz, 1H) ppm;

**$^{13}$C NMR** (101 MHz, $CDCl_3$) δ 164.3 ($C_q$), 158.9 ($C_q$), 156.6 (CH), 151.0 (CH), 146.2 ($C_q$), 141.2 ($C_q$), 136.4 ($C_q$), 134.9 ($C_q$), 133.8 ($C_q$), 132.8 (CH), 132.7 (CH), 131.2 ($C_q$), 130.7 (CH), 129.6 (CH), 128.6 (CH), 128.5 (CH), 128.1 (CH), 127.1 ($C_q$), 126.8 ($C_q$), 125.6 (CH), 123.9 (CH), 123.2 (CH), 121.9 (CH), 117.3 (CH), 112.0 (CH), 76.5 (CH), 44.4 (CH) ppm;

**IR** (ATR): $\tilde{\nu}$ = 1657, 1580, 1555, 1482, 1434, 1386, 1285, 938, 753, 746 $cm^{-1}$;

**HRMS (ESI):** $m/z$ $[M+H]^+$ calcd for $C_{28}H_{19}ClN_5O$: 476.1273; found: 476.1265;

**$[\alpha]^D_{20}$** = –185.2 (c = 1.00, $CHCl_3$);

**$R_t$** (AD-3 column, *n*-hexane/*i*-PrOH 70/30, 1.0 mL/min, 250.4 nm): tr(major) = 31.1 min, tr(minor) = 8.4 min, 99% ee.

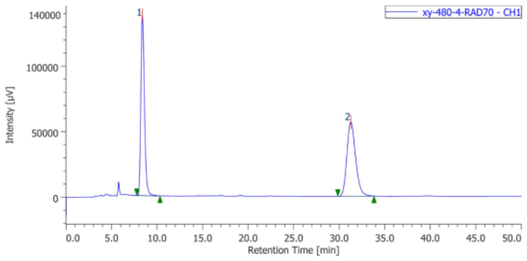


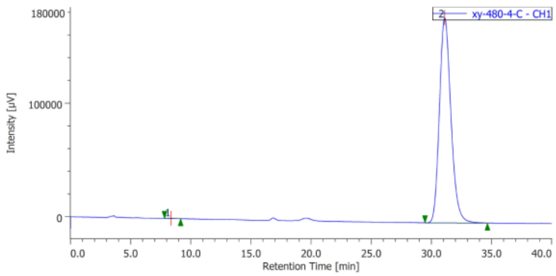


| # | Peak Name | CH | tR [min] | Area [μV·sec] | Height [μV] | Area% | Height% |
|---|---|---|---|---|---|---|---|
| 1 | Unknown | 1 | 8.387 | 3763327 | 137203 | 50.096 | 70.808 |
| 2 | Unknown | 1 | 31.263 | 3748835 | 56565 | 49.904 | 29.192 |
| Total | | | | 7512162 | 193768 | | |

| # | Peak Name | CH | tR [min] | Area [μV·sec] | Height [μV] | Area% | Height% |
|---|---|---|---|---|---|---|---|
| 1 | Unknown | 1 | 8.357 | 8296 | 276 | 0.069 | 0.152 |
| 2 | Unknown | 1 | 31.100 | 12051572 | 180732 | 99.931 | 99.848 |
| Total | | | | 12059868 | 181008 | | |

**(6a*R*,11b*R*)-2-Acetyl-6-(5-methylquinolin-8-yl)-7-(pyrimidin-2-yl)-6,6a,7,11b-tetrahydro-5*H*-indolo[2,3-*c*]isoquinolin-5-one (22)**

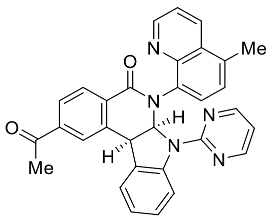


Prepared according to general procedure **A** on a 0.15 mmol scale, column chromatography (ethyl acetate/*n*-hexane = 2:1) afforded the title compound as a pale yellow sticky liquid (52 mg, 0.11 mmol, 70%), with >20:1 dr, 99% ee.

**$^{1}$H NMR** (400 MHz, $CDCl_3$) δ 8.89 (d, $J$ = 4.1 Hz, 1H), 8.42 – 8.34 (m, 2H), 8.19 (s, 1H), 8.06 (d, $J$ = 8.0 Hz, 1H), 8.01 (d, $J$ = 8.3 Hz, 1H), 7.71 (d, $J$ = 4.8 Hz, 2H), 7.51 (d, $J$ = 6.3 Hz, 1H), 7.45 – 7.32 (m, 3H), 7.10 – 6.94 (m, 3H), 6.28 (t, $J$ = 4.7 Hz, 1H), 4.91 (d, $J$ = 6.4 Hz, 1H), 2.77 (s, 3H), 2.24 (s, 3H) ppm;

**$^{13}$C NMR** (101 MHz, $CDCl_3$) δ 197.7 ($C_q$), 163.2 ($C_q$), 159.1 ($C_q$), 156.4 (CH), 149.7 (CH), 143.9 ($C_q$), 141.3 ($C_q$), 140.0 ($C_q$), 136.2 ($C_q$), 135.5 ($C_q$), 135.5 ($C_q$), 135.1 (CH), 133.3 ($C_q$), 133.0 (CH), 131.0 ($C_q$), 130.1 (CH), 128.9 ($C_q$), 128.7 (CH), 128.1 (CH), 128.0 (CH), 127.0 (CH), 123.9 (CH), 123.1 (CH), 121.2 (CH), 117.2 (CH), 112.0 (CH), 76.2 (CH), 44.2 (CH), 27.1 ($CH_3$), 21.1 ($CH_3$) ppm;

**IR** (ATR): $\tilde{\nu}$ = 1688, 1657, 1581, 1554, 1482, 1429, 1386, 1168, 790, 752 $cm^{-1}$;

**HRMS (ESI):** *m/z* $[M+H]^+$ calcd for $C_{31}H_{24}N_5O_2$: 498.1925; found: 498.1922;

**$[\alpha]^{D}_{20}$** = –322.2 (c = 0.50, $CHCl_3$);

**$R_t$** (OD-3 column, *n*-hexane/*i*-PrOH 80/20, 1.0 mL/min, 250.4 nm): tr(major) = 41.5 min, tr(minor) = 51.4 min, 99% ee.

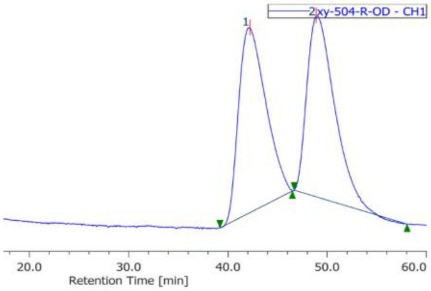


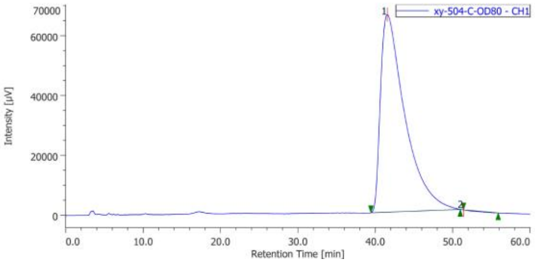


| # | Peak Name | CH | tR [min] | Area [µV·sec] | Height [µV] | Area% | Height% |
|---|---|---|---|---|---|---|---|
| 1 | Unknown | 1 | 42.193 | 2362207 | 12417 | 49.887 | 50.486 |
| 2 | Unknown | 1 | 48.883 | 2372861 | 12178 | 50.113 | 49.514 |
| Total | | | | 4735068 | 24595 | | |

| # | Peak Name | CH | tR [min] | Area [µV·sec] | Height [µV] | Area% | Height% |
|---|---|---|---|---|---|---|---|
| 1 | Unknown | 1 | 41.540 | 14443397 | 66016 | 100.000 | 99.986 |
| 2 | Unknown | 1 | 51.390 | 9 | 9 | 0.000 | 0.014 |
| Total | | | | 14443406 | 66025 | | |

**(6a*R*,11b*R*)-2-acetyl-7-(pyrimidin-2-yl)-6-(quinolin-8-yl)-6,6a,7,11b-tetrahydro-5*H*-indolo[2,3-*c*]isoquinolin-5-one (41)**

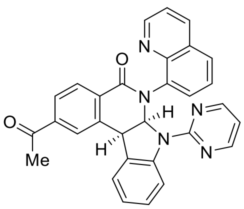


Prepared according to general procedure **A** on a 0.15 mmol scale, column chromatography (ethyl acetate/*n*-hexane = 2:1) afforded the title compound as a pale yellow sticky liquid (49 mg, 0.11 mmol, 67%), with >20:1 dr, 99% ee.

**¹H NMR** (400 MHz, $CDCl_3$) δ 8.97 (d, *J* = 3.0 Hz, 1H), 8.38 (d, *J* = 8.0 Hz, 1H), 8.33 (d, *J* = 8.0 Hz, 1H), 8.19 (s, 1H), 8.13 (d, *J* = 7.5 Hz, 1H), 8.06 (d, *J* = 9.7 Hz, 1H), 7.68 (d, *J* = 4.7 Hz, 2H), 7.61 (dd, *J* = 5.6, 4.1 Hz, 1H), 7.54 (d, *J* = 6.4 Hz, 1H), 7.44 (dd, *J* = 8.3, 4.2 Hz, 1H), 7.39 (t, *J* = 7.6 Hz, 1H), 7.22 – 7.15 (m, 2H), 7.09 – 6.97 (m, 2H), 6.26 (t, *J* = 4.8 Hz, 1H), 4.92 (d, *J* = 6.4 Hz, 1H), 2.77 (s, 3H) ppm;

**¹³C NMR** (101 MHz, $CDCl_3$) δ 197.7 ($C_q$), 163.3 ($C_q$), 158.9 ($C_q$), 156.5 (CH), 150.5 (CH), 145.3 ($C_q$), 141.2 ($C_q$), 140.0 ($C_q$), 136.6 ($C_q$), 136.0 (CH), 135.5 ($C_q$), 133.3 ($C_q$), 131.0 ($C_q$), 130.9 (CH), 130.1 (CH), 128.9 ($C_q$), 128.6 (CH), 128.3 (CH), 128.1 (CH), 128.0 (CH), 125.6 (CH), 123.9 (CH), 123.2 (CH), 121.3 (CH), 117.4 (CH), 112.0 (CH), 76.3 (CH), 44.3 (CH), 27.1 ($CH_3$) ppm;

**IR** (ATR): $\tilde{\nu}$ = 1688, 1657, 1581, 1555, 1482, 1429, 1384, 1168, 790, 752 $cm^{-1}$;

**HRMS (ESI):** *m/z* $[M+H]^+$ calcd for $C_{30}H_{22}N_5O_2$: 484.1768; found: 484.1766;

**$[\alpha]^D_{20}$** = –304.6 (c = 0.50, $CHCl_3$);

**$R_t$** (OD-3 column, *n*-hexane/*i*-PrOH 60/40, 1.0 mL/min, 273.0 nm): tr(major) = 26.3 min, tr(minor) = 16.3 min, 99% ee.

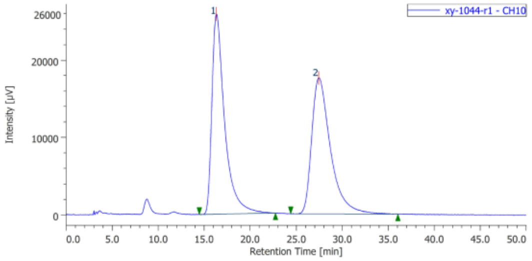


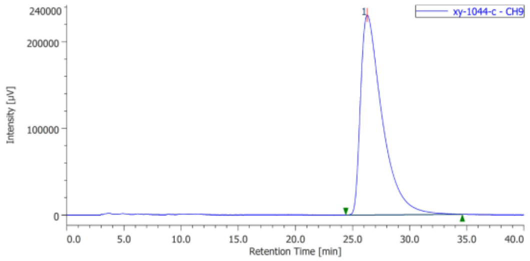


| # | Peak Name | CH | tR [min] | Area [μV·sec] | Height [μV] | Area% |
|---|---|---|---|---|---|---|
| 1 | Unknown | 10 | 16.337 | 2484472 | 25809 | 50.104 |
| 2 | Unknown | 10 | 27.417 | 2474203 | 17538 | 49.896 |

| # | Peak Name | CH | tR [min] | Area [μV·sec] | Height [μV] | Area% |
|---|---|---|---|---|---|---|
| 1 | Unknown | 9 | 26.270 | 30372767 | 230833 | 100.000 |

**(6a*R*,11b*R*)-7-(Pyrimidin-2-yl)-6-(6-(trifluoromethyl)quinolin-8-yl)-6,6a,7,11b-tetrahydro-5*H*-indolo[2,3-*c*]isoquinolin-5-one (23)**

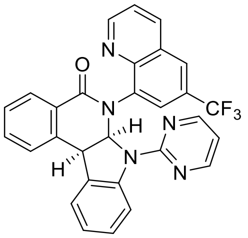

Prepared according to general procedure **A** on a 0.15 mmol scale, column chromatography (ethyl acetate/*n*-hexane = 2:1) afforded the title compound as a pale yellow sticky liquid (56 mg, 0.11 mmol, 73%), with 17:1 dr, 99% ee.

**$^{1}$H NMR** (400 MHz, $CDCl_3$) δ 9.08 (d, *J* = 3.5 Hz, 1H), 8.36 (d, *J* = 8.0 Hz, 1H), 8.28 (d, *J* = 7.8 Hz, 1H), 8.22 (d, *J* = 8.3 Hz, 1H), 7.91 (s, 1H), 7.77 – 7.65 (m, 3H), 7.63 – 7.49 (m, 3H), 7.44 – 7.36 (m, 2H), 7.34 (s, 1H), 7.06 (q, *J* = 7.7 Hz, 2H), 6.27 (t, *J* = 4.5 Hz, 1H), 4.86 (d, *J* = 6.4 Hz, 1H) ppm;

**$^{13}$C NMR** (101 MHz, $CDCl_3$) δ 164.21 ($C_q$), 158.82 ($C_q$), 156.58 (CH), 152.56 (CH), 146.77 ($C_q$), 141.07 ($C_q$), 138.69 ($C_q$), 136.72 (CH), 134.93 ($C_q$), 133.60 ($C_q$), 132.77 (CH), 129.59 (CH), 128.68 (CH), 128.60 (CH), 128.12 (CH), 127.83 ($C_q$), 127.69 (d, $J_{CF}$ = 33.0 Hz, $C_q$), 126.94 ($C_q$), 126.79 (q, $J_{CF}$ = 3.0 Hz, CH), 125.78 (q, $J_{CF}$ = 4.4 Hz, CH), 123.90 (CH), 123.36 (q, $J_{CF}$ = 272.6 Hz, $C_q$), 123.26 (CH), 122.31 (CH), 117.37 (CH), 111.96 (CH), 76.45 (CH), 44.39 (CH) ppm;

**$^{19}$F NMR** (377 MHz, $CDCl_3$) δ -62.65 ppm;

**IR** (ATR): $\tilde{\nu}$ = 1660, 1581, 1555, 1482, 1461, 1345, 1287, 1128, 795, 749 $cm^{-1}$;

**HRMS (ESI):** *m/z* $[M+H]^+$ calcd for $C_{29}H_{19}F_3N_5O$: 510.1536; found: 510.1531;

**$[\alpha]^{D}_{20}$** = –245.6 (c = 0.50, $CHCl_3$);

**$R_t$** (AD-3 column, *n*-hexane/*i*-PrOH 70/30, 1.0 mL/min, 250.4 nm): tr(major) = 5.3 min, tr(minor) = 8.6 min, 99% ee.

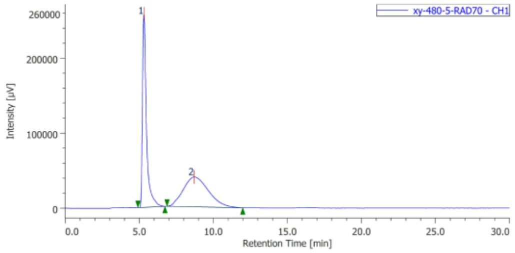


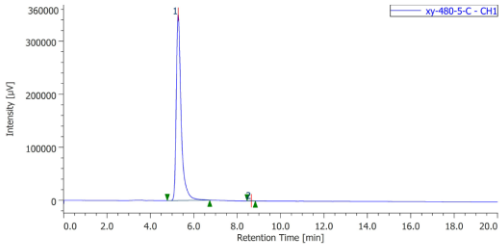


| # | Peak Name | CH | tR [min] | Area [µV·sec] | Height [µV] | Area% | Height% |
|---|---|---|---|---|---|---|---|
| 1 | Unknown | 1 | 5.307 | 4826715 | 257749 | 50.655 | 86.612 |
| 2 | Unknown | 1 | 8.700 | 4701956 | 39841 | 49.345 | 13.388 |
| Total | | | | 9528671 | 297590 | | |

| # | Peak Name | CH | tR [min] | Area [µV·sec] | Height [µV] | Area% | Height% |
|---|---|---|---|---|---|---|---|
| 1 | Unknown | 1 | 5.277 | 5508224 | 351043 | 99.891 | 99.858 |
| 2 | Unknown | 1 | 8.647 | 6009 | 500 | 0.109 | 0.142 |
| Total | | | | 5514233 | 351543 | | |

**(6a*R*,11b*R*)-11-Methyl-7-(pyrimidin-2-yl)-6-(quinolin-8-yl)-6,6a,7,11b-tetrahydro-5*H*-indolo[2,3-*c*]isoquinolin-5-one (24)**

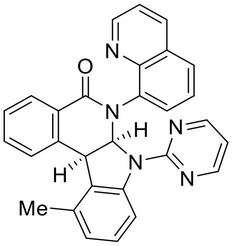

Prepared according to general procedure **A** on a 0.15 mmol scale, column chromatography (ethyl acetate/*n*-hexane = 2:1) afforded the title compound as a pale yellow sticky liquid (43 mg, 0.09 mmol, 63%), with >20:1 dr, 99% ee.

**$^{1}$H NMR** (400 MHz, $CDCl_3$) δ 8.93 (s, 1H), 8.28 – 8.15 (m, 2H), 8.07 (d, *J* = 8.2 Hz, 1H), 7.77 – 7.46 (m, 6H), 7.45 – 7.35 (m, 2H), 7.30 – 7.13 (m, 3H), 6.78 (d, *J* = 7.6 Hz, 1H), 6.22 (s, 1H), 4.92 (d, *J* = 5.4 Hz, 1H), 1.97 (s, 3H) ppm;

**$^{13}$C NMR** (101 MHz, $CDCl_3$) δ 164.7 ($C_q$), 158.8 ($C_q$), 156.4 (CH), 150.3 (CH), 145.6 ($C_q$), 141.6 ($C_q$), 136.7 ($C_q$), 135.7 (CH), 135.6 ($C_q$), 135.1 ($C_q$), 131.5 (CH), 131.1 (CH), 131.0 (CH), 130.8 ($C_q$), 129.1 (CH), 128.8 ($C_q$), 128.3 (CH), 128.2 (CH), 128.0 (CH), 126.2 (CH), 125.6 (CH), 121.1 (CH), 114.9 (CH), 111.8 (CH), 76.9 (CH), 46.5 (CH), 19.9 ($CH_3$) ppm;

**IR** (ATR): $\tilde{\nu}$ = 1656, 1579, 1555, 1471, 1460, 1414, 1398, 1289, 794, 748 $cm^{-1}$;

**HRMS (ESI):** *m/z* $[M+H]^+$ calcd for $C_{29}H_{22}N_5O$: 456.1819; found: 456.1813;

**$[\alpha]^{D}_{20}$** = –155.4 (c = 0.50, $CHCl_3$);

**$R_t$** (ID-3 column, *n*-hexane/*i*-PrOH 50/50, 1.0 mL/min, 250.4 nm): tr(major) = 60.3 min, tr(minor) = 10.5 min, 99% ee.

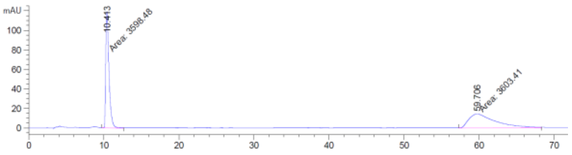


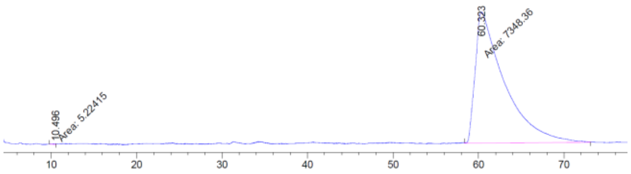


| Peak # | RetTime [min] | Type | Width [min] | Area [mAU*s] | Height [mAU] | Area % |
|---|---|---|---|---|---|---|
| 1 | 10.413 | MM | 0.5031 | 3598.47949 | 119.19971 | 49.9658 |
| 2 | 59.706 | MM | 4.1754 | 3603.41016 | 14.38344 | 50.0342 |

| Peak # | RetTime [min] | Type | Width [min] | Area [mAU*s] | Height [mAU] | Area % |
|---|---|---|---|---|---|---|
| 1 | 10.496 | MM | 0.4530 | 5.22415 | 1.92200e-1 | 0.0710 |
| 2 | 60.323 | MM | 3.9564 | 7348.35547 | 30.95551 | 99.9290 |

**(6a*R*,11b*R*)-10-Methyl-7-(pyrimidin-2-yl)-6-(quinolin-8-yl)-6,6a,7,11b-tetrahydro-5*H*-indolo[2,3-*c*]isoquinolin-5-one (25)**

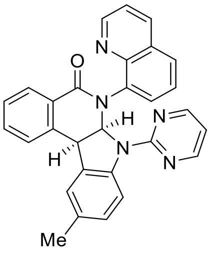

Prepared according to general procedure **A** on a 0.15 mmol scale, column chromatography (ethyl acetate/*n*-hexane = 2:1) afforded the title compound as a pale yellow sticky liquid (48 mg, 0.11 mmol, 70%), with >20:1 dr, 99% ee.

**$^1$H NMR** (400 MHz, $CDCl_3$) δ 8.95 (s, 1H), 8.40 – 8.03 (m, 3H), 7.81 – 7.33 (m, 8H), 7.22 – 7.08 (m, 3H), 6.81 (s, 1H), 6.19 (s, 1H), 4.80 (s, 1H), 2.30 (s, 3H) ppm;

**$^{13}$C NMR** (101 MHz, $CDCl_3$) δ 164.2 ($C_q$), 159.0 ($C_q$), 156.5 (CH), 150.4 (CH), 145.7 ($C_q$), 138.9 ($C_q$), 137.1 ($C_q$), 135.7 (CH), 135.1 ($C_q$), 134.0 ($C_q$), 132.7 ($C_q$), 132.5 (CH), 130.9 (CH), 129.6 (CH), 128.9 ($C_q$), 128.7 (CH), 128.5 (CH), 128.0 (CH), 128.0 (CH), 127.3 ($C_q$), 125.6 (CH), 124.6 (CH), 121.1 (CH), 117.0 (CH), 111.6 (CH), 76.5 (CH), 44.4 (CH), 21.1 ($CH_3$) ppm;

**IR** (ATR): $\tilde{\nu}$ = 1659, 1580, 1554, 1488, 1464, 1416, 1290, 1162, 795, 755 $cm^{-1}$;

**HRMS (ESI):** *m/z* $[M+H]^+$ calcd for $C_{29}H_{22}N_5O$: 456.1819; found: 456.1812;

**$[\alpha]^D_{20}$** = –212.3 (c = 1.00, $CHCl_3$);

**$R_t$** (ID-3 column, *n*-hexane/*i*-PrOH 50/50, 1.0 mL/min, 250.4 nm): tr(major) = 19.3 min, tr(minor) = 9.7 min, 99% ee.

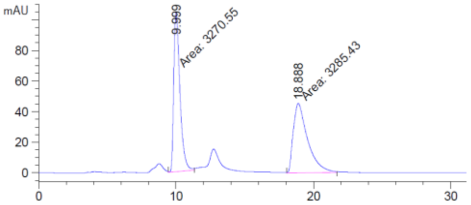


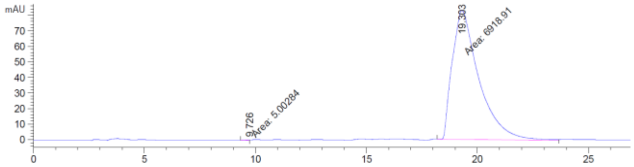


| Peak # | RetTime [min] | Type | Width [min] | Area [mAU*s] | Height [mAU] | Area % |
|---|---|---|---|---|---|---|
| 1 | 9.999 | MM | 0.5225 | 3270.55371 | 104.31578 | 49.8866 |
| 2 | 18.888 | MM | 1.2051 | 3285.42871 | 45.43853 | 50.1134 |

| Peak # | RetTime [min] | Type | Width [min] | Area [mAU*s] | Height [mAU] | Area % |
|---|---|---|---|---|---|---|
| 1 | 9.726 | MM | 0.2283 | 5.00284 | 3.65240e-1 | 0.0723 |
| 2 | 19.303 | MM | 1.3898 | 6918.90820 | 82.97143 | 99.9277 |

**(6a*R*,11b*R*)-10-Chloro-7-(pyrimidin-2-yl)-6-(quinolin-8-yl)-6,6a,7,11b-tetrahydro-5*H*-indolo[2,3-*c*]isoquinolin-5-one (26)**

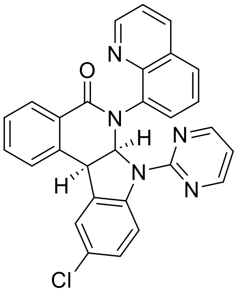

Prepared according to general procedure **A** on a 0.15 mmol scale, column chromatography (*n*-hexane/ethyl acetate = 1:2) afforded the title compound as a pale yellow sticky liquid (32 mg, 0.07 mmol, 45%), with >20:1 dr, 99% ee.

**$^1$H NMR** (400 MHz, $CDCl_3$) δ 8.96 (d, *J* = 4.2 Hz, 1H), 8.28 (t, *J* = 8.7 Hz, 2H), 8.10 (d, *J* = 8.2 Hz, 1H), 7.72 (t, *J* = 7.5 Hz, 1H), 7.66 (d, *J* = 4.8 Hz, 2H), 7.59 – 7.52 (m, 4H), 7.41 (dd, *J* = 8.6, 4.1 Hz, 1H), 7.32 (d, *J* = 8.7 Hz, 1H), 7.18 – 7.19 (m, 2H), 6.97 (s, 1H), 6.25 (t, *J* = 4.8 Hz, 1H), 4.82 (d, *J* = 6.5 Hz, 1H) ppm;

**$^{13}$C NMR** (101 MHz, $CDCl_3$) δ 164.0 ($C_q$), 158.6 ($C_q$), 156.4 (CH), 150.4 (CH), 145.5 ($C_q$), 139.9 ($C_q$), 136.8 ($C_q$), 135.8 ($C_q$), 135.7 (CH), 134.0 ($C_q$), 132.7 (CH), 130.7 (CH), 129.7 (CH), 128.8 ($C_q$), 128.3 (CH), 128.3 (CH), 128.2 (CH), 128.1 (CH), 127.9 ($C_q$), 127.1 ($C_q$), 125.5 (CH), 124.1 (CH), 121.1 (CH), 118.1 (CH), 112.1 (CH), 76.4 (CH), 44.2 (CH) ppm.

**IR** (ATR): $\tilde{\nu}$ = 3038, 1710, 1657, 1578, 1476, 1411, 1286, 1153, 794, 756 $cm^{-1}$;

**HRMS (ESI):** *m/z* $[M+H]^+$ calcd for $C_{28}H_{19}ClN_5O$: 476.1273; found: 476.1270;

**$[\alpha]^D_{20}$** = –162.0 (c = 0.50, $CHCl_3$);

**$R_t$** (ID-3 column, *n*-hexane/*i*-PrOH 50/50, 1.0 mL/min, 250.4 nm): tr(major) = 27.8 min, tr(minor) = 11.2 min, 99% ee.

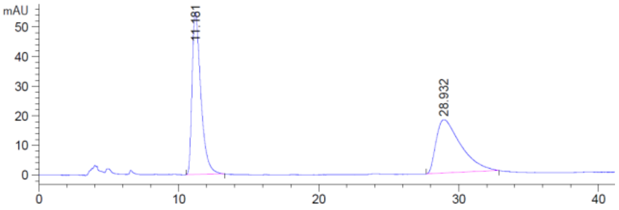


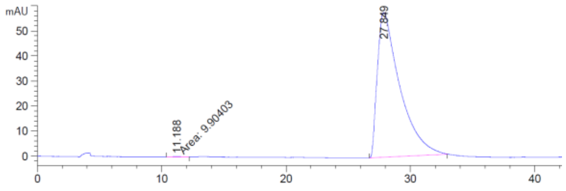


| Peak # | RetTime [min] | Type | Width [min] | Area [mAU*s] | Height [mAU] | Area % |
|---|---|---|---|---|---|---|
| 1 | 11.181 | BB | 0.6095 | 2382.79053 | 54.88533 | 51.7643 |
| 2 | 28.932 | BB | 1.4381 | 2220.36255 | 18.08871 | 48.2357 |

| Peak # | RetTime [min] | Type | Width [min] | Area [mAU*s] | Height [mAU] | Area % |
|---|---|---|---|---|---|---|
| 1 | 11.188 | MM | 0.8476 | 9.90403 | 1.94743e-1 | 0.1368 |
| 2 | 27.849 | BB | 1.4647 | 7228.16455 | 57.69373 | 99.8632 |

**(6a*R*,11b*R*)-10-Bromo-7-(pyrimidin-2-yl)-6-(quinolin-8-yl)-6,6a,7,11b-tetrahydro-5*H*-indolo[2,3-*c*]isoquinolin-5-one (27)**

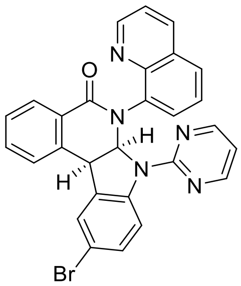

Prepared according to general procedure **A** on a 0.15 mmol scale, column chromatography (ethyl acetate/*n*-hexane = 2:1) afforded the title compound as a pale yellow sticky liquid (27 mg, 0.05 mmol, 35%), with >20:1 dr, 99% ee.

**$^{1}$H NMR** (400 MHz, $CDCl_3$) δ 8.95 (d, *J* = 4.0 Hz, 1H), 8.29 (d, *J* = 7.7 Hz, 1H), 8.22 (d, *J* = 8.5 Hz, 1H), 8.10 (d, *J* = 8.2 Hz, 1H), 7.77 – 7.63 (m, 3H), 7.61 – 7.34 (m, 6H), 7.20 – 7.06 (m, 3H), 6.25 (t, *J* = 4.9 Hz, 1H), 4.83 (d, *J* = 6.4 Hz, 1H) ppm;

**$^{13}$C NMR** (101 MHz, $CDCl_3$) δ 164.1 ($C_q$), 158.7 ($C_q$), 156.5 (CH), 150.5 (CH), 145.5 ($C_q$), 140.5 ($C_q$), 136.9 ($C_q$), 136.3 ($C_q$), 135.8 (CH), 134.0 ($C_q$), 132.8 (CH), 131.3 (CH), 130.8 (CH), 129.8

(CH), 128.9 ($C_q$), 128.4 (CH), 128.4 (CH), 128.2 (CH), 127.2 ($C_q$), 127.0 (CH), 125.6 (CH), 121.2 (CH), 118.7 (CH), 115.4 ($C_q$), 112.2 (CH), 76.4 (CH), 44.2 (CH) ppm;

**IR** (ATR): $\tilde{\nu}$ = 1656, 1577, 1555, 1477, 1439, 1407, 1287, 1236, 794, 754 $cm^{-1}$;

**HRMS (ESI):** *m/z* $[M+H]^+$ calcd for $C_{28}H_{19}BrN_5O$: 520.0767; found: 520.0762;

$[\alpha]^D_{20}$ = –128.2 (c = 0.05, $CHCl_3$);

$R_t$ (ID-3 column, *n*-hexane/*i*-PrOH 50/50, 1.0 mL/min, 250.4 nm): tr(major) = 21.5 min, tr(minor) = 10.9min, 99% ee.

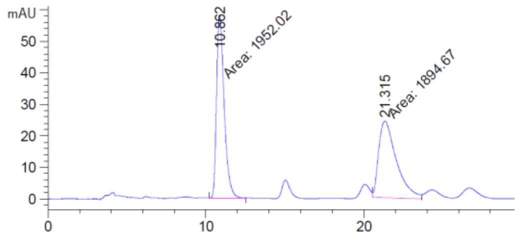


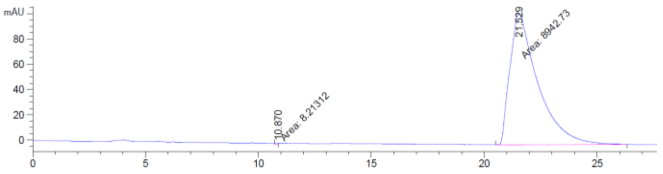


| Peak # | RetTime [min] | Type | Width [min] | Area [mAU*s] | Height [mAU] | Area % |
|---|---|---|---|---|---|---|
| 1 | 10.862 | MM | 0.5598 | 1952.02051 | 58.12019 | 50.7454 |
| 2 | 21.315 | MM | 1.3001 | 1894.67261 | 24.28886 | 49.2546 |

| Peak # | RetTime [min] | Type | Width [min] | Area [mAU*s] | Height [mAU] | Area % |
|---|---|---|---|---|---|---|
| 1 | 10.870 | MM | 0.1494 | 8.21312 | 6.55778e-1 | 0.0918 |
| 2 | 21.529 | MM | 1.4294 | 8942.72754 | 104.27115 | 99.9082 |

**(6a*R*,11b*R*)-10-(Benzyloxy)-7-(pyrimidin-2-yl)-6-(quinolin-8-yl)-6,6a,7,11b-tetrahydro-5*H*-indolo[2,3-*c*]isoquinolin-5-one (28)**

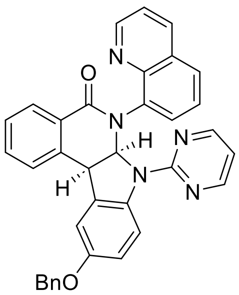

Prepared according to general procedure **A** on a 0.15 mmol scale, column chromatography (*n*-hexane/ethyl acetate = 1:2) afforded the title compound as a pale yellow sticky liquid (38 mg, 0.07 mmol, 46%), with >20:1 dr, 99% ee.

$^1$**H NMR** (400 MHz, $CDCl_3$) δ 9.02 (d, *J* = 4.2 Hz, 1H), 8.28 (d, *J* = 7.7 Hz, 1H), 8.18 (d, *J* = 8.7 Hz, 1H), 8.10 (d, *J* = 8.2 Hz, 1H), 7.68 – 7.47 (m, 7H), 7.36 (m, 6H), 7.19 – 7.12 (m, 2H), 6.93 (d, *J* = 8.7 Hz, 1H), 6.68 (s, 1H), 6.17 (t, *J* = 4.7 Hz, 1H), 5.00 (s, 2H), 4.79 (d, *J* = 6.3 Hz, 1H) ppm;

$^{13}$**C NMR** (101 MHz, $CDCl_3$) δ 164.1 ($C_q$), 158.7 ($C_q$), 156.4 (CH), 155.2 ($C_q$), 150.3 (CH), 145.4 ($C_q$), 137.0 ($C_q$), 136.7 ($C_q$), 136.0 (CH), 135.5 ($C_q$), 134.9 ($C_q$), 134.6 ($C_q$), 132.4 (CH), 131.0 (CH), 129.5 (CH), 128.8 ($C_q$), 128.5 (CH), 128.3 (CH), 128.0 (CH), 127.96 (CH), 127.9 (CH),

127.6 (CH), 127.2 ($C_q$), 125.5 (CH), 121.1 (CH), 117.7 (CH), 113.2 (CH), 112.1 (CH), 111.4 (CH), 76.4 (CH), 70.6 ($CH_2$), 44.4 (CH) ppm.

**IR** (ATR): $\tilde{\nu}$ = 3038, 1658, 1581, 1485, 1423, 1383, 1288, 1271, 1023, 794 $cm^{-1}$;

**HRMS (ESI):** *m/z* $[M+H]^+$ calcd for $C_{35}H_{26}N_5O_2$: 548.2081; found: 548.2077;

$[\alpha]^D_{20}$ = –161.7 (c = 0.30, $CHCl_3$);

**$R_t$** (AD-3 column, *n*-hexane/*i*-PrOH 70/30, 1.0 mL/min, 250.4 nm): tr(major) = 21.9 min, tr(minor) = 17.7 min, 99% ee.

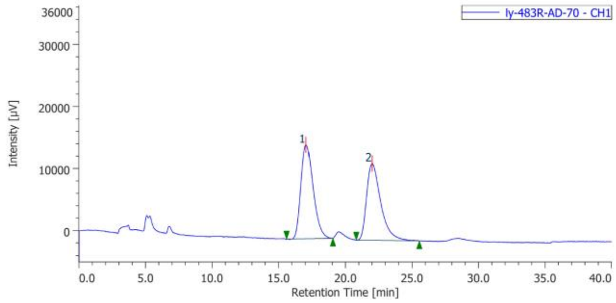


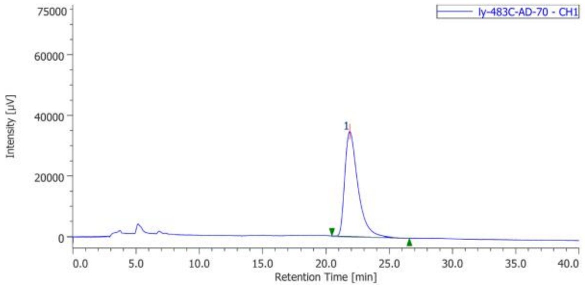


| # | Peak Name | CH | tR [min] | Area [µV·sec] | Height [µV] | Area% | Height% |
|---|---|---|---|---|---|---|---|
| 1 | Unknown | 1 | 17.050 | 951648 | 15137 | 50.987 | 55.014 |
| 2 | Unknown | 1 | 22.003 | 914787 | 12378 | 49.013 | 44.986 |
| Total | | | | 1866435 | 27515 | | |

| # | Peak Name | CH | tR [min] | Area [µV·sec] | Height [µV] | Area% | Height% |
|---|---|---|---|---|---|---|---|
| 1 | Unknown | 1 | 21.867 | 2490373 | 34572 | 100.000 | 100.000 |
| Total | | | | 2490373 | 34572 | | |

**(6a*R*,11b*R*)-10-Methoxy-7-(pyrimidin-2-yl)-6-(quinolin-8-yl)-6,6a,7,11b-tetrahydro-5*H*-indolo[2,3-*c*]isoquinolin-5-one (29)**

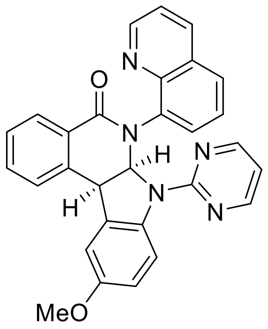

Prepared according to general procedure **A** on a 0.15 mmol scale, column chromatography (ethyl acetate/*n*-hexane = 2:1) afforded the title compound as a pale yellow sticky liquid (41 mg, 0.09 mmol, 58%), with >20:1 dr, 99% ee.

**$^1$H NMR** (400 MHz, $CDCl_3$) δ 8.95 (s, 1H), 8.32 – 8.03 (m, 3H), 7.75 – 7.33 (m, 9H), 7.17 (s, 2H), 6.88 (s, 1H), 6.59 (s, 1H), 6.19 (s, 1H), 4.81 (s, 1H), 3.77 (s, 3H) ppm;

**$^{13}$C NMR** (101 MHz, $CDCl_3$) δ 164.1 ($C_q$), 158.8 ($C_q$), 156.5 (CH), 156.1 ($C_q$), 150.4 (CH), 145.6 ($C_q$), 137.0 ($C_q$), 135.8 (CH), 135.5 ($C_q$), 134.9 ($C_q$), 134.8 ($C_q$), 132.5 (CH), 130.8 (CH), 129.6

(CH), 128.9 ($C_q$), 128.4 (CH), 128.1 (CH), 127.3 ($C_q$), 125.6 (CH), 121.1 (CH), 117.9 (CH), 112.5 (CH), 111.4 (CH), 111.0 (CH), 76.5 (CH), 55.9 ($CH_3$), 44.5 (CH) ppm;

**IR** (ATR): $\tilde{\nu}$ = 1658, 1580, 1554, 1487, 1462, 1422, 1289, 1271, 794, 756 $cm^{-1}$;

**HRMS (ESI):** *m/z* $[M+H]^+$ calcd for $C_{29}H_{21}N_5O_2$: 472.1768; found: 472.1763;

$[\alpha]^D_{20}$ = –196.2 (c = 0.50, $CHCl_3$);

**$R_t$** (ID-3 column, *n*-hexane/*i*-PrOH 50/50, 1.0 mL/min, 250.4 nm): tr(major) = 59.5 min, tr(minor) = 13.9 min, 99% ee.

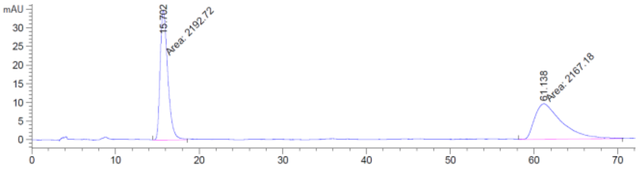


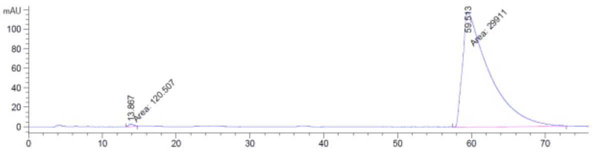


| Peak # | RetTime [min] | Type | Width [min] | Area [mAU*s] | Height [mAU] | Area % |
|---|---|---|---|---|---|---|
| 1 | 15.702 | MM | 1.0513 | 2192.71582 | 34.76276 | 50.2929 |
| 2 | 61.138 | MM | 3.7748 | 2167.17627 | 9.56851 | 49.7071 |

| Peak # | RetTime [min] | Type | Width [min] | Area [mAU*s] | Height [mAU] | Area % |
|---|---|---|---|---|---|---|
| 1 | 13.867 | MM | 0.7764 | 120.50748 | 2.58689 | 0.4013 |
| 2 | 59.513 | MM | 4.2220 | 2.99110e4 | 118.07565 | 99.5987 |

**(6a*R*,11b*R*)-10-Cyclopropyl-7-(pyrimidin-2-yl)-6-(quinolin-8-yl)-6,6a,7,11b-tetrahydro-5*H*-indolo[2,3-*c*]isoquinolin-5-one (30)**

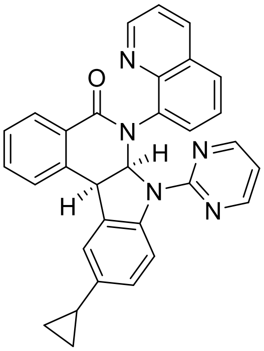

Prepared according to general procedure **A** on a 0.15 mmol scale, column chromatography (ethyl acetate/*n*-hexane = 2:1) afforded the title compound as a pale yellow sticky liquid (37 mg, 0.08 mmol, 51%), with >20:1 dr, 99% ee.

**$^1$H NMR** (400 MHz, $CDCl_3$) δ 8.96 (d, *J* = 4.2 Hz, 1H), 8.28 (d, *J* = 7.7 Hz, 1H), 8.16 (d, *J* = 8.2 Hz, 1H), 8.10 (d, *J* = 8.2 Hz, 1H), 7.71 (t, *J* = 7.5 Hz, 1H), 7.64 (d, *J* = 4.8 Hz, 2H), 7.60 – 7.55 (m, 2H), 7.54 – 7.47 (m, 2H), 7.40 (dd, *J* = 8.4, 4.2 Hz, 1H), 7.16 (d, *J* = 4.8 Hz, 2H), 7.05 (d, *J* = 8.4 Hz, 1H), 6.75 (s, 1H), 6.20 (t, *J* = 4.8 Hz, 1H), 4.80 (d, *J* = 6.3 Hz, 1H), 1.91 – 1.80 (m, 1H), 0.90 (d, *J* = 7.8 Hz, 2H), 0.68 – 0.55 (m, 2H) ppm;

**$^{13}$C NMR** (101 MHz, $CDCl_3$) δ 164.2 ($C_q$), 158.9 ($C_q$), 156.4 (CH), 150.4 (CH), 145.6 ($C_q$), 139.0 ($C_q$), 138.8 ($C_q$), 137.0 ($C_q$), 135.8 (CH), 135.0 ($C_q$), 134.0 ($C_q$), 132.5 (CH), 130.9 (CH), 129.6 (CH), 128.9 ($C_q$), 128.5 (CH), 128.0 (CH), 128.0 (CH), 127.3 ($C_q$), 125.6 (CH), 125.4 (CH), 121.8

(CH), 121.1 (CH), 117.1 (CH), 111.6 (CH), 76.5 (CH), 44.4 (CH), 15.2 (CH), 8.9 ($CH_2$), 8.7 ($CH_2$) ppm;

**IR** (ATR): $\tilde{\nu}$ = 1658, 1580, 1555, 1487, 1434, 1383, 1289, 1271, 795, 749 $cm^{-1}$;

**HRMS (ESI):** *m/z* $[M+H]^+$ calcd for $C_{31}H_{24}N_5O$: 482.1975; found: 482.1969;

**$[\alpha]^D_{20}$** = –129.0 (c = 0.50, $CHCl_3$);

**$R_t$** (OD-3 column, *n*-hexane/*i*-PrOH 70/30, 1.0 mL/min, 250.4 nm): tr(major) = 14.4 min, tr(minor) = 8.8 min, 99% ee.

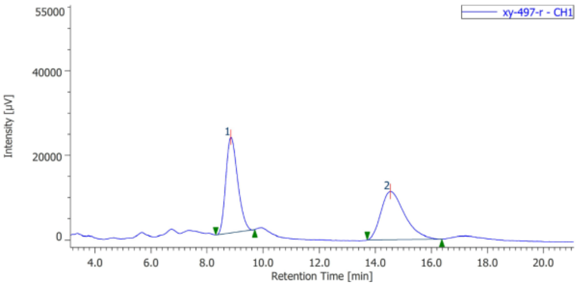


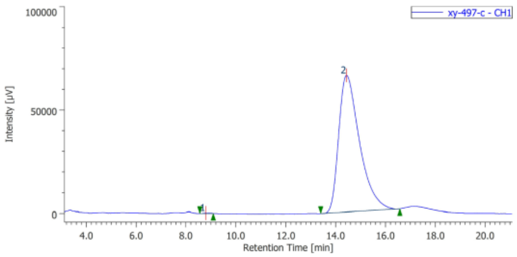


| # | Peak Name | CH | tR [min] | Area [µV·sec] | Height [µV] | Area% | Height% |
|---|---|---|---|---|---|---|---|
| 1 | Unknown | 1 | 8.850 | 669932 | 22662 | 50.255 | 66.642 |
| 2 | Unknown | 1 | 14.530 | 663141 | 11344 | 49.745 | 33.358 |
| Total | | | | 1333073 | 34006 | | |

| # | Peak Name | CH | tR [min] | Area [µV·sec] | Height [µV] | Area% | Height% |
|---|---|---|---|---|---|---|---|
| 1 | Unknown | 1 | 8.790 | 3051 | 168 | 0.080 | 0.254 |
| 2 | Unknown | 1 | 14.430 | 3832401 | 66090 | 99.920 | 99.746 |
| Total | | | | 3835452 | 66258 | | |

**(6a*R*,11b*R*)-9-Methyl-7-(pyrimidin-2-yl)-6-(quinolin-8-yl)-6,6a,7,11b-tetrahydro-5*H*-indolo[2,3-*c*]isoquinolin-5-one (31)**

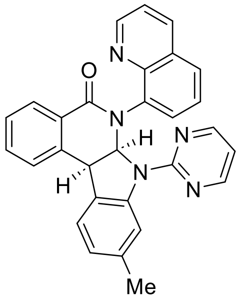


Prepared according to general procedure **A** on a 0.15 mmol scale, column chromatography (*n*-hexane/ethyl acetate = 1:2) afforded the title compound as a pale yellow sticky liquid (45 mg, 0.10 mmol, 66%), with >20:1 dr, 99% ee.

**$^1$H NMR** (400 MHz, $CDCl_3$) δ 8.95 (d, *J* = 4.1 Hz, 1H), 8.27 (d, *J* = 7.7 Hz, 1H), 8.15 (s, 1H), 8.09 (d, *J* = 8.2 Hz, 1H), 7.69 – 7.64 (m, 3H), 7.56 (t, *J* = 7.9 Hz, 2H), 7.51 – 7.47 (m, 2H), 7.39 (dd, *J* = 8.3, 4.2 Hz, 1H), 7.20 – 7.14 (d, *J* = 7.2 Hz, 2H), 6.89 (d, *J* = 7.6 Hz, 1H), 6.84 (d, *J* = 7.7 Hz, 1H), 6.21 (t, *J* = 4.8 Hz, 1H), 4.79 (d, *J* = 6.4 Hz, 1H), 2.45 (s, 3H) ppm;

**$^{13}$C NMR** (101 MHz, $CDCl_3$) δ 164.1 ($C_q$), 158.9 ($C_q$), 156.3 (CH), 150.3 (CH), 145.5 ($C_q$), 141.2 ($C_q$), 138.3 ($C_q$), 137.0 ($C_q$), 135.7 (CH), 135.1 ($C_q$), 132.4 (CH), 131.1 ($C_q$), 130.8 (CH), 129.4

(CH), 128.8 ($C_q$), 128.4 (CH), 127.9 (CH), 127.8 (CH), 127.1 ($C_q$), 125.5 (CH), 123.6 (CH), 123.5 (CH), 121.0 (CH), 117.8 (CH), 111.6 (CH), 76.5 (CH), 43.9 (CH), 21.9 ($CH_3$) ppm.

**IR** (ATR): $\tilde{\nu}$ = 3103, 1657, 1581, 1496, 1451, 1384, 1291, 794, 613, 422 $cm^{-1}$;

**HRMS (ESI):** *m/z* $[M+H]^+$ calcd for $C_{29}H_{22}N_5O$: 456.1819; found: 456.1815;

$[\alpha]^{D}_{20}$ = –190.2 (c = 0.50, $CHCl_3$);

**$R_t$** (ID-3 column, *n*-hexane/*i*-PrOH 50/50, 1.0 mL/min, 250.4 nm): tr(major) = 35.8 min, tr(minor) = 10.9 min, 99% ee.

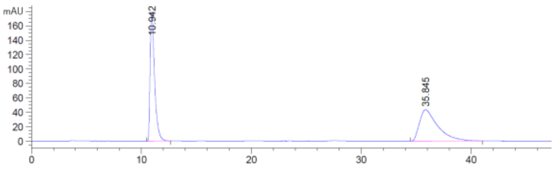


| Peak # | RetTime [min] | Type | Width [min] | Area [mAU*s] | Height [mAU] | Area % |
|---|---|---|---|---|---|---|
| 1 | 10.942 | BB | 0.4309 | 5101.60254 | 178.78563 | 50.4363 |
| 2 | 35.845 | BB | 1.3515 | 5013.33301 | 43.69608 | 49.5637 |

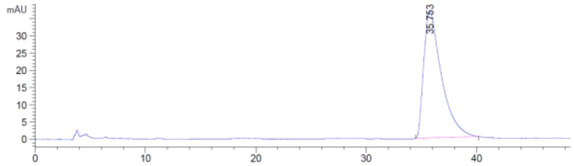


| Peak # | RetTime [min] | Type | Width [min] | Area [mAU*s] | Height [mAU] | Area % |
|---|---|---|---|---|---|---|
| 1 | 35.753 | BB | 1.3008 | 4095.33032 | 36.90054 | 100.0000 |

**(6a*R*,11b*R*)-8-Methyl-7-(pyrimidin-2-yl)-6-(quinolin-8-yl)-6,6a,7,11b-tetrahydro-5*H*-indolo[2,3-*c*]isoquinolin-5-one (32)**

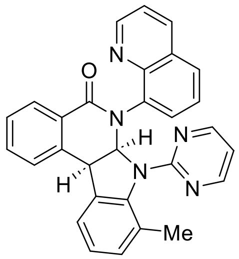


Prepared according to general procedure **A** on a 0.15 mmol scale, column chromatography (*n*-hexane/ethyl acetate = 1:2) afforded the title compound as a pale yellow sticky liquid (32 mg, 0.07 mmol, 47%), with >20:1 dr, 99% ee.

**$^1$H NMR** (400 MHz, $CDCl_3$) δ 9.04 (d, *J* = 4.3 Hz, 1H), 8.26 (d, *J* = 7.8 Hz, 1H), 8.19 (d, *J* = 8.3 Hz, 1H), 7.83 (d, *J* = 4.9 Hz, 2H), 7.76 (d, *J* = 8.2 Hz, 1H), 7.65 (t, *J* = 7.3 Hz, 1H), 7.52 (d, *J* = 7.7 Hz, 1H), 7.49 – 7.39 (m, 3H), 7.31 (dd, *J* = 12.6, 6.7 Hz, 2H), 7.17 (d, *J* = 7.6 Hz, 1H), 7.04 (t, *J* = 7.5 Hz, 1H), 6.85 (d, *J* = 7.6 Hz, 1H), 6.39 (t, *J* = 4.9 Hz, 1H), 4.94 (d, *J* = 6.1 Hz, 1H), 2.17 (s, 3H) ppm;

**$^{13}$C NMR** (101 MHz, $CDCl_3$) δ 164.1 ($C_q$), 159.4 ($C_q$), 157.0 (CH), 150.1 (CH), 145.3 ($C_q$), 140.4 ($C_q$), 136.5 ($C_q$), 136.2 (CH), 135.5 ($C_q$), 135.4 ($C_q$), 132.3 (CH), 131.2 (CH), 130.5 (CH), 129.4 (CH), 129.2 ($C_q$), 129.0 ($C_q$), 128.4 (CH), 128.2 (CH), 127.8 (CH), 127.2 ($C_q$), 125.6 (CH), 125.1 (CH), 121.5 (CH), 121.0 (CH), 112.9 (CH), 81.8 (CH), 45.7 (CH), 20.3 ($CH_3$) ppm.

**IR** (ATR): $\tilde{\nu}$ = 3388, 1657, 1579, 1459, 1421, 1289, 1167, 795, 573, 440 $cm^{-1}$;

**HRMS (ESI):** *m/z* $[M+H]^+$ calcd for $C_{29}H_{22}N_5O$: 456.1819; found: 456.1817;

$[\alpha]^D_{20}$ = –143.8 (c = 0.50, $CHCl_3$);

$R_t$ (ID-3 column, *n*-hexane/*i*-PrOH 50/50, 1.0 mL/min, 250.4 nm): tr(major) = 25.2 min, tr(minor) =12.3 min, 99% ee.

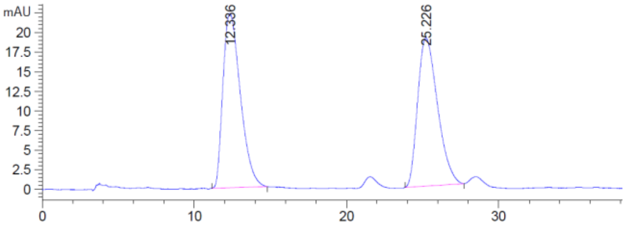


| Peak # | RetTime [min] | Type | Width [min] | Area [mAU*s] | Height [mAU] | Area % |
|---|---|---|---|---|---|---|
| 1 | 12.336 | BB | 0.9364 | 1777.88000 | 22.29314 | 50.8388 |
| 2 | 25.226 | BB | 1.0654 | 1719.21094 | 18.93324 | 49.1612 |

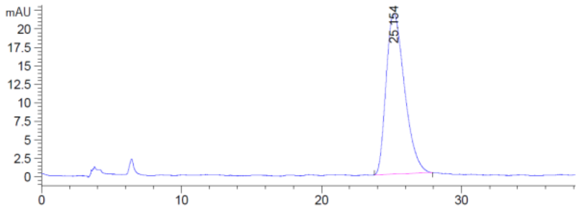


| Peak # | RetTime [min] | Type | Width [min] | Area [mAU*s] | Height [mAU] | Area % |
|---|---|---|---|---|---|---|
| 1 | 25.154 | BB | 1.0699 | 1989.31030 | 21.75242 | 100.0000 |

**(6a*R*,11b*R*)-7-(Pyridin-2-yl)-6-(quinolin-8-yl)-6,6a,7,11b-tetrahydro-5*H*-indolo[2,3-*c*]isoquinolin-5-one (33)**

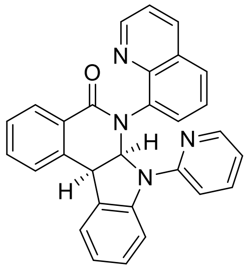

Prepared according to general procedure **A** on a 0.15 mmol scale, column chromatography (*n*-hexane/ethyl acetate = 1:2) afforded the title compound as a pale yellow sticky liquid (34 mg, 0.08 mmol, 52%), with >20:1 dr, 98% ee.

**$^1$H NMR** (400 MHz, $CDCl_3$) δ 8.97 (d, *J* = 3.8 Hz, 1H), 8.26 (d, *J* = 7.7 Hz, 1H), 8.14 (d, *J* = 8.2 Hz, 1H), 7.99 (d, *J* = 7.9 Hz, 1H), 7.74 – 7.63 (m, 3H), 7.56 (d, *J* = 7.6 Hz, 1H), 7.51 (t, *J* = 7.6 Hz, 1H), 7.43 (dd, *J* = 8.4, 4.2 Hz, 1H), 7.39 – 7.20 (m, 3H), 7.08 – 6.95 (m, 3H), 6.79 (t, *J* = 7.6 Hz, 1H), 6.36 (t, *J* = 6.1 Hz, 1H), 5.99 (d, *J* = 8.6 Hz, 1H), 4.86 (d, *J* = 6.0 Hz, 1H) ppm;

**$^{13}$C NMR** (101 MHz, $CDCl_3$) δ 164.3 ($C_q$), 154.3 ($C_q$), 150.6 (CH), 147.2 (CH), 145.3 ($C_q$), 142.5 ($C_q$), 136.6 ($C_q$), 136.3 (CH), 136.2 (CH), 135.0 ($C_q$), 133.6 ($C_q$), 132.6 (CH), 131.7 (CH), 129.5 ($C_q$), 129.1 (CH), 128.5 (CH), 128.3 (CH), 128.0 (CH), 127.2 ($C_q$), 126.2 (CH), 124.0 (CH), 122.5 (CH), 121.3 (CH), 116.5 (CH), 114.9 (CH), 107.9 (CH), 78.1 (CH), 45.3 (CH) ppm.

**IR** (ATR): $\tilde{\nu}$ = 1657, 1574, 1555, 1535, 1485, 1441, 1391, 1286, 783, 746 $cm^{-1}$;

**HRMS (ESI):** *m/z* $[M+H]^+$ calcd for $C_{29}H_{21}N_4O$: 441.1710; found: 441.1705;

**$[\alpha]^D_{20}$** = –172.4 (c = 1.00, $CHCl_3$);

**$R_t$** (ID-3 column, *n*-hexane/*i*-PrOH 50/50, 1.0 mL/min, 250.4 nm): tr(major) = 33.4 min, tr(minor) = 10.6 min, 98% ee.

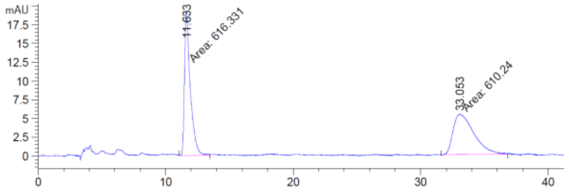


| Peak # | RetTime [min] | Type | Width [min] | Area [mAU*s] | Height [mAU] | Area % |
|---|---|---|---|---|---|---|
| 1 | 11.633 | MM | 0.5331 | 616.33130 | 19.26868 | 50.2483 |
| 2 | 33.053 | MM | 1.8837 | 610.24017 | 5.39939 | 49.7517 |

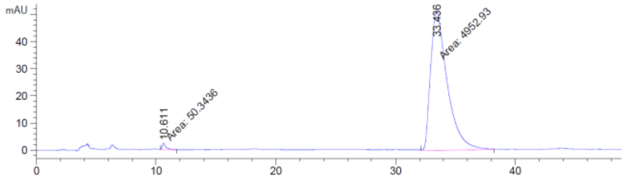


| Peak # | RetTime [min] | Type | Width [min] | Area [mAU*s] | Height [mAU] | Area % |
|---|---|---|---|---|---|---|
| 1 | 10.611 | MM | 0.3462 | 50.34361 | 2.42381 | 1.0062 |
| 2 | 33.436 | MM | 1.6111 | 4952.92822 | 51.23758 | 98.9938 |

**(6a*R*,11b*R*)-7-(5-Chloropyrimidin-2-yl)-6-(quinolin-8-yl)-6,6a,7,11b-tetrahydro-5*H*-indolo[2,3-*c*]isoquinolin-5-one (34)**

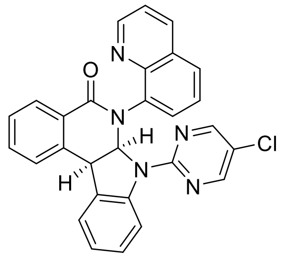

Prepared according to general procedure **A** on a 0.15 mmol scale, column chromatography (*n*-hexane/ethyl acetate = 1:2) afforded the title compound as a pale yellow sticky liquid (31 mg, 0.06 mmol, 43%), with >20:1 dr, 99% ee.

**$^1$H NMR** (400 MHz, $CDCl_3$) δ 9.07 – 8.91 (m, 1H), 8.32 (d, *J* = 7.8 Hz, 1H), 8.27 (d, *J* = 8.0 Hz, 1H), 8.19 (d, *J* = 8.2 Hz, 1H), 7.75 (t, *J* = 7.5 Hz, 1H), 7.69 (d, *J* = 8.1 Hz, 1H), 7.67 – 7.53 (m, 4H), 7.48 (d, *J* = 6.6 Hz, 2H), 7.41 (t, *J* = 7.6 Hz, 1H), 7.23 (dt, *J* = 16.2, 7.3 Hz, 2H), 7.15 – 7.03 (m, 2H), 4.88 (d, *J* = 6.4 Hz, 1H) ppm;

**$^{13}$C NMR** (101 MHz, $CDCl_3$) δ 164.1 ($C_q$), 157.0 ($C_q$), 154.6 (CH), 150.5 (CH), 145.5 ($C_q$), 140.8 ($C_q$), 136.9 ($C_q$), 135.9 (CH), 134.7 ($C_q$), 133.9 ($C_q$), 132.6 (CH), 130.9 (CH), 129.6 (CH), 128.9 ($C_q$), 128.5 (CH), 128.4 (CH), 128.2 (CH), 128.1 (CH), 127.1 ($C_q$), 125.7 (CH), 124.0 (CH), 123.4 (CH), 121.3 (CH), 120.3 ($C_q$), 117.2 (CH), 44.3 (CH) ppm.

**IR** (ATR): $\tilde{\nu}$ = 1650, 1580, 1554, 1483, 1432, 1388, 1294, 1115, 795, 750 $cm^{-1}$;

**HRMS (ESI):** $m/z$ $[M+H]^+$ calcd for $C_{28}H_{19}ClN_5O$: 476.1273; found: 476.1271;

$[\alpha]^D{}_{20}$ = –175.4 (c = 0.50, $CHCl_3$);

$R_t$ (ID-3 column, *n*-hexane/*i*-PrOH 50/50, 1.0 mL/min, 250.4 nm): tr(major) = 32.5 min, tr(minor) = 12.3 min, 99% ee.

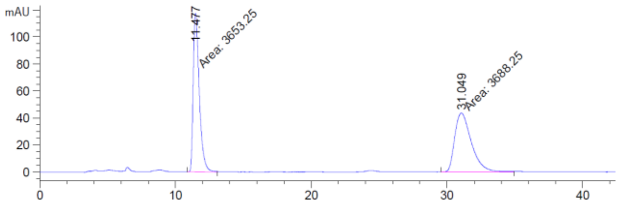


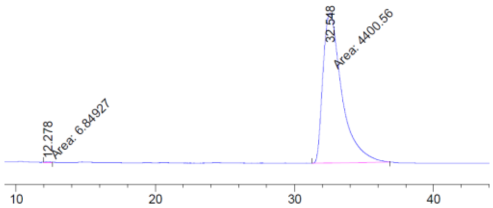


| Peak # | RetTime [min] | Type | Width [min] | Area [mAU*s] | Height [mAU] | Area % |
|---|---|---|---|---|---|---|
| 1 | 11.477 | MM | 0.5188 | 3653.24683 | 117.35151 | 49.7616 |
| 2 | 31.049 | MM | 1.4153 | 3688.25366 | 43.43379 | 50.2384 |

| Peak # | RetTime [min] | Type | Width [min] | Area [mAU*s] | Height [mAU] | Area % |
|---|---|---|---|---|---|---|
| 1 | 12.278 | MM | 0.4718 | 6.84927 | 2.41950e-1 | 0.1554 |
| 2 | 32.548 | MM | 1.5775 | 4400.56006 | 46.49390 | 99.8446 |

**(6a*R*,11b*R*)-7-(5-Methylpyrimidin-2-yl)-6-(quinolin-8-yl)-6,6a,7,11b-tetrahydro-5*H*-indolo[2,3-*c*]isoquinolin-5-one (35)**

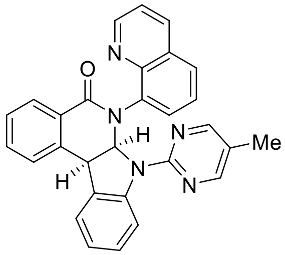


Prepared according to general procedure **A** on a 0.15 mmol scale, column chromatography (*n*-hexane/ethyl acetate = 1:2) afforded the title compound as a pale yellow sticky liquid (42 mg, 0.09 mmol, 62%), with >20:1 dr, 99% ee.

**$^1$H NMR** (400 MHz, $CDCl_3$) δ 8.96 (d, $J$ = 2.9 Hz, 1H), 8.27 (d, $J$ = 7.8 Hz, 2H), 8.11 (d, $J$ = 8.1 Hz, 1H), 7.69 (t, $J$ = 7.4 Hz, 1H), 7.57 (d, $J$ = 6.8 Hz, 2H), 7.54 – 7.46 (m, 4H), 7.44 – 7.31 (m, 2H), 7.16 (d, $J$ = 4.9 Hz, 2H), 7.01 (d, $J$ = 4.5 Hz, 2H), 4.83 (d, $J$ = 6.4 Hz, 1H), 1.88 (s, 3H) ppm;

**$^{13}$C NMR** (101 MHz, $CDCl_3$) δ 164.2 ($C_q$), 157.5 ($C_q$), 156.4 (CH), 150.4 (CH), 145.6 ($C_q$), 141.5 ($C_q$), 137.1 ($C_q$), 135.8 (CH), 135.0 ($C_q$), 133.8 ($C_q$), 132.5 (CH), 130.9 (CH), 129.6 (CH), 128.9 ($C_q$), 128.5 (CH), 128.4 (CH), 127.9 (CH), 127.8 (CH), 127.3 ($C_q$), 125.6 (CH), 123.8 (CH), 122.7 (CH), 121.1 (CH), 120.3 ($C_q$), 116.9 (CH), 76.5 (CH), 44.4 (CH), 14.6 (CH) ppm.

**IR** (ATR): $\tilde{\nu}$ = 3388, 1655, 1579, 1482, 1433, 1289, 1167, 795, 749 $cm^{-1}$;

**HRMS (ESI):** $m/z$ $[M+H]^+$ calcd for $C_{29}H_{22}N_5O$: 456.1819; found: 456.1817;

**[α]$^{D}_{20}$** = –170.8 (c = 0.50, $CHCl_3$);

**R$_t$** (ID-3 column, *n*-hexane/*i*-PrOH 50/50, 1.0 mL/min, 250.4 nm): tr(major) = 42.6 min, tr(minor) = 13.9 min, 99% ee.

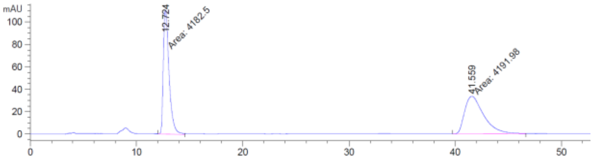


| Peak # | RetTime [min] | Type | Width [min] | Area [mAU*s] | Height [mAU] | Area % |
|---|---|---|---|---|---|---|
| 1 | 12.724 | MM | 0.6281 | 4182.50488 | 110.99054 | 49.9435 |
| 2 | 41.559 | MM | 2.0830 | 4191.97559 | 33.54043 | 50.0565 |

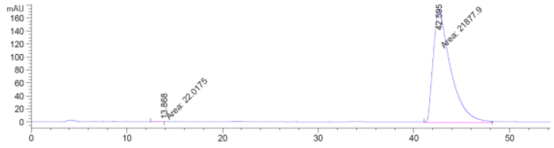


| Peak # | RetTime [min] | Type | Width [min] | Area [mAU*s] | Height [mAU] | Area % |
|---|---|---|---|---|---|---|
| 1 | 13.868 | MM | 1.2919 | 22.01746 | 2.84041e-1 | 0.1005 |
| 2 | 42.595 | MM | 2.1002 | 2.18779e4 | 173.62083 | 99.8995 |

**(3a*S*,9b*S*)-4-(Quinolin-8-yl)-1,2,3,3a,4,9b-hexahydro-5H-cyclopenta[*c*]isoquinolin-5-one (36)**

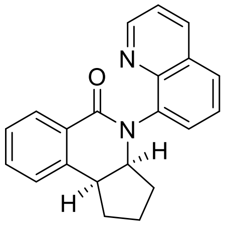


Prepared according to general procedure **C** for 16h on a 0.30 mmol scale, column chromatography (*n*-hexane/ethyl acetate = 1:1) afforded the title compound as a pale yellow sticky liquid (78 mg, 0.22 mmol, 82%), with >20:1 dr, 99% ee.

**$^{1}$H NMR** (400 MHz, $CDCl_3$) δ 8.88 (s, 1H), 8.24 – 8.14 (m, 2H), 7.83 (d, *J* = 7.9 Hz, 1H), 7.72 – 7.55 (m, 2H), 7.49 (td, *J* = 7.5, 1.4 Hz, 1H), 7.42 – 7.27 (m, 3H), 4.96 (s, 1H), 3.49 (q, *J* = 8.0 Hz, 1H), 2.45 – 2.01 (m, 2H), 1.96 – 1.52 (m, 4H) ppm;

**$^{13}$C NMR** (101 MHz, $CDCl_3$) δ 164.9 ($C_q$), 150.5 (CH), 144.5 ($C_q$), 141.4 ($C_q$), 138.2 ($C_q$), 136.4 (CH), 132.4 (CH), 132.1 (CH), 130.7 (CH), 129.8 ($C_q$), 129.1 (CH), 128.0 (CH), 127.7 ($C_q$), 127.0 (CH), 126.7 (CH), 126.3 (CH), 121.5 (CH), 63.1 (CH), 42.4 (CH), 33.0 ($CH_2$), 32.4 ($CH_2$), 22.2 ($CH_2$) ppm.

**IR** (ATR): $\tilde{\nu}$ = 2960, 1650, 1601, 1461, 1392, 1269, 829, 793, 754, 697 cm$^{-1}$;

**HRMS (ESI):** *m/z* $[M+H]^+$ calcd for $C_{21}H_{19}N_2O$: 315.1492; found: 315.1492;

**m.p.:** 63-64 °C;

**[α]$^{D}_{20}$** = –13.1 (c = 0.50, $CHCl_3$);

**R$_t$** (ID-3 column, *n*-hexane/*i*-PrOH 50/50, 1.0 mL/min, 250.4 nm): tr(major) = 16.0 min, tr(minor) = 13.5 min, 99% ee.

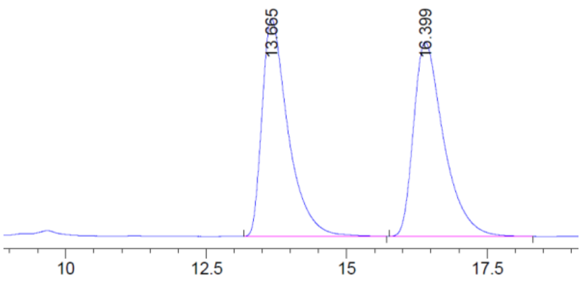


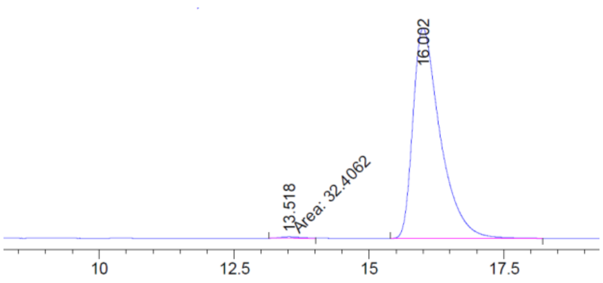


| Peak # | RetTime [min] | Type | Width [min] | Area [mAU*s] | Height [mAU] | Area % |
|---|---|---|---|---|---|---|
| 1 | 13.665 | BB | 0.4707 | 6371.22559 | 197.33699 | 49.1730 |
| 2 | 16.399 | BB | 0.5439 | 6585.54053 | 176.26939 | 50.8270 |

| Peak # | RetTime [min] | Type | Width [min] | Area [mAU*s] | Height [mAU] | Area % |
|---|---|---|---|---|---|---|
| 1 | 13.518 | MM | 0.4455 | 32.40618 | 1.21230 | 0.5234 |
| 2 | 16.002 | BB | 0.5278 | 6159.12451 | 174.47806 | 99.4766 |

**(*S*)-3-Phenyl-2-(quinolin-8-yl)-3,4-dihydroisoquinolin-1(2*H*)-one (37)**

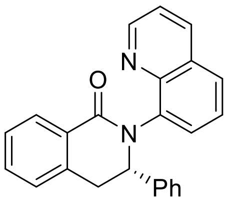


Prepared according to general procedure **C** for 36h on a 0.30 mmol scale, column chromatography (*n*-hexane/ethyl acetate = 1:1) afforded the title compound as a pale yellow sticky liquid (74 mg, 0.20 mmol, 70%), with >20:1 rr, 90% ee.

**$^1$H NMR** (400 MHz, $CDCl_3$) δ 8.94 (dd, *J* = 4.2, 1.8 Hz, 1H), 8.23 (d, *J* = 7.6 Hz, 1H), 8.14 (d, *J* = 8.2 Hz, 1H), 7.72 (d, *J* = 7.6 Hz, 1H), 7.57 – 7.49 (m, 1H), 7.46 – 7.35 (m, 4H), 7.24 – 7.08 (m, 6H), 5.45 (dd, *J* = 6.2, 4.3 Hz, 1H), 4.21 (s, 1H), 3.23 (d, *J* = 13.9 Hz, 1H) ppm;

$^{13}$C NMR (101 MHz, $CDCl_3$) δ 165.2 ($C_q$), 150.5 (CH), 144.4 ($C_q$), 140.9 ($C_q$), 139.2 ($C_q$), 136.8 ($C_q$), 136.3 (CH), 132.2 (CH), 130.2 (CH), 129.9 ($C_q$), 129.6 ($C_q$), 128.5 (CH), 128.3 (CH), 127.8 (CH), 127.6 (CH), 127.4 (CH), 127.1 (CH), 127.0 (CH), 126.1 (CH), 121.4 (CH), 63.2 (CH), 36.4 ($CH_2$) ppm.

**IR** (ATR): $\tilde{\nu}$ = 3033, 1652, 1495, 1472, 1417, 1331, 1252, 795, 754, 700 $cm^{-1}$;

**HRMS (ESI):** *m/z* $[M+H]^+$ calcd for $C_{24}H_{19}N_2O$: 351.1492; found: 351.1493;

**$[\alpha]^D_{20}$** = –208.4 (c = 1.00, $CHCl_3$);

**$R_t$** (IB-3 column, *n*-hexane/*i*-PrOH 80/20, 1.0 mL/min, 250.4 nm): tr(major) = 15.0 min, tr(minor) = 13.6 min, 90% ee.

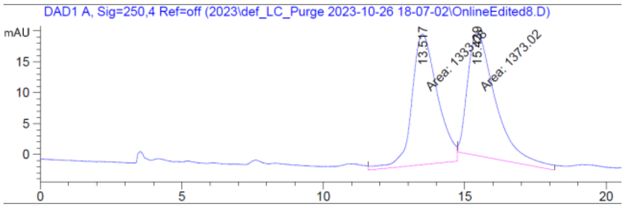


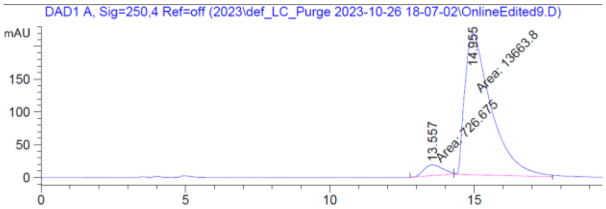


| Peak # | RetTime [min] | Type | Width [min] | Area [mAU*s] | Height [mAU] | Area % |
|---|---|---|---|---|---|---|
| 1 | 13.517 | MM | 1.0509 | 1333.08118 | 21.14235 | 49.2620 |
| 2 | 15.439 | MM | 1.1510 | 1373.02344 | 19.88186 | 50.7380 |

| Peak # | RetTime [min] | Type | Width [min] | Area [mAU*s] | Height [mAU] | Area % |
|---|---|---|---|---|---|---|
| 1 | 13.557 | MM | 0.7525 | 726.67487 | 16.09569 | 5.0497 |
| 2 | 14.955 | MM | 1.0566 | 1.36638e4 | 215.52831 | 94.9503 |

**(*R*)-Ethyl 2-(2-(7-methylquinolin-8-yl)-1-oxo-1,2-dihydroisoquinolin-3-yl)acetate (38)**

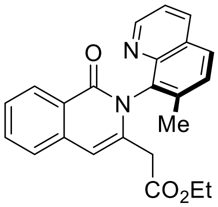


Prepared according to general procedure **C** for 48h on a 0.30 mmol scale, column chromatography (*n*-hexane/ethyl acetate = 1:1) afforded the title compound as a pale yellow sticky liquid (68 mg, 0.17 mmol, 61%), with 99% ee.

**$^{1}$H NMR** (400 MHz, $CDCl_3$) δ 8.86 (dd, *J* = 4.2, 1.7 Hz, 1H), 8.49 – 8.41 (m, 1H), 8.22 (dd, *J* = 8.3, 1.7 Hz, 1H), 7.91 (d, *J* = 8.4 Hz, 1H), 7.76 – 7.68 (m, 1H), 7.62 (d, *J* = 7.7 Hz, 1H), 7.58 (d, *J* = 8.5 Hz, 1H), 7.56 – 7.48 (m, 1H), 7.41 (dd, *J* = 8.3, 4.2 Hz, 1H), 6.72 (s, 1H), 3.87 (qd, *J* = 7.1, 2.9 Hz, 2H), 3.38 – 3.25 (m, 1H), 3.14 (dd, *J* = 16.3, 0.7 Hz, 1H), 2.40 (s, 3H), 1.06 (t, *J* = 7.1 Hz, 3H) ppm;

**$^{13}$C NMR** (101 MHz, $CDCl_3$) δ 168.86 ($C_q$), 162.59 ($C_q$), 151.31 (CH), 144.78 ($C_q$), 139.51 ($C_q$), 137.24 ($C_q$), 136.76 ($C_q$), 136.05 (CH), 133.71 ($C_q$), 132.61 (CH), 129.44 (CH), 128.75 (CH), 128.39 (CH), 127.68 ($C_q$), 126.62 (CH), 125.98 (CH), 125.51 ($C_q$), 121.05 (CH), 108.01 (CH), 61.02 (CH), 39.82 (CH), 18.52 ($CH_3$), 13.91 ($CH_3$) ppm.

**IR** (ATR): $\tilde{\nu}$ = 2965, 1734, 1658, 1627, 1400, 1300, 1160, 838, 757, 694 $cm^{-1}$;

**HRMS (ESI):** *m/z* $[M+H]^+$ calcd for $C_{23}H_{21}N_2O_3$: 373.1547; found: 373.1545;

**$[\alpha]^{D}_{20}$** = –6.8 (c = 1.00, $CHCl_3$);

**$R_t$** (OD-3 column, *n*-hexane/*i*-PrOH 70/30, 1.0 mL/min, 250.4 nm): tr(major) = 9.8 min, tr(minor) = 16.6 min, 99% ee.

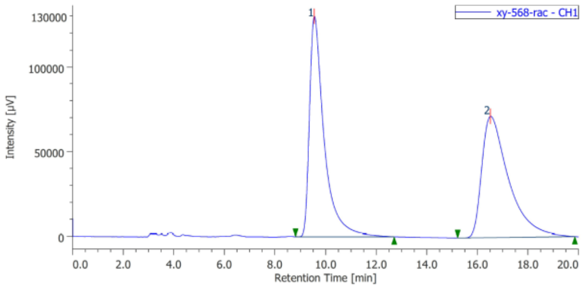


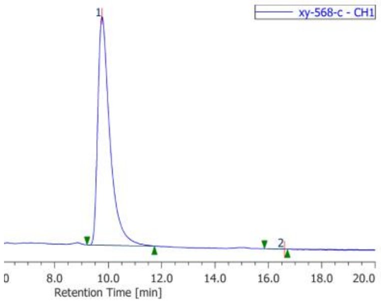


| # | Peak Name | CH | tR [min] | Area [μV·sec] | Height [μV] | Area% | Height% |
|---|---|---|---|---|---|---|---|
| 1 | Unknown | 1 | 9.547 | 5227144 | 129916 | 49.869 | 64.530 |
| 2 | Unknown | 1 | 16.513 | 5254559 | 71409 | 50.131 | 35.470 |
| Total | | | | 10481703 | 201325 | | |

| # | Peak Name | CH | tR [min] | Area [μV·sec] | Height [μV] | Area% | Height% |
|---|---|---|---|---|---|---|---|
| 1 | Unknown | 1 | 9.760 | 1814055 | 57841 | 99.992 | 99.934 |
| 2 | Unknown | 1 | 16.600 | 152 | 38 | 0.008 | 0.066 |
| Total | | | | 1814207 | 57879 | | |

**(*R*)-2-(7-Methylquinolin-8-yl)-3,4-diphenylisoquinolin-1(2*H*)-one (39)**

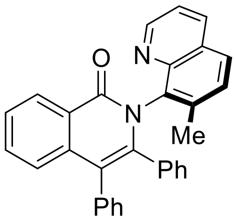


Prepared according to general procedure **B** for 48h on a 0.30 mmol scale, column chromatography (*n*-hexane/ethyl acetate = 1:1) afforded the title compound as a pale yellow sticky liquid (99 mg, 0.23 mmol, 75%), with 99% ee.

**$^{1}$H NMR** (400 MHz, $CDCl_3$) δ 8.90 (s, 1H), 8.60 (d, $J$ = 7.9 Hz, 1H), 7.99 (d, $J$ = 8.1 Hz, 1H), 7.70 – 7.47 (m, 3H), 7.44 – 7.15 (m, 8H), 6.96 (d, $J$ = 7.7 Hz, 1H), 6.86 – 6.65 (m, 3H), 6.46 (t, $J$ = 7.5 Hz, 1H), 2.39 (s, 3H) ppm;

**$^{13}$C NMR** (101 MHz, $CDCl_3$) δ 162.0 ($C_q$), 150.6 ($C_q$), 145.1 ($C_q$), 142.0 ($C_q$), 138.3 ($C_q$), 137.6 ($C_q$), 136.7 ($C_q$), 135.8 ($C_q$), 135.7 (CH), 134.6 ($C_q$), 132.4 (CH), 131.9 (CH), 131.8 (CH), 129.9 (CH), 129.1 (CH), 128.9 (CH), 128.5 (CH), 128.1 (CH), 127.9 (CH), 127.8 (CH), 127.4 (CH), 127.1 ($C_q$), 126.7 (CH), 126.6 (CH), 126.4 (CH), 126.2 (CH), 125.7 ($C_q$), 125.7 (CH), 120.7 (CH), 118.9 ($C_q$), 19.0 ($CH_3$) ppm.

**IR** (ATR): $\tilde{\nu}$ = 1657, 1609, 1554, 1442, 1366, 1325, 832, 756, 702 $cm^{-1}$;

**HRMS (ESI):** *m/z* $[M+H]^+$ calcd for $C_{31}H_{23}N_2O$: 439.1805; found: 439.1807;

**$[\alpha]^{D}_{20}$** = +88.3 (c = 1.00, $CHCl_3$);

**$R_t$** (AD-3 column, *n*-hexane/*i*-PrOH 80/20, 1.0 mL/min, 250.4 nm): tr(major) = 7.2 min, tr(minor) = 10.3 min, 99% ee.

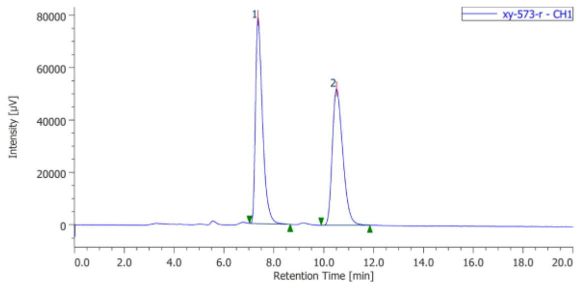


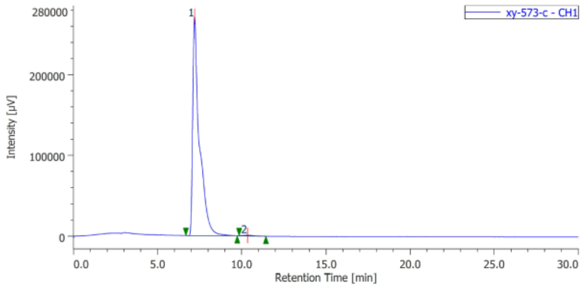


| # | Peak Name | CH | tR [min] | Area [µV·sec] | Height [µV] | Area% | Height% |
|---|---|---|---|---|---|---|---|
| 1 | Unknown | 1 | 7.367 | 1552596 | 78538 | 50.397 | 60.101 |
| 2 | Unknown | 1 | 10.503 | 1528133 | 52140 | 49.603 | 39.899 |
| Total | | | | 3080729 | 130678 | | |

| # | Peak Name | CH | tR [min] | Area [µV·sec] | Height [µV] | Area% | Height% |
|---|---|---|---|---|---|---|---|
| 1 | Unknown | 1 | 7.200 | 7526448 | 271327 | 99.577 | 99.676 |
| 2 | Unknown | 1 | 10.347 | 31953 | 882 | 0.423 | 0.324 |
| Total | | | | 7558401 | 272209 | | |

**(*R*)-4-(7-Methylquinolin-8-yl)-3,3-di-*p*-tolylfuro[3,4-*c*]isoquinoline-1,5(3*H*,4*H*)-dione (40)**

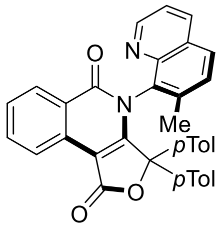


Prepared according to general procedure **C** for 48h on a 0.30 mmol scale, column chromatography (*n*-hexane/ethyl acetate = 1:1) afforded the title compound as a pale yellow sticky liquid (93 mg, 0.23 mmol, 59%), with 99% ee.

**$^1$H NMR** (400 MHz, $CDCl_3$) δ 8.91 (d, *J* = 8.0 Hz, 1H), 8.48 (dd, *J* = 8.2, 1.4 Hz, 1H), 8.43 (dd, *J* = 4.3, 1.7 Hz, 1H), 7.98 – 7.86 (m, 2H), 7.75 – 7.62 (m, 2H), 7.20 (d, *J* = 8.4 Hz, 1H), 7.15 (dd, *J* = 8.2, 4.2 Hz, 1H), 7.07 (d, *J* = 8.1 Hz, 2H), 6.92 (d, *J* = 8.4 Hz, 2H), 6.84 (d, *J* = 8.3 Hz, 2H), 6.55 (d, *J* = 8.0 Hz, 2H), 2.40 (s, 3H), 2.10 (s, 3H), 1.83 (s, 3H) ppm;

**$^{13}$C NMR** (101 MHz, $CDCl_3$) δ 168.2 ($C_q$), 163.6 ($C_q$), 160.9 ($C_q$), 150.5 (CH), 144.8 ($C_q$), 140.1 ($C_q$), 139.3 ($C_q$), 137.9 ($C_q$), 134.9 ($C_q$), 134.1 (CH), 133.8 ($C_q$), 131.8 ($C_q$), 131.6 ($C_q$), 131.4 ($C_q$), 129.3 (CH), 129.2 (CH), 129.2 (CH), 129.0 (CH), 128.6 (CH), 128.4 (CH), 127.5 (CH), 127.5 (CH), 125.1 ($C_q$), 123.8 (CH), 120.4 (CH), 103.4 ($C_q$), 90.2 ($C_q$), 21.2 ($CH_3$), 20.8 ($CH_3$), 18.8 ($CH_3$) ppm.

**IR** (ATR): $\tilde{\nu}$ = 1754, 1685, 1628, 1500, 1321, 1070, 965, 783, 756 $cm^{-1}$;

**HRMS (ESI):** *m/z* $[M+H]^+$ calcd for $C_{35}H_{27}N_2O$: 523.2016; found: 523.2022;

**$[\alpha]^D_{20}$** = –15.2 (c = 1.00, $CHCl_3$);

**$R_t$** (IA-3 column, *n*-hexane/*i*-PrOH 50/50, 1.0 mL/min, 250.4 nm): tr(major) = 20.9 min, tr(minor) = 14.5 min, 99% ee.

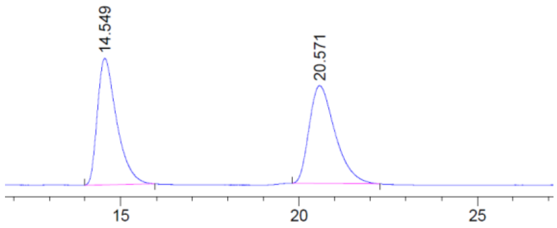


| Peak # | RetTime [min] | Type | Width [min] | Area [mAU*s] | Height [mAU] | Area % |
|---|---|---|---|---|---|---|
| 1 | 14.549 | BB | 0.5189 | 2051.13184 | 54.40099 | 49.0431 |
| 2 | 20.571 | BB | 0.5980 | 2131.17505 | 42.07385 | 50.9569 |

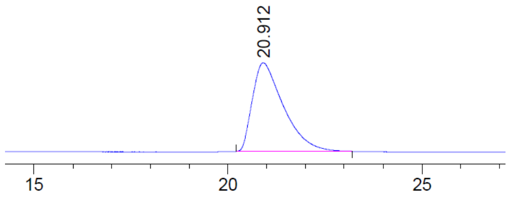


| Peak # | RetTime [min] | Type | Width [min] | Area [mAU*s] | Height [mAU] | Area % |
|---|---|---|---|---|---|---|
| 1 | 20.912 | BB | 0.6569 | 5219.05664 | 93.69074 | 100.0000 |

**(*S*)-3-butyl-2-(quinolin-8-yl)-3,4-dihydroisoquinolin-1(2*H*)-one (42)**

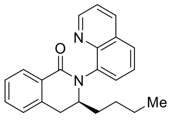
42

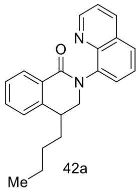
42a

Prepared according to general procedure **C** for 24h on a 0.30 mmol scale, column chromatography (*n*-hexane/ethyl acetate = 1:1) afforded the title compound **42** and **42a** as a pale yellow sticky liquid (91 mg, 0.28 mmol, 92%), with 70% ee (**42**).

**$^{1}$H NMR** (300 MHz, $CDCl_3$) δ 8.87 – 8.71 (m, 2H), 8.17 – 7.99 (m, 4H), 7.80 – 7.59 (m, 4H), 7.58 – 7.47 (m, 2H), 7.46 – 7.23 (m, 6H), 7.25 – 7.15 (m, 2H), 4.69 – 3.98 (m, 2H), 3.95 – 3.48 (m, 2H), 3.07 – 2.82 (m, 2H), 1.93 – 1.64 (m, 2H), 1.64 – 1.47 (m, 2H), 1.41 – 1.21 (m, 4H), 1.13 – 0.90 (m, 4H), 0.83 (t, *J* = 6.9 Hz, 3H), 0.65 (t, *J* = 6.9 Hz, 3H) ppm;

**$^{13}$C NMR** (75 MHz, $CDCl_3$) δ 164.8 ($C_q$), 164.4 ($C_q$), 150.5 (CH), 150.4 (CH), 144.4 ($C_q$), 143.4 ($C_q$), 141.0 ($C_q$), 137.6 ($C_q$), 136.3 (CH), 136.2 (CH), 131.9 (CH), 131.8 (CH), 129.9 ($C_q$), 129.7 ($C_q$), 129.6 ($C_q$), 129.0 ($C_q$), 128.9 (CH), 128.8 ($C_q$), 128.5 (CH), 127.8 (CH), 127.7 (CH), 126.9 (CH), 126.8 (CH), 126.7 (CH), 126.5 (CH), 126.1 (CH), 121.5 (CH), 121.4 (CH), 60.4 (CH), 54.1 (CH), 38.5 ($CH_2$), 33.4 ($CH_2$), 32.1 ($CH_2$), 31.8 ($CH_2$), 29.6 ($CH_2$), 28.6 ($CH_2$), 22.8 ($CH_2$), 22.5 ($CH_2$), 14.0 ($CH_3$), 13.9 ($CH_3$) ppm;.

**$R_t$** (OD-3 column, *n*-hexane/*i*-PrOH 50/20, 1.0 mL/min, 273.0 nm): tr(major) = 11.8 min, tr(minor) = 9.1 min, 70% ee.

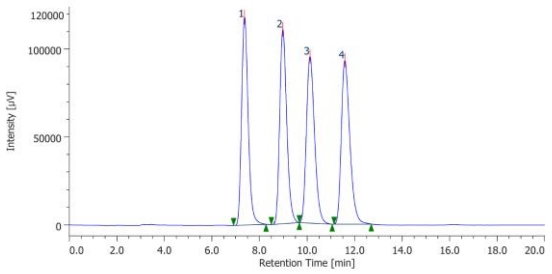


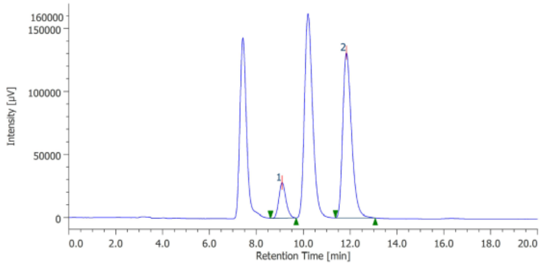


| # | Peak Name | CH | tR [min] | Area [μV·sec] | Height [μV] | Area% |
|---|---|---|---|---|---|---|
| 1 | Unknown | 10 | 7.373 | 2301141 | 118224 | 25.145 |
| 2 | Unknown | 10 | 8.970 | 2269799 | 110475 | 24.802 |
| 3 | Unknown | 10 | 10.113 | 2253670 | 94745 | 24.626 |
| 4 | Unknown | 10 | 11.580 | 2326911 | 92983 | 25.426 |

# Mechanistic Studies

## 6.1 Investigation of the Kinetic Isotope Effect (KIE)

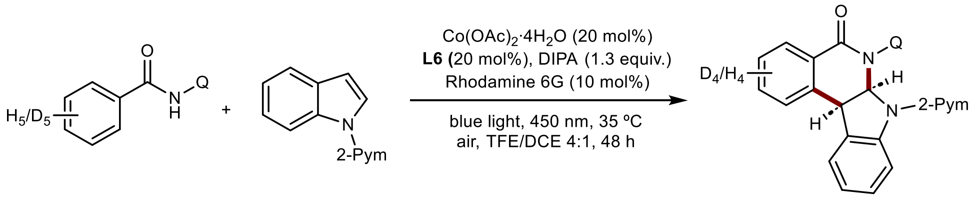


**1a/[D$_5$]-1a** **2a** **3/[D$_4$]-3**

**Parallel Reaction:** Two parallel reactions of **1a** and **1a-D** with **2a** were performed to determine the KIE by comparison of the initial reaction rates through $^1$H-NMR-analysis with 1,3,5-Trimethoxybenzene as the the internal standard. A suspension of **1a** (37.2 mg, 0.15 mmol) or **1a-D** (37.2 mg, 0.15 mmol), **2a** (29.3 mg, 0.15 mmol), $Co(OAc)_2·4H_2O$ (7.5 mg, 20 mol%), **L6** (10.6 mg, 20 mol%), Rhodamine 6G (7.2 mg, 10 mol%), diisopropylamine (27 µL, 0.20 mmol, 1.3 equiv.) and a teflon-coated magnetic stirring bar in DCE (0.25 mL) and TFE (1 mL) was stirred at room temperature under blue LEDs (450 nm). Aliquots (25 µL) were periodically removed to provide the following conversions as determined by $^1$H-NMR. No significant kinetic isotope effect ($k_H/k_D \approx 1.2$) was observed in the analysis.

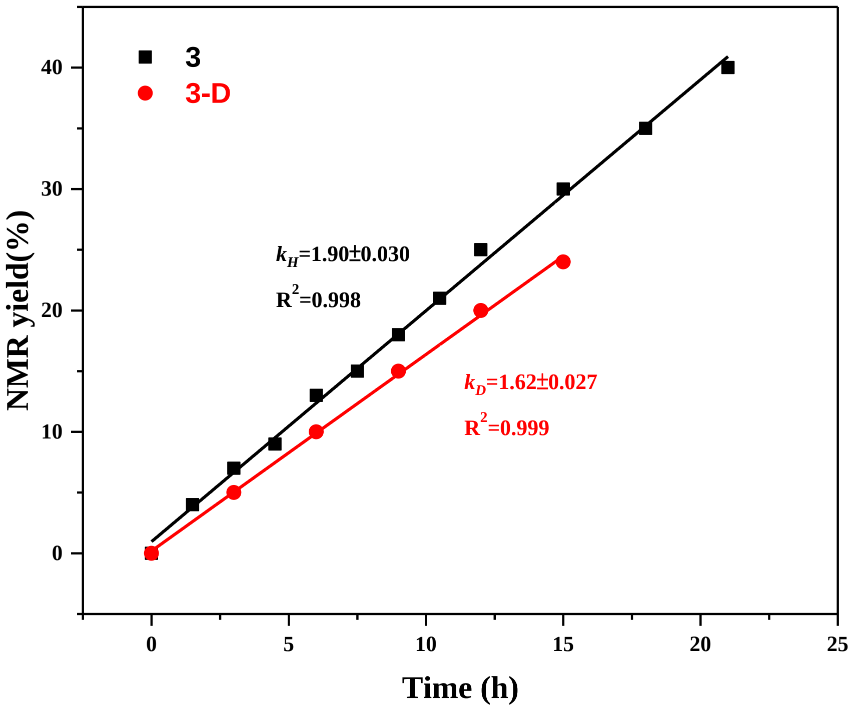


**Figure S3.** Pictures of the Kinetic Isotope Effect (KIE).

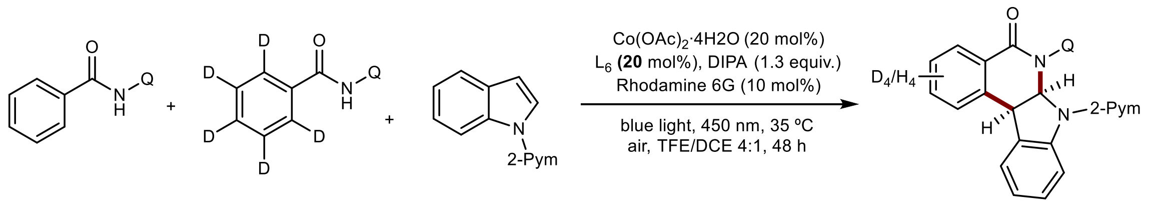


**Intermolecular Competition Kinetic Isotope Effect:** To a 10 mL vial equipped with a magnetic stir-bar were added benzamide **1a** (18.6 mg, 0.075 mmol), **1a-D5** (19.0 mg, 0.075 mmol), **2a** (29.3 mg, 0.15 mmol), $Co(OAc)_2·4H_2O$ (7.5 mg, 20 mol%), **L6** (10.6 mg, 20 mol%), Rhodamine 6G (7.2 mg, 10 mol%), diisopropylamine (27 μL, 0.20 mmol, 1.3 equiv.). Then, DCE (0.25 mL) and TFE (1 mL) were added. After the reaction mixture was stirred for 10 min, the reaction mixture was then irradiated with blue LEDs (450 nm) at ambient temperature for 8 h. The reaction mixture was concentrated in vacuo. The resulting residue was purified by silica gel flash chromatography to give the mixture products **3** and **3-D4**. A mixture of **3** and **3-D4** was determined on the basis of $^1$H

NMR analysis ($CDCl_3$). Based on the integrations related to different hydrogen resonances, the kinetic isotope effect is calculated to be $k_H/k_D \approx 1.2$.

### 6.2 The Radical Trapping Experiments

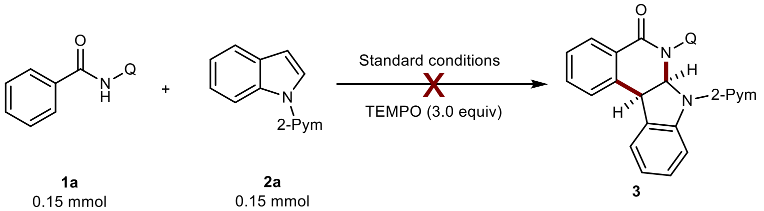


A 10 mL vial was charged with the amide **1a** (0.15 mmol, 1.0 equiv.), indole **2a** (0.15 mmol, 1.0 equiv.), $Co(OAc)_2 \cdot 4H_2O$ (7.5 mg, 20 mol%), **L6** (10.6 mg, 20 mol%), Rhodamine 6G (7.2 mg, 10 mol%), DIPA (27 µL, 0.20 mmol, 1.3 equiv.), TEMPO (35.2 mg, 0.225 mmol, 1.5 equiv.) and a teflon-coated magnetic stirring bar. Then DCE (0.25 mL) and TFE (1 mL) were added. The vial was stirred at room temperature under blue LEDs (450 nm) for 48 h. The reaction was monitored by TLC and no desired product **3** was observed.

### 6.3 Rate Studies

The kinetic profile of the reaction was determined using the variable time normalization analysis (VTNA) method described by Burés[6]. Rates were monitored using $^1$H-NMR analysis with 1,3,5-trimethoxybenzene as a standard. 1,3,5-trimethoxybenzene was determined to have no effect on the reaction.

**Determination of the reaction order of benzamide 1a**

Representative procedure: A 10 mL vial was charged with the benzamide **1a** (0.075 mmol)/(0.15 mmol)/(0.2 mmol), indole **2a** (0.15 mmol), $Co(OAc)_2 \cdot 4H_2O$ (7.5 mg, 20 mol%), **L6** (10.6 mg, 20 mol%), Rhodamine 6G (7.2 mg, 10 mol%), diisopropylamine (27 µL, 0.20 mmol, 1.3 equiv.), 1,3,5-trimethoxybenzene (16.8 mg, 0.1 mmol) and a teflon-coated magnetic stirring bar. Then DCE (0.25 mL) and TFE (1 mL) were added. The vial was stirred at room temperature under blue LEDs (450 nm). Aliquots (25 µL) were removed every 1.5 hours to provide the following conversions as determined by $^1$H-NMR. By visual analysis, it is concluded that the reaction rate is **negative order** with respect to benzamide **1a**.

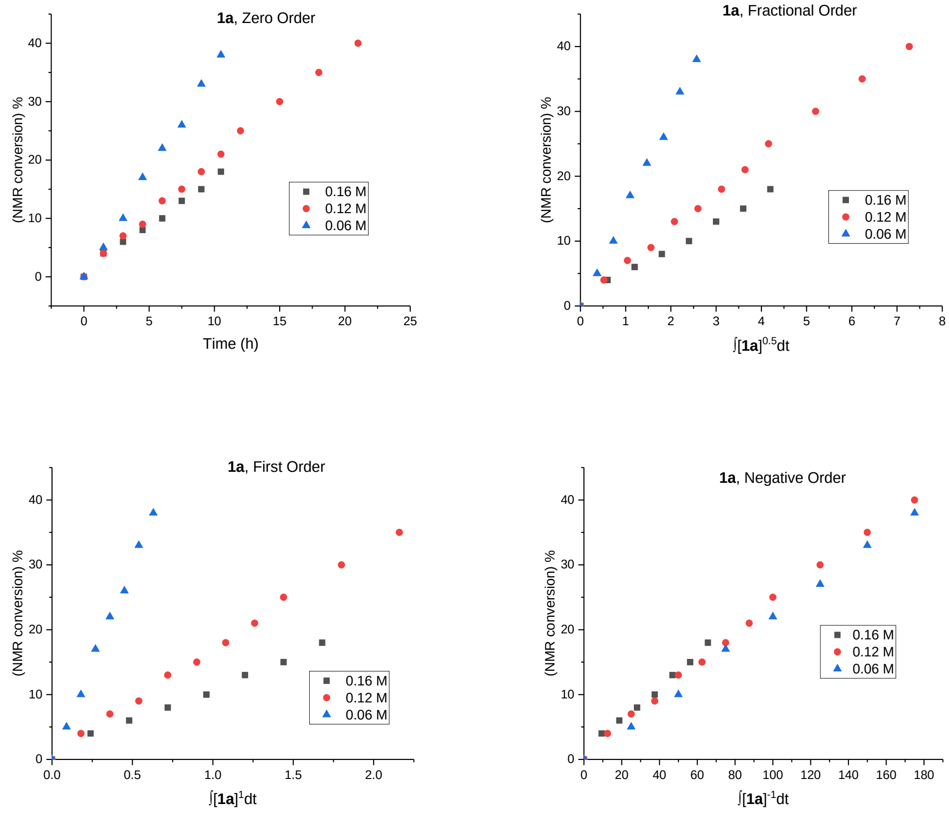


**Figure S4.** VTNA graphs for benzamide **1a**.

Representative procedure: A 10 mL vial was charged with the benzamide **1a** (0.15 mmol), indole **2a** (0.30 mmol)/(0.15 mmol)/(0.075 mmol), $Co(OAc)_2 \cdot 4H_2O$ (7.5 mg, 20 mol%), **L6** (10.6 mg, 20 mol%), Rhodamine 6G (7.2 mg, 10 mol%), diisopropylamine (27 µL, 0.20 mmol, 1.3 equiv.), 1,3,5-trimethoxybenzene (16.8 mg, 0.1 mmol) and a teflon-coated magnetic stirring bar. Then DCE (0.25 mL) and TFE (1 mL) were added. The vial was stirred at room temperature under blue LEDs (450 nm). Aliquots (25 µL) were removed every 1.5 hours to provide the following conversions as determined by $^1$H-NMR. By visual analysis, it is concluded that the reaction rate is **first order** with respect to indole **2a**.

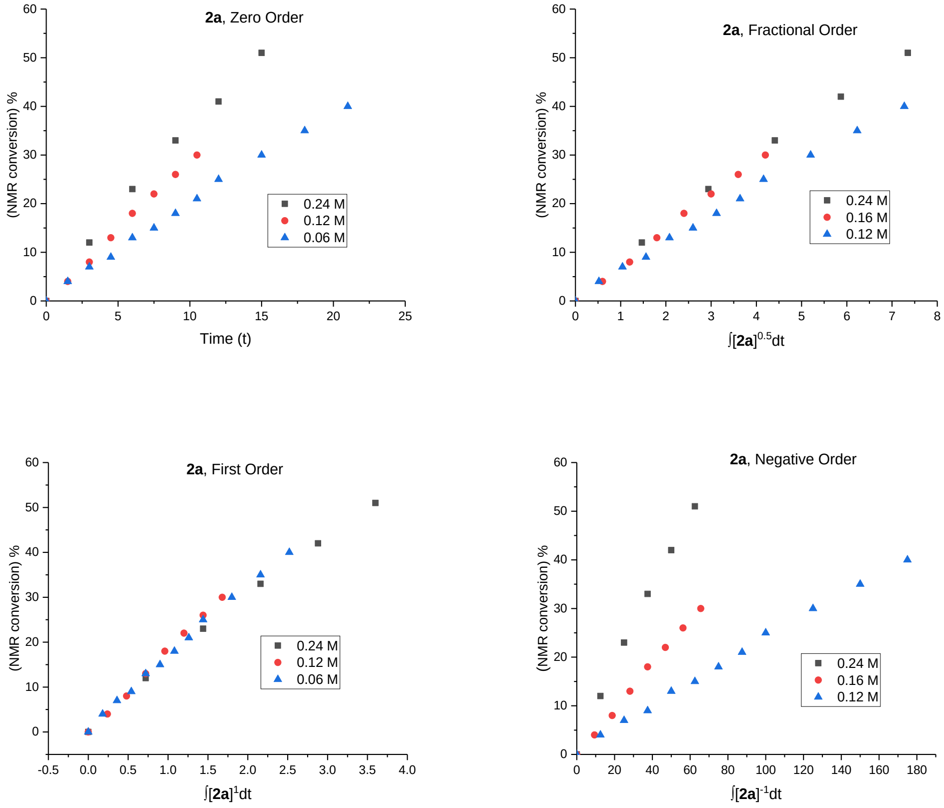


**Figure S5.** VTNA graphs for indole **2a**.

Representative procedure: A 10 mL vial was charged with the benzamide **1a** (0.15 mmol), indole **2a** (0.15 mmol), $Co(OAc)_2{\cdot}4H_2O$ (10 mol%)/(20 mol%)/(40 mol%), **L6** (10.6 mg, 20 mol%), Rhodamine 6G (7.2 mg, 10 mol%), diisopropylamine (27 μL, 0.20 mmol, 1.3 equiv.), 1,3,5-trimethoxybenzene (16.8 mg, 0.1 mmol) and a teflon-coated magnetic stirring bar. Then DCE (0.25 mL) and TFE (1 mL) were added. The vial was stirred at room temperature under blue LEDs (450 nm). Aliquots (25 μL) were removed every 1.5 hours to provide the following conversions as determined by $^{1}$H-NMR. By visual analysis, it is concluded that the reaction rate is **fractional order** with respect to $Co(OAc)_2{\cdot}4H_2O$.

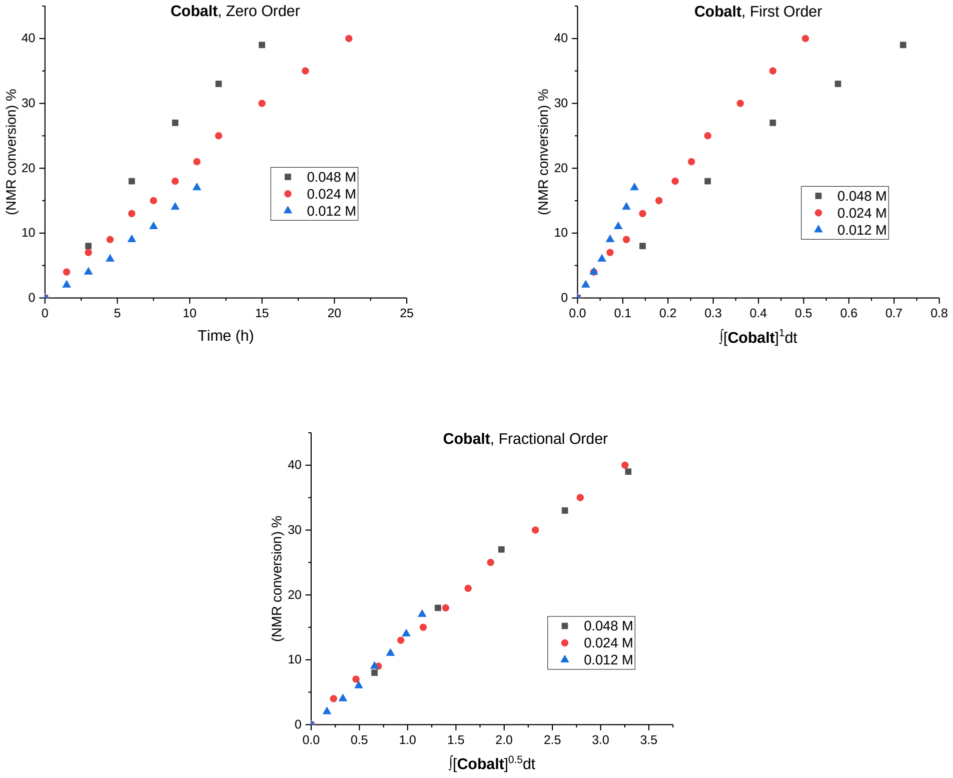


**Figure S6.** VTNA graphs for $Co(OAc)_2{\cdot}4H_2O$.

Representative procedure: A 10 mL vial was charged with the benzamide **1a** (0.15 mmol), indole **2a** (0.15 mmol), $Co(OAc)_2{\cdot}4H_2O$ (7.5 mg, 20 mol%), **L6** (10 mol%)/(20 mol%)/(40 mol%), Rhodamine 6G (7.2 mg, 10 mol%), diisopropylamine (27 μL, 0.20 mmol, 1.3 equiv.), 1,3,5-trimethoxybenzene (16.8 mg, 0.1 mmol) and a teflon-coated magnetic stirring bar. Then DCE (0.25 mL) and TFE (1 mL) were added. The vial was stirred at room temperature under blue LEDs (450 nm). Aliquots (25 μL) were removed every 1.5 hours to provide the following conversions as determined by $^{1}$H-NMR. By visual analysis, it is concluded that the reaction rate is **fractional order** with respect to **L6**.

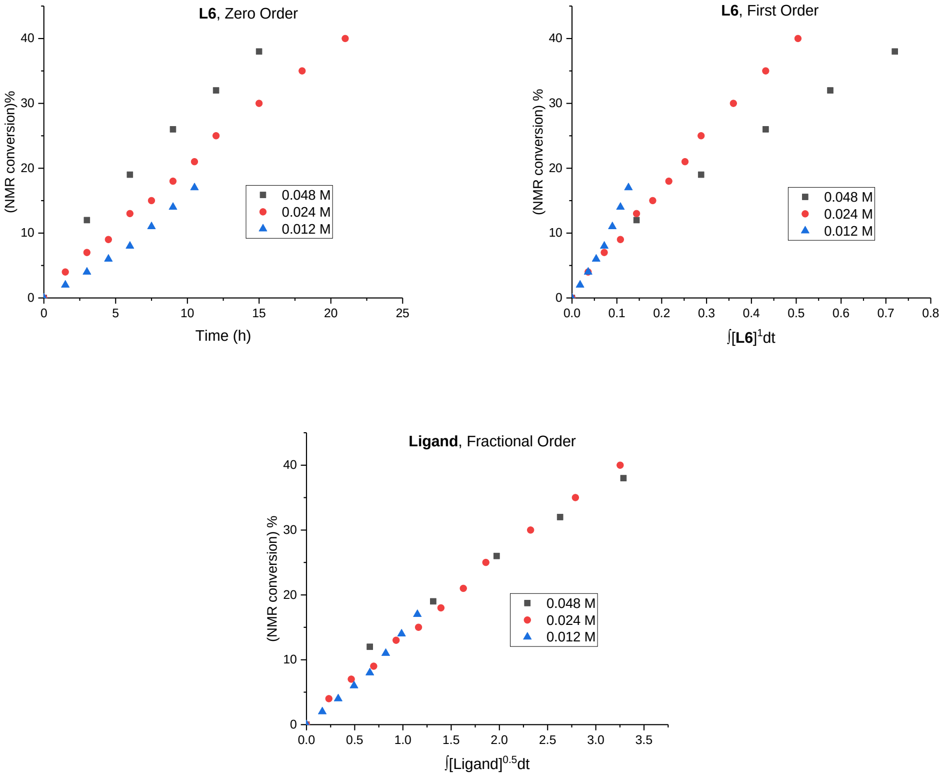


**Figure S7.** VTNA graphs for **L6**.

Representative procedure: A 10 mL vial was charged with the benzamide **1a** (0.15 mmol), indole **2a** (0.15 mmol), $Co(OAc)_2 \cdot 4H_2O$ (7.5 mg, 20 mol%), **L6** (10.6 mg, 20 mol%), Rhodamine 6G (10 mol%)/(15 mol%)/(20 mol%), diisopropylamine (27 µL, 0.20 mmol, 1.3 equiv.), 1,3,5-trimethoxybenzene (16.8 mg, 0.1 mmol) and a teflon-coated magnetic stirring bar. Then DCE (0.25 mL) and TFE (1 mL) were added. The vial was stirred at room temperature under blue LEDs (450 nm). Aliquots (25 µL) were removed every 1.5 hours to provide the following conversions as determined by $^1$H-NMR. By visual analysis, it is concluded that the reaction rate is **zero order** with respect to Rhodamine 6G.

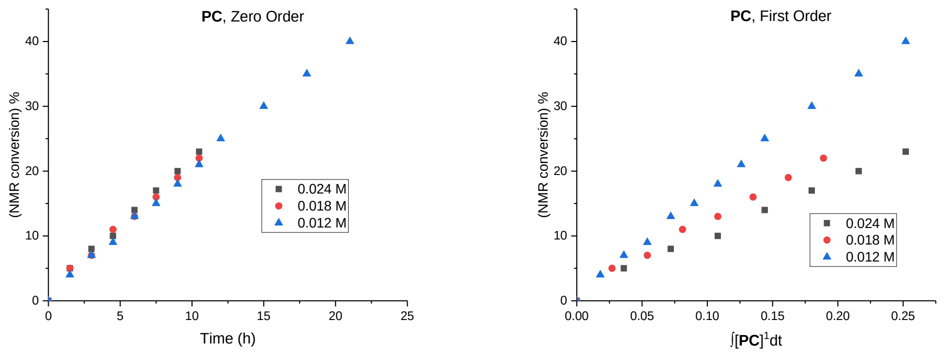


**Figure S8.** VTNA graphs for Rhodamine 6G.

Representative procedure: A 10 mL vial was charged with the benzamide **1a** (0.15 mmol), indole **2a** (0.15 mmol), $Co(OAc)_2 \cdot 4H_2O$ (7.5 mg, 20 mol%), **L6** (10.6 mg, 20 mol%), Rhodamine 6G (7.2 mg, 10 mol%), diisopropylamine (0.195 mmol)/(0.293 mmol)/(0.39 mmol), 1,3,5-trimethoxybenzene (16.8 mg, 0.1 mmol) and a teflon-coated magnetic stirring bar. Then DCE (0.25 mL) and TFE (1 mL) were added. The vial was stirred at room temperature under blue LEDs (450 nm). Aliquots (25 μL) were removed every 1.5 hours to provide the following conversions as determined by $^1$H-NMR. By visual analysis, it is concluded that the reaction rate is **zero order** with respect to DIPA.

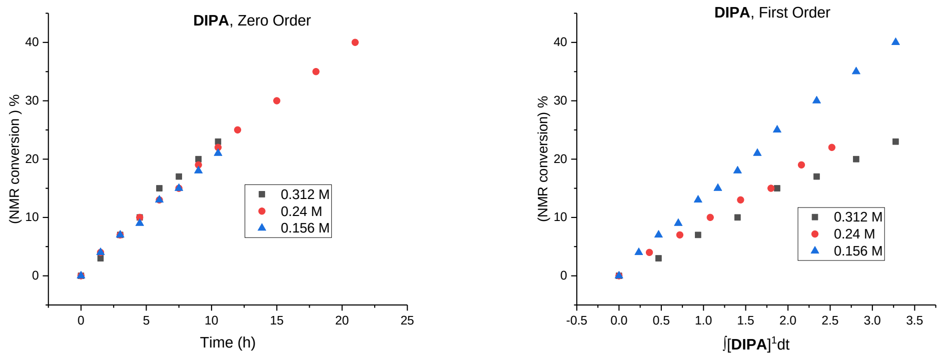


**Figure S9.** VTNA graphs for DIPA.

## 6.4 Synthesis and Characterization of Cobalt(III) Intermediate

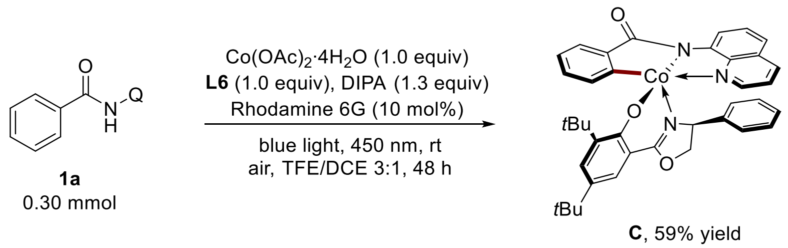


A 25 mL flask was charged with the amide **1a** (74.4 mg, 0.30 mmol, 1.0 equiv.), $Co(OAc)_2 \cdot 4H_2O$ (74.4 mg, 0.30 mmol, 1.0 equiv.), **L6** (105.4 mg, 0.30 mmol, 1.0 equiv.), Rhodamine 6G (7.2 mg, 10 mol%), DIPA (27 μL, 0.20 mmol, 1.3 equiv.) and a teflon-coated magnetic stirring bar. Then DCE (0.5 mL) and TFE (2 mL) were added. The mixture was stirred at room temperature under blue LEDs (450 nm) for 48 h. After completion of the reaction, the solvent was then removed under vacuum and the residue was purified by column chromatography on silica gel with DCM/MeOH (10:1) as eluent to give corresponding crude product **C** (118 mg, 59% yield).

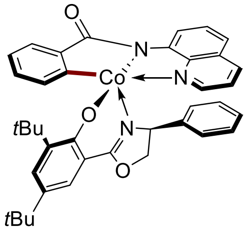

**$^1$H NMR** (300 MHz, $CD_3OD$_SPE) δ 9.54 (d, *J* = 4.6 Hz, 1H), 8.87 (d, *J* = 7.7 Hz, 1H), 8.16 (d, *J* = 8.3 Hz, 1H), 7.97 – 7.83 (m, 1H), 7.77 – 7.63 (m, 2H), 7.46 (d, *J* = 2.6 Hz, 1H), 7.38 – 7.25 (m, 4H), 7.19 (d, *J* = 8.0 Hz, 1H), 6.55 (t, *J* = 7.3 Hz, 1H), 6.45 (t, *J* = 7.4 Hz, 2H), 6.17 (d, *J* = 7.4 Hz, 2H), 1.54 (s, 9H), 1.30 (s, 9H) ppm.

**$^{13}$C NMR** (75 MHz, $CD_3OD$_SPE) δ 178.3 ($C_q$), 168.3 ($C_q$) 164.6 ($C_q$), 150.7 ($C_q$), 150.4 (CH), 148.4 ($C_q$), 147.6 ($C_q$), 142.2 ($C_q$), 140.6 ($C_q$), 138.9 (CH), 135.9 ($C_q$), 131.0 (CH), 130.9 ($C_q$), 129.9 (CH), 128.9 (CH), 128.1 (CH), 127.8 (CH), 126.9 (CH), 125.8 (CH), 125.4 (CH), 123.4 (CH), 123.3 (CH), 122.1 (CH), 120.2 (CH), 108.9 ($C_q$), 75.3 ($C_q$), 67.5 (CH), 36.6 ($CH_2$), 31.9 ($CH_3$), 30.2 ($CH_3$).

**HRMS (ESI):** *m/z* [M+H]+ calcd for $C_{39}H_{39}CoN_3O_3$: 656.2318; found: 656.2311.

**The Stoichiometric Reaction of Cobaltacycle C**

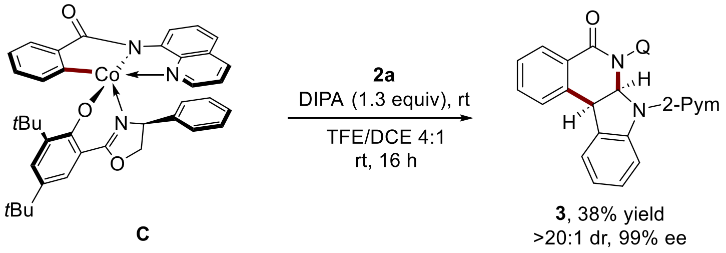

A 10 mL flask was charged with the cobaltacycle **C** (118 mg), indole **2a** (58.5 mg, 0.30 mmol, 1.0 equiv.), DIPA (54 µL, 0.39mmol, 1.3 equiv.) and a teflon-coated magnetic stirring bar. Then DCE (0.5 mL) and TFE (2 mL) were added. The mixture was stirred at room temperature for 24 h. After completion of the reaction, the solvent was then removed under vacuum and the residue was purified by column chromatography on silica gel with petroleum ether/ethyl acetate (1:1) as eluent to give corresponding product **3** (50.3 mg, 38% yield, >20:1 dr, 99% ee).

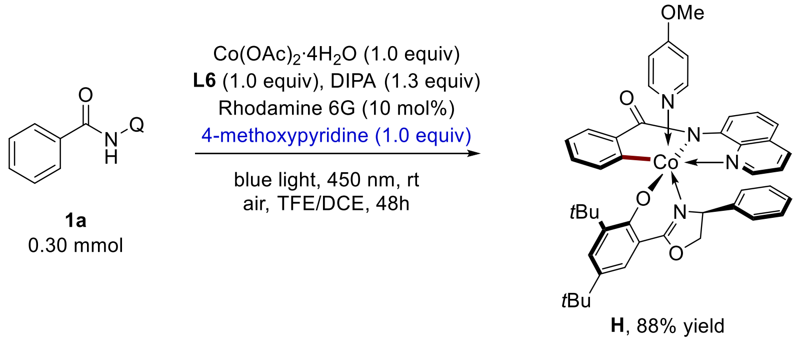


A 25 mL flask was charged with the amide **1a** (74.4 mg, 0.30 mmol, 1.0 equiv.), $Co(OAc)_2·4H_2O$ (74.4 mg, 0.30 mmol, 1.0 equiv.), **L6** (105.4 mg, 0.30 mmol, 1.0 equiv.), Rhodamine 6G (7.2 mg, 10 mol%), DIPA (27 µL, 0.20 mmol, 1.3 equiv.) and a teflon-coated magnetic stirring bar. Then DCE (0.5 mL) and TFE (2 mL) were added. The mixture was stirred at room temperature under blue LEDs (450 nm) for 48 h. After completion of the reaction, the solvent was then removed under vacuum and the residue was purified by column chromatography on silica gel with petroleum ether/ethyl acetate (1:1) as eluent to give corresponding product **H** (202 mg, 88% yield).

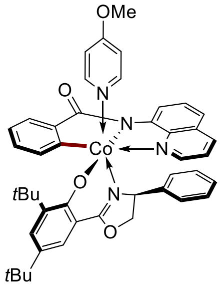


**$^1$H NMR** (400 MHz, $CDCl_3$) δ 9.49 (d, *J* = 4.5 Hz, 1H), 9.03 (d, *J* = 6.0 Hz, 1H), 7.99 – 7.82 (m, 4H), 7.65 (d, *J* = 7.4 Hz, 1H), 7.53 (dd, *J* = 8.4, 4.6 Hz, 1H), 7.46 (s, 1H), 7.41 (s, 1H), 7.37 – 7.19 (m, 4H), 6.98 (d, *J* = 8.1 Hz, 1H), 6.61 (t, *J* = 7.3 Hz, 1H), 6.50 (t, *J* = 7.5 Hz, 2H), 6.38 – 6.27 (m, 4H), 4.37 (d, *J* = 5.9 Hz, 1H), 4.01 (t, *J* = 9.0 Hz, 1H), 3.76 (dd, *J* = 8.4, 3.5 Hz, 1H), 3.62 (s, 3H), 1.40 (s, 9H), 1.36 (d, *J* = 1.4 Hz, 9H) ppm;

**$^{13}$C NMR** (101 MHz, CDCl3) δ 168.5 ($C_q$), 167.0 ($C_q$), 166.0 ($C_q$), 164.2 ($C_q$), 153.0 (CH), 147.6 (CH), 140.7 ($C_q$), 139.6 ($C_q$), 136.7 (CH), 135.8 (CH), 134.7 ($C_q$), 129.9 (CH), 129.0 (CH), 128.3 (CH), 126.9 (CH), 126.4 (CH), 124.6 (CH), 123.9 (CH), 123.1 (CH), 121.8 (CH), 121.0 (CH),

117.7 (CH), 109.1 (CH), 109.1 ($C_q$), 74.4 ($C_q$), 66.0 (CH), 55.3 ($CH_3$), 35.4 ($C_q$), 34.0 ($CH_2$), 31.53 ($CH_3$), 29.7 ($CH_3$) ppm.

**HRMS (ESI):** *m/z* [M+H]+ calcd for $C_{45}H_{46}CoN_4O_4$: 765.2846; found: 765.2846.

**The Stoichiometric Reaction of Cobaltacycle H**

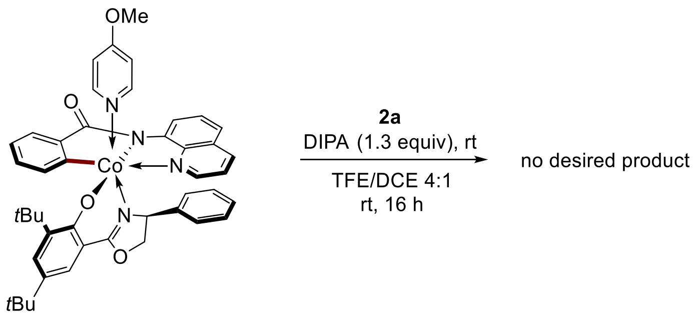

**H**, 88% yield

A 10 mL flask was charged with the cobaltacycle intermediate **H** (76.5 mg, 0.10 mmol, 1.0 equiv.), indole **2a** (19.5 mg, 0.10 mmol, 1.0 equiv.), DIPA (18 μL, 0.13 mmol, 1.3 equiv.) and a teflon-coated magnetic stirring bar. Then DCE (0.25 mL) and TFE (1 mL) were added. The mixture was stirred at room temperature for 24 h. No desired product **3** was detected by TLC.

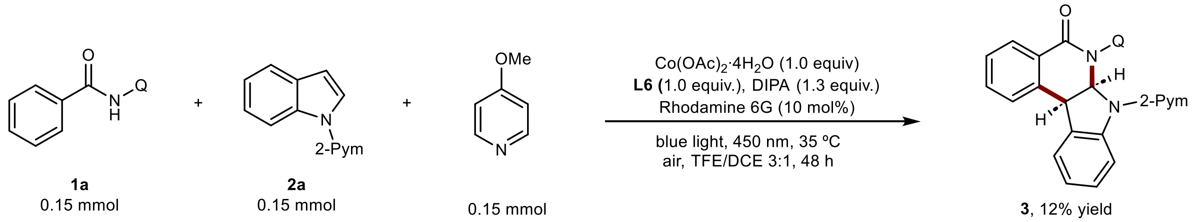


A 10 mL vial was charged with the amide **1a** (0.15 mmol, 1.0 equiv.), indole **2a** (0.15 mmol, 1.0 equiv.), $Co(OAc)_2 \cdot 4H_2O$ (7.5 mg, 20 mol%), **L6** (10.6 mg, 20 mol%), Rhodamine 6G (7.2 mg, 10 mol%), DIPA (27 μL, 0.20 mmol, 1.3 equiv.), 4-methoxypyridine (0.15 mmol, 1.0 equiv.) and a teflon-coated magnetic stirring bar. Then DCE (0.25 mL) and TFE (1 mL) were added. The vial was stirred at room temperature under blue LEDs (450 nm) for 48 h. After completion of the reaction, the solvent was then removed under vacuum and the residue was purified by column chromatography on silica gel with petroleum ether/ethyl acetate (3:1-1:1) as eluent to give corresponding product **3** (7.9 mg, 12% yield).

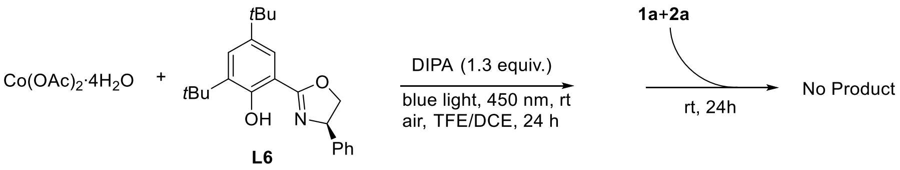


A 10 mL vial was charged with the $Co(OAc)_2·4H_2O$ (7.5 mg, 20 mol%), **L6** (10.6 mg, 20 mol%), Rhodamine 6G (7.2 mg, 10 mol%), DIPA (27 μL, 0.20 mmol, 1.3 equiv.) and a teflon-coated magnetic stirring bar. Then DCE (0.25 mL) and TFE (1 mL) were added. The vial was stirred at room temperature under blue LEDs (450 nm) for 24 h. Then remove the blue light and add **1a** (0.15 mmol, 1.0 equiv.) and **2a** (0.15 mmol, 1.0 equiv.) to the reaction and continue the reaction at 35 °C for 24h. The reaction was monitored by TLC and no desired product **3** was observed.

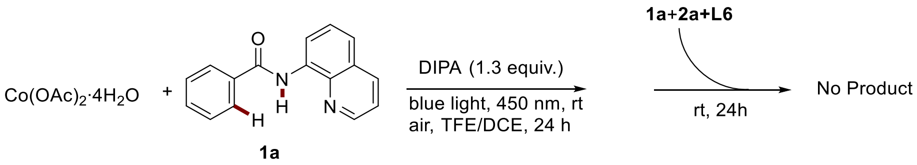


A 10 mL vial was charged with the $Co(OAc)_2·4H_2O$ (7.5 mg, 20 mol%), **1a** (0.15 mmol, 1.0 equiv.), Rhodamine 6G (7.2 mg, 10 mol%), DIPA (27 μL, 0.20 mmol, 1.3 equiv.) and a teflon-coated magnetic stirring bar. Then DCE (0.25 mL) and TFE (1 mL) were added. The vial was stirred at room temperature under blue LEDs (450 nm) for 24 h. Then remove the blue light and add **1a** (0.15 mmol, 1.0 equiv.), **2a** (0.15 mmol, 1.0 equiv.) and **L6** (10.6 mg, 20 mol%) to the reaction and continue the reaction at 35 °C for 24h. The reaction was monitored by TLC and no desired product **3** was observed.

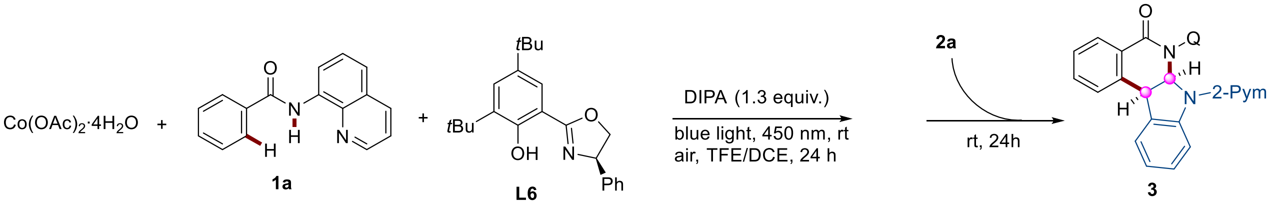


A 10 mL vial was charged with the $Co(OAc)_2·4H_2O$ (7.5 mg, 20 mol%), **L6** (10.6 mg, 20 mol%), **1a** (0.15 mmol, 1.0 equiv.), Rhodamine 6G (7.2 mg, 10 mol%), DIPA (27 μL, 0.20 mmol, 1.3 equiv.) and a teflon-coated magnetic stirring bar. Then DCE (0.25 mL) and TFE (1 mL) were added. The vial was stirred at room temperature under blue LEDs (450 nm) for 24 h. Then remove

the blue light and add **2a** (0.15 mmol, 1.0 equiv.) to the reaction and continue the reaction at 35 °C for 24h. The reaction was monitored by TLC and desired product **3** was observed.

Based on these mechanistic investigations and VTNA experiments, a plausible mechanism is proposed. We found an inverse-first-order dependence on benzamide (**2a**), and a fractional order (0.5) for $Co(OAc)_2 \cdot 4H_2O$ and **L6**, which we attributed to the coordination of benzamide (**1a**) with cobalt to generate an ineffective catalytic intermediate **B2**, or **L6** with cobalt to generate an ineffective catalytic intermediate **B1**.

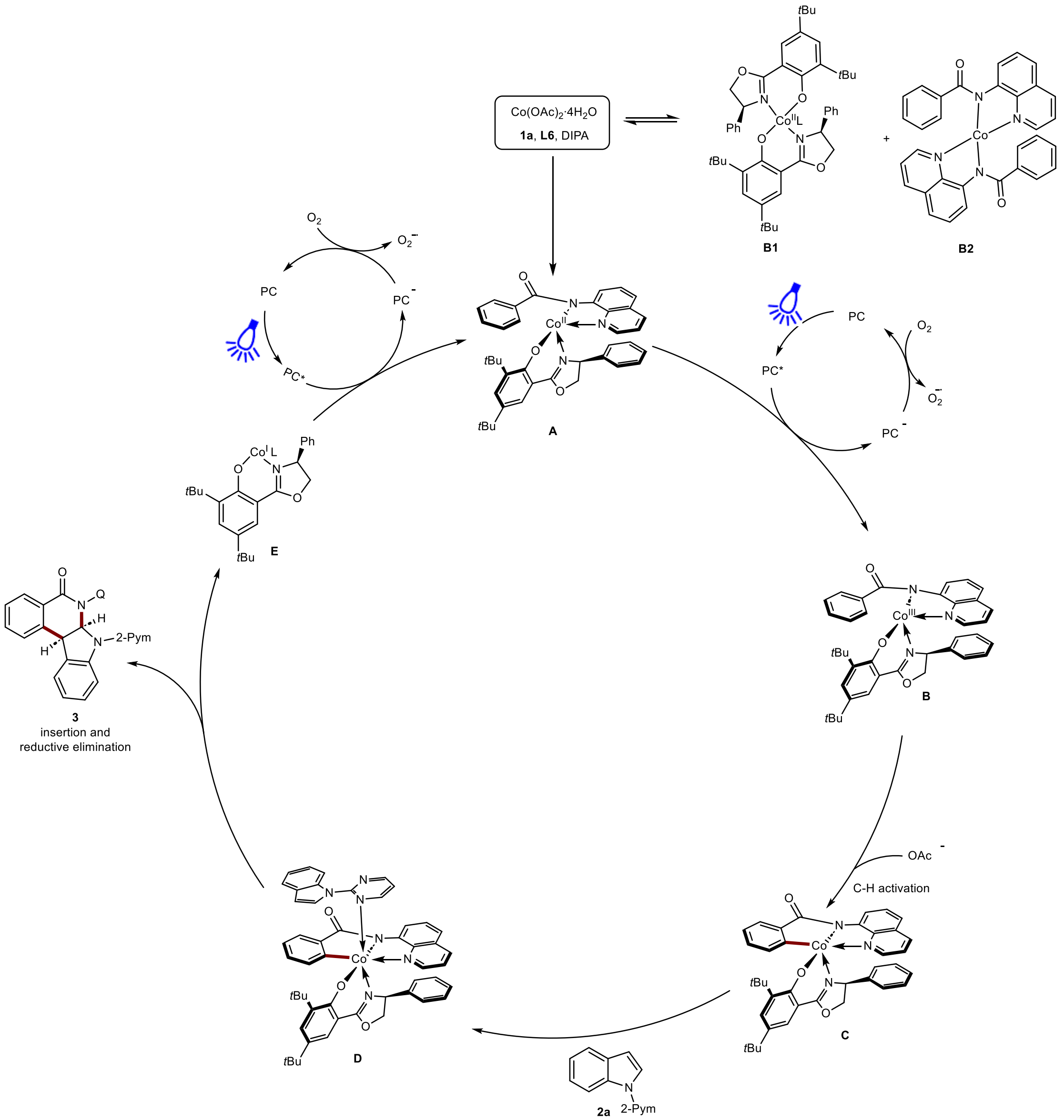

### 6.5 Cyclic Voltammetry Measurements

The cyclic voltammetry measurements were carried out using a Metrohm Autolab PGSTAT204 workstation, and the following analysis was performed with Nova 2.1 software. For all experiments a glassy-carbon (GC) electrode (3 mm-diameter, disc-electrode) was used as the working electrode and a saturated calomel electrode (SCE) was used as the reference electrode. The measurements were recorded at a scan rate of 100 mVs–1. The working temperature was 298 K, if not indicated otherwise. The solutions were heated at rt for 3 hours before measurement to ensure that the components were completely dissolved. Dry nitrogen was bubbled through the solutions for at least 5 min before the experiment was performed. The experiments were performed under a constant flow of dry nitrogen.

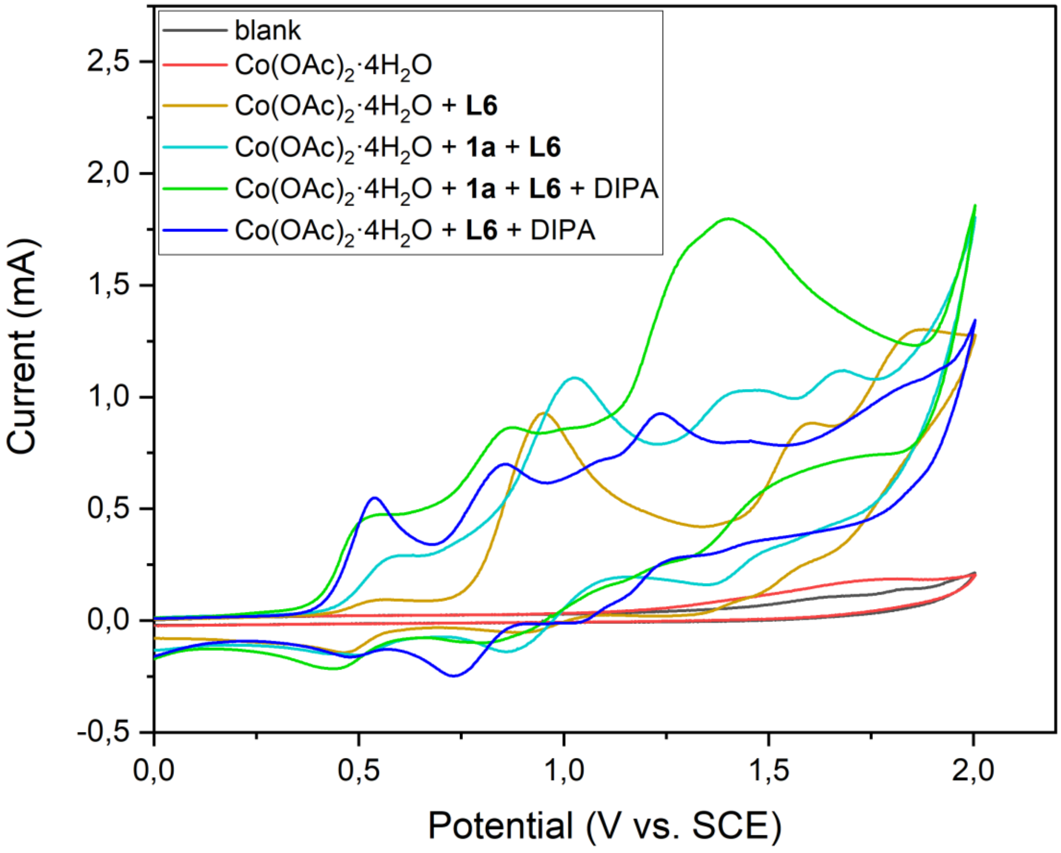


**Figure S9.** Cyclic voltammograms at 100 mV/s. *n*-$Bu_4NPF_6$ (0.1 M in TFE/DCE 4:1). concentration of substrates 10 mM. Blank; $Co(OAc)_2 \cdot 4H_2O$; $Co(OAc)_2 \cdot 4H_2O$ and **L6**; $Co(OAc)_2 \cdot 4H_2O$, substrate **1a** and **L6**; $Co(OAc)_2 \cdot 4H_2O$, substrate **1a**, **L6** and DIPA; $Co(OAc)_2 \cdot 4H_2O$, **L6** and DIPA.

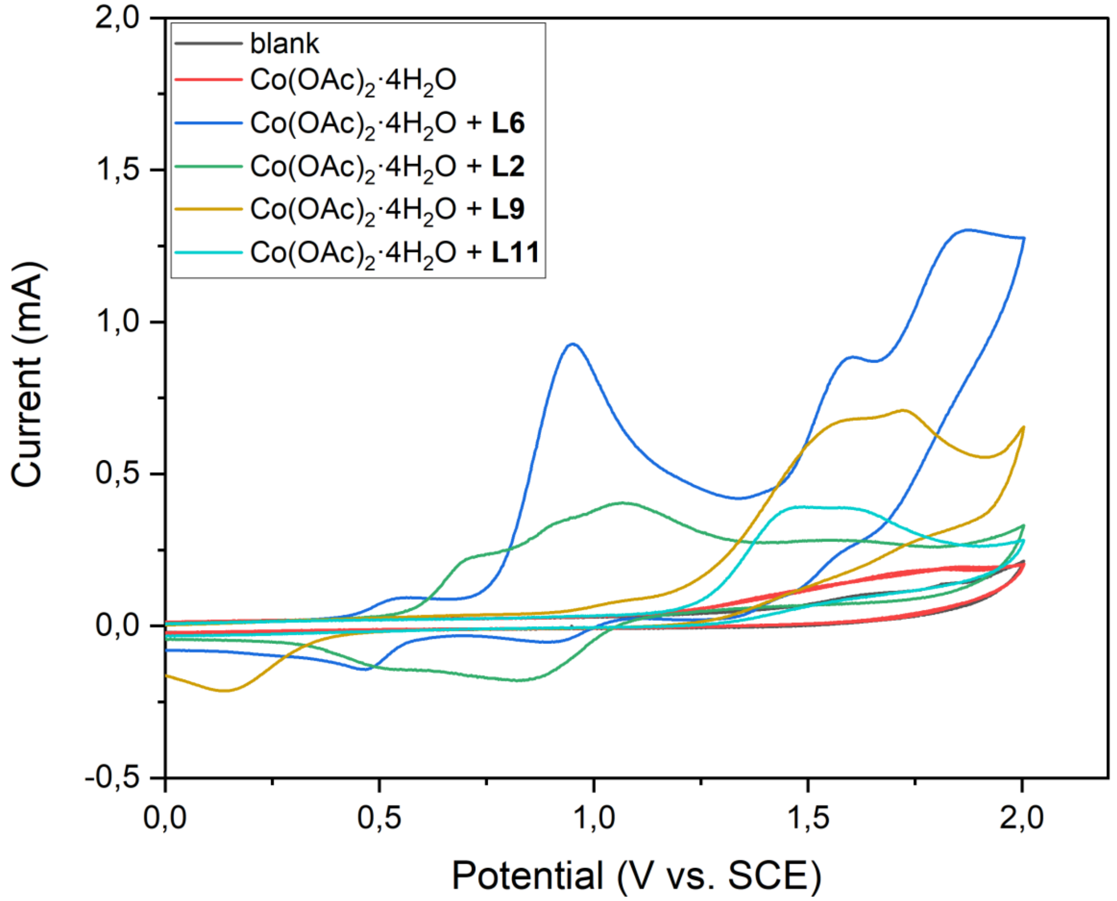


**Figure S10.** Cyclic voltammograms at 100 mV/s. *n*-$Bu_4NPF_6$ (0.1 M in TFE/DCE 4:1). concentration of substrates 10 mM. Blank; $Co(OAc)_2{\cdot}4H_2O$; $Co(OAc)_2{\cdot}4H_2O$ and **L6**; $Co(OAc)_2{\cdot}4H_2O$ and **L2**; $Co(OAc)_2{\cdot}4H_2O$ and **L9**; $Co(OAc)_2{\cdot}4H_2O$ and **L11**.

## 6.6 Thermal and Electrochemical Conditions

Thermal condition:

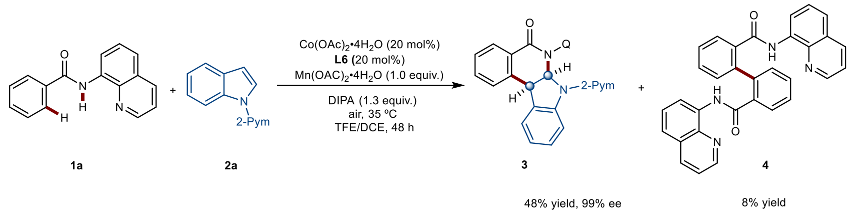


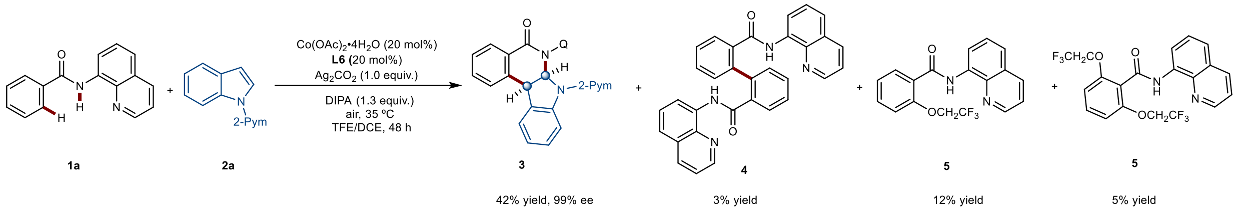

Electrochemical condition:

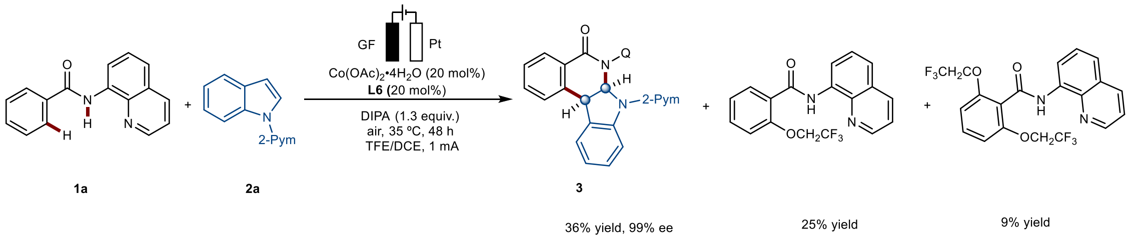

## 6.7 Computational Studies

All calculations were performed using the Gaussian 16, Revision A.03 package.[7] All structures were optimized at the TPSS[8] level of theory in combination with D3 dispersion corrections with the Becke-Johnson damping scheme (D3BJ).[9,10] Analytical frequency calculations were carried out at the same level of theory in order to identify each stationary point as either an intermediate (no imaginary frequencies) or a transition state (only one imaginary frequency) and to provided thermal and non-thermal corrections to the Gibbs free energy at 308.15 K and 1 atm. All atoms were described with the def2-SVP basis set.[11,12] The electronic energy was then refined through wB97X-D[13] single-point calculations on the optimized with a def2-TZVPP basis set.[11,12] Solvent effects were included implicitly through the use of the SMD[14] model for 2,2,2-trifluoroethanol (TFE). Energies reported herein are based on gas-phase Gibbs free energies with a def2-SVP basis set for which the electronic energies were corrected to wB97X-D a def2-TZVPP basis set and solvent effects.

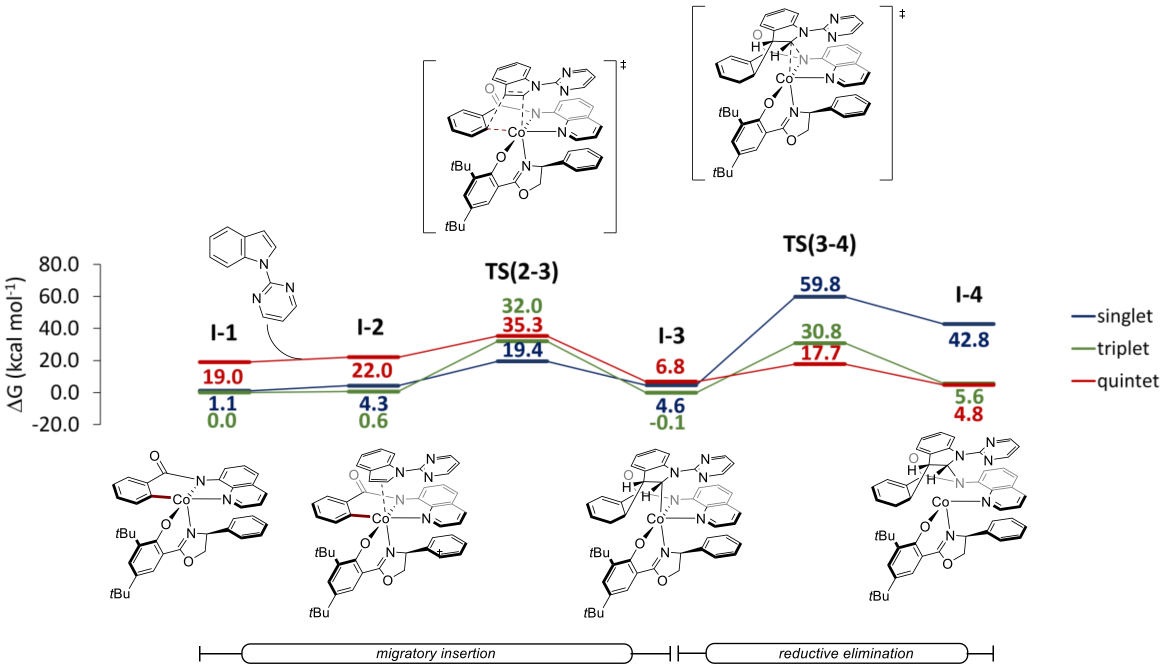


**Figure S11.** Computed relative Gibbs free energies ($\Delta G_{308.15}$) in kcal mol$^{-1}$ for the migratory insertion and reductive elimination elementary steps for the major enantiomer at the wB97X-D/def2 TZVPP+SMD(TFE)//TPSS-D3(BJ)/def2-SVP level of theory with the chiral ligand **L6**.

**Table S10**. Calculated electronic energies at the wB97X-D/def2-TZVPP+SMD(TFE)//TPSS-D3(BJ)/def2-SVP level of theory and Gibbs free energies with dispersion corrections for all structures in the present work (all in Hartree).[a]

| Structure | Electronic Energy | Total Gibbs Free Energy |
|---|---|---|
| **I-1$^1$** | -3282.013383 | -3281.412891 |
| **I-1$^3$** | -3282.012632 | -3281.414582 |
| **I-1$^5$** | -3281.977622 | -3281.384272 |
| **I-2$^1$** | -3909.009228 | -3908.233653 |
| **I-2$^3$** | -3909.008323 | -3908.239587 |
| **I-2$^5$** | -3908.970923 | -3908.205439 |
| **TS(2-3)$^1$** | -3908.985537 | -3908.209630 |
| **TS(2-3)$^3$** | -3908.959487 | -3908.189523 |
| **TS(2-3)$^5$** | -3908.949766 | -3908.184273 |
| **I-3$^1$** | -3909.009034 | -3908.233291 |
| **I-3$^3$** | -3909.011789 | -3908.240783 |
| **I-3$^5$** | -3908.997720 | -3908.229670 |
| **TS(3-4)$^1$** | -3908.915487 | -3908.145281 |
| **TS(3-4)$^3$** | -3908.960876 | -3908.191406 |
| **TS(3-4)$^5$** | -3908.979945 | -3908.212325 |
| **I-4$^1$** | -3908.945031 | -3908.172393 |
| **I-4$^3$** | -3909.002714 | -3908.231611 |
| **I-4$^5$** | -3909.001704 | -3908.232971 |
| **2a** | -626.972154 | -626.825973 |

[a] Superscripts correspond to the spin state of the respective stationary point.

**Cartesian coordinates of the optimized structures**
**I-1$^1$**
Lowest frequency = 13.3545 cm$^{-1}$
Charge = 0, Multiplicity = 1

84

| | | | |
|---|---|---|---|
| C | 1.428911 | 3.283197 | -0.112499 |
| C | -0.987638 | 3.111834 | 0.016656 |
| C | 1.340149 | 4.625472 | -0.511890 |
| C | -1.079205 | 4.459257 | -0.380792 |
| H | -1.905226 | 2.544901 | 0.206568 |
| C | 0.075754 | 5.214943 | -0.647369 |
| H | 2.264725 | 5.182559 | -0.700908 |
| H | -2.067272 | 4.922623 | -0.487229 |
| H | -0.014910 | 6.261205 | -0.958780 |
| N | 2.489324 | 1.293425 | 0.542754 |

| | | | |
|---|---|---|---|
| C | 2.927554 | -0.923921 | 1.204312 |
| C | 0.980066 | -2.191741 | 1.467840 |
| C | 3.771664 | -2.031560 | 1.526302 |
| C | 3.463416 | 0.363285 | 0.863041 |
| C | 1.736625 | -3.345542 | 1.775126 |
| H | -0.114471 | -2.202039 | 1.445870 |
| C | 3.122453 | -3.265339 | 1.807819 |
| C | 5.179757 | -1.825637 | 1.540407 |
| C | 4.856495 | 0.529847 | 0.900306 |
| H | 1.219279 | -4.285201 | 1.989916 |
| H | 3.726984 | -4.146558 | 2.049154 |
| C | 5.686116 | -0.565339 | 1.243216 |
| H | 5.844275 | -2.659295 | 1.788013 |
| H | 5.276792 | 1.503905 | 0.648761 |
| H | 6.770047 | -0.406228 | 1.261683 |
| N | 1.560972 | -1.023919 | 1.194102 |
| C | 2.733655 | 2.587189 | 0.083769 |
| O | 3.847280 | 3.077614 | -0.122355 |
| O | -0.974706 | 0.221988 | 1.278490 |
| C | -2.073939 | -0.082792 | 0.634552 |
| C | -2.095221 | -0.257765 | -0.788933 |
| C | -3.306205 | -0.258234 | 1.359574 |
| C | -3.292203 | -0.598853 | -1.469937 |
| C | -0.883679 | -0.081726 | -1.545720 |
| C | -4.445800 | -0.597331 | 0.631409 |
| C | -4.482840 | -0.778553 | -0.778289 |
| H | -3.238482 | -0.713027 | -2.554133 |
| N | 0.305478 | 0.251302 | -1.097026 |
| O | -0.926137 | -0.280196 | -2.885150 |
| H | -5.381197 | -0.726150 | 1.181973 |
| C | 1.281067 | 0.305640 | -2.207632 |
| C | 0.344526 | 0.159247 | -3.436134 |
| H | 1.770053 | 1.292385 | -2.203470 |
| C | 2.328450 | -0.782767 | -2.074678 |
| H | 0.174754 | 1.120218 | -3.950016 |
| C | 1.937536 | -2.122205 | -1.888912 |
| C | 3.694448 | -0.465885 | -2.129067 |
| C | 2.901103 | -3.128328 | -1.756595 |
| H | 0.872945 | -2.375051 | -1.828583 |
| C | 4.660672 | -1.474069 | -2.001438 |
| H | 4.003113 | 0.579549 | -2.238809 |
| C | 4.266194 | -2.804747 | -1.813097 |
| H | 2.587817 | -4.165664 | -1.600009 |
| H | 5.723192 | -1.213558 | -2.026401 |
| H | 5.021360 | -3.589244 | -1.698245 |
| C | 0.273138 | 2.513620 | 0.162772 |
| Co | 0.696912 | 0.719112 | 0.634633 |
| C | -3.345028 | -0.046624 | 2.884019 |

| | | | |
|---|---|---|---|
| C | -5.809323 | -1.149349 | -1.461172 |
| C | -6.853899 | -0.038676 | -1.190051 |
| H | -7.819142 | -0.292070 | -1.665202 |
| H | -7.033934 | 0.092748 | -0.108998 |
| H | -6.509155 | 0.928073 | -1.596667 |
| C | -6.325080 | -2.491412 | -0.886030 |
| H | -6.489424 | -2.428439 | 0.203670 |
| H | -7.285376 | -2.769885 | -1.357171 |
| H | -5.598256 | -3.301098 | -1.073564 |
| C | -5.644574 | -1.302641 | -2.985226 |
| H | -5.300380 | -0.363332 | -3.452360 |
| H | -4.922079 | -2.098799 | -3.236773 |
| H | -6.614027 | -1.570334 | -3.440914 |
| C | -2.939161 | 1.413150 | 3.212579 |
| H | -1.927868 | 1.637823 | 2.842409 |
| H | -3.647411 | 2.122931 | 2.749497 |
| H | -2.958318 | 1.572966 | 4.306101 |
| C | -4.755671 | -0.286807 | 3.458701 |
| H | -5.498664 | 0.408835 | 3.030109 |
| H | -5.102809 | -1.320205 | 3.280331 |
| H | -4.735484 | -0.123197 | 4.550225 |
| C | -2.375782 | -1.034484 | 3.581732 |
| H | -2.658556 | -2.078557 | 3.355679 |
| H | -1.339732 | -0.868233 | 3.251800 |
| H | -2.421975 | -0.896510 | 4.677320 |
| H | 0.683268 | -0.601089 | -4.154645 |

**I-1$^3$**

Lowest frequency = 16.2695 cm$^{-1}$

Charge = 0, Multiplicity = 3

84

| | | | |
|---|---|---|---|
| C | 1.032733 | 3.015155 | 1.066903 |
| C | -1.317464 | 2.472561 | 1.252023 |
| C | 0.754062 | 4.389216 | 1.151453 |
| C | -1.600065 | 3.847479 | 1.350514 |
| H | -2.147029 | 1.760486 | 1.293985 |
| C | -0.574034 | 4.807269 | 1.292591 |
| H | 1.587781 | 5.099504 | 1.113502 |
| H | -2.640182 | 4.171938 | 1.474784 |
| H | -0.815124 | 5.873255 | 1.365418 |
| N | 2.369527 | 1.090494 | 0.992366 |
| C | 3.156829 | -1.122849 | 0.838002 |
| C | 1.431666 | -2.669395 | 0.521099 |
| C | 4.162994 | -2.135607 | 0.795669 |
| C | 3.480455 | 0.263251 | 1.013764 |

| | | | |
|---|---|---|---|
| C | 2.358697 | -3.734701 | 0.461551 |
| H | 0.352448 | -2.824729 | 0.421200 |
| C | 3.715275 | -3.470929 | 0.597850 |
| C | 5.522079 | -1.739859 | 0.950158 |
| C | 4.830320 | 0.612900 | 1.168441 |
| H | 1.993565 | -4.754781 | 0.310529 |
| H | 4.449447 | -4.282841 | 0.554077 |
| C | 5.824297 | -0.395779 | 1.135941 |
| H | 6.310454 | -2.498662 | 0.924574 |
| H | 5.093223 | 1.663531 | 1.292697 |
| H | 6.870539 | -0.094460 | 1.258063 |
| N | 1.823748 | -1.408806 | 0.704491 |
| C | 2.418544 | 2.489709 | 0.965988 |
| O | 3.448058 | 3.161912 | 0.870392 |
| O | -0.897002 | -0.691517 | 1.050703 |
| C | -2.055639 | -0.568094 | 0.436186 |
| C | -2.160681 | -0.062013 | -0.903985 |
| C | -3.258208 | -0.990860 | 1.101942 |
| C | -3.411883 | 0.002274 | -1.559199 |
| C | -0.979676 | 0.387862 | -1.615520 |
| C | -4.463406 | -0.890459 | 0.403390 |
| C | -4.582389 | -0.403547 | -0.922993 |
| H | -3.425028 | 0.389968 | -2.579175 |
| N | 0.227725 | 0.480922 | -1.137314 |
| O | -1.119084 | 0.769560 | -2.912303 |
| H | -5.378151 | -1.203043 | 0.913035 |
| C | 1.126210 | 1.044272 | -2.155519 |
| C | 0.206057 | 1.103858 | -3.408721 |
| H | 1.415420 | 2.059425 | -1.826774 |
| C | 2.379976 | 0.205176 | -2.307855 |
| H | 0.155169 | 2.101462 | -3.870045 |
| C | 2.278822 | -1.174434 | -2.569586 |
| C | 3.649627 | 0.780801 | -2.145828 |
| C | 3.429842 | -1.963261 | -2.672650 |
| H | 1.289590 | -1.636465 | -2.666219 |
| C | 4.804443 | -0.007990 | -2.252478 |
| H | 3.734924 | 1.844319 | -1.897056 |
| C | 4.697363 | -1.379427 | -2.515216 |
| H | 3.339435 | -3.037614 | -2.863765 |
| H | 5.788166 | 0.448747 | -2.106858 |
| H | 5.598909 | -1.996892 | -2.583559 |
| C | 0.009694 | 2.038482 | 1.106607 |
| Co | 0.690674 | 0.253087 | 0.809416 |
| C | -3.203073 | -1.512568 | 2.550404 |
| C | -5.967118 | -0.337146 | -1.587013 |
| C | -6.888025 | 0.589742 | -0.755409 |
| H | -7.893067 | 0.644067 | -1.211941 |
| H | -7.005740 | 0.221970 | 0.278567 |

| | | | |
|---|---|---|---|
| H | -6.472124 | 1.611244 | -0.707440 |
| C | -6.579018 | -1.758816 | -1.638922 |
| H | -6.688246 | -2.190138 | -0.628855 |
| H | -7.580877 | -1.729793 | -2.104632 |
| H | -5.940089 | -2.437216 | -2.230921 |
| C | -5.888682 | 0.213670 | -3.023738 |
| H | -5.480225 | 1.239124 | -3.043530 |
| H | -5.257147 | -0.422590 | -3.668207 |
| H | -6.899494 | 0.244627 | -3.466813 |
| C | -2.632318 | -0.411602 | 3.480411 |
| H | -1.621812 | -0.109690 | 3.168050 |
| H | -3.282803 | 0.480760 | 3.468071 |
| H | -2.583745 | -0.785853 | 4.519221 |
| C | -4.603135 | -1.892581 | 3.074276 |
| H | -5.289422 | -1.027335 | 3.080392 |
| H | -5.063535 | -2.698383 | 2.475215 |
| H | -4.514663 | -2.256098 | 4.112804 |
| C | -2.312561 | -2.779299 | 2.622858 |
| H | -2.710385 | -3.570773 | 1.962487 |
| H | -1.280640 | -2.548995 | 2.320552 |
| H | -2.299058 | -3.169368 | 3.656911 |
| H | 0.482900 | 0.355283 | -4.170053 |

**I-1$^5$**

Lowest frequency = 10.4911 cm$^{-1}$

Charge = 0, Multiplicity = 5

84

| | | | |
|---|---|---|---|
| C | 1.332018 | 3.144732 | 0.901991 |
| C | -0.984827 | 2.861467 | 1.549320 |
| C | 1.167985 | 4.538929 | 0.826450 |
| C | -1.159583 | 4.255286 | 1.467010 |
| H | -1.838112 | 2.230944 | 1.826139 |
| C | -0.086943 | 5.092841 | 1.109519 |
| H | 2.027908 | 5.161161 | 0.553296 |
| H | -2.141167 | 4.694320 | 1.683939 |
| H | -0.234633 | 6.177337 | 1.058686 |
| N | 2.541905 | 1.117583 | 0.730448 |
| C | 3.199565 | -1.127019 | 0.927908 |
| C | 1.405772 | -2.617989 | 1.043005 |
| C | 4.161252 | -2.183297 | 0.970875 |
| C | 3.600093 | 0.254142 | 0.822787 |
| C | 2.281392 | -3.725866 | 1.040984 |
| H | 0.318102 | -2.735617 | 1.089774 |
| C | 3.653928 | -3.509978 | 1.018295 |
| C | 5.544926 | -1.843443 | 0.957616 |

| | | | |
|---|---|---|---|
| C | 4.977615 | 0.544068 | 0.836384 |
| H | 1.867584 | -4.737882 | 1.077670 |
| H | 4.353364 | -4.353034 | 1.036372 |
| C | 5.922077 | -0.505502 | 0.908658 |
| H | 6.294080 | -2.641048 | 0.987146 |
| H | 5.291456 | 1.585502 | 0.753437 |
| H | 6.987203 | -0.248931 | 0.903945 |
| N | 1.855867 | -1.361617 | 0.995300 |
| C | 2.653710 | 2.504611 | 0.625437 |
| O | 3.684864 | 3.119060 | 0.330786 |
| O | -1.009100 | -0.558892 | 1.094494 |
| C | -2.156280 | -0.495304 | 0.472157 |
| C | -2.249930 | -0.080547 | -0.904479 |
| C | -3.375862 | -0.865354 | 1.149947 |
| C | -3.496769 | -0.052737 | -1.577905 |
| C | -1.062194 | 0.298481 | -1.632554 |
| C | -4.569423 | -0.802448 | 0.433205 |
| C | -4.673299 | -0.405053 | -0.927572 |
| H | -3.497835 | 0.262011 | -2.622814 |
| N | 0.158417 | 0.411824 | -1.165147 |
| O | -1.189784 | 0.589790 | -2.950449 |
| H | -5.492523 | -1.076227 | 0.950424 |
| C | 1.068498 | 0.852181 | -2.235270 |
| C | 0.136264 | 0.857189 | -3.480175 |
| H | 1.416414 | 1.872956 | -1.995184 |
| C | 2.271122 | -0.066948 | -2.337459 |
| H | 0.105050 | 1.825377 | -4.002236 |
| C | 2.089695 | -1.462975 | -2.359860 |
| C | 3.572246 | 0.457023 | -2.380228 |
| C | 3.193727 | -2.320382 | -2.423752 |
| H | 1.077666 | -1.878493 | -2.296280 |
| C | 4.678895 | -0.401644 | -2.447543 |
| H | 3.723476 | 1.539959 | -2.313746 |
| C | 4.492953 | -1.789930 | -2.468312 |
| H | 3.042056 | -3.404781 | -2.424594 |
| H | 5.689822 | 0.017439 | -2.459108 |
| H | 5.358836 | -2.458925 | -2.504130 |
| C | 0.264809 | 2.288075 | 1.259138 |
| Co | 0.672759 | 0.345475 | 0.738495 |
| C | -3.341455 | -1.302587 | 2.626271 |
| C | -6.052266 | -0.376849 | -1.605689 |
| C | -6.974672 | 0.609598 | -0.847424 |
| H | -7.976001 | 0.636992 | -1.314504 |
| H | -7.102266 | 0.314574 | 0.208488 |
| H | -6.553798 | 1.629987 | -0.866497 |
| C | -6.672824 | -1.795247 | -1.565648 |
| H | -6.792627 | -2.156351 | -0.529519 |
| H | -7.670996 | -1.792216 | -2.040181 |

| | | | |
|---|---|---|---|
| H | -6.033712 | -2.516063 | -2.104985 |
| C | -5.959137 | 0.073915 | -3.075952 |
| H | -5.543728 | 1.093163 | -3.162105 |
| H | -5.326669 | -0.608711 | -3.670161 |
| H | -6.966166 | 0.080735 | -3.528631 |
| C | -2.775131 | -0.155146 | 3.500596 |
| H | -1.754020 | 0.111435 | 3.190666 |
| H | -3.414186 | 0.742181 | 3.421495 |
| H | -2.751970 | -0.466106 | 4.560972 |
| C | -4.748868 | -1.648159 | 3.153999 |
| H | -5.431598 | -0.781559 | 3.103286 |
| H | -5.205506 | -2.484406 | 2.595171 |
| H | -4.674263 | -1.953327 | 4.212259 |
| C | -2.455881 | -2.564981 | 2.781305 |
| H | -2.844736 | -3.391406 | 2.159753 |
| H | -1.419992 | -2.349688 | 2.481249 |
| H | -2.455069 | -2.897331 | 3.835401 |
| H | 0.383689 | 0.055108 | -4.195304 |

**I-2[1]**
Lowest frequency = 12.6275 $cm^{-1}$
Charge = 0, Multiplicity = 1

108

| | | | |
|---|---|---|---|
| C | 0.805835 | 2.482970 | -2.037429 |
| C | -1.421942 | 2.945319 | -1.190618 |
| C | 0.706405 | 3.547817 | -2.948786 |
| C | -1.520357 | 4.013782 | -2.100358 |
| H | -2.258200 | 2.724999 | -0.517638 |
| C | -0.462628 | 4.317149 | -2.978684 |
| H | 1.555839 | 3.750250 | -3.610731 |
| H | -2.435613 | 4.617311 | -2.124387 |
| H | -0.557524 | 5.152844 | -3.680490 |
| N | 1.789967 | 0.620596 | -1.009644 |
| C | 2.054684 | -1.464469 | 0.051860 |
| C | 0.125101 | -2.184289 | 1.156790 |
| C | 2.759099 | -2.672398 | 0.353233 |
| C | 2.652822 | -0.415545 | -0.726584 |
| C | 0.724648 | -3.429991 | 1.453874 |
| H | -0.891085 | -1.936953 | 1.478534 |
| C | 2.035356 | -3.668067 | 1.066979 |
| C | 4.109329 | -2.790820 | -0.076773 |
| C | 3.995916 | -0.560657 | -1.112706 |
| H | 0.150430 | -4.182443 | 2.002380 |
| H | 2.525685 | -4.618209 | 1.305180 |
| C | 4.697741 | -1.739937 | -0.773807 |

| | | | |
|---|---|---|---|
| H | 4.666135 | -3.706499 | 0.145167 |
| H | 4.462054 | 0.235611 | -1.692373 |
| H | 5.742875 | -1.828122 | -1.091189 |
| N | 0.775030 | -1.240604 | 0.480415 |
| C | 2.016216 | 1.618530 | -1.945583 |
| O | 3.040350 | 1.744274 | -2.628491 |
| O | -1.444395 | 0.558887 | 0.980910 |
| C | -2.674348 | 0.204040 | 0.711359 |
| C | -3.080799 | -0.274929 | -0.577426 |
| C | -3.686219 | 0.293660 | 1.740412 |
| C | -4.434583 | -0.600846 | -0.853444 |
| C | -2.107970 | -0.445987 | -1.625623 |
| C | -4.994334 | -0.053654 | 1.407419 |
| C | -5.413743 | -0.496283 | 0.124276 |
| H | -4.671417 | -0.942301 | -1.862322 |
| N | -0.811032 | -0.258639 | -1.573724 |
| O | -2.562042 | -0.879062 | -2.829801 |
| H | -5.755705 | 0.024125 | 2.187739 |
| C | -0.210001 | -0.637019 | -2.874187 |
| C | -1.465622 | -0.745796 | -3.769777 |
| H | 0.440994 | 0.176693 | -3.224807 |
| C | 0.589308 | -1.922915 | -2.776360 |
| H | -1.635601 | 0.170821 | -4.360621 |
| C | 0.023492 | -3.071789 | -2.193444 |
| C | 1.896428 | -1.982320 | -3.286488 |
| C | 0.755487 | -4.263217 | -2.123480 |
| H | -0.990329 | -3.029597 | -1.780492 |
| C | 2.629277 | -3.175418 | -3.217095 |
| H | 2.351292 | -1.080240 | -3.710054 |
| C | 2.060524 | -4.317089 | -2.636997 |
| H | 0.309739 | -5.149497 | -1.659920 |
| H | 3.653968 | -3.205424 | -3.600412 |
| H | 2.637674 | -5.245678 | -2.573544 |
| C | -0.251459 | 2.173478 | -1.153351 |
| Co | 0.110724 | 0.584800 | -0.126049 |
| C | -3.321282 | 0.764084 | 3.161201 |
| C | -6.893374 | -0.834697 | -0.118537 |
| C | -7.757919 | 0.420651 | 0.154979 |
| H | -8.828517 | 0.195404 | -0.003355 |
| H | -7.637388 | 0.776318 | 1.193025 |
| H | -7.472067 | 1.245150 | -0.521277 |
| C | -7.324834 | -1.973341 | 0.838166 |
| H | -7.192929 | -1.685214 | 1.895604 |
| H | -8.390866 | -2.223379 | 0.686437 |
| H | -6.725289 | -2.882608 | 0.656810 |
| C | -7.144337 | -1.293613 | -1.567600 |
| H | -6.871396 | -0.508315 | -2.294017 |
| H | -6.567658 | -2.203370 | -1.810218 |

| | | | |
|---|---|---|---|
| H | -8.215029 | -1.525316 | -1.706606 |
| C | -2.772243 | 2.211289 | 3.109442 |
| H | -1.883371 | 2.264693 | 2.466314 |
| H | -3.537917 | 2.899287 | 2.709517 |
| H | -2.496441 | 2.552288 | 4.124007 |
| C | -4.542513 | 0.762142 | 4.103474 |
| H | -5.333058 | 1.449703 | 3.754563 |
| H | -4.980026 | -0.246409 | 4.209476 |
| H | -4.226046 | 1.098023 | 5.106479 |
| C | -2.261894 | -0.188253 | 3.770020 |
| H | -2.667741 | -1.212157 | 3.855100 |
| H | -1.360067 | -0.222379 | 3.142278 |
| H | -1.974380 | 0.153671 | 4.780827 |
| H | -1.466722 | -1.629376 | -4.423704 |
| C | 1.975927 | 2.958982 | 0.869526 |
| N | 2.472894 | 0.988490 | 1.912773 |
| C | 0.732758 | 2.314373 | 1.254324 |
| C | 1.076280 | 1.114815 | 1.887323 |
| C | 3.043193 | 2.096013 | 1.244357 |
| C | 2.242539 | 4.151946 | 0.180215 |
| C | 3.111715 | -0.099682 | 2.511664 |
| C | 4.374859 | 2.388241 | 0.920114 |
| C | 3.571201 | 4.462013 | -0.128002 |
| H | 1.420557 | 4.804133 | -0.128475 |
| N | 4.437933 | -0.219459 | 2.336882 |
| N | 2.316099 | -0.921063 | 3.224697 |
| C | 4.614664 | 3.586118 | 0.232123 |
| H | 5.174332 | 1.699390 | 1.188902 |
| H | 3.802503 | 5.381702 | -0.674397 |
| C | 5.007086 | -1.296819 | 2.892453 |
| C | 2.908781 | -1.986472 | 3.771403 |
| H | 5.644608 | 3.840258 | -0.039436 |
| C | 4.280261 | -2.240854 | 3.628309 |
| H | 6.088986 | -1.403663 | 2.740920 |
| H | 2.260058 | -2.660151 | 4.346172 |
| H | 4.755813 | -3.119228 | 4.072278 |
| H | -0.239728 | 2.794192 | 1.322532 |
| H | 0.484304 | 0.470536 | 2.529883 |

**I-2$^3$**
Lowest frequency = 12.7371 cm$^{-1}$
Charge = 0, Multiplicity = 3

108

| | | | |
|---|---|---|---|
| C | -0.416510 | -0.974846 | -2.973588 |
| C | 1.772399 | -1.693217 | -2.223245 |

| | | | |
|---|---|---|---|
| C | -0.178958 | -1.494743 | -4.256745 |
| C | 2.007164 | -2.228425 | -3.502760 |
| H | 2.548484 | -1.783568 | -1.456042 |
| C | 1.040836 | -2.127226 | -4.521326 |
| H | -0.964663 | -1.401337 | -5.014858 |
| H | 2.959973 | -2.731364 | -3.707377 |
| H | 1.244748 | -2.545936 | -5.512795 |
| N | -1.649785 | 0.025758 | -1.248628 |
| C | -2.282967 | 1.136725 | 0.726479 |
| C | -0.532179 | 1.315284 | 2.262507 |
| C | -3.174851 | 1.883898 | 1.554888 |
| C | -2.679121 | 0.641837 | -0.560437 |
| C | -1.333557 | 2.090831 | 3.130581 |
| H | 0.503347 | 1.053364 | 2.500794 |
| C | -2.648080 | 2.369686 | 2.782742 |
| C | -4.505903 | 2.091217 | 1.094720 |
| C | -4.005094 | 0.848719 | -0.969495 |
| H | -0.907979 | 2.452245 | 4.071290 |
| H | -3.289623 | 2.961040 | 3.444978 |
| C | -4.893545 | 1.564631 | -0.131764 |
| H | -5.206972 | 2.658268 | 1.715095 |
| H | -4.320836 | 0.469368 | -1.940701 |
| H | -5.922443 | 1.713641 | -0.477365 |
| N | -0.995205 | 0.859137 | 1.100236 |
| C | -1.701400 | -0.327340 | -2.601007 |
| O | -2.651523 | -0.113734 | -3.358031 |
| O | 1.469196 | -0.572054 | 0.784265 |
| C | 2.751295 | -0.296734 | 0.672123 |
| C | 3.249400 | 0.730446 | -0.201120 |
| C | 3.707825 | -1.013420 | 1.478849 |
| C | 4.633801 | 1.007475 | -0.289101 |
| C | 2.338855 | 1.532994 | -0.994657 |
| C | 5.059519 | -0.690365 | 1.344842 |
| C | 5.564123 | 0.303651 | 0.469773 |
| H | 4.942624 | 1.797849 | -0.975035 |
| N | 1.041901 | 1.438816 | -1.030514 |
| O | 2.875222 | 2.499509 | -1.787601 |
| H | 5.779844 | -1.243615 | 1.952499 |
| C | 0.501161 | 2.412699 | -1.989190 |
| C | 1.773292 | 3.197200 | -2.425552 |
| H | 0.071427 | 1.853137 | -2.839857 |
| C | -0.587142 | 3.261540 | -1.361604 |
| H | 1.944751 | 3.183096 | -3.512730 |
| C | -0.349296 | 3.922834 | -0.142103 |
| C | -1.846966 | 3.375407 | -1.969809 |
| C | -1.353018 | 4.694749 | 0.453718 |
| H | 0.622136 | 3.810987 | 0.352851 |
| C | -2.852991 | 4.150350 | -1.374672 |

| | | | |
|---|---|---|---|
| H | -2.053653 | 2.825650 | -2.894639 |
| C | -2.608272 | 4.811682 | -0.164478 |
| H | -1.161019 | 5.196687 | 1.407800 |
| H | -3.837024 | 4.217995 | -1.848856 |
| H | -3.398942 | 5.405888 | 0.305497 |
| C | 0.549831 | -1.060657 | -1.945494 |
| Co | -0.006554 | -0.176371 | -0.327430 |
| C | 3.244839 | -2.120489 | 2.444811 |
| C | 7.078183 | 0.558139 | 0.396573 |
| C | 7.791908 | -0.740369 | -0.054324 |
| H | 8.884704 | -0.582402 | -0.105436 |
| H | 7.602474 | -1.570646 | 0.647911 |
| H | 7.438486 | -1.052671 | -1.052499 |
| C | 7.599003 | 0.971840 | 1.794985 |
| H | 7.405581 | 0.187975 | 2.547627 |
| H | 8.689611 | 1.148645 | 1.763376 |
| H | 7.106299 | 1.899186 | 2.135929 |
| C | 7.423518 | 1.677590 | -0.604078 |
| H | 7.092689 | 1.423812 | -1.626280 |
| H | 6.956002 | 2.635986 | -0.317891 |
| H | 8.516800 | 1.830067 | -0.630965 |
| C | 2.578820 | -3.255306 | 1.629972 |
| H | 1.739815 | -2.859237 | 1.042777 |
| H | 3.307143 | -3.712834 | 0.937349 |
| H | 2.198874 | -4.043965 | 2.304550 |
| C | 4.421078 | -2.731189 | 3.233558 |
| H | 5.163978 | -3.204152 | 2.567385 |
| H | 4.938447 | -1.976612 | 3.852412 |
| H | 4.034719 | -3.513279 | 3.910237 |
| C | 2.239258 | -1.546058 | 3.474784 |
| H | 2.710364 | -0.740990 | 4.066929 |
| H | 1.351965 | -1.138629 | 2.969667 |
| H | 1.917156 | -2.341372 | 4.171361 |
| H | 1.772227 | 4.238389 | -2.062168 |
| C | -1.942517 | -3.132753 | -0.898293 |
| N | -2.607591 | -2.095318 | 1.020230 |
| C | -0.796873 | -2.962448 | -0.037630 |
| C | -1.222846 | -2.327246 | 1.105440 |
| C | -3.069279 | -2.573318 | -0.226048 |
| C | -2.089790 | -3.662421 | -2.193129 |
| C | -3.331855 | -1.478067 | 2.038624 |
| C | -4.338102 | -2.532948 | -0.822720 |
| C | -3.351521 | -3.624960 | -2.791525 |
| H | -1.223365 | -4.069968 | -2.722186 |
| N | -4.645971 | -1.277298 | 1.839497 |
| N | -2.628753 | -1.149870 | 3.142952 |
| C | -4.455386 | -3.066730 | -2.112724 |
| H | -5.183357 | -2.091249 | -0.297199 |

| | | | |
|---|---|---|---|
| H | -3.484753 | -4.017365 | -3.804747 |
| C | -5.307329 | -0.673478 | 2.833880 |
| C | -3.314729 | -0.555858 | 4.122917 |
| H | -5.431337 | -3.039311 | -2.608660 |
| C | -4.686634 | -0.281437 | 4.026592 |
| H | -6.378322 | -0.499951 | 2.666010 |
| H | -2.741985 | -0.285610 | 5.019920 |
| H | -5.238087 | 0.207460 | 4.833938 |
| H | 0.215791 | -3.301937 | -0.242108 |
| H | -0.700855 | -2.055288 | 2.018921 |

**I-2$^5$**

Lowest frequency = 11.3705 cm$^{-1}$

Charge = 0, Multiplicity = 5

108

| | | | |
|---|---|---|---|
| C | 1.346723 | 3.166270 | -0.817940 |
| C | -0.730752 | 3.749394 | 0.259953 |
| C | 1.591445 | 4.514031 | -1.141117 |
| C | -0.497245 | 5.101345 | -0.052825 |
| H | -1.645883 | 3.477801 | 0.803452 |
| C | 0.662038 | 5.485478 | -0.753884 |
| H | 2.512809 | 4.772579 | -1.674839 |
| H | -1.224819 | 5.864953 | 0.250078 |
| H | 0.835184 | 6.541681 | -0.988542 |
| N | 1.898015 | 0.844330 | -0.750747 |
| C | 1.934662 | -1.508970 | -0.594354 |
| C | -0.077021 | -2.435236 | 0.125683 |
| C | 2.523632 | -2.795665 | -0.801688 |
| C | 2.652921 | -0.294048 | -0.892085 |
| C | 0.396283 | -3.746044 | -0.110144 |
| H | -1.081858 | -2.251830 | 0.519330 |
| C | 1.697569 | -3.925595 | -0.557106 |
| C | 3.876542 | -2.863691 | -1.239177 |
| C | 3.998348 | -0.411310 | -1.288830 |
| H | -0.260227 | -4.598843 | 0.086257 |
| H | 2.100717 | -4.930409 | -0.724187 |
| C | 4.585495 | -1.686879 | -1.448735 |
| H | 4.340335 | -3.842090 | -1.400351 |
| H | 4.558680 | 0.498152 | -1.501647 |
| H | 5.631743 | -1.739735 | -1.769431 |
| N | 0.671121 | -1.359842 | -0.110005 |
| C | 2.336138 | 2.109817 | -1.168837 |
| O | 3.386946 | 2.324469 | -1.783002 |
| O | -1.581652 | 0.276317 | 0.976192 |
| C | -2.837283 | 0.064807 | 0.670009 |

| | | | |
|---|---|---|---|
| C | -3.309975 | 0.135610 | -0.686085 |
| C | -3.796817 | -0.242586 | 1.703467 |
| C | -4.673681 | -0.081243 | -0.998385 |
| C | -2.392210 | 0.441924 | -1.761423 |
| C | -5.123751 | -0.450249 | 1.328299 |
| C | -5.602192 | -0.379618 | -0.006420 |
| H | -4.966658 | -0.006362 | -2.046874 |
| N | -1.099133 | 0.603969 | -1.677320 |
| O | -2.907865 | 0.569429 | -3.013372 |
| H | -5.847731 | -0.680533 | 2.114103 |
| C | -0.544839 | 0.827729 | -3.025463 |
| C | -1.829423 | 1.010601 | -3.880460 |
| H | 0.059401 | 1.749861 | -3.010383 |
| C | 0.330538 | -0.341781 | -3.441813 |
| H | -2.016250 | 2.065780 | -4.141892 |
| C | -0.166349 | -1.657027 | -3.364272 |
| C | 1.641501 | -0.129418 | -3.895256 |
| C | 0.636401 | -2.741270 | -3.735756 |
| H | -1.182388 | -1.833820 | -2.993828 |
| C | 2.447070 | -1.215751 | -4.266275 |
| H | 2.051045 | 0.886083 | -3.920837 |
| C | 1.947706 | -2.522021 | -4.187200 |
| H | 0.243434 | -3.760591 | -3.661317 |
| H | 3.475350 | -1.038826 | -4.596562 |
| H | 2.582682 | -3.369957 | -4.464095 |
| C | 0.190896 | 2.758435 | -0.121052 |
| Co | 0.023180 | 0.701826 | -0.005460 |
| C | -3.352240 | -0.327145 | 3.174699 |
| C | -7.091883 | -0.625998 | -0.293733 |
| C | -7.940454 | 0.417398 | 0.474348 |
| H | -9.017020 | 0.250841 | 0.287683 |
| H | -7.772313 | 0.353736 | 1.563341 |
| H | -7.685602 | 1.441557 | 0.150654 |
| C | -7.476192 | -2.049772 | 0.179278 |
| H | -7.293885 | -2.180650 | 1.260008 |
| H | -8.548247 | -2.241908 | -0.008760 |
| H | -6.886881 | -2.812204 | -0.359678 |
| C | -7.413597 | -0.506890 | -1.795693 |
| H | -7.179009 | 0.500286 | -2.182079 |
| H | -6.849102 | -1.245551 | -2.391298 |
| H | -8.489543 | -0.690151 | -1.962528 |
| C | -2.762101 | 1.036093 | 3.616013 |
| H | -1.919338 | 1.322643 | 2.971012 |
| H | -3.532794 | 1.824774 | 3.556997 |
| H | -2.407212 | 0.976964 | 4.660958 |
| C | -4.527222 | -0.658521 | 4.116810 |
| H | -5.315675 | 0.113734 | 4.078581 |
| H | -4.984259 | -1.635478 | 3.878664 |

| | | | |
|---|---|---|---|
| H | -4.156006 | -0.706649 | 5.155384 |
| C | -2.293199 | -1.447000 | 3.331340 |
| H | -2.726222 | -2.427611 | 3.064393 |
| H | -1.428259 | -1.257992 | 2.680274 |
| H | -1.942271 | -1.498881 | 4.377808 |
| H | -1.845365 | 0.388322 | -4.787553 |
| C | 2.372350 | 2.106834 | 2.197067 |
| N | 2.566733 | -0.163056 | 2.310507 |
| C | 1.075309 | 1.540235 | 2.495536 |
| C | 1.225320 | 0.174254 | 2.558343 |
| C | 3.291842 | 1.025142 | 2.065542 |
| C | 2.806399 | 3.425928 | 1.977403 |
| C | 3.022458 | -1.479731 | 2.309765 |
| C | 4.633564 | 1.235122 | 1.714524 |
| C | 4.142889 | 3.641511 | 1.631433 |
| H | 2.097967 | 4.256782 | 2.045362 |
| N | 4.314270 | -1.695904 | 2.006472 |
| N | 2.105651 | -2.418564 | 2.627592 |
| C | 5.038398 | 2.559257 | 1.500819 |
| H | 5.315730 | 0.393553 | 1.604010 |
| H | 4.495907 | 4.659564 | 1.438475 |
| C | 4.713695 | -2.972994 | 2.003909 |
| C | 2.532589 | -3.684026 | 2.623217 |
| H | 6.077021 | 2.754248 | 1.213872 |
| C | 3.853703 | -4.035354 | 2.310616 |
| H | 5.766328 | -3.149200 | 1.746547 |
| H | 1.785633 | -4.447757 | 2.877373 |
| H | 4.192217 | -5.074742 | 2.308303 |
| H | 0.158523 | 2.090452 | 2.696655 |
| H | 0.526224 | -0.618424 | 2.808100 |

**TS(2-3)[1]**
Lowest frequency = -261.2522 cm$^{-1}$
Charge = 0, Multiplicity = 1

108

| | | | |
|---|---|---|---|
| C | 0.578854 | -1.204256 | 2.793182 |
| C | -1.543470 | -2.099745 | 2.001361 |
| C | 0.148471 | -1.336454 | 4.118132 |
| C | -1.982942 | -2.194276 | 3.326958 |
| H | -2.199119 | -2.422669 | 1.185302 |
| C | -1.136093 | -1.829720 | 4.390762 |
| H | 0.828387 | -1.015806 | 4.915147 |
| H | -2.991592 | -2.568132 | 3.534310 |
| H | -1.482115 | -1.927319 | 5.425176 |
| N | 1.785227 | -0.052110 | 1.165648 |

| | | | |
|---|---|---|---|
| C | 2.241644 | 1.304573 | -0.682821 |
| C | 0.377663 | 1.559244 | -2.077654 |
| C | 3.049096 | 2.161241 | -1.489688 |
| C | 2.741112 | 0.699662 | 0.514372 |
| C | 1.093246 | 2.454279 | -2.905104 |
| H | -0.652657 | 1.260033 | -2.285422 |
| C | 2.420735 | 2.745157 | -2.625020 |
| C | 4.400391 | 2.367726 | -1.094179 |
| C | 4.078205 | 0.920999 | 0.871908 |
| H | 0.589584 | 2.891434 | -3.772123 |
| H | 2.994243 | 3.420572 | -3.268763 |
| C | 4.885112 | 1.745143 | 0.052296 |
| H | 5.041455 | 3.019511 | -1.695812 |
| H | 4.463953 | 0.464548 | 1.783700 |
| H | 5.929039 | 1.903735 | 0.344627 |
| N | 0.940571 | 1.009962 | -1.003054 |
| C | 1.862804 | -0.516150 | 2.459387 |
| O | 2.794559 | -0.325466 | 3.252119 |
| O | -1.362406 | -0.637753 | -0.895070 |
| C | -2.620889 | -0.322978 | -0.710961 |
| C | -3.074772 | 0.558758 | 0.324124 |
| C | -3.615575 | -0.881698 | -1.597989 |
| C | -4.452959 | 0.848100 | 0.493446 |
| C | -2.126444 | 1.201440 | 1.205257 |
| C | -4.953263 | -0.553214 | -1.379831 |
| C | -5.415669 | 0.300834 | -0.344546 |
| H | -4.724881 | 1.521341 | 1.307887 |
| N | -0.830830 | 1.046949 | 1.238466 |
| O | -2.624203 | 2.093741 | 2.103837 |
| H | -5.701025 | -0.987025 | -2.048709 |
| C | -0.259084 | 1.955150 | 2.253437 |
| C | -1.529284 | 2.493321 | 2.963674 |
| H | 0.348717 | 1.372663 | 2.962121 |
| C | 0.618648 | 3.018506 | 1.620013 |
| H | -1.684583 | 2.026792 | 3.951418 |
| C | 0.144780 | 3.785581 | 0.539922 |
| C | 1.920701 | 3.236471 | 2.098946 |
| C | 0.962902 | 4.754650 | -0.052991 |
| H | -0.863071 | 3.606611 | 0.148783 |
| C | 2.740262 | 4.208293 | 1.507305 |
| H | 2.305830 | 2.614015 | 2.914247 |
| C | 2.263682 | 4.967468 | 0.430484 |
| H | 0.589504 | 5.338366 | -0.900944 |
| H | 3.760412 | 4.355483 | 1.875508 |
| H | 2.908527 | 5.716881 | -0.040651 |
| C | -0.254332 | -1.603969 | 1.708128 |
| Co | 0.175667 | -0.300267 | 0.191363 |
| C | -3.201687 | -1.831976 | -2.739381 |

| | | | |
|---|---|---|---|
| C | -6.920973 | 0.577100 | -0.199226 |
| C | -7.665912 | -0.757360 | 0.050481 |
| H | -8.753302 | -0.582579 | 0.146451 |
| H | -7.509679 | -1.468606 | -0.779132 |
| H | -7.307226 | -1.235400 | 0.978866 |
| C | -7.453805 | 1.226043 | -1.500313 |
| H | -7.293163 | 0.570931 | -2.374129 |
| H | -8.538778 | 1.422131 | -1.419475 |
| H | -6.940900 | 2.184028 | -1.695806 |
| C | -7.217218 | 1.527969 | 0.976202 |
| H | -6.876445 | 1.103162 | 1.936617 |
| H | -6.725883 | 2.507097 | 0.838895 |
| H | -8.305024 | 1.703034 | 1.051051 |
| C | -2.546321 | -3.103432 | -2.144499 |
| H | -1.660904 | -2.837202 | -1.550228 |
| H | -3.261818 | -3.638740 | -1.495075 |
| H | -2.234844 | -3.789108 | -2.953604 |
| C | -4.413665 | -2.282651 | -3.580798 |
| H | -5.152510 | -2.838155 | -2.976577 |
| H | -4.925690 | -1.426823 | -4.055400 |
| H | -4.066619 | -2.954967 | -4.384921 |
| C | -2.208462 | -1.125114 | -3.696690 |
| H | -2.668778 | -0.216932 | -4.125959 |
| H | -1.285993 | -0.838741 | -3.171420 |
| H | -1.942081 | -1.800729 | -4.530105 |
| H | -1.548738 | 3.589484 | 3.056925 |
| C | 1.814837 | -3.062142 | 0.877998 |
| N | 2.399452 | -1.860929 | -0.984751 |
| C | 0.546272 | -2.515329 | 0.316841 |
| C | 0.977998 | -1.762873 | -0.873076 |
| C | 2.906029 | -2.586095 | 0.111298 |
| C | 2.034459 | -3.849458 | 2.012467 |
| C | 3.087468 | -1.295883 | -2.045540 |
| C | 4.231659 | -2.859289 | 0.486392 |
| C | 3.351699 | -4.157545 | 2.375832 |
| H | 1.188175 | -4.192304 | 2.616000 |
| N | 4.435245 | -1.273924 | -1.992484 |
| N | 2.319794 | -0.820987 | -3.053603 |
| C | 4.429607 | -3.654870 | 1.623156 |
| H | 5.058657 | -2.457120 | -0.097154 |
| H | 3.545073 | -4.773116 | 3.259887 |
| C | 5.054729 | -0.681826 | -3.019274 |
| C | 2.967714 | -0.240570 | -4.065992 |
| H | 5.454493 | -3.883376 | 1.934541 |
| C | 4.365204 | -0.123933 | -4.104046 |
| H | 6.151506 | -0.654074 | -2.969636 |
| H | 2.341965 | 0.147472 | -4.880809 |
| H | 4.885005 | 0.363436 | -4.932911 |

| | | | |
|---|---|---|---|
| H | -0.293911 | -3.200888 | 0.165462 |
| H | 0.435005 | -1.782057 | -1.818455 |

**TS(2-3)$^3$**
Lowest frequency = -400.6485 cm$^{-1}$
Charge = 0, Multiplicity = 3

108

| | | | |
|---|---|---|---|
| C | 0.465066 | 1.364668 | 2.699553 |
| C | -1.703662 | 0.252567 | 2.708623 |
| C | 0.022421 | 2.242728 | 3.697491 |
| C | -2.151683 | 1.163950 | 3.676735 |
| H | -2.378296 | -0.534840 | 2.355081 |
| C | -1.288884 | 2.149765 | 4.188162 |
| H | 0.721669 | 3.010539 | 4.047521 |
| H | -3.180018 | 1.088290 | 4.047958 |
| H | -1.641045 | 2.841638 | 4.960495 |
| N | 1.791866 | 0.896119 | 0.834876 |
| C | 2.477598 | 0.297418 | -1.330399 |
| C | 0.752703 | -0.578173 | -2.646211 |
| C | 3.420546 | 0.212388 | -2.403196 |
| C | 2.844202 | 0.829651 | -0.042831 |
| C | 1.601740 | -0.645081 | -3.773785 |
| H | -0.289781 | -0.909184 | -2.679973 |
| C | 2.930931 | -0.267493 | -3.649664 |
| C | 4.761433 | 0.618181 | -2.156561 |
| C | 4.180906 | 1.209682 | 0.159050 |
| H | 1.203728 | -1.015960 | -4.722888 |
| H | 3.614165 | -0.333238 | -4.503397 |
| C | 5.114363 | 1.089673 | -0.895993 |
| H | 5.497477 | 0.558410 | -2.964616 |
| H | 4.466213 | 1.613960 | 1.130806 |
| H | 6.150486 | 1.394522 | -0.710564 |
| N | 1.183217 | -0.118257 | -1.473730 |
| C | 1.803119 | 1.544568 | 2.048114 |
| O | 2.719050 | 2.232997 | 2.520248 |
| O | -1.463471 | -1.060301 | -0.323313 |
| C | -2.723269 | -0.722874 | -0.388779 |
| C | -3.160035 | 0.647870 | -0.354745 |
| C | -3.737274 | -1.747041 | -0.511419 |
| C | -4.536116 | 0.980856 | -0.411199 |
| C | -2.196387 | 1.723390 | -0.300514 |
| C | -5.072664 | -1.351746 | -0.560261 |
| C | -5.513811 | -0.002378 | -0.506046 |
| H | -4.797722 | 2.039501 | -0.378176 |
| N | -0.893597 | 1.626425 | -0.225687 |

| | | | |
|---|---|---|---|
| O | -2.680344 | 2.991604 | -0.341371 |
| H | -5.835814 | -2.129675 | -0.644047 |
| C | -0.305709 | 2.978856 | -0.174463 |
| C | -1.549022 | 3.897964 | -0.331386 |
| H | 0.149514 | 3.110357 | 0.822134 |
| C | 0.763943 | 3.182487 | -1.228161 |
| H | -1.680520 | 4.598608 | 0.507604 |
| C | 0.511070 | 2.846925 | -2.570933 |
| C | 2.022296 | 3.694218 | -0.873410 |
| C | 1.504068 | 3.014826 | -3.542880 |
| H | -0.460946 | 2.423576 | -2.848466 |
| C | 3.016257 | 3.867770 | -1.847095 |
| H | 2.240511 | 3.914477 | 0.177445 |
| C | 2.760684 | 3.526445 | -3.181494 |
| H | 1.302920 | 2.734227 | -4.582040 |
| H | 4.000719 | 4.246324 | -1.555031 |
| H | 3.543286 | 3.645944 | -3.938071 |
| C | -0.388131 | 0.338806 | 2.208892 |
| Co | 0.105476 | -0.022540 | 0.239085 |
| C | -3.339407 | -3.234112 | -0.568211 |
| C | -7.018432 | 0.306798 | -0.555803 |
| C | -7.718960 | -0.371861 | 0.647333 |
| H | -8.805508 | -0.170142 | 0.625291 |
| H | -7.577738 | -1.466496 | 0.633137 |
| H | -7.313603 | 0.010524 | 1.600441 |
| C | -7.613453 | -0.245052 | -1.874848 |
| H | -7.470668 | -1.336332 | -1.958624 |
| H | -8.698736 | -0.041223 | -1.923105 |
| H | -7.131615 | 0.228616 | -2.747974 |
| C | -7.295418 | 1.820947 | -0.491291 |
| H | -6.909335 | 2.264249 | 0.443186 |
| H | -6.835368 | 2.353801 | -1.341743 |
| H | -8.383857 | 2.003277 | -0.527292 |
| C | -2.635510 | -3.631264 | 0.753263 |
| H | -1.735301 | -3.020706 | 0.908113 |
| H | -3.314588 | -3.485996 | 1.612298 |
| H | -2.338532 | -4.695465 | 0.724240 |
| C | -4.566859 | -4.152537 | -0.739076 |
| H | -5.271638 | -4.062019 | 0.106351 |
| H | -5.114754 | -3.936940 | -1.673501 |
| H | -4.230002 | -5.203029 | -0.781632 |
| C | -2.393017 | -3.489047 | -1.769362 |
| H | -2.892891 | -3.218759 | -2.716813 |
| H | -1.469720 | -2.899246 | -1.675819 |
| H | -2.124933 | -4.560401 | -1.816224 |
| H | -1.548285 | 4.453050 | -1.284075 |
| C | 1.616856 | -1.407166 | 2.916539 |
| N | 2.289399 | -1.996794 | 0.806245 |

| | | | |
|---|---|---|---|
| C | 0.407001 | -1.506501 | 2.088491 |
| C | 0.874523 | -1.863264 | 0.756428 |
| C | 2.748069 | -1.631115 | 2.090752 |
| C | 1.780763 | -1.043696 | 4.259940 |
| C | 3.031733 | -2.452077 | -0.274955 |
| C | 4.052765 | -1.473474 | 2.582841 |
| C | 3.078285 | -0.910441 | 4.766747 |
| H | 0.905637 | -0.842900 | 4.885431 |
| N | 4.374571 | -2.379574 | -0.193872 |
| N | 2.317906 | -2.947690 | -1.310611 |
| C | 4.192992 | -1.115231 | 3.930879 |
| H | 4.908653 | -1.622887 | 1.926641 |
| H | 3.229319 | -0.623690 | 5.812189 |
| C | 5.053967 | -2.802912 | -1.265314 |
| C | 3.023537 | -3.364302 | -2.365018 |
| H | 5.200772 | -0.983641 | 4.338843 |
| C | 4.424312 | -3.308457 | -2.410275 |
| H | 6.147929 | -2.733367 | -1.201693 |
| H | 2.443205 | -3.760378 | -3.209006 |
| H | 4.990135 | -3.645315 | -3.282703 |
| H | -0.513167 | -1.955891 | 2.466362 |
| H | 0.352113 | -2.530221 | 0.070001 |

**TS(2-3)[5]**
Lowest frequency = -368.1683 $cm^{-1}$
Charge = 0, Multiplicity = 5

108

| | | | |
|---|---|---|---|
| C | -0.121476 | 0.037394 | 2.799853 |
| C | 1.877749 | 1.289310 | 2.173985 |
| C | 0.568423 | -0.519263 | 3.886705 |
| C | 2.582393 | 0.671985 | 3.220901 |
| H | 2.388777 | 2.021535 | 1.538253 |
| C | 1.924016 | -0.213953 | 4.090264 |
| H | 0.029883 | -1.216075 | 4.539581 |
| H | 3.643996 | 0.900832 | 3.368065 |
| H | 2.469643 | -0.668021 | 4.924482 |
| N | -1.637887 | -0.399914 | 1.070812 |
| C | -2.589247 | -0.805790 | -1.047037 |
| C | -1.098639 | -0.525226 | -2.824012 |
| C | -3.655118 | -1.186541 | -1.922713 |
| C | -2.774650 | -0.751564 | 0.381465 |
| C | -2.081486 | -0.921039 | -3.759686 |
| H | -0.086151 | -0.243930 | -3.133316 |
| C | -3.354743 | -1.242624 | -3.311502 |
| C | -4.926780 | -1.484864 | -1.358133 |

| | | | |
|---|---|---|---|
| C | -4.048294 | -1.044362 | 0.894294 |
| H | -1.828534 | -0.957677 | -4.823506 |
| H | -4.138373 | -1.540452 | -4.016854 |
| C | -5.100425 | -1.399458 | 0.018572 |
| H | -5.750575 | -1.779339 | -2.016268 |
| H | -4.193837 | -1.008695 | 1.974317 |
| H | -6.082703 | -1.623300 | 0.449676 |
| N | -1.347950 | -0.474152 | -1.517125 |
| C | -1.497993 | -0.453248 | 2.441489 |
| O | -2.305170 | -0.905535 | 3.263195 |
| O | 1.499130 | 0.633558 | -1.005607 |
| C | 2.774260 | 0.384411 | -0.827354 |
| C | 3.242076 | -0.804781 | -0.162219 |
| C | 3.764884 | 1.305836 | -1.328761 |
| C | 4.628435 | -1.047018 | 0.009457 |
| C | 2.314305 | -1.799712 | 0.332340 |
| C | 5.111285 | 1.012472 | -1.115768 |
| C | 5.583617 | -0.146878 | -0.446188 |
| H | 4.915452 | -1.966618 | 0.521965 |
| N | 1.008011 | -1.723739 | 0.372099 |
| O | 2.831035 | -2.957717 | 0.824969 |
| H | 5.855995 | 1.721917 | -1.485868 |
| C | 0.462324 | -2.934033 | 1.008392 |
| C | 1.715888 | -3.841988 | 1.113332 |
| H | 0.099626 | -2.654165 | 2.013986 |
| C | -0.696908 | -3.500356 | 0.214957 |
| H | 1.869384 | -4.270187 | 2.114852 |
| C | -0.574262 | -3.699436 | -1.173040 |
| C | -1.917963 | -3.788250 | 0.844619 |
| C | -1.655714 | -4.182986 | -1.918035 |
| H | 0.365527 | -3.441769 | -1.674364 |
| C | -3.000653 | -4.277651 | 0.099923 |
| H | -2.036208 | -3.583365 | 1.914340 |
| C | -2.872459 | -4.474906 | -1.280939 |
| H | -1.555584 | -4.318281 | -3.000029 |
| H | -3.954550 | -4.478124 | 0.597431 |
| H | -3.724335 | -4.839626 | -1.864253 |
| C | 0.535654 | 0.950679 | 1.936972 |
| Co | 0.010541 | -0.045046 | -0.006205 |
| C | 3.329198 | 2.586833 | -2.066282 |
| C | 7.095039 | -0.352113 | -0.254728 |
| C | 7.667683 | 0.823959 | 0.574405 |
| H | 8.757216 | 0.703098 | 0.716664 |
| H | 7.497101 | 1.792649 | 0.073450 |
| H | 7.190019 | 0.867642 | 1.569007 |
| C | 7.790063 | -0.392481 | -1.637844 |
| H | 7.626225 | 0.542474 | -2.201153 |
| H | 8.880491 | -0.528620 | -1.518289 |

| | | | |
|---|---|---|---|
| H | 7.399945 | -1.227629 | -2.245479 |
| C | 7.405530 | -1.667028 | 0.485651 |
| H | 6.950955 | -1.682651 | 1.491675 |
| H | 7.035545 | -2.544107 | -0.073571 |
| H | 8.497395 | -1.779183 | 0.606532 |
| C | 2.494878 | 3.475482 | -1.111122 |
| H | 1.620325 | 2.922652 | -0.741322 |
| H | 3.105512 | 3.794530 | -0.247486 |
| H | 2.144775 | 4.382274 | -1.637176 |
| C | 4.537461 | 3.416246 | -2.546371 |
| H | 5.168737 | 3.751536 | -1.704435 |
| H | 5.170842 | 2.849679 | -3.251799 |
| H | 4.173750 | 4.317462 | -3.070361 |
| C | 2.485256 | 2.219091 | -3.313288 |
| H | 3.074642 | 1.592442 | -4.006265 |
| H | 1.579714 | 1.667829 | -3.020285 |
| H | 2.185490 | 3.137947 | -3.849931 |
| H | 1.714939 | -4.647725 | 0.358707 |
| C | -1.671546 | 2.634698 | 2.177342 |
| N | -2.524567 | 2.342751 | 0.074003 |
| C | -0.547411 | 2.621374 | 1.231681 |
| C | -1.146172 | 2.519014 | -0.052889 |
| C | -2.866472 | 2.388065 | 1.450575 |
| C | -1.710865 | 2.736352 | 3.575615 |
| C | -3.372968 | 2.178444 | -1.019879 |
| C | -4.100349 | 2.221370 | 2.094453 |
| C | -2.940151 | 2.588997 | 4.226806 |
| H | -0.788362 | 2.898392 | 4.141456 |
| N | -4.679517 | 1.971537 | -0.776021 |
| N | -2.783492 | 2.260722 | -2.234043 |
| C | -4.112771 | 2.326601 | 3.492061 |
| H | -4.999199 | 2.012144 | 1.516918 |
| H | -2.989426 | 2.656681 | 5.318187 |
| C | -5.460098 | 1.805525 | -1.848935 |
| C | -3.588652 | 2.099831 | -3.287319 |
| H | -5.061123 | 2.193428 | 4.023033 |
| C | -4.964900 | 1.860124 | -3.159583 |
| H | -6.524169 | 1.624260 | -1.649381 |
| H | -3.112482 | 2.162479 | -4.274722 |
| H | -5.612028 | 1.723974 | -4.029866 |
| H | 0.366363 | 3.201353 | 1.376091 |
| H | -0.714889 | 2.594429 | -1.048555 |

**I-3[1]**

Lowest frequency = 10.1297 $cm^{-1}$

Charge = 0, Multiplicity = 1

108

| | | | |
|---|---|---|---|
| C | -0.511022 | 0.439249 | -2.957304 |
| C | 1.432812 | -1.028730 | -2.859019 |
| C | 0.224753 | 1.334723 | -3.748337 |
| C | 2.184970 | -0.085500 | -3.565477 |
| H | 1.896424 | -1.968538 | -2.542355 |
| C | 1.570597 | 1.083883 | -4.043654 |
| H | -0.270110 | 2.252046 | -4.085043 |
| H | 3.244156 | -0.274336 | -3.765998 |
| H | 2.146805 | 1.804169 | -4.634324 |
| N | -1.780674 | 0.664604 | -1.029847 |
| C | -2.270002 | 0.730025 | 1.241961 |
| C | -0.402802 | 0.149670 | 2.543285 |
| C | -3.078055 | 0.978796 | 2.389573 |
| C | -2.754908 | 0.921639 | -0.088867 |
| C | -1.121585 | 0.431170 | 3.725116 |
| H | 0.623209 | -0.222839 | 2.548608 |
| C | -2.452305 | 0.821080 | 3.657726 |
| C | -4.426195 | 1.379882 | 2.168941 |
| C | -4.087759 | 1.310727 | -0.271047 |
| H | -0.617564 | 0.306965 | 4.687801 |
| H | -3.027191 | 1.011743 | 4.569875 |
| C | -4.901810 | 1.521546 | 0.868181 |
| H | -5.073341 | 1.578380 | 3.028845 |
| H | -4.466208 | 1.456333 | -1.283555 |
| H | -5.943613 | 1.822575 | 0.712353 |
| N | -0.971014 | 0.292313 | 1.342958 |
| C | -1.829828 | 0.903764 | -2.376860 |
| O | -2.708528 | 1.503949 | -3.002803 |
| O | 1.343867 | -0.972997 | 0.362639 |
| C | 2.610308 | -0.642434 | 0.364268 |
| C | 3.098574 | 0.650881 | -0.021813 |
| C | 3.581461 | -1.628904 | 0.780489 |
| C | 4.486311 | 0.945692 | -0.012544 |
| C | 2.176681 | 1.695435 | -0.400842 |
| C | 4.929491 | -1.274424 | 0.765780 |
| C | 5.424651 | -0.002253 | 0.373316 |
| H | 4.785491 | 1.948889 | -0.320729 |
| N | 0.879106 | 1.603051 | -0.532129 |
| O | 2.695327 | 2.932583 | -0.631695 |
| H | 5.659630 | -2.027319 | 1.073590 |
| C | 0.329051 | 2.941729 | -0.823954 |
| C | 1.610233 | 3.776530 | -1.088112 |
| H | -0.286480 | 2.891643 | -1.735148 |
| C | -0.546201 | 3.441602 | 0.310291 |
| H | 1.764163 | 3.977912 | -2.162113 |
| C | -0.074626 | 3.438846 | 1.635756 |

| | | | |
|---|---|---|---|
| C | -1.848750 | 3.895608 | 0.046053 |
| C | -0.894657 | 3.884014 | 2.679633 |
| H | 0.932547 | 3.065541 | 1.852853 |
| C | -2.670441 | 4.343633 | 1.089949 |
| H | -2.231981 | 3.862619 | -0.979981 |
| C | -2.195265 | 4.337394 | 2.407894 |
| H | -0.523080 | 3.866583 | 3.709493 |
| H | -3.690648 | 4.675865 | 0.874134 |
| H | -2.841359 | 4.673397 | 3.225763 |
| C | 0.073833 | -0.799810 | -2.546744 |
| Co | -0.182078 | -0.057477 | -0.306360 |
| C | 3.128538 | -3.042447 | 1.197943 |
| C | 6.938505 | 0.264484 | 0.392263 |
| C | 7.647832 | -0.733407 | -0.556014 |
| H | 8.740408 | -0.565077 | -0.549053 |
| H | 7.465026 | -1.778973 | -0.252977 |
| H | 7.284082 | -0.611988 | -1.591478 |
| C | 7.476874 | 0.074981 | 1.831744 |
| H | 7.288633 | -0.947609 | 2.202302 |
| H | 8.567757 | 0.251237 | 1.863717 |
| H | 6.989783 | 0.782237 | 2.525644 |
| C | 7.273078 | 1.695958 | -0.068703 |
| H | 6.928611 | 1.879720 | -1.101517 |
| H | 6.809425 | 2.452108 | 0.588691 |
| H | 8.366132 | 1.851367 | -0.044388 |
| C | 2.456480 | -3.746267 | -0.008083 |
| H | 1.588011 | -3.168604 | -0.355964 |
| H | 3.173199 | -3.850758 | -0.842314 |
| H | 2.113632 | -4.756661 | 0.280526 |
| C | 4.319071 | -3.916216 | 1.644592 |
| H | 5.055696 | -4.058156 | 0.834095 |
| H | 4.841115 | -3.484057 | 2.516796 |
| H | 3.946981 | -4.914088 | 1.935618 |
| C | 2.132464 | -2.971122 | 2.384361 |
| H | 2.590139 | -2.440929 | 3.239229 |
| H | 1.205690 | -2.454234 | 2.097483 |
| H | 1.872741 | -3.993224 | 2.715998 |
| H | 1.646628 | 4.718701 | -0.520669 |
| C | -2.190794 | -1.978363 | -2.526259 |
| N | -2.427903 | -2.018365 | -0.241387 |
| C | -0.782932 | -1.961305 | -1.971408 |
| C | -1.004246 | -1.840782 | -0.456941 |
| C | -3.125337 | -1.984374 | -1.469498 |
| C | -2.615592 | -1.933546 | -3.853751 |
| C | -2.952041 | -2.224193 | 1.017280 |
| C | -4.504753 | -1.933860 | -1.728456 |
| C | -3.992732 | -1.898893 | -4.127145 |
| H | -1.882405 | -1.903614 | -4.666650 |

| | | | |
|---|---|---|---|
| N | -4.297063 | -2.225252 | 1.160381 |
| N | -2.050317 | -2.416769 | 2.011856 |
| C | -4.916245 | -1.896716 | -3.070594 |
| H | -5.213882 | -1.921282 | -0.902584 |
| H | -4.344327 | -1.858130 | -5.162894 |
| C | -4.762041 | -2.402914 | 2.399841 |
| C | -2.548339 | -2.592159 | 3.237000 |
| H | -5.988373 | -1.854026 | -3.291222 |
| C | -3.924824 | -2.587867 | 3.508569 |
| H | -5.855142 | -2.395157 | 2.510439 |
| H | -1.812494 | -2.741929 | 4.039067 |
| H | -4.318831 | -2.725640 | 4.518816 |
| H | -0.240432 | -2.893824 | -2.217886 |
| H | -0.415716 | -2.491245 | 0.198578 |

**I-3[3]**
Lowest frequency = 10.7511 cm$^{-1}$
Charge = 0, Multiplicity = 3

108

| | | | |
|---|---|---|---|
| C | 0.131682 | 1.066178 | -2.601238 |
| C | 1.957072 | -0.532392 | -2.754458 |
| C | 1.014489 | 2.082536 | -3.002756 |
| C | 2.848451 | 0.502953 | -3.067073 |
| H | 2.322946 | -1.562274 | -2.686981 |
| C | 2.372461 | 1.812786 | -3.216551 |
| H | 0.615335 | 3.096310 | -3.114778 |
| H | 3.910923 | 0.280556 | -3.208622 |
| H | 3.057717 | 2.621949 | -3.489678 |
| N | -1.560233 | 0.897783 | -0.956520 |
| C | -2.750793 | 0.469447 | 1.041606 |
| C | -1.434747 | -0.396089 | 2.765972 |
| C | -3.928615 | 0.461705 | 1.853365 |
| C | -2.782811 | 0.948178 | -0.314609 |
| C | -2.539609 | -0.416487 | 3.647483 |
| H | -0.444155 | -0.748498 | 3.072072 |
| C | -3.781967 | -0.001517 | 3.190287 |
| C | -5.148581 | 0.921910 | 1.282929 |
| C | -4.004564 | 1.386013 | -0.840907 |
| H | -2.404433 | -0.775590 | 4.671983 |
| H | -4.657443 | -0.024111 | 3.848379 |
| C | -5.167379 | 1.363326 | -0.034440 |
| H | -6.058161 | 0.924810 | 1.892203 |
| H | -4.033224 | 1.754955 | -1.866506 |
| H | -6.109767 | 1.712795 | -0.470452 |
| N | -1.544734 | 0.028876 | 1.510857 |

| | | | |
|---|---|---|---|
| C | -1.263166 | 1.468487 | -2.171488 |
| O | -1.954959 | 2.276378 | -2.801209 |
| O | 1.355899 | -0.923553 | 0.713317 |
| C | 2.628437 | -0.612966 | 0.642769 |
| C | 3.092855 | 0.735329 | 0.462404 |
| C | 3.622868 | -1.649331 | 0.790728 |
| C | 4.475154 | 1.033077 | 0.378099 |
| C | 2.151620 | 1.827649 | 0.398693 |
| C | 4.966666 | -1.290917 | 0.689196 |
| C | 5.433901 | 0.031850 | 0.468939 |
| H | 4.754926 | 2.077680 | 0.232600 |
| N | 0.845175 | 1.745324 | 0.357771 |
| O | 2.649214 | 3.092597 | 0.386950 |
| H | 5.715298 | -2.081946 | 0.779892 |
| C | 0.283388 | 3.106103 | 0.243527 |
| C | 1.520904 | 3.995564 | 0.517185 |
| H | -0.061948 | 3.243920 | -0.796124 |
| C | -0.896364 | 3.330461 | 1.163817 |
| H | 1.649284 | 4.812853 | -0.207555 |
| C | -0.812102 | 3.004441 | 2.529446 |
| C | -2.101615 | 3.837269 | 0.650729 |
| C | -1.918225 | 3.181952 | 3.368826 |
| H | 0.115714 | 2.577611 | 2.926719 |
| C | -3.208645 | 4.020283 | 1.491183 |
| H | -2.183923 | 4.046136 | -0.421610 |
| C | -3.119335 | 3.691112 | 2.850063 |
| H | -1.849504 | 2.908505 | 4.426856 |
| H | -4.149247 | 4.395609 | 1.076418 |
| H | -3.989114 | 3.816966 | 3.503383 |
| C | 0.593478 | -0.279068 | -2.515813 |
| Co | -0.128970 | 0.043916 | 0.023523 |
| C | 3.203298 | -3.116068 | 1.009987 |
| C | 6.942564 | 0.293814 | 0.338020 |
| C | 7.490498 | -0.500928 | -0.873305 |
| H | 8.577576 | -0.335343 | -0.985913 |
| H | 7.323296 | -1.585459 | -0.754152 |
| H | 6.992072 | -0.181306 | -1.805565 |
| C | 7.663770 | -0.170765 | 1.626968 |
| H | 7.504157 | -1.246453 | 1.815697 |
| H | 8.752594 | -0.000300 | 1.542615 |
| H | 7.291437 | 0.386763 | 2.504090 |
| C | 7.247544 | 1.787996 | 0.119210 |
| H | 6.776004 | 2.166671 | -0.804569 |
| H | 6.891715 | 2.402284 | 0.964707 |
| H | 8.337718 | 1.937691 | 0.027617 |
| C | 2.487814 | -3.630147 | -0.263543 |
| H | 1.619117 | -2.999650 | -0.499256 |
| H | 3.178152 | -3.616686 | -1.125704 |

| | | | |
|---|---|---|---|
| H | 2.136841 | -4.667824 | -0.116794 |
| C | 4.420565 | -4.029458 | 1.265588 |
| H | 5.111404 | -4.053404 | 0.404300 |
| H | 4.988657 | -3.714294 | 2.158850 |
| H | 4.068270 | -5.061768 | 1.435909 |
| C | 2.261509 | -3.243653 | 2.235590 |
| H | 2.757764 | -2.854650 | 3.142943 |
| H | 1.325263 | -2.690154 | 2.076224 |
| H | 2.020352 | -4.308838 | 2.407049 |
| H | 1.524458 | 4.402740 | 1.543619 |
| C | -1.704221 | -1.329759 | -2.960640 |
| N | -2.166719 | -1.992875 | -0.810291 |
| C | -0.359121 | -1.458899 | -2.273338 |
| C | -0.759158 | -1.670826 | -0.797523 |
| C | -2.739396 | -1.664156 | -2.062817 |
| C | -1.995943 | -0.935881 | -4.265694 |
| C | -2.776520 | -2.619027 | 0.258023 |
| C | -4.084703 | -1.611993 | -2.456957 |
| C | -3.338973 | -0.887716 | -4.677789 |
| H | -1.188956 | -0.652133 | -4.949022 |
| N | -4.119009 | -2.776595 | 0.227144 |
| N | -1.953260 | -3.034830 | 1.252334 |
| C | -4.361904 | -1.225314 | -3.779018 |
| H | -4.872110 | -1.859096 | -1.746846 |
| H | -3.586291 | -0.575437 | -5.697360 |
| C | -4.668324 | -3.397344 | 1.274723 |
| C | -2.533705 | -3.651181 | 2.283347 |
| H | -5.406462 | -1.174636 | -4.105069 |
| C | -3.917395 | -3.867924 | 2.361135 |
| H | -5.759899 | -3.519303 | 1.244815 |
| H | -1.861325 | -3.984111 | 3.086413 |
| H | -4.380436 | -4.370652 | 3.214176 |
| H | 0.162627 | -2.364019 | -2.645711 |
| H | -0.191444 | -2.405451 | -0.217201 |

**I-3[5]**
Lowest frequency = 11.4285 cm$^{-1}$
Charge = 0, Multiplicity = 5

108

| | | | |
|---|---|---|---|
| C | 0.328023 | 2.538407 | 1.741390 |
| C | -1.562547 | 1.702329 | 3.018172 |
| C | -0.390206 | 3.728773 | 1.517838 |
| C | -2.305254 | 2.846676 | 2.701886 |
| H | -2.009090 | 0.921450 | 3.644476 |
| C | -1.705371 | 3.880511 | 1.968957 |

| | | | |
|---|---|---|---|
| H | 0.109982 | 4.526735 | 0.958629 |
| H | -3.338341 | 2.941410 | 3.051999 |
| H | -2.259676 | 4.799736 | 1.751922 |
| N | 1.809376 | 1.210891 | 0.390278 |
| C | 2.842835 | -0.288556 | -1.133447 |
| C | 1.390550 | -1.871803 | -2.072068 |
| C | 3.963416 | -0.814541 | -1.852924 |
| C | 2.978851 | 0.834801 | -0.244735 |
| C | 2.438071 | -2.446229 | -2.823214 |
| H | 0.364920 | -2.251974 | -2.112699 |
| C | 3.718326 | -1.920508 | -2.711283 |
| C | 5.235831 | -0.204506 | -1.672202 |
| C | 4.255406 | 1.399470 | -0.093131 |
| H | 2.228692 | -3.299049 | -3.474995 |
| H | 4.550520 | -2.349109 | -3.280671 |
| C | 5.358873 | 0.872989 | -0.802490 |
| H | 6.100818 | -0.592914 | -2.219375 |
| H | 4.373838 | 2.255574 | 0.570752 |
| H | 6.340193 | 1.338933 | -0.659602 |
| N | 1.592927 | -0.832675 | -1.261798 |
| C | 1.662852 | 2.416127 | 1.037053 |
| O | 2.434716 | 3.384626 | 0.977006 |
| O | -1.357289 | -1.142587 | -0.381437 |
| C | -2.616615 | -0.792852 | -0.344413 |
| C | -3.049221 | 0.559287 | -0.603559 |
| C | -3.651699 | -1.772519 | -0.091885 |
| C | -4.414220 | 0.931184 | -0.541105 |
| C | -2.091119 | 1.543019 | -1.047291 |
| C | -4.974407 | -1.336882 | -0.021190 |
| C | -5.397653 | 0.004094 | -0.221100 |
| H | -4.660575 | 1.973853 | -0.750768 |
| N | -0.788133 | 1.467607 | -0.959506 |
| O | -2.560595 | 2.655190 | -1.669071 |
| H | -5.745697 | -2.079214 | 0.199721 |
| C | -0.179485 | 2.634492 | -1.625313 |
| C | -1.417412 | 3.371591 | -2.205155 |
| H | 0.308636 | 3.260990 | -0.863008 |
| C | 0.879633 | 2.213957 | -2.625029 |
| H | -1.488751 | 4.421363 | -1.882267 |
| C | 0.607945 | 1.221904 | -3.584708 |
| C | 2.165702 | 2.775964 | -2.561578 |
| C | 1.609652 | 0.796647 | -4.465608 |
| H | -0.382622 | 0.753626 | -3.613755 |
| C | 3.168087 | 2.354480 | -3.446641 |
| H | 2.394356 | 3.508875 | -1.779886 |
| C | 2.892783 | 1.362578 | -4.397356 |
| H | 1.394771 | 0.010963 | -5.197625 |
| H | 4.172872 | 2.781896 | -3.372003 |

| | | | |
|---|---|---|---|
| H | 3.680267 | 1.019346 | -5.076580 |
| C | -0.244786 | 1.528817 | 2.556474 |
| Co | 0.223134 | 0.011432 | -0.033611 |
| C | -3.296053 | -3.259070 | 0.099110 |
| C | -6.886886 | 0.360600 | -0.091508 |
| C | -7.367729 | 0.025855 | 1.342086 |
| H | -8.441195 | 0.264009 | 1.456510 |
| H | -7.231469 | -1.044708 | 1.573816 |
| H | -6.800491 | 0.608970 | 2.088721 |
| C | -7.707584 | -0.463499 | -1.113886 |
| H | -7.585018 | -1.548255 | -0.950963 |
| H | -8.783979 | -0.227682 | -1.025578 |
| H | -7.385139 | -0.236710 | -2.145136 |
| C | -7.141961 | 1.857095 | -0.354285 |
| H | -6.593265 | 2.492846 | 0.362660 |
| H | -6.837596 | 2.147138 | -1.375138 |
| H | -8.218639 | 2.077894 | -0.247002 |
| C | -2.396706 | -3.434227 | 1.346224 |
| H | -1.418234 | -2.957618 | 1.190124 |
| H | -2.882153 | -3.001783 | 2.239380 |
| H | -2.224398 | -4.509767 | 1.538996 |
| C | -4.553042 | -4.131980 | 0.293016 |
| H | -5.109519 | -3.856629 | 1.206483 |
| H | -5.241722 | -4.063438 | -0.567853 |
| H | -4.246783 | -5.187857 | 0.396007 |
| C | -2.548767 | -3.778997 | -1.155040 |
| H | -3.185244 | -3.690655 | -2.053473 |
| H | -1.626129 | -3.200966 | -1.312926 |
| H | -2.288004 | -4.845922 | -1.023345 |
| H | -1.468620 | 3.315885 | -3.305804 |
| C | 2.007608 | 0.461095 | 3.226735 |
| N | 1.795604 | -1.425631 | 1.935929 |
| C | 0.521504 | 0.281860 | 2.997628 |
| C | 0.478481 | -0.860577 | 1.990548 |
| C | 2.720265 | -0.586387 | 2.603236 |
| C | 2.684544 | 1.458673 | 3.927475 |
| C | 2.058638 | -2.605706 | 1.257914 |
| C | 4.120196 | -0.658756 | 2.676005 |
| C | 4.085914 | 1.396748 | 4.012071 |
| H | 2.130276 | 2.284235 | 4.384930 |
| N | 3.343009 | -2.980150 | 1.081785 |
| N | 0.967446 | -3.273501 | 0.816990 |
| C | 4.784593 | 0.345329 | 3.396612 |
| H | 4.655174 | -1.461679 | 2.171834 |
| H | 4.634161 | 2.174859 | 4.552611 |
| C | 3.539376 | -4.114735 | 0.404203 |
| C | 1.192804 | -4.407512 | 0.150610 |
| H | 5.877330 | 0.311316 | 3.462221 |

| | | | |
|---|---|---|---|
| C | 2.484624 | -4.891830 | -0.100948 |
| H | 4.585878 | -4.412626 | 0.254607 |
| H | 0.301177 | -4.942729 | -0.203186 |
| H | 2.660422 | -5.817358 | -0.655131 |
| H | 0.053198 | -0.047810 | 3.950638 |
| H | -0.325994 | -1.599799 | 2.038568 |

**TS(3-4)**[1]
Lowest frequency = -99.6165 $cm^{-1}$
Charge = 0, Multiplicity = 1

108

| | | | |
|---|---|---|---|
| C | 0.018831 | 1.342254 | -1.995790 |
| C | -1.768324 | 2.405609 | -0.735711 |
| C | -0.816162 | 1.287188 | -3.118265 |
| C | -2.633141 | 2.244264 | -1.830509 |
| H | -2.133208 | 2.878339 | 0.181981 |
| C | -2.147594 | 1.724714 | -3.037166 |
| H | -0.420543 | 0.844408 | -4.038457 |
| H | -3.680666 | 2.548136 | -1.740746 |
| H | -2.807481 | 1.640062 | -3.906549 |
| N | 1.488170 | -0.082383 | -0.791723 |
| C | 2.443342 | -1.612312 | 0.692195 |
| C | 0.912688 | -2.286932 | 2.339508 |
| C | 3.475839 | -2.432628 | 1.224849 |
| C | 2.652412 | -0.805472 | -0.459178 |
| C | 1.876676 | -3.138138 | 2.905129 |
| H | -0.094586 | -2.183574 | 2.750481 |
| C | 3.160632 | -3.224496 | 2.366317 |
| C | 4.742811 | -2.394278 | 0.570633 |
| C | 3.909351 | -0.765776 | -1.066646 |
| H | 1.600984 | -3.724103 | 3.787750 |
| H | 3.917650 | -3.883577 | 2.803170 |
| C | 4.945509 | -1.571313 | -0.531722 |
| H | 5.554767 | -3.024180 | 0.949879 |
| H | 4.068254 | -0.148366 | -1.950734 |
| H | 5.928324 | -1.551363 | -1.014964 |
| N | 1.170901 | -1.532698 | 1.244132 |
| C | 1.317769 | 0.594863 | -2.011414 |
| O | 2.057478 | 0.501505 | -2.988834 |
| O | -1.492398 | -0.781172 | 1.264857 |
| C | -2.740259 | -0.487095 | 0.962046 |
| C | -3.319518 | -0.768254 | -0.323389 |
| C | -3.589959 | 0.123871 | 1.953120 |
| C | -4.681974 | -0.504164 | -0.595954 |
| C | -2.481387 | -1.319722 | -1.354676 |

| | | | |
|---|---|---|---|
| C | -4.916472 | 0.395679 | 1.612502 |
| C | -5.503216 | 0.091696 | 0.355786 |
| H | -5.052230 | -0.759412 | -1.591399 |
| N | -1.173670 | -1.246239 | -1.372971 |
| O | -3.036335 | -1.975162 | -2.410247 |
| H | -5.548870 | 0.876811 | 2.363410 |
| C | -0.650407 | -1.967821 | -2.548942 |
| C | -1.946868 | -2.548875 | -3.181273 |
| H | -0.176769 | -1.243670 | -3.233694 |
| C | 0.403905 | -2.966709 | -2.108436 |
| H | -2.082868 | -2.260335 | -4.234926 |
| C | 0.148669 | -3.828163 | -1.022054 |
| C | 1.666667 | -2.989103 | -2.719548 |
| C | 1.145019 | -4.691736 | -0.555537 |
| H | -0.823404 | -3.792859 | -0.517641 |
| C | 2.665638 | -3.858491 | -2.253580 |
| H | 1.886429 | -2.285361 | -3.529404 |
| C | 2.408445 | -4.706980 | -1.170574 |
| H | 0.945497 | -5.339232 | 0.304333 |
| H | 3.655067 | -3.848529 | -2.721623 |
| H | 3.194742 | -5.367610 | -0.791058 |
| C | -0.437388 | 1.967474 | -0.803949 |
| Co | -0.066234 | -0.798956 | 0.063742 |
| C | -3.029084 | 0.469150 | 3.345483 |
| C | -6.976402 | 0.446590 | 0.096505 |
| C | -7.163128 | 1.978409 | 0.227804 |
| H | -8.218508 | 2.258878 | 0.054684 |
| H | -6.877688 | 2.335099 | 1.232627 |
| H | -6.537363 | 2.510503 | -0.510803 |
| C | -7.873468 | -0.270707 | 1.134772 |
| H | -7.611991 | 0.022754 | 2.166152 |
| H | -8.936853 | -0.017147 | 0.969714 |
| H | -7.760351 | -1.365998 | 1.055240 |
| C | -7.428309 | 0.020684 | -1.313458 |
| H | -6.841158 | 0.529407 | -2.098232 |
| H | -7.325780 | -1.068838 | -1.459077 |
| H | -8.490326 | 0.284459 | -1.462587 |
| C | -1.805639 | 1.411543 | 3.206800 |
| H | -1.030523 | 0.948699 | 2.577963 |
| H | -2.113397 | 2.370535 | 2.751358 |
| H | -1.378369 | 1.629528 | 4.202800 |
| C | -4.069743 | 1.183190 | 4.231439 |
| H | -4.407481 | 2.134323 | 3.782468 |
| H | -4.956994 | 0.552158 | 4.415806 |
| H | -3.615660 | 1.415906 | 5.210815 |
| C | -2.611934 | -0.837890 | 4.064904 |
| H | -3.491036 | -1.487785 | 4.219931 |
| H | -1.873340 | -1.386606 | 3.462641 |

| | | | |
|---|---|---|---|
| H | -2.170185 | -0.609411 | 5.052511 |
| H | -2.004242 | -3.646544 | -3.086802 |
| C | 1.379101 | 3.637053 | -0.407326 |
| N | 2.903092 | 2.184717 | 0.509177 |
| C | 0.582321 | 2.490181 | 0.206162 |
| C | 1.683207 | 1.567242 | 0.653126 |
| C | 2.756123 | 3.401242 | -0.236965 |
| C | 0.935835 | 4.757985 | -1.107039 |
| C | 4.080726 | 1.690732 | 1.106041 |
| C | 3.723173 | 4.275216 | -0.746608 |
| C | 1.890810 | 5.654061 | -1.617063 |
| H | -0.135130 | 4.922837 | -1.263299 |
| N | 5.245281 | 2.248261 | 0.741111 |
| N | 3.899216 | 0.714677 | 2.015095 |
| C | 3.261642 | 5.409804 | -1.435321 |
| H | 4.783195 | 4.066824 | -0.608457 |
| H | 1.564371 | 6.542039 | -2.167764 |
| C | 6.341193 | 1.750181 | 1.331355 |
| C | 5.006022 | 0.219547 | 2.573758 |
| H | 3.994796 | 6.111377 | -1.846843 |
| C | 6.286299 | 0.707066 | 2.263980 |
| H | 7.296898 | 2.205035 | 1.039840 |
| H | 4.859352 | -0.590050 | 3.299853 |
| H | 7.187498 | 0.296767 | 2.726602 |
| H | 0.025775 | 2.830351 | 1.103473 |
| H | 1.579170 | 0.812831 | 1.431869 |

**TS(3-4)[3]**
Lowest frequency = -466.5691 $cm^{-1}$
Charge = 0, Multiplicity = 3

108

| | | | |
|---|---|---|---|
| C | 0.589331 | -0.690924 | -2.250525 |
| C | 2.320531 | -2.189967 | -1.441832 |
| C | 1.525790 | -0.086109 | -3.105782 |
| C | 3.264725 | -1.542915 | -2.253066 |
| H | 2.628061 | -3.022453 | -0.801671 |
| C | 2.863904 | -0.497881 | -3.095798 |
| H | 1.184778 | 0.719363 | -3.763023 |
| H | 4.312051 | -1.857750 | -2.223027 |
| H | 3.595548 | 0.003964 | -3.736878 |
| N | -1.421443 | -0.186771 | -0.985474 |
| C | -3.039957 | 0.934294 | 0.356099 |
| C | -2.185521 | 2.058801 | 2.220851 |
| C | -4.388119 | 1.270476 | 0.704739 |
| C | -2.766140 | 0.175236 | -0.819340 |

| | | | |
|---|---|---|---|
| C | -3.478636 | 2.415873 | 2.641440 |
| H | -1.300503 | 2.368291 | 2.785866 |
| C | -4.587936 | 2.024570 | 1.897225 |
| C | -5.431590 | 0.842310 | -0.160654 |
| C | -3.818369 | -0.225402 | -1.653633 |
| H | -3.593564 | 3.010694 | 3.552934 |
| H | -5.603131 | 2.294946 | 2.204877 |
| C | -5.142104 | 0.120686 | -1.318430 |
| H | -6.467097 | 1.089822 | 0.096389 |
| H | -3.599790 | -0.798476 | -2.556153 |
| H | -5.957119 | -0.197104 | -1.977044 |
| N | -1.953120 | 1.324361 | 1.112559 |
| C | -0.814325 | -0.170028 | -2.238483 |
| O | -1.325290 | 0.358146 | -3.231391 |
| O | 1.344363 | -0.125934 | 1.376000 |
| C | 2.611162 | 0.044675 | 1.089888 |
| C | 3.072414 | 1.020335 | 0.135330 |
| C | 3.609330 | -0.763021 | 1.752128 |
| C | 4.447990 | 1.120230 | -0.197673 |
| C | 2.151322 | 1.900284 | -0.545036 |
| C | 4.941537 | -0.625445 | 1.364880 |
| C | 5.399069 | 0.290400 | 0.380955 |
| H | 4.726935 | 1.868481 | -0.941165 |
| N | 0.841349 | 1.920225 | -0.465650 |
| O | 2.676266 | 2.808654 | -1.423524 |
| H | 5.687415 | -1.257710 | 1.854140 |
| C | 0.327178 | 2.847168 | -1.498868 |
| C | 1.584803 | 3.668944 | -1.837911 |
| H | 0.028651 | 2.242646 | -2.374239 |
| C | -0.894446 | 3.599352 | -1.023598 |
| H | 1.704344 | 3.882436 | -2.909828 |
| C | -0.834440 | 4.456434 | 0.090290 |
| C | -2.127673 | 3.382873 | -1.660865 |
| C | -1.992142 | 5.088032 | 0.558608 |
| H | 0.118163 | 4.605074 | 0.611472 |
| C | -3.288909 | 4.010704 | -1.187979 |
| H | -2.178522 | 2.682829 | -2.502357 |
| C | -3.223526 | 4.863388 | -0.078189 |
| H | -1.939566 | 5.746030 | 1.432838 |
| H | -4.248815 | 3.814172 | -1.676464 |
| H | -4.131751 | 5.344579 | 0.299829 |
| C | 0.983063 | -1.771873 | -1.433568 |
| Co | -0.222633 | 0.618356 | 0.527045 |
| C | 3.201407 | -1.724188 | 2.886828 |
| C | 6.891636 | 0.332943 | 0.014792 |
| C | 7.323579 | -1.051333 | -0.528604 |
| H | 8.399860 | -1.052379 | -0.781432 |
| H | 7.148528 | -1.849275 | 0.213741 |

| | | | |
|---|---|---|---|
| H | 6.754331 | -1.305385 | -1.440329 |
| C | 7.726467 | 0.671478 | 1.274160 |
| H | 7.577188 | -0.077218 | 2.071350 |
| H | 8.804601 | 0.696017 | 1.030383 |
| H | 7.438203 | 1.657912 | 1.677470 |
| C | 7.186362 | 1.391666 | -1.064832 |
| H | 6.627406 | 1.186679 | -1.994826 |
| H | 6.920039 | 2.406187 | -0.720609 |
| H | 8.263444 | 1.388844 | -1.309072 |
| C | 2.235744 | -2.814172 | 2.362384 |
| H | 1.337196 | -2.349077 | 1.934151 |
| H | 2.728376 | -3.428865 | 1.587804 |
| H | 1.931580 | -3.483747 | 3.187761 |
| C | 4.420023 | -2.440473 | 3.503771 |
| H | 4.947497 | -3.069044 | 2.764116 |
| H | 5.144796 | -1.724755 | 3.930209 |
| H | 4.078655 | -3.100689 | 4.320534 |
| C | 2.507540 | -0.916330 | 4.013706 |
| H | 3.198927 | -0.158880 | 4.423838 |
| H | 1.611511 | -0.405906 | 3.629431 |
| H | 2.209182 | -1.592506 | 4.836359 |
| H | 1.643598 | 4.610405 | -1.261169 |
| C | -1.121677 | -3.242195 | -1.474417 |
| N | -2.207351 | -2.290116 | 0.305267 |
| C | -0.069856 | -2.513400 | -0.647835 |
| C | -0.957318 | -1.661053 | 0.235157 |
| C | -2.377575 | -3.111322 | -0.846378 |
| C | -0.992643 | -3.968909 | -2.655814 |
| C | -3.131465 | -1.985171 | 1.310095 |
| C | -3.526595 | -3.716223 | -1.368093 |
| C | -2.138404 | -4.577770 | -3.199173 |
| H | -0.019210 | -4.050052 | -3.149968 |
| N | -4.404539 | -2.368830 | 1.129842 |
| N | -2.624953 | -1.344933 | 2.383599 |
| C | -3.380181 | -4.454742 | -2.555722 |
| H | -4.487222 | -3.594608 | -0.868668 |
| H | -2.062167 | -5.146768 | -4.131212 |
| C | -5.261963 | -2.018388 | 2.099582 |
| C | -3.499678 | -1.001140 | 3.331790 |
| H | -4.263585 | -4.932713 | -2.992200 |
| C | -4.866595 | -1.304431 | 3.236105 |
| H | -6.306326 | -2.322960 | 1.953219 |
| H | -3.091243 | -0.466214 | 4.198225 |
| H | -5.578702 | -1.009758 | 4.010863 |
| H | 0.434093 | -3.245063 | 0.014844 |
| H | -0.600629 | -1.318154 | 1.216535 |

**TS(3-4)[5]**
Lowest frequency = -401.9179 $cm^{-1}$
Charge = 0, Multiplicity = 5

108

| | | | |
|---|---|---|---|
| C | 0.580453 | -1.019872 | -2.056244 |
| C | 2.135255 | -2.683074 | -1.213192 |
| C | 1.603541 | -0.497427 | -2.868072 |
| C | 3.164273 | -2.123180 | -1.982759 |
| H | 2.337713 | -3.553209 | -0.580518 |
| C | 2.894838 | -1.032428 | -2.821264 |
| H | 1.365823 | 0.347951 | -3.520548 |
| H | 4.174218 | -2.541175 | -1.927907 |
| H | 3.692376 | -0.593217 | -3.428717 |
| N | -1.373552 | -0.261550 | -0.818706 |
| C | -2.888392 | 1.121485 | 0.434137 |
| C | -1.964506 | 2.312155 | 2.263646 |
| C | -4.217905 | 1.563488 | 0.761208 |
| C | -2.679529 | 0.249952 | -0.680583 |
| C | -3.225600 | 2.749682 | 2.662717 |
| H | -1.053412 | 2.589480 | 2.802315 |
| C | -4.359117 | 2.386458 | 1.917889 |
| C | -5.287771 | 1.154127 | -0.076596 |
| C | -3.769849 | -0.124896 | -1.501378 |
| H | -3.317212 | 3.386319 | 3.548312 |
| H | -5.356468 | 2.728495 | 2.213518 |
| C | -5.054172 | 0.333901 | -1.194977 |
| H | -6.302497 | 1.490169 | 0.162288 |
| H | -3.592414 | -0.774537 | -2.360672 |
| H | -5.894044 | 0.037271 | -1.832038 |
| N | -1.797116 | 1.509912 | 1.172831 |
| C | -0.755377 | -0.339141 | -2.077690 |
| O | -1.175505 | 0.237046 | -3.082264 |
| O | 1.316931 | 0.063778 | 1.285254 |
| C | 2.602509 | 0.138196 | 1.025368 |
| C | 3.163553 | 1.001957 | 0.014668 |
| C | 3.521094 | -0.666164 | 1.797008 |
| C | 4.554103 | 0.982871 | -0.271819 |
| C | 2.336916 | 1.892725 | -0.762301 |
| C | 4.870333 | -0.648316 | 1.450147 |
| C | 5.423502 | 0.142947 | 0.408139 |
| H | 4.908536 | 1.647603 | -1.061279 |
| N | 1.028381 | 1.985165 | -0.740657 |
| O | 2.931359 | 2.724790 | -1.661309 |
| H | 5.553976 | -1.281466 | 2.021497 |
| C | 0.584645 | 2.885787 | -1.829868 |
| C | 1.895246 | 3.625589 | -2.153531 |

| | | | |
|---|---|---|---|
| H | 0.282104 | 2.250228 | -2.683330 |
| C | -0.628577 | 3.673465 | -1.385341 |
| H | 2.067712 | 3.783213 | -3.227247 |
| C | -0.546413 | 4.623444 | -0.350384 |
| C | -1.886506 | 3.330503 | -1.911347 |
| C | -1.708119 | 5.223800 | 0.148283 |
| H | 0.425444 | 4.871094 | 0.092014 |
| C | -3.049607 | 3.930742 | -1.408738 |
| H | -1.954102 | 2.549705 | -2.676637 |
| C | -2.962566 | 4.874653 | -0.377839 |
| H | -1.638963 | 5.952359 | 0.962981 |
| H | -4.025828 | 3.630913 | -1.802004 |
| H | -3.872124 | 5.326772 | 0.030491 |
| C | 0.840979 | -2.141507 | -1.238866 |
| Co | -0.125620 | 0.869039 | 0.328205 |
| C | 3.013690 | -1.499171 | 2.991121 |
| C | 6.924191 | 0.044237 | 0.091554 |
| C | 7.256965 | -1.408306 | -0.330432 |
| H | 8.335487 | -1.512046 | -0.549137 |
| H | 7.001213 | -2.128894 | 0.465676 |
| H | 6.691281 | -1.688457 | -1.236617 |
| C | 7.747393 | 0.415202 | 1.349343 |
| H | 7.519513 | -0.255619 | 2.195670 |
| H | 8.829824 | 0.338057 | 1.139283 |
| H | 7.527581 | 1.449303 | 1.667214 |
| C | 7.330764 | 0.989717 | -1.054731 |
| H | 6.786874 | 0.753540 | -1.986272 |
| H | 7.133526 | 2.045348 | -0.798759 |
| H | 8.411071 | 0.887618 | -1.259131 |
| C | 2.027632 | -2.589549 | 2.508473 |
| H | 1.191375 | -2.132832 | 1.962814 |
| H | 2.540467 | -3.305185 | 1.842249 |
| H | 1.625913 | -3.149996 | 3.372226 |
| C | 4.167359 | -2.207493 | 3.730534 |
| H | 4.696353 | -2.926409 | 3.079764 |
| H | 4.905612 | -1.487746 | 4.126216 |
| H | 3.756372 | -2.772437 | 4.585422 |
| C | 2.307632 | -0.566565 | 4.009180 |
| H | 3.010302 | 0.202078 | 4.377308 |
| H | 1.443501 | -0.064345 | 3.549588 |
| H | 1.956201 | -1.155316 | 4.876247 |
| H | 1.991197 | 4.584299 | -1.613180 |
| C | -1.369346 | -3.456351 | -1.325781 |
| N | -2.431721 | -2.366419 | 0.393266 |
| C | -0.290114 | -2.793851 | -0.477613 |
| C | -1.135427 | -1.873541 | 0.363710 |
| C | -2.629519 | -3.207639 | -0.741638 |
| C | -1.262093 | -4.223592 | -2.483645 |

| | | | |
|---|---|---|---|
| C | -3.382382 | -1.908776 | 1.318069 |
| C | -3.806256 | -3.732680 | -1.287959 |
| C | -2.435460 | -4.752118 | -3.050873 |
| H | -0.283440 | -4.397469 | -2.942729 |
| N | -4.671537 | -2.225297 | 1.098931 |
| N | -2.880438 | -1.227792 | 2.368057 |
| C | -3.682536 | -4.510606 | -2.452334 |
| H | -4.767980 | -3.518735 | -0.822982 |
| H | -2.376789 | -5.349771 | -3.966137 |
| C | -5.543029 | -1.750634 | 2.000730 |
| C | -3.771426 | -0.761560 | 3.246214 |
| H | -4.588128 | -4.925581 | -2.907481 |
| C | -5.151146 | -0.989381 | 3.106350 |
| H | -6.599054 | -1.993825 | 1.824572 |
| H | -3.368220 | -0.187663 | 4.089737 |
| H | -5.876065 | -0.593673 | 3.821965 |
| H | 0.148537 | -3.559790 | 0.197088 |
| H | -0.776757 | -1.405097 | 1.281641 |

**I-4[1]**
Lowest frequency = 11.6721 cm$^{-1}$
Charge = 0, Multiplicity = 1

108

| | | | |
|---|---|---|---|
| C | 0.054501 | 1.418551 | -2.015020 |
| C | -1.709146 | 2.490752 | -0.725334 |
| C | -0.792554 | 1.355551 | -3.131072 |
| C | -2.580883 | 2.343251 | -1.817132 |
| H | -2.062067 | 2.955190 | 0.200742 |
| C | -2.113838 | 1.813931 | -3.030274 |
| H | -0.416516 | 0.893786 | -4.049398 |
| H | -3.624009 | 2.660109 | -1.721495 |
| H | -2.787364 | 1.736331 | -3.889787 |
| N | 1.666978 | 0.259632 | -0.635068 |
| C | 2.421267 | -1.614329 | 0.626954 |
| C | 0.863154 | -2.321571 | 2.235389 |
| C | 3.391840 | -2.561754 | 1.065488 |
| C | 2.716723 | -0.718184 | -0.425400 |
| C | 1.775095 | -3.270994 | 2.724362 |
| H | -0.136049 | -2.193704 | 2.658578 |
| C | 3.038165 | -3.413585 | 2.150893 |
| C | 4.640616 | -2.583089 | 0.379052 |
| C | 3.942357 | -0.744975 | -1.077265 |
| H | 1.470007 | -3.899162 | 3.567228 |
| H | 3.750091 | -4.159955 | 2.516442 |
| C | 4.901042 | -1.703570 | -0.665307 |

| | | | |
|---|---|---|---|
| H | 5.396241 | -3.314711 | 0.685079 |
| H | 4.153070 | -0.044355 | -1.885854 |
| H | 5.867131 | -1.741948 | -1.178965 |
| N | 1.154409 | -1.504964 | 1.193471 |
| C | 1.304683 | 0.629470 | -1.996685 |
| O | 1.873217 | 0.153919 | -2.972443 |
| O | -1.471256 | -0.707202 | 1.248505 |
| C | -2.723221 | -0.419390 | 0.951004 |
| C | -3.301923 | -0.695412 | -0.333143 |
| C | -3.572990 | 0.175584 | 1.950929 |
| C | -4.666036 | -0.436927 | -0.602806 |
| C | -2.458379 | -1.241071 | -1.363362 |
| C | -4.902727 | 0.437706 | 1.615031 |
| C | -5.490047 | 0.142697 | 0.356405 |
| H | -5.035396 | -0.686021 | -1.600134 |
| N | -1.153378 | -1.137338 | -1.393234 |
| O | -3.009299 | -1.930266 | -2.396991 |
| H | -5.537346 | 0.905721 | 2.372263 |
| C | -0.622106 | -1.909856 | -2.534618 |
| C | -1.922294 | -2.370748 | -3.254551 |
| H | -0.036421 | -1.246032 | -3.188836 |
| C | 0.300868 | -2.996571 | -2.009936 |
| H | -2.049358 | -1.891829 | -4.239328 |
| C | -0.117936 | -3.836016 | -0.958282 |
| C | 1.598393 | -3.140952 | -2.528464 |
| C | 0.747345 | -4.804568 | -0.439444 |
| H | -1.114700 | -3.705306 | -0.522305 |
| C | 2.464553 | -4.115954 | -2.010572 |
| H | 1.941709 | -2.458757 | -3.312492 |
| C | 2.041676 | -4.947767 | -0.966313 |
| H | 0.419385 | -5.439607 | 0.389769 |
| H | 3.479383 | -4.207606 | -2.410523 |
| H | 2.723135 | -5.696097 | -0.548393 |
| C | -0.385751 | 2.039945 | -0.814570 |
| Co | -0.051823 | -0.672131 | 0.056395 |
| C | -3.012447 | 0.512628 | 3.345591 |
| C | -6.966468 | 0.489153 | 0.104274 |
| C | -7.164895 | 2.017937 | 0.252573 |
| H | -8.222878 | 2.291806 | 0.085021 |
| H | -6.879988 | 2.366052 | 1.260562 |
| H | -6.545169 | 2.562977 | -0.481623 |
| C | -7.855583 | -0.246351 | 1.136634 |
| H | -7.594239 | 0.038066 | 2.170591 |
| H | -8.921235 | 0.000714 | 0.976600 |
| H | -7.734180 | -1.339817 | 1.045082 |
| C | -7.417982 | 0.074805 | -1.309237 |
| H | -6.836428 | 0.596450 | -2.089697 |
| H | -7.307485 | -1.012310 | -1.466641 |

| | | | |
|---|---|---|---|
| H | -8.482267 | 0.331981 | -1.453459 |
| C | -1.796730 | 1.465995 | 3.215194 |
| H | -1.013114 | 1.013412 | 2.589397 |
| H | -2.109453 | 2.423744 | 2.760851 |
| H | -1.376828 | 1.684316 | 4.214161 |
| C | -4.058320 | 1.210452 | 4.238469 |
| H | -4.404225 | 2.162493 | 3.797848 |
| H | -4.940044 | 0.570464 | 4.418348 |
| H | -3.605165 | 1.438581 | 5.219279 |
| C | -2.584240 | -0.796781 | 4.053752 |
| H | -3.456352 | -1.458425 | 4.198275 |
| H | -1.835806 | -1.331426 | 3.450932 |
| H | -2.149458 | -0.573077 | 5.045451 |
| H | -1.995519 | -3.464718 | -3.360311 |
| C | 1.504512 | 3.606443 | -0.405960 |
| N | 3.041846 | 2.068769 | 0.342980 |
| C | 0.682936 | 2.454529 | 0.167199 |
| C | 1.755194 | 1.370381 | 0.411916 |
| C | 2.879363 | 3.323578 | -0.295575 |
| C | 1.066029 | 4.785653 | -1.004850 |
| C | 4.148086 | 1.618706 | 1.051104 |
| C | 3.845554 | 4.222646 | -0.769957 |
| C | 2.021928 | 5.701213 | -1.479293 |
| H | -0.005785 | 4.985124 | -1.108330 |
| N | 5.333660 | 2.212113 | 0.809782 |
| N | 3.923535 | 0.620502 | 1.932539 |
| C | 3.390792 | 5.415637 | -1.357167 |
| H | 4.905221 | 3.988910 | -0.671509 |
| H | 1.697434 | 6.634063 | -1.951324 |
| C | 6.382809 | 1.726514 | 1.487328 |
| C | 4.988973 | 0.139313 | 2.575109 |
| H | 4.128050 | 6.131743 | -1.735760 |
| C | 6.279624 | 0.657490 | 2.384822 |
| H | 7.348145 | 2.213832 | 1.294413 |
| H | 4.798655 | -0.686908 | 3.272728 |
| H | 7.149005 | 0.257285 | 2.912690 |
| H | 0.228174 | 2.735691 | 1.132434 |
| H | 1.659112 | 0.857286 | 1.375571 |

**I-4$^3$**
Lowest frequency = 14.9100 cm$^{-1}$
Charge = 0, Multiplicity = 3

108

| | | | |
|---|---|---|---|
| C | -0.607143 | 0.900662 | -2.018635 |
| C | -2.158239 | 2.550036 | -1.143162 |

| | | | |
|---|---|---|---|
| C | -1.604087 | 0.428634 | -2.892222 |
| C | -3.164542 | 2.046369 | -1.981093 |
| H | -2.370693 | 3.385754 | -0.468912 |
| C | -2.880803 | 0.997870 | -2.869482 |
| H | -1.360781 | -0.398292 | -3.565238 |
| H | -4.170261 | 2.474979 | -1.939996 |
| H | -3.663689 | 0.610528 | -3.529066 |
| N | 1.442006 | 0.370716 | -0.749854 |
| C | 2.914372 | -1.178214 | 0.404364 |
| C | 1.979262 | -2.286189 | 2.249035 |
| C | 4.213167 | -1.722267 | 0.668977 |
| C | 2.739085 | -0.265412 | -0.665869 |
| C | 3.228768 | -2.820870 | 2.599799 |
| H | 1.081811 | -2.497416 | 2.839378 |
| C | 4.350627 | -2.561717 | 1.810821 |
| C | 5.275096 | -1.378425 | -0.214034 |
| C | 3.791777 | 0.052845 | -1.519207 |
| H | 3.303226 | -3.451631 | 3.491013 |
| H | 5.328680 | -2.986721 | 2.057898 |
| C | 5.060815 | -0.524361 | -1.292346 |
| H | 6.269722 | -1.798688 | -0.030022 |
| H | 3.631395 | 0.757634 | -2.338069 |
| H | 5.889613 | -0.272443 | -1.961545 |
| N | 1.801159 | -1.493256 | 1.168513 |
| C | 0.705513 | 0.210032 | -1.975905 |
| O | 1.123226 | -0.532467 | -2.865020 |
| O | -1.172950 | -0.185833 | 1.242310 |
| C | -2.462100 | -0.138982 | 1.035138 |
| C | -3.139449 | -0.893148 | 0.002990 |
| C | -3.290722 | 0.682660 | 1.893631 |
| C | -4.532060 | -0.738929 | -0.225844 |
| C | -2.426337 | -1.832756 | -0.821868 |
| C | -4.646417 | 0.804213 | 1.599133 |
| C | -5.301680 | 0.130873 | 0.532574 |
| H | -4.970653 | -1.325824 | -1.035008 |
| N | -1.126894 | -1.994769 | -0.887553 |
| O | -3.130239 | -2.657701 | -1.653417 |
| H | -5.253880 | 1.453016 | 2.235927 |
| C | -0.802087 | -3.005417 | -1.915309 |
| C | -2.188569 | -3.620446 | -2.201601 |
| H | -0.414351 | -2.470644 | -2.801897 |
| C | 0.309634 | -3.884124 | -1.372372 |
| H | -2.406850 | -3.736754 | -3.273169 |
| C | 0.076584 | -4.837757 | -0.364047 |
| C | 1.633139 | -3.603486 | -1.759032 |
| C | 1.149092 | -5.505163 | 0.240577 |
| H | -0.945848 | -5.043144 | -0.027262 |
| C | 2.706790 | -4.267719 | -1.148436 |

| | | | |
|---|---|---|---|
| H | 1.816671 | -2.827173 | -2.509480 |
| C | 2.467629 | -5.218013 | -0.147417 |
| H | 0.957147 | -6.242468 | 1.027552 |
| H | 3.732039 | -4.025289 | -1.445270 |
| H | 3.305944 | -5.727693 | 0.338731 |
| C | -0.874904 | 1.989005 | -1.164981 |
| Co | 0.178872 | -1.109715 | 0.180902 |
| C | -2.678599 | 1.372064 | 3.129447 |
| C | -6.799965 | 0.370238 | 0.286610 |
| C | -7.037102 | 1.872466 | -0.004651 |
| H | -8.112499 | 2.073523 | -0.163912 |
| H | -6.693557 | 2.505375 | 0.831821 |
| H | -6.489409 | 2.183363 | -0.911794 |
| C | -7.604617 | -0.039649 | 1.544592 |
| H | -7.291768 | 0.539464 | 2.430633 |
| H | -8.685023 | 0.136426 | 1.389122 |
| H | -7.454923 | -1.110108 | 1.769328 |
| C | -7.320608 | -0.444905 | -0.912445 |
| H | -6.787324 | -0.180289 | -1.842549 |
| H | -7.203633 | -1.529970 | -0.746173 |
| H | -8.394809 | -0.240410 | -1.067563 |
| C | -1.590556 | 2.387246 | 2.707589 |
| H | -0.798667 | 1.873900 | 2.145952 |
| H | -2.026627 | 3.181296 | 2.075222 |
| H | -1.145486 | 2.864545 | 3.599918 |
| C | -3.737776 | 2.135030 | 3.950585 |
| H | -4.204096 | 2.949195 | 3.367374 |
| H | -4.537912 | 1.465913 | 4.313381 |
| H | -3.254188 | 2.590923 | 4.832502 |
| C | -2.049500 | 0.295946 | 4.051997 |
| H | -2.819929 | -0.421125 | 4.387225 |
| H | -1.259320 | -0.254432 | 3.519138 |
| H | -1.610557 | 0.774566 | 4.947041 |
| H | -2.347131 | -4.585232 | -1.687798 |
| C | 1.189251 | 3.450216 | -1.292509 |
| N | 2.547371 | 2.339607 | 0.178967 |
| C | 0.280638 | 2.589738 | -0.417190 |
| C | 1.291839 | 1.584299 | 0.161185 |
| C | 2.524365 | 3.293704 | -0.865575 |
| C | 0.871749 | 4.305014 | -2.344001 |
| C | 3.558789 | 2.064042 | 1.083321 |
| C | 3.567902 | 4.009124 | -1.468411 |
| C | 1.909412 | 5.026088 | -2.964742 |
| H | -0.166068 | 4.404776 | -2.678928 |
| N | 4.756744 | 2.651532 | 0.888205 |
| N | 3.236623 | 1.226845 | 2.093828 |
| C | 3.232888 | 4.878294 | -2.522593 |
| H | 4.592118 | 3.883188 | -1.118379 |

| | | | |
|---|---|---|---|
| H | 1.683196 | 5.700655 | -3.796685 |
| C | 5.711186 | 2.346585 | 1.777111 |
| C | 4.210322 | 0.933038 | 2.958305 |
| H | 4.032149 | 5.443824 | -3.013720 |
| C | 5.502716 | 1.467992 | 2.847229 |
| H | 6.687098 | 2.825297 | 1.618902 |
| H | 3.944480 | 0.241054 | 3.767927 |
| H | 6.296524 | 1.219040 | 3.556091 |
| H | -0.091104 | 3.201897 | 0.423192 |
| H | 1.063055 | 1.212951 | 1.166923 |

**I-4[5]**
Lowest frequency = 14.2276 $cm^{-1}$
Charge = 0, Multiplicity = 5

108

| | | | |
|---|---|---|---|
| C | 0.605368 | -1.011751 | -1.995554 |
| C | 2.190195 | -2.579976 | -1.030996 |
| C | 1.604141 | -0.546969 | -2.875867 |
| C | 3.194158 | -2.084853 | -1.878458 |
| H | 2.415743 | -3.385472 | -0.325233 |
| C | 2.894780 | -1.077722 | -2.811104 |
| H | 1.348380 | 0.244915 | -3.585822 |
| H | 4.209221 | -2.487089 | -1.810519 |
| H | 3.676242 | -0.696504 | -3.476040 |
| N | -1.398566 | -0.399458 | -0.696274 |
| C | -2.901626 | 1.196850 | 0.386342 |
| C | -2.018086 | 2.479551 | 2.176889 |
| C | -4.231740 | 1.683175 | 0.644268 |
| C | -2.704451 | 0.237185 | -0.635857 |
| C | -3.285310 | 2.927734 | 2.531757 |
| H | -1.119309 | 2.793947 | 2.716249 |
| C | -4.404174 | 2.545805 | 1.764594 |
| C | -5.281965 | 1.248122 | -0.208662 |
| C | -3.756700 | -0.163878 | -1.479771 |
| H | -3.395960 | 3.597093 | 3.390302 |
| H | -5.406409 | 2.909450 | 2.013437 |
| C | -5.038380 | 0.358420 | -1.265336 |
| H | -6.296204 | 1.616037 | -0.019639 |
| H | -3.567066 | -0.893792 | -2.269912 |
| H | -5.863578 | 0.041101 | -1.910938 |
| N | -1.817189 | 1.635578 | 1.121986 |
| C | -0.711080 | -0.345509 | -1.975352 |
| O | -1.144118 | 0.367041 | -2.877605 |
| O | 1.265964 | 0.175249 | 1.332486 |
| C | 2.551918 | 0.187313 | 1.060601 |

| | | | |
|---|---|---|---|
| C | 3.130971 | 0.967730 | -0.004322 |
| C | 3.445450 | -0.601022 | 1.874928 |
| C | 4.517249 | 0.881641 | -0.298154 |
| C | 2.329936 | 1.851155 | -0.815955 |
| C | 4.791847 | -0.651911 | 1.519543 |
| C | 5.362605 | 0.055329 | 0.427757 |
| H | 4.888543 | 1.484550 | -1.128343 |
| N | 1.024653 | 1.992460 | -0.792504 |
| O | 2.949875 | 2.631167 | -1.741856 |
| H | 5.458466 | -1.271158 | 2.125130 |
| C | 0.605887 | 2.896976 | -1.887814 |
| C | 1.948609 | 3.550791 | -2.267762 |
| H | 0.232108 | 2.264449 | -2.714111 |
| C | -0.534782 | 3.773353 | -1.413518 |
| H | 2.109148 | 3.640385 | -3.351289 |
| C | -0.343155 | 4.748341 | -0.416962 |
| C | -1.836420 | 3.493994 | -1.864776 |
| C | -1.438868 | 5.437804 | 0.115219 |
| H | 0.663072 | 4.949904 | -0.031498 |
| C | -2.933751 | 4.181024 | -1.326504 |
| H | -1.989009 | 2.696926 | -2.599748 |
| C | -2.737263 | 5.151428 | -0.336182 |
| H | -1.283390 | 6.188067 | 0.897771 |
| H | -3.944913 | 3.933346 | -1.663700 |
| H | -3.595672 | 5.673903 | 0.098100 |
| C | 0.894100 | -2.053330 | -1.085808 |
| Co | -0.148820 | 0.963048 | 0.337776 |
| C | 2.919109 | -1.327957 | 3.128765 |
| C | 6.859410 | -0.103996 | 0.116706 |
| C | 7.160491 | -1.591026 | -0.192905 |
| H | 8.237048 | -1.734064 | -0.398139 |
| H | 6.886905 | -2.244528 | 0.653465 |
| H | 6.592587 | -1.926416 | -1.078524 |
| C | 7.692749 | 0.343603 | 1.342727 |
| H | 7.451501 | -0.256281 | 2.237254 |
| H | 8.772921 | 0.227534 | 1.139052 |
| H | 7.495425 | 1.403226 | 1.581353 |
| C | 7.284420 | 0.741749 | -1.098720 |
| H | 6.728044 | 0.451650 | -2.007406 |
| H | 7.117975 | 1.818645 | -0.921828 |
| H | 8.360229 | 0.594885 | -1.299410 |
| C | 1.854344 | -2.384290 | 2.747790 |
| H | 1.016242 | -1.909781 | 2.219335 |
| H | 2.294960 | -3.161645 | 2.098910 |
| H | 1.467401 | -2.877287 | 3.658008 |
| C | 4.048195 | -2.055945 | 3.886511 |
| H | 4.521364 | -2.839723 | 3.268392 |
| H | 4.834147 | -1.357764 | 4.224561 |

| | | | |
|---|---|---|---|
| H | 3.626515 | -2.545873 | 4.781450 |
| C | 2.296407 | -0.287535 | 4.095678 |
| H | 3.052849 | 0.458556 | 4.397314 |
| H | 1.453766 | 0.235712 | 3.619174 |
| H | 1.928788 | -0.793620 | 5.007067 |
| H | 2.101340 | 4.532362 | -1.785188 |
| C | -1.162972 | -3.530895 | -1.177995 |
| N | -2.530736 | -2.366047 | 0.242380 |
| C | -0.257920 | -2.645532 | -0.323220 |
| C | -1.274946 | -1.628623 | 0.226301 |
| C | -2.501611 | -3.350854 | -0.770056 |
| C | -0.839942 | -4.422180 | -2.197094 |
| C | -3.561152 | -2.026606 | 1.111267 |
| C | -3.543526 | -4.079681 | -1.359593 |
| C | -1.875816 | -5.157836 | -2.803278 |
| H | 0.200385 | -4.539708 | -2.518532 |
| N | -4.769262 | -2.581308 | 0.901644 |
| N | -3.230412 | -1.171472 | 2.100356 |
| C | -3.202812 | -4.986358 | -2.379323 |
| H | -4.570139 | -3.930458 | -1.025732 |
| H | -1.645879 | -5.861776 | -3.609427 |
| C | -5.736775 | -2.206905 | 1.753906 |
| C | -4.215047 | -0.806779 | 2.928962 |
| H | -4.000765 | -5.562745 | -2.859873 |
| C | -5.522915 | -1.297542 | 2.795278 |
| H | -6.725323 | -2.654706 | 1.585988 |
| H | -3.944732 | -0.096372 | 3.719905 |
| H | -6.327402 | -0.989727 | 3.468176 |
| H | 0.117682 | -3.233283 | 0.533182 |
| H | -1.063216 | -1.254766 | 1.236657 |

**2a**
Lowest frequency = 54.8908 cm$^{-1}$
Charge = 0, Multiplicity = 1

24

| | | | |
|---|---|---|---|
| C | -0.209060 | -0.331455 | 0.058779 |
| C | 0.843929 | 0.469987 | 0.526976 |
| C | -0.698714 | 2.380548 | 0.482951 |
| C | -1.506398 | 0.208155 | -0.200196 |
| C | -1.573443 | -2.010256 | -0.681031 |
| N | -0.270288 | -1.708624 | -0.245363 |
| H | -1.798413 | -3.037141 | -0.959259 |
| H | -0.867963 | 3.448528 | 0.656732 |
| C | 0.576267 | 1.829627 | 0.733254 |
| C | -1.744665 | 1.578902 | 0.016805 |

| | | | |
|---|---|---|---|
| C | -2.340158 | -0.876928 | -0.664596 |
| H | -2.734729 | 2.004328 | -0.178023 |
| H | 1.380050 | 2.477901 | 1.098239 |
| H | 1.825455 | 0.037640 | 0.718065 |
| H | -3.389976 | -0.814251 | -0.952026 |
| C | 0.740283 | -2.667344 | -0.155496 |
| C | 1.357283 | -4.845138 | -0.432088 |
| C | 2.895122 | -3.210704 | 0.348230 |
| C | 2.657399 | -4.546664 | 0.002190 |
| H | 1.076343 | -5.865814 | -0.724930 |
| H | 3.882107 | -2.881065 | 0.699314 |
| H | 3.436946 | -5.310229 | 0.066426 |
| N | 1.949893 | -2.265218 | 0.274457 |
| N | 0.395853 | -3.921116 | -0.515092 |

## References


1. Sen, C., Sahoo, T., Singh, H., Suresh, E. & Ghosh, S. C. Visible Light-Promoted Photocatalytic C-5 Carboxylation of 8-Aminoquinoline Amides and Sulfonamides via a Single Electron Transfer Pathway. *J. Org. Chem.* **84**, 9869–9896 (2019).
2. Ackermann, L. & Lygin, A. V. Ruthenium-Catalyzed Direct C–H Bond Arylations of Heteroarenes. *Org. Lett.* **13**, 3332–3335 (2011).
3. Ban, Y. -L., You, L., Wang, T., Wu, L.-Z. & Liu, Q. Metallaphotoredox Dearomatization of Indoles by a BenzamideEmpowered [4+2] Annulation: Facile Access to Indolo[2,3-*c*]isoquinolin-5-ones. *ACS Catal.* **11**, 5054−5060 (2021).
4. Rout, L. & Harned, A. M. Allene Carboxylates as Dipolarophiles in Rh-Catalyzed Carbonyl Ylide Cycloadditions. *Chem.–Eur. J.* **15**, 12926–13267 (2009).
5. Devleshova, N. A., Lozovskiy, S. V. & Vasilyev, A. V. Reactions of Alkyl 4-Hydroxybut-2-ynoates with Arenes Under Superelectrophilic Activation with Triflic Acid or HUSY Zeolite: Alternative Propargylation or Allenylation of Arenes, And Synthesis of Furan-2-ones. *Tetrahedron* **75**, 130517 (2019).
6. Burés, J. Variable Time Normalization Analysis: General Graphical Elucidation of Reaction Orders from Concentration Profiles. *Angew. Chem. Int. Ed.* **55**, 16084–16087 (2016).
7. Frisch, M. J., Trucks, G. W., Schlegel, H. B., Scuseria, G. E., Robb, M. A., Cheeseman, J. R., Scalmani, G., Barone, V., Petersson, G. A., Nakatsuji, H., Li, X., Caricato, M., Marenich, A. V., Bloino, J., Janesko, B. G., Gomperts, R., Mennucci, B., Hratchian, H. P., Ortiz, J. V., Izmaylov, A. F., Sonnenberg, J. L., Williams, Ding, F., Lipparini, F., Egidi, F., Goings, J., Peng, B., Petrone, A., Henderson, T., Ranasinghe, D., Zakrzewski, V. G., Gao, J., Rega, N., Zheng, G., Liang, W., Hada, M., Ehara, M., Toyota, K., Fukuda, R., Hasegawa, J., Ishida, M., Nakajima, T., Honda, Y., Kitao, O., Nakai, H., Vreven, T., Throssell, K., Montgomery Jr., J. A., Peralta, J. E., Ogliaro, F., Bearpark, M. J., Heyd, J. J., Brothers, E. N., Kudin, K. N., Staroverov, V. N., Keith, T. A., Kobayashi, R., Normand, J., Raghavachari, K., Rendell, A. P., Burant, J. C., Iyengar, S. S., Tomasi, J., Cossi, M., Millam, J. M., Klene, M., Adamo, C., Cammi, R., Ochterski, J. W., Martin, R. L., Morokuma, K., Farkas, O., Foresman, J. B. & Fox, D. J. *Gaussian 16 Rev. A.03*, Wallingford, CT, 2016.
8. Tao, J., Perdew, J. P., Staroverov, V. N. & Scuseria, G. E. Climbing the Density Functional Ladder: Nonempirical Meta-Generalized Gradient Approximation Designed for Molecules

and Solids. *Phys. Rev. Lett.* **2003,** *91*, 146401.

9. Grimme, S., Ehrlich, S. & Goerigk, L. Effect of the damping function in dispersion corrected density functional theory. *J. Comput. Chem.* **2011,** *32*, 1456-1465.
10. Grimme, S., Antony, J., Ehrlich, S. & Krieg, H. A consistent and accurate ab initio parametrization of density functional dispersion correction (DFT-D) for the 94 elements H-Pu. *J. Chem. Phys.* **2010,** *132*, 154104.
11. Weigend, F. Accurate Coulomb-fitting basis sets for H to Rn. *Phys. Chem. Chem. Phys.* **2006,** *8*, 1057-1065.
12. Weigend, F. & Ahlrichs, R. Balanced basis sets of split valence, triple zeta valence and quadruple zeta valence quality for H to Rn: Design and assessment of accuracy. *Phys. Chem. Chem. Phys.* **2005,** *7*, 3297-3305.
13. Chai, J.-D. & Head-Gordon, M. Long-range corrected hybrid density functionals with damped atom–atom dispersion corrections. *Phys. Chem. Chem. Phys.* **2008,** *10*, 6615-6620.
14. Marenich, A. V., Cramer, C. J. & Truhlar, D. G. Universal Solvation Model Based on Solute Electron Density and on a Continuum Model of the Solvent Defined by the Bulk Dielectric Constant and Atomic Surface Tensions. *J. Phys. Chem. B* **2009,** *113*, 6378-6396.

# X-Ray Crystallographic Data

Compound **4**

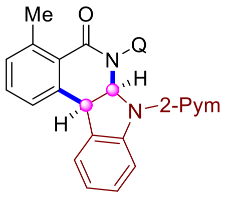


**4**

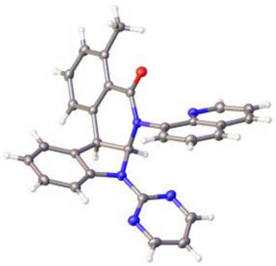

**CCDC: 2282475**

| | |
|---|---|
| Empirical formula | $C_{29}H_{21}N_5O$ |
| Formula weight | 455.51 |
| Temperature [K] | 100.00 |
| Crystal system | monoclinic |
| Space group (number) | $P2_1$ (4) |
| $a$ [Å] | 9.5820(3) |
| $b$ [Å] | 10.6434(3) |
| $c$ [Å] | 11.5298(4) |
| α [°] | 90 |
| β [°] | 108.1510(10) |
| γ [°] | 90 |
| Volume [$Å^3$] | 1117.35(6)) |
| $Z$ | 2 |
| $\rho_{calc}$ [$gcm^{-3}$] | 1.354 |

| | |
|---|---|
| $\mu$ [mm$^{-1}$] | 0.678 |
| $F$(000) | 476 |
| Crystal size [mm$^3$] | 0.316×0.238×0.231 |
| Crystal colour | yellow |
| Crystal shape | block |
| Radiation | Cu$K_\alpha$ (λ=1.54178 Å) |
| 2Θ range [°] | 8.07 to 158.81 (0.78 Å) |
| Index ranges | $-12 \leq h \leq 12$, $-13 \leq k \leq 13$, $-14 \leq l \leq 14$ |
| Reflections collected | 38036 |
| Independent reflections | 4748 [$R_{int}$ = 0.0391, $R_{sigma}$ = 0.0224] |
| Completeness to Θ = 67.679° | 99.9 % |
| Data / Restraints / Parameters | 4748/1/318 |
| Goodness-of-fit on $F^2$ | 1.021 |
| Final $R$ indexes [$I \geq 2\sigma(I)$] | $R_1$ = 0.0318, w$R_2$ = 0.0837 |
| Final $R$ indexes [all data] | $R_1$ = 0.0319, w$R_2$ = 0.0838 |
| Largest peak/hole [eÅ$^{-3}$] | 0.19/-0.17 |
| Flack X parameter | 0.15(16) |
| Extinction coefficient | 0.0092(12) |

Complex **35**

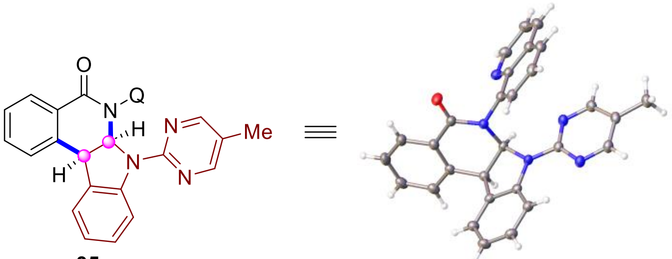


**35**

**CCDC: 2282491**

| | |
|---|---|
| Empirical formula | $C_{29}H_{21}N_5O$ |
| Formula weight | 455.51 |
| Temperature [K] | 100.00 |
| Crystal system | orthorhombic |
| Space group (number) | $P2_12_12_1$ (19) |
| *a* [Å] | 7.74820(10) |
| *b* [Å] | 8.9337(2) |
| *c* [Å] | 32.1632(6) |
| α [°] | 90 |
| β [°] | 90 |
| γ [°] | 90 |
| Volume [Å$^3$] | 2226.34(7) |
| *Z* | 4 |
| $\rho_{calc}$ [gcm$^{-3}$] | 1.359 |
| $\mu$ [mm$^{-1}$] | 0.681 |
| *F*(000) | 952 |

| | |
|---|---|
| Crystal size [$mm^3$] | 0.286×0.211×0.167 |
| Crystal colour | yellow |
| Crystal shape | block |
| Radiation | Cu$K_\alpha$ ($\lambda$=1.54178 Å) |
| 2θ range [°] | 5.50 to 158.99 (0.78 Å) |
| Index ranges | $-9 \leq h \leq 9$<br>$-11 \leq k \leq 11$<br>$-38 \leq l \leq 41$ |
| Reflections collected | 78695 |
| Independent reflections | 4803<br>$R_{int}$ = 0.0513<br>$R_{sigma}$ = 0.0150 |
| Completeness to Θ = 67.679° | 100.0 % |
| Data / Restraints / Parameters | 4803/0/318 |
| Goodness-of-fit on $F^2$ | 1.030 |
| Final *R* indexes [$I \geq 2\sigma(I)$] | $R_1$ = 0.0246<br>w$R_2$ = 0.0640 |
| Final *R* indexes [all data] | $R_1$ = 0.0247<br>w$R_2$ = 0.0640 |
| Largest peak/hole [eÅ$^{-3}$] | 0.18/-0.13 |
| Flack X parameter | 0.09(6) |
| Extinction coefficient | 0.0026(2) |

# NMR Spectra

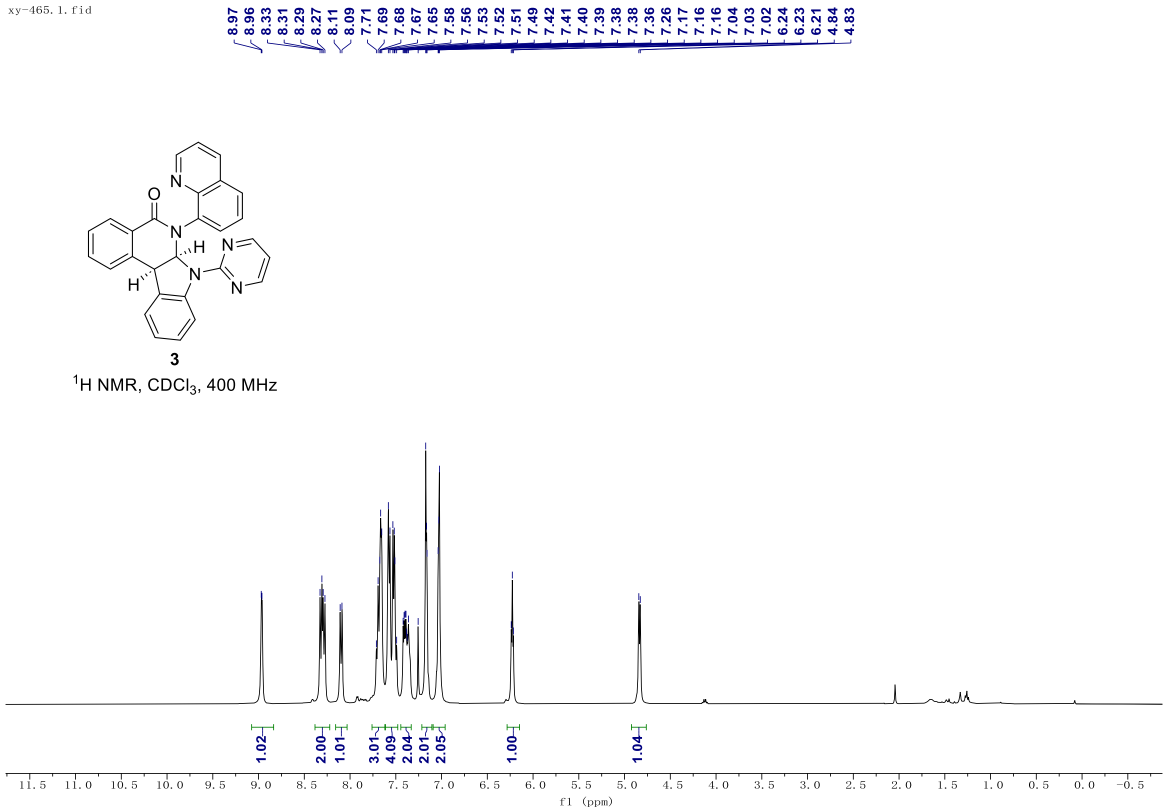


**3**

$^{1}$H NMR, $CDCl_3$, 400 MHz

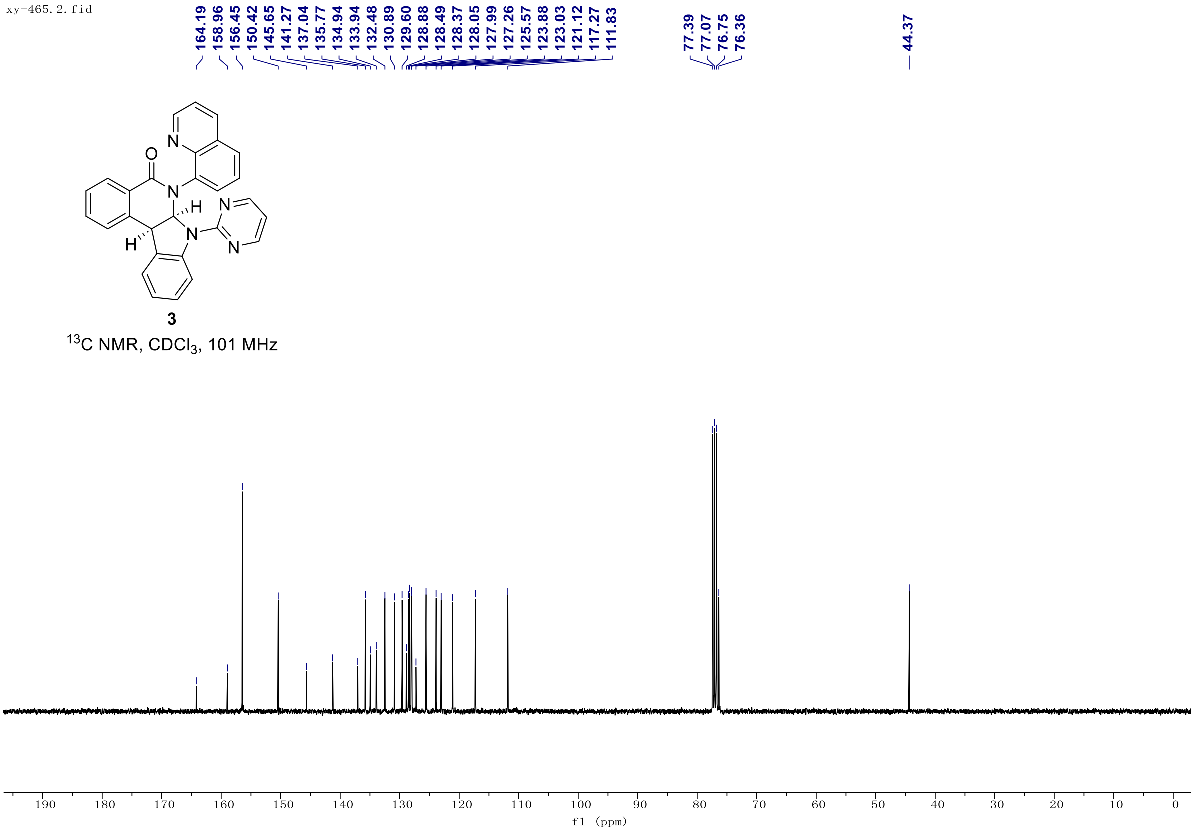


**3**

$^{13}$C NMR, $CDCl_3$, 101 MHz

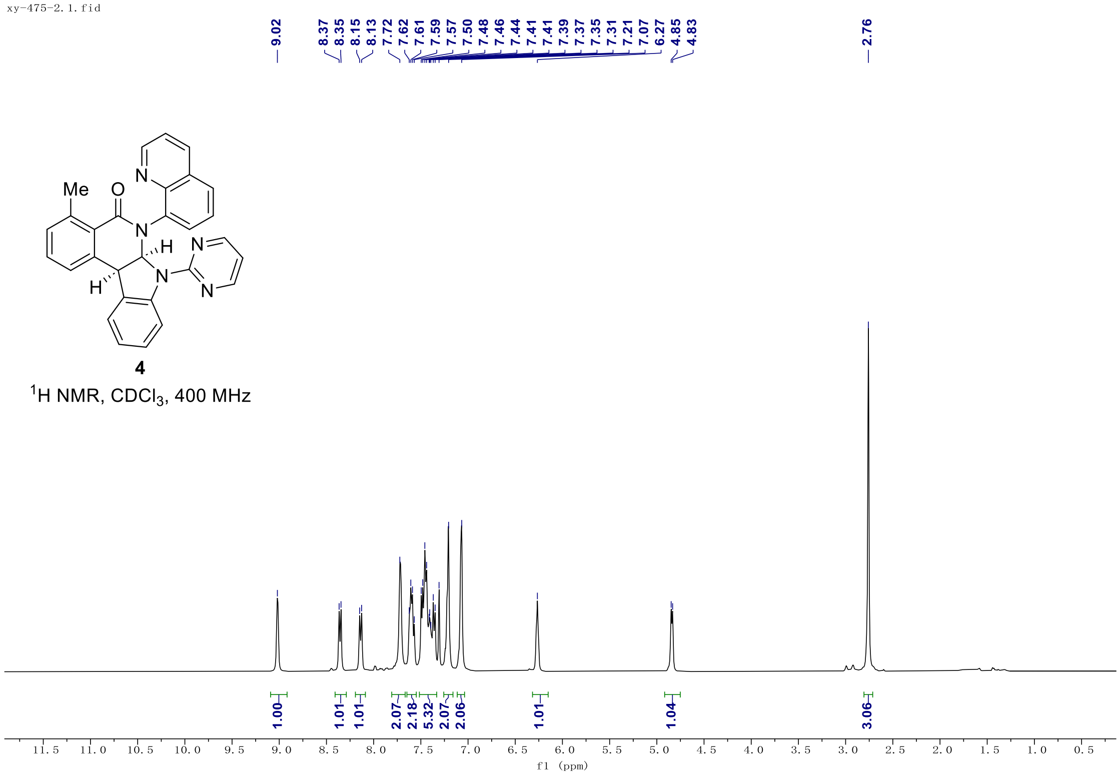
xy-475-2.1.fid
9.02
8.37
8.35
8.15
8.13
7.72
7.62
7.61
7.59
7.57
7.50
7.48
7.46
7.44
7.41
7.41
7.39
7.37
7.35
7.31
7.21
7.07
6.27
4.85
4.83
2.76
Me
O
N
N
H
H
N
N
N
4
$^1$H NMR, $CDCl_3$, 400 MHz
1.00
1.01
1.01
2.07
2.18
5.32
2.07
2.06
1.01
1.04
3.06
11.5 11.0 10.5 10.0 9.5 9.0 8.5 8.0 7.5 7.0 6.5 6.0 5.5 5.0 4.5 4.0 3.5 3.0 2.5 2.0 1.5 1.0 0.5
f1 (ppm)

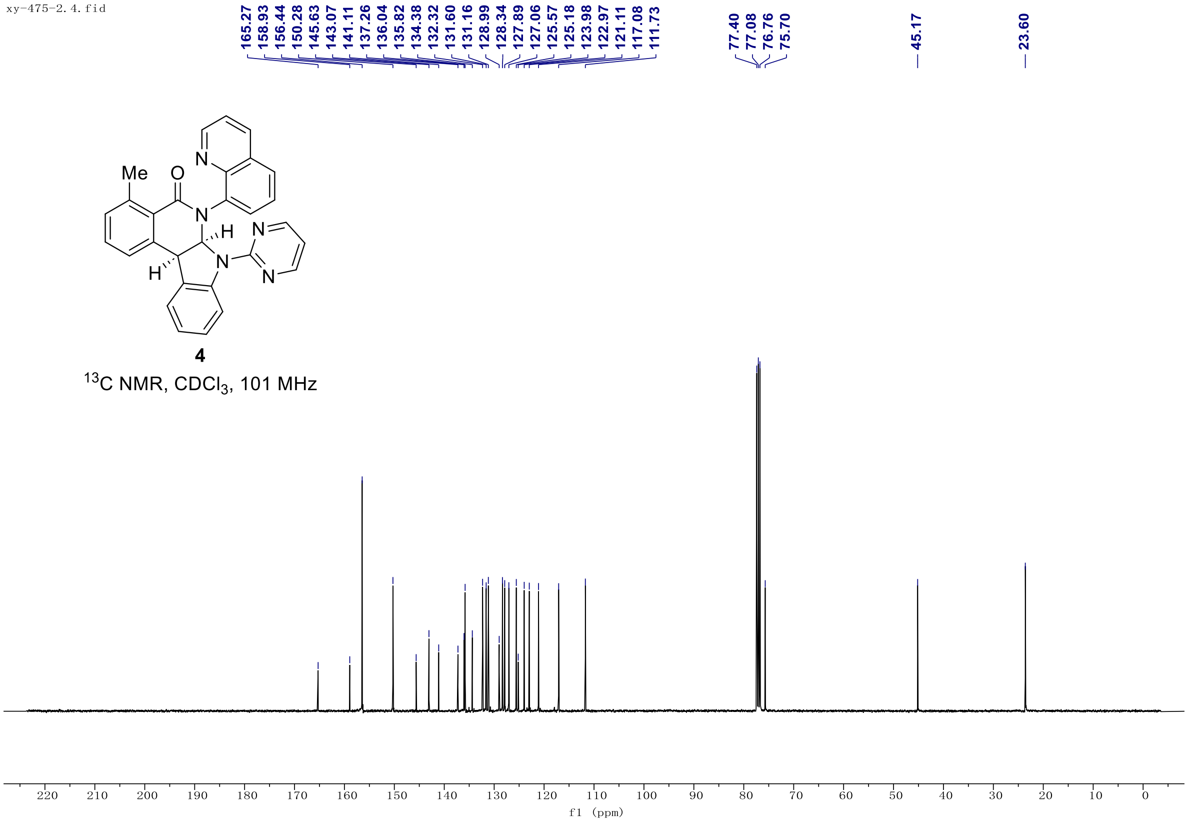
xy-475-2.4.fid
165.27
158.93
156.44
150.28
145.63
143.07
141.11
137.26
136.04
135.82
134.38
132.32
131.60
131.16
128.99
128.34
127.89
127.06
125.57
125.18
123.98
122.97
121.11
117.08
111.73
77.40
77.08
76.76
75.70
45.17
23.60
Me
O
N
N
H
H
N
N
N
4
$^{13}$C NMR, $CDCl_3$, 101 MHz
220 210 200 190 180 170 160 150 140 130 120 110 100 90 80 70 60 50 40 30 20 10 0
f1 (ppm)

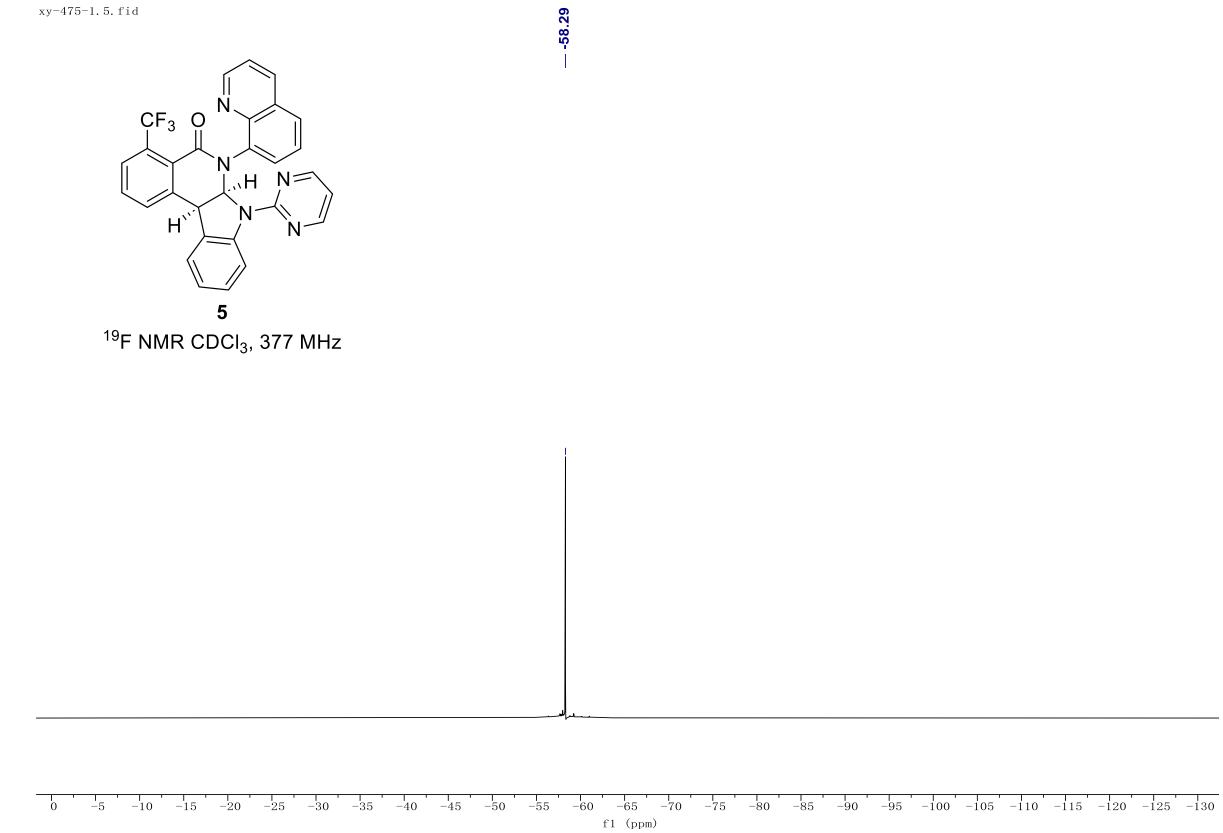

xy-475-1.5.fid
-58.29
5
$^{19}$F NMR CDCl$_3$, 377 MHz
f1 (ppm)


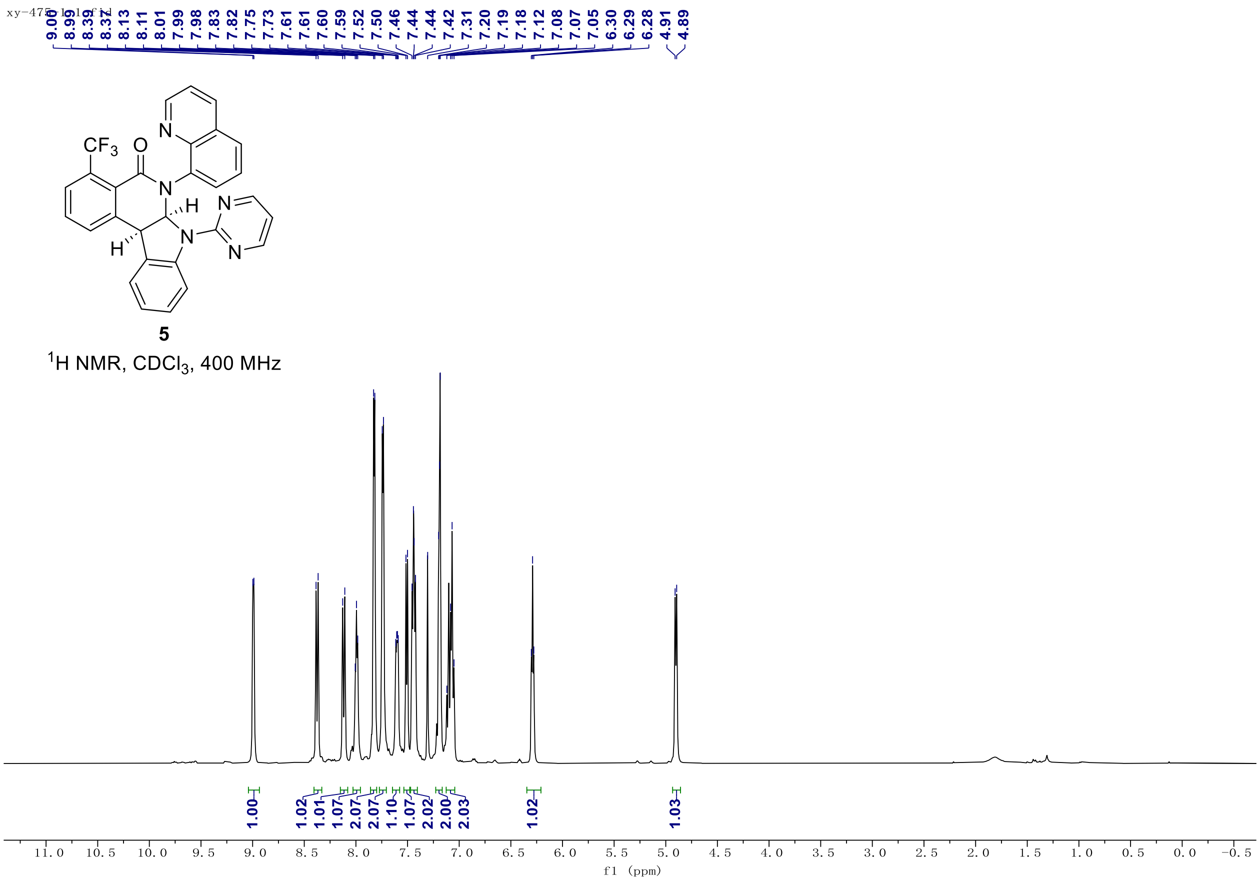

9.00
8.99
8.39
8.37
8.13
8.11
8.01
7.99
7.98
7.83
7.82
7.75
7.73
7.61
7.61
7.60
7.59
7.52
7.50
7.46
7.44
7.44
7.42
7.31
7.20
7.19
7.18
7.12
7.08
7.07
7.05
6.30
6.29
6.28
4.91
4.89
5
$^{1}$H NMR, CDCl$_3$, 400 MHz
1.00
1.02
1.01
1.07
2.07
2.07
1.10
1.07
2.02
2.00
2.03
1.02
1.03
f1 (ppm)

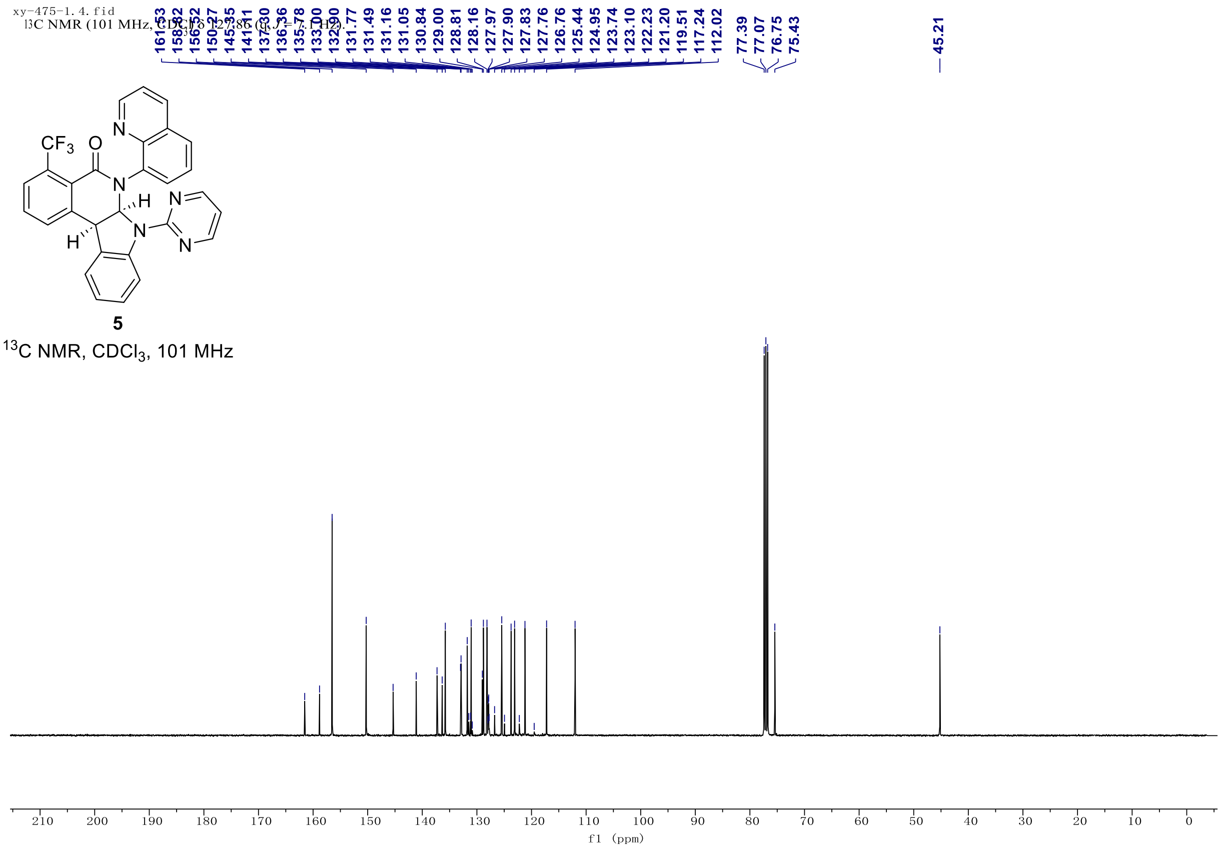
xy-475-1.4.fid
161.53
158.82
156.52
150.27
145.35
141.11
137.30
136.36
135.78
133.00
132.90
131.77
131.49
131.16
131.05
130.84
129.00
128.81
128.16
127.97
127.90
127.83
127.76
126.76
125.44
124.95
123.74
123.10
122.23
121.20
119.51
117.24
112.02
77.39
77.07
76.75
75.43
45.21
CF3
O
N
N
H
H
N
N
N
5
13C NMR, CDCl3, 101 MHz
210 200 190 180 170 160 150 140 130 120 110 100 90 80 70 60 50 40 30 20 10 0
f1 (ppm)

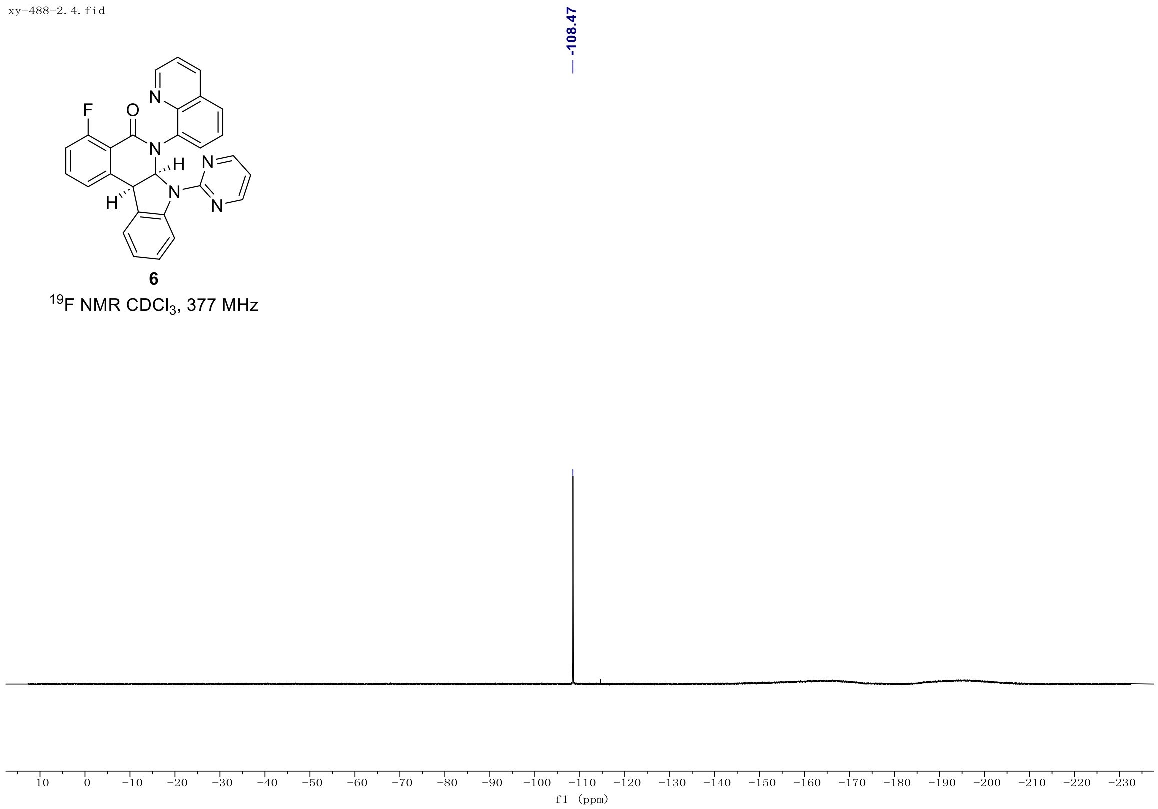
xy-488-2.4.fid
-108.47
F
O
N
N
H
H
N
N
N
6
19F NMR CDCl3, 377 MHz
10 0 −10 −20 −30 −40 −50 −60 −70 −80 −90 −100 −110 −120 −130 −140 −150 −160 −170 −180 −190 −200 −210 −220 −230
f1 (ppm)

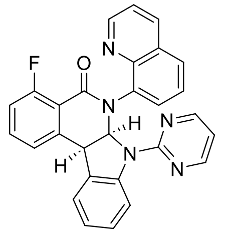

6

$^{1}H$ NMR, $CDCl_3$, 400 MHz

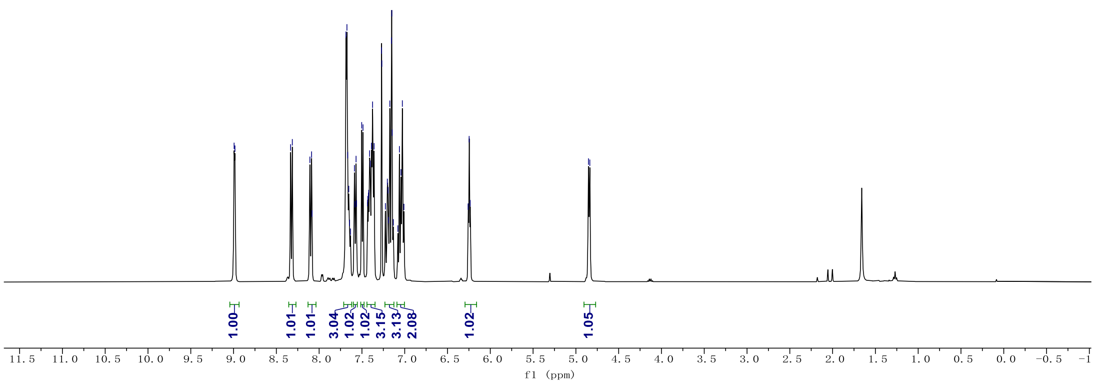


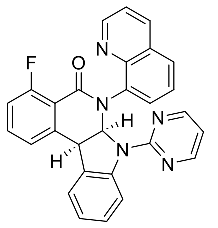

6

$^{13}C$ NMR, $CDCl_3$, 101 MHz

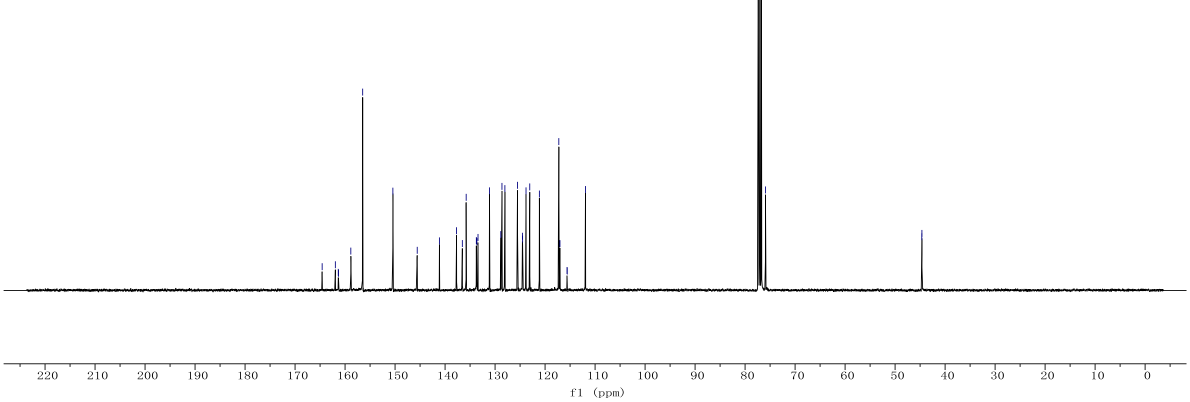

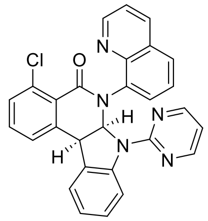

**7**

$^{1}H$ NMR, $CDCl_3$, 400 MHz

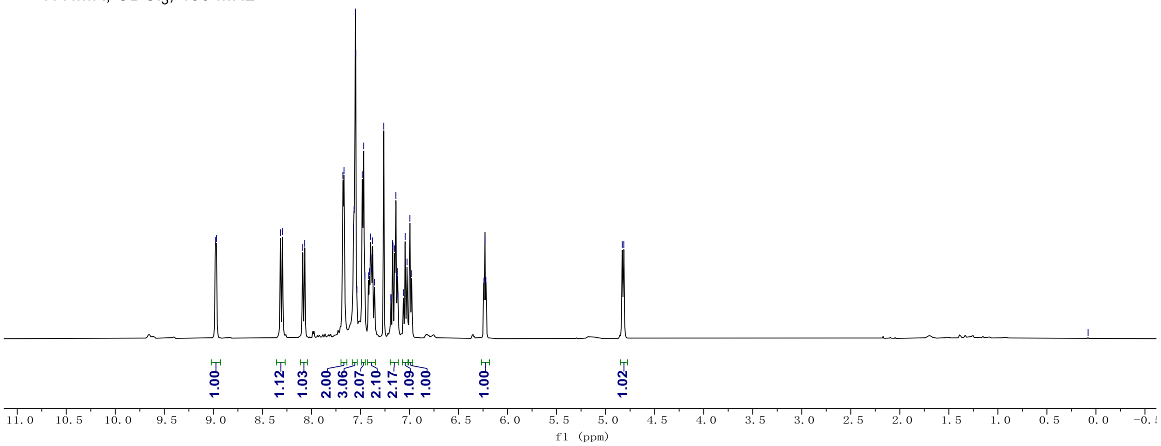


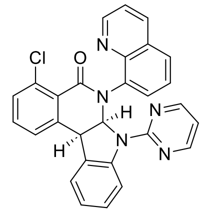

**7**

$^{13}C$ NMR, $CDCl_3$, 101 MHz

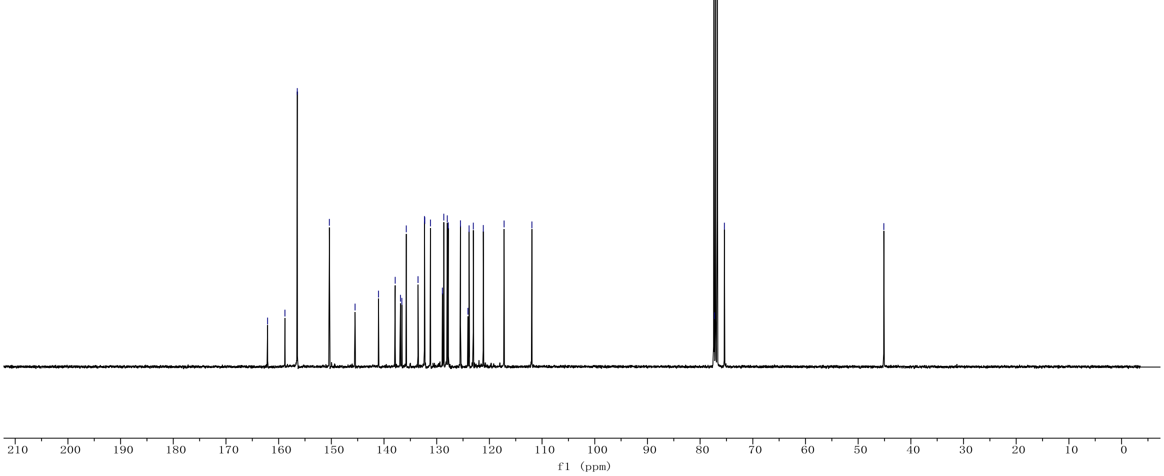

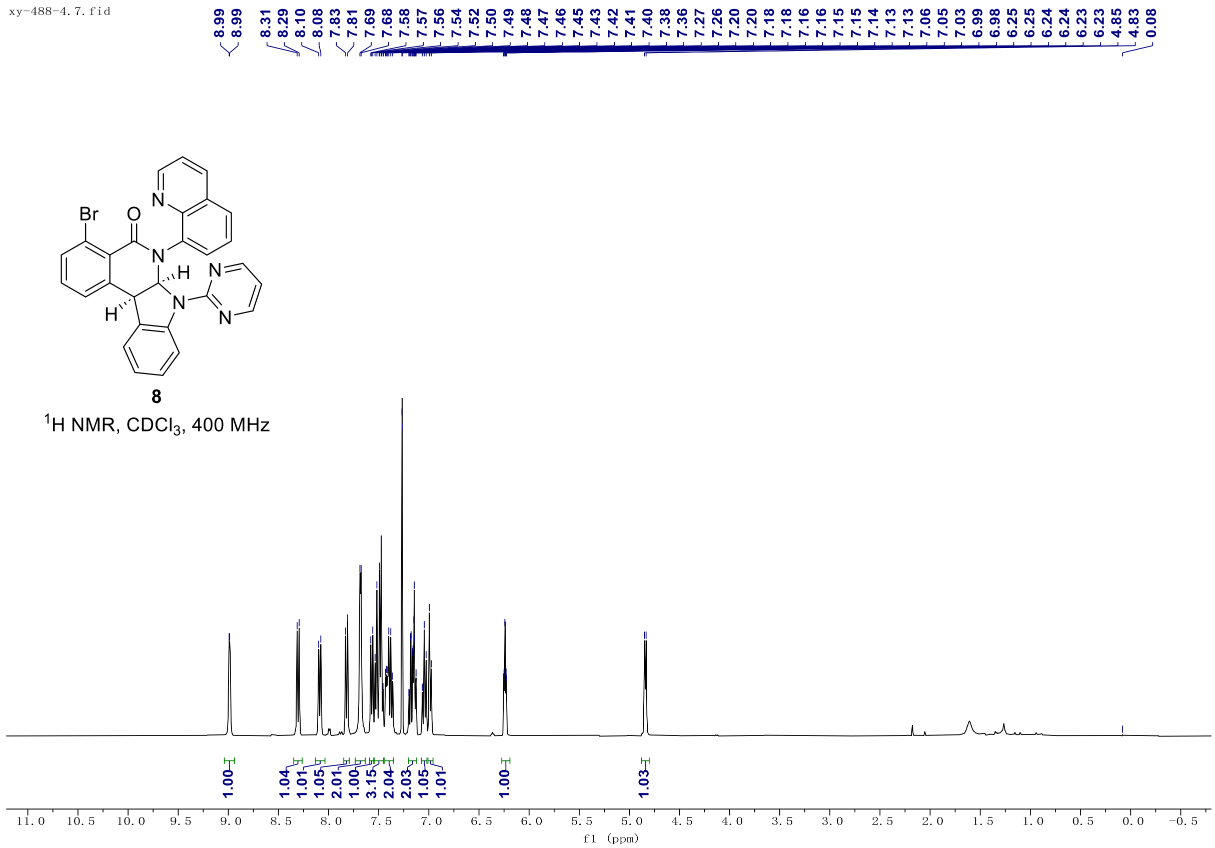
xy-488-4.7.fid
8.99
8.99
8.31
8.29
8.10
8.08
7.83
7.81
7.69
7.68
7.58
7.57
7.56
7.54
7.52
7.50
7.49
7.48
7.47
7.46
7.45
7.43
7.42
7.41
7.40
7.38
7.36
7.27
7.26
7.20
7.20
7.18
7.16
7.16
7.15
7.15
7.14
7.13
7.13
7.06
7.05
7.03
6.99
6.98
6.25
6.24
6.24
6.23
4.85
4.83
0.08
Br
O
N
N
H
H
N
N
N
8
1H NMR, CDCl3, 400 MHz
1.00
1.04
1.01
1.05
2.01
1.00
3.15
2.04
2.03
1.05
1.01
1.00
1.03
11.0 10.5 10.0 9.5 9.0 8.5 8.0 7.5 7.0 6.5 6.0 5.5 5.0 4.5 4.0 3.5 3.0 2.5 2.0 1.5 1.0 0.5 0.0 -0.5
f1 (ppm)

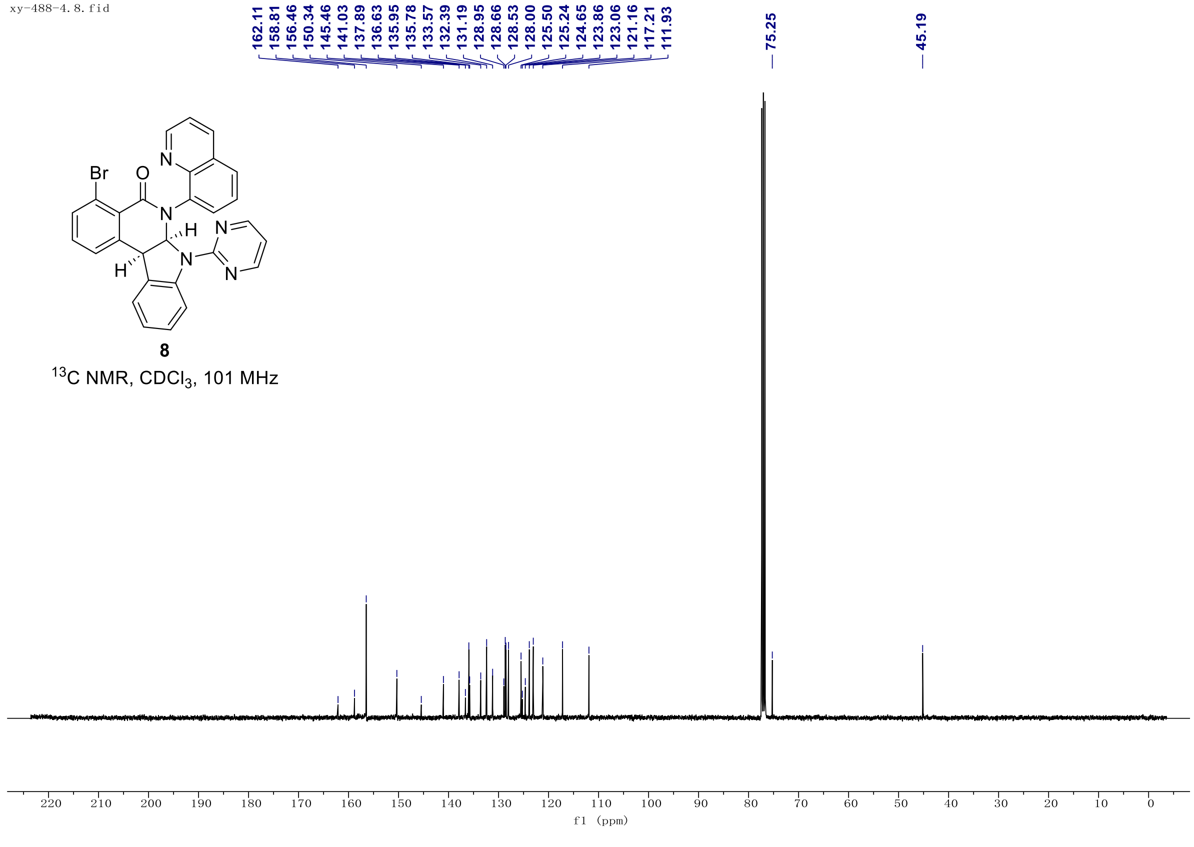
xy-488-4.8.fid
162.11
158.81
156.46
150.34
145.46
141.03
137.89
136.63
135.95
135.78
133.57
132.39
131.19
128.95
128.66
128.53
128.00
125.50
125.24
124.65
123.86
123.06
121.16
117.21
111.93
75.25
45.19
Br
O
N
N
H
H
N
N
N
8
13C NMR, CDCl3, 101 MHz
220 210 200 190 180 170 160 150 140 130 120 110 100 90 80 70 60 50 40 30 20 10 0
f1 (ppm)

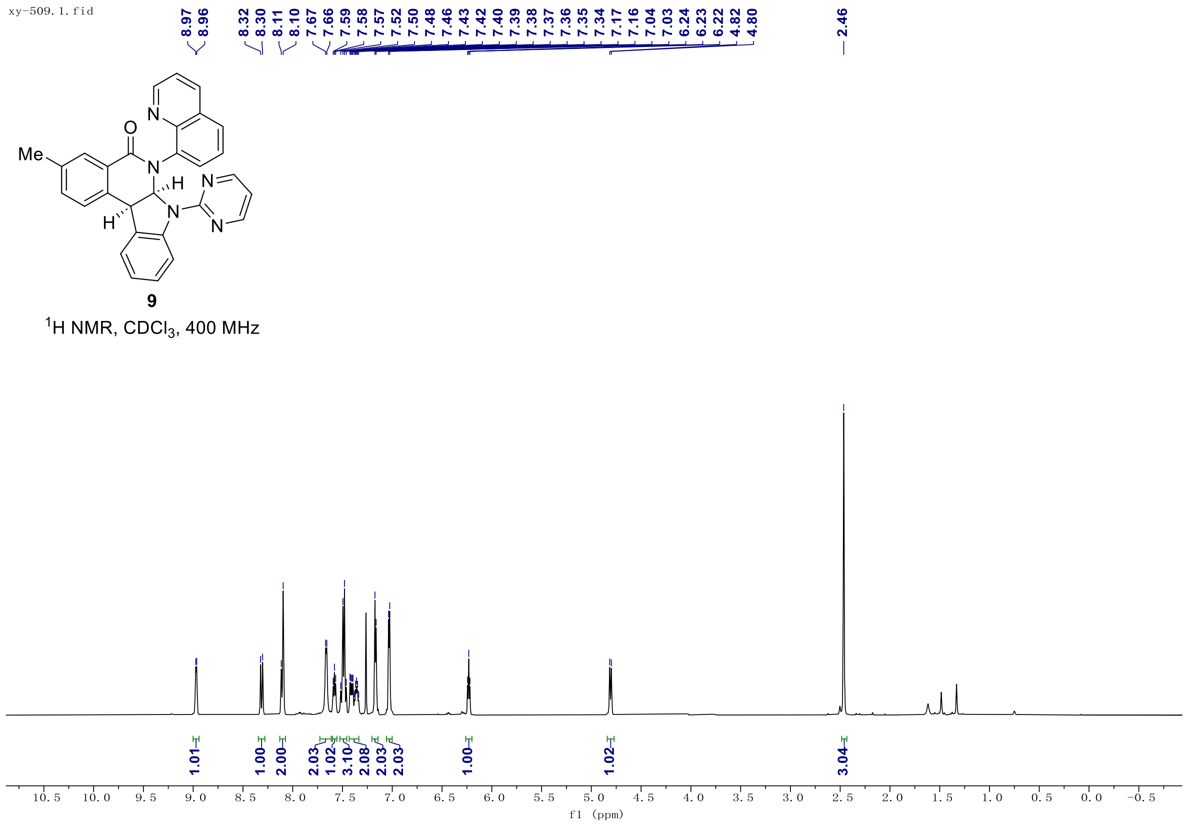
xy-509.1.fid
8.97 8.96 8.32 8.30 8.11 8.10 7.67 7.66 7.59 7.58 7.57 7.52 7.50 7.48 7.46 7.43 7.42 7.40 7.39 7.38 7.37 7.36 7.35 7.34 7.17 7.16 7.04 7.03 6.24 6.23 6.22 4.82 4.80
2.46
Me
O
N
N
H
H
N
N
N
9
$^{1}$H NMR, CDCl$_3$, 400 MHz
1.01 1.00 2.00 2.03 1.02 3.10 2.08 2.03 2.03 1.00 1.02 3.04
10.5 10.0 9.5 9.0 8.5 8.0 7.5 7.0 6.5 6.0 5.5 5.0 4.5 4.0 3.5 3.0 2.5 2.0 1.5 1.0 0.5 0.0 -0.5
f1 (ppm)

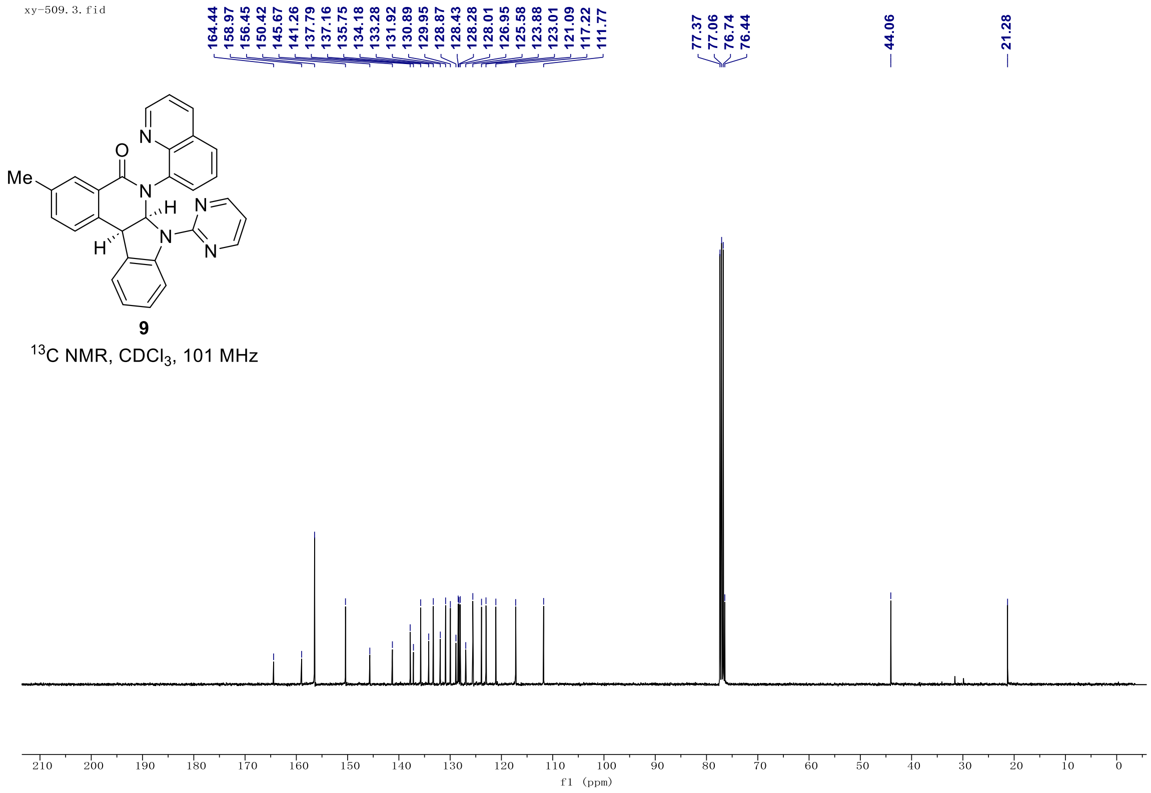
xy-509.3.fid
164.44 158.97 156.45 150.42 145.67 141.26 137.79 137.16 135.75 134.18 133.28 131.92 130.89 129.95 128.87 128.43 128.28 128.01 126.95 125.58 123.88 123.01 121.09 117.22 111.77
77.37 77.06 76.74 76.44
44.06
21.28
Me
O
N
N
H
H
N
N
N
9
$^{13}$C NMR, CDCl$_3$, 101 MHz
210 200 190 180 170 160 150 140 130 120 110 100 90 80 70 60 50 40 30 20 10 0
f1 (ppm)

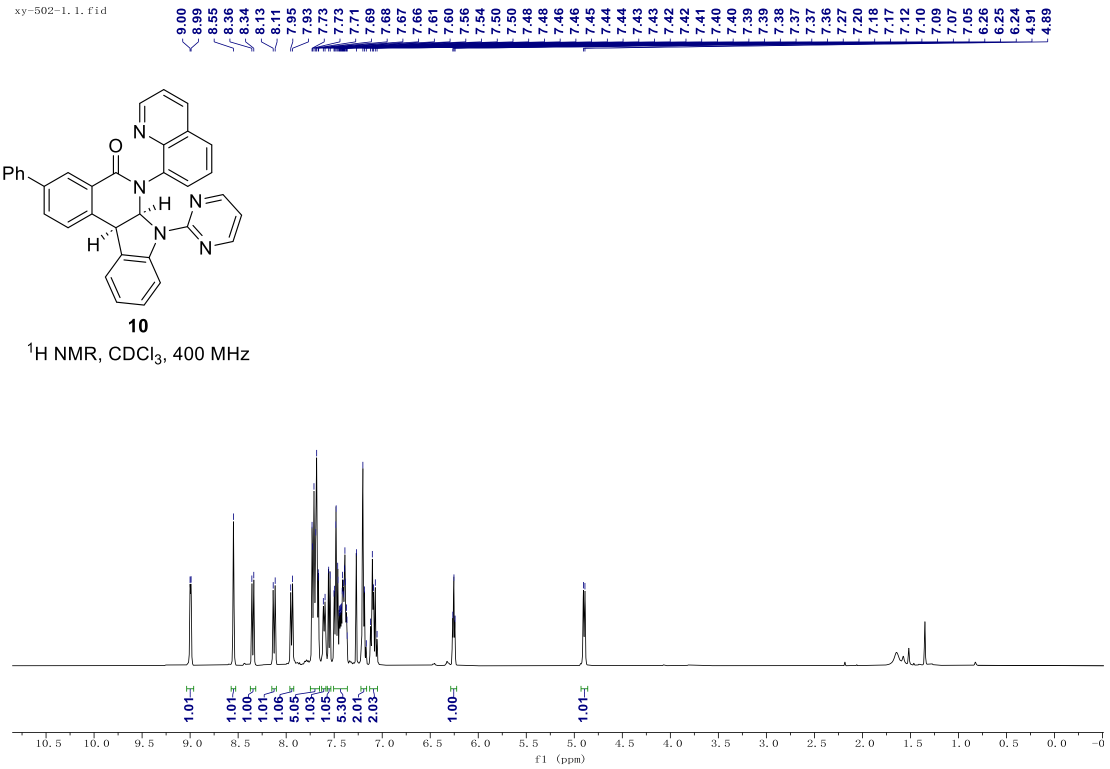

**10**

$^1H$ NMR, $CDCl_3$, 400 MHz

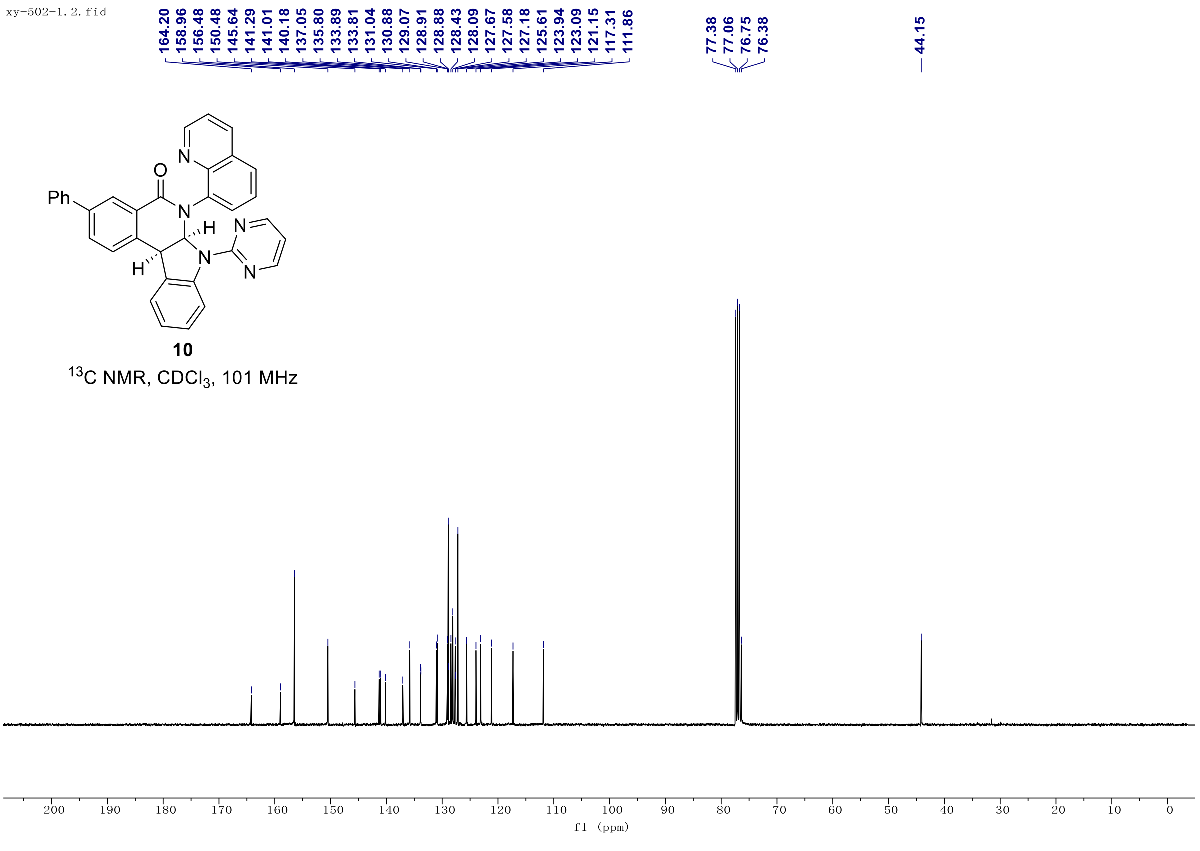

**10**

$^{13}C$ NMR, $CDCl_3$, 101 MHz

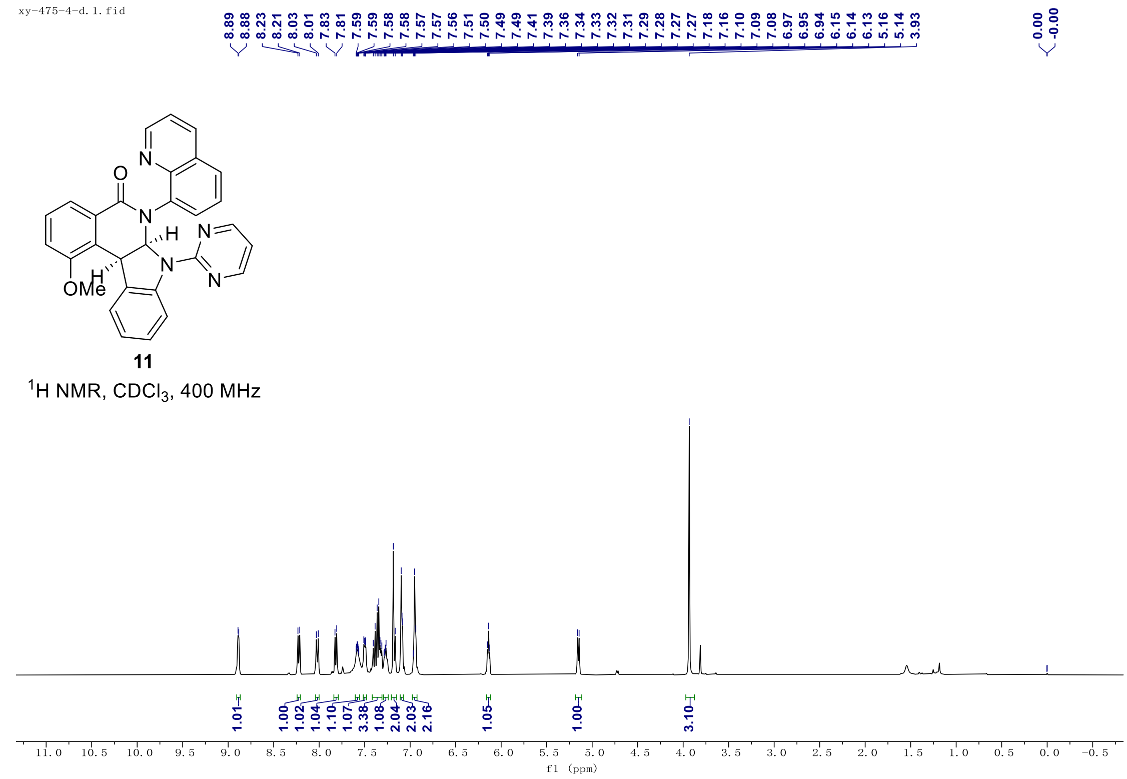


**11**

$^{1}H$ NMR, $CDCl_3$, 400 MHz

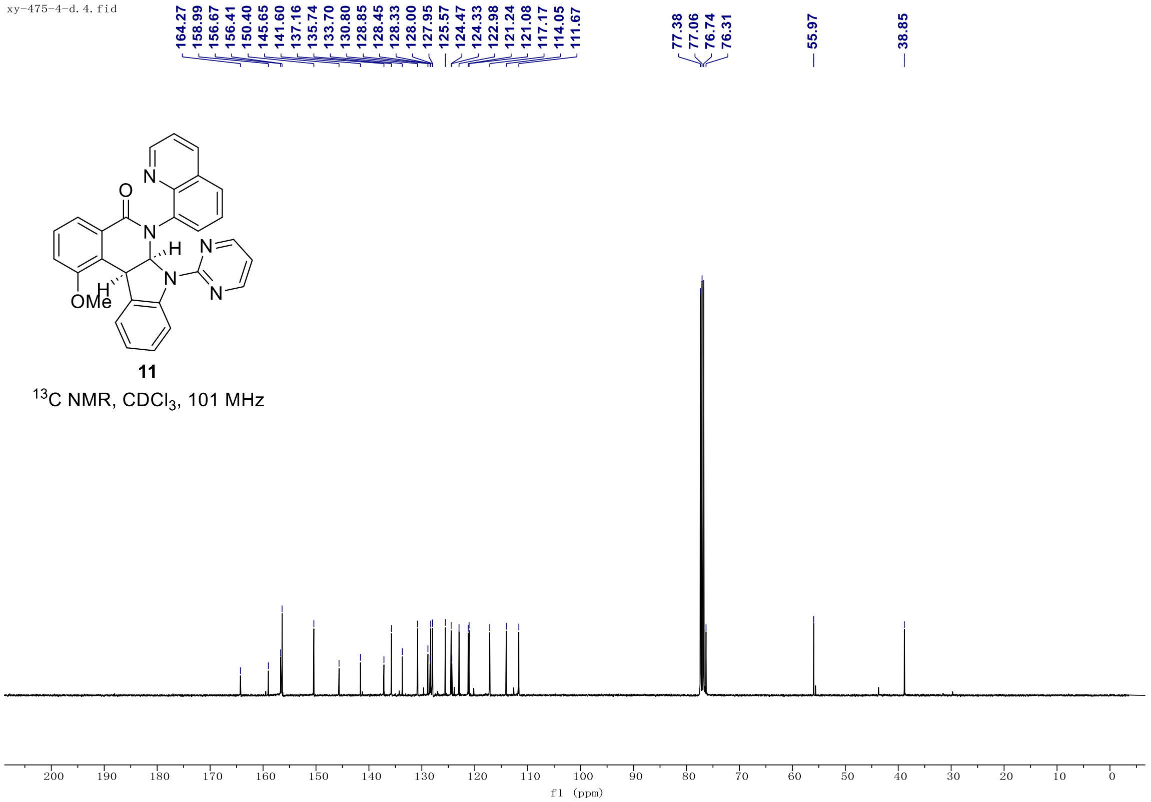


**11**

$^{13}C$ NMR, $CDCl_3$, 101 MHz

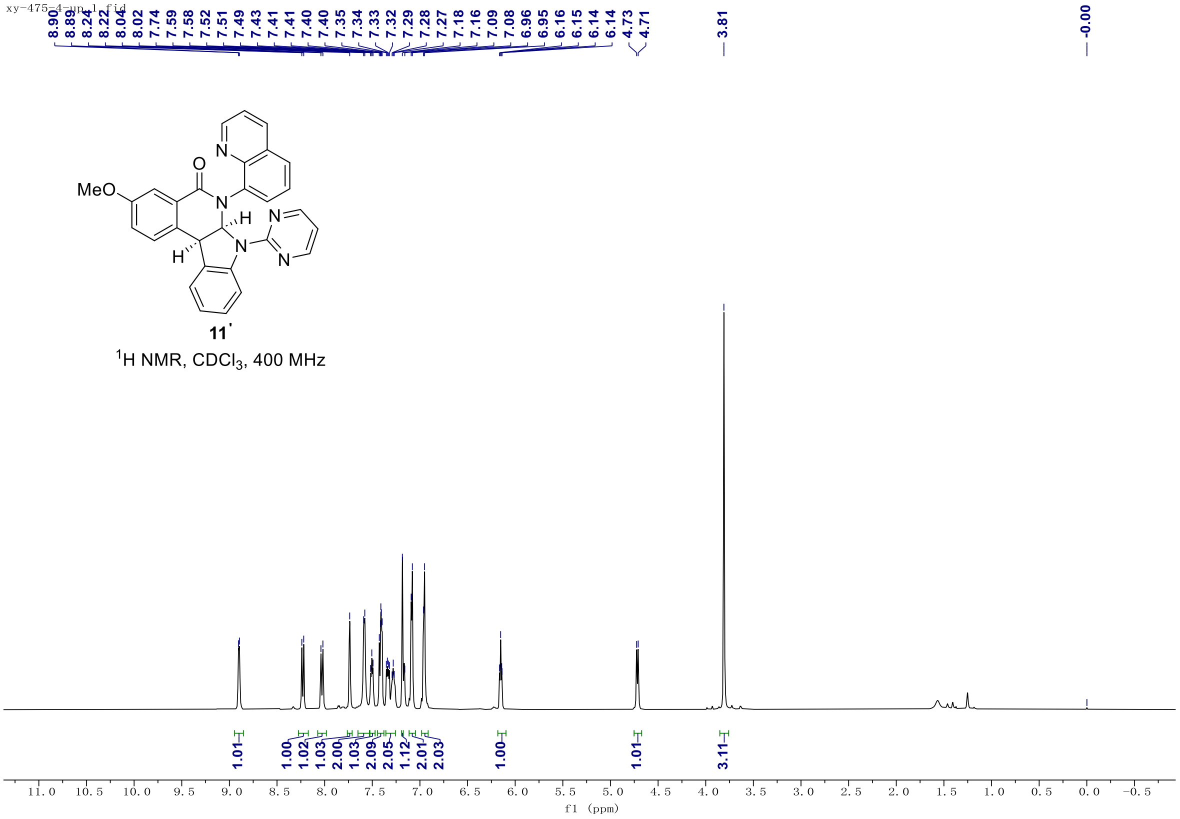
xy-475-4-up.1.fid
8.90 8.89 8.24 8.22 8.04 8.02 7.74 7.59 7.58 7.52 7.51 7.49 7.43 7.41 7.41 7.40 7.40 7.35 7.34 7.33 7.32 7.29 7.28 7.27 7.18 7.16 7.09 7.08 6.96 6.95 6.16 6.15 6.14 6.14 4.73 4.71 3.81 -0.00
MeO
O
N
H
11'
1H NMR, CDCl3, 400 MHz
1.01 1.00 1.02 1.03 2.00 1.03 2.09 2.05 1.12 2.01 2.03 1.00 1.01 3.11
11.0 10.5 10.0 9.5 9.0 8.5 8.0 7.5 7.0 6.5 6.0 5.5 5.0 4.5 4.0 3.5 3.0 2.5 2.0 1.5 1.0 0.5 0.0 -0.5
f1 (ppm)

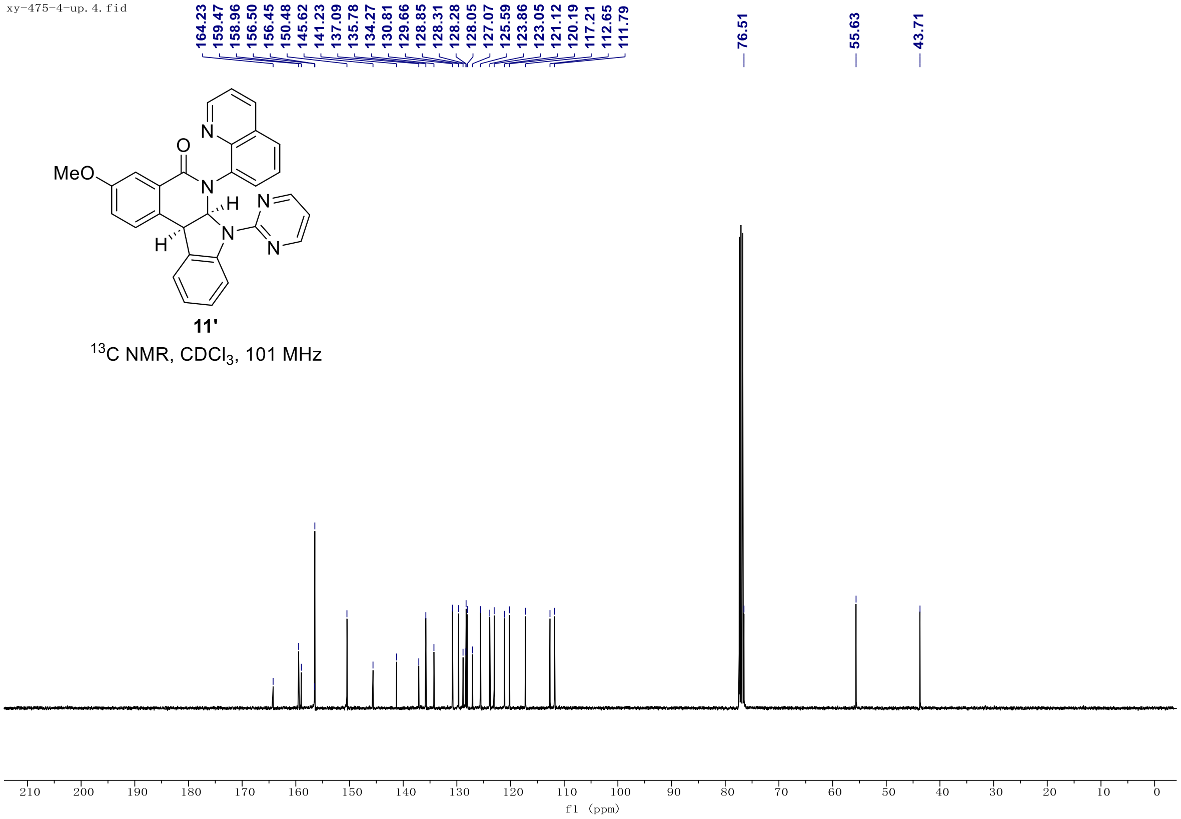
xy-475-4-up.4.fid
164.23 159.47 158.96 156.50 156.45 150.48 145.62 141.23 137.09 135.78 134.27 130.81 129.66 128.85 128.31 128.28 128.05 127.07 125.59 123.86 123.05 121.12 120.19 117.21 112.65 111.79 76.51 55.63 43.71
MeO
O
N
H
11'
13C NMR, CDCl3, 101 MHz
210 200 190 180 170 160 150 140 130 120 110 100 90 80 70 60 50 40 30 20 10 0
f1 (ppm)

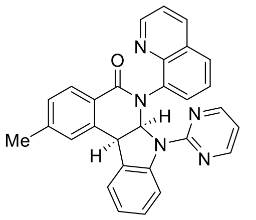

**12**

$^1$H NMR, $CDCl_3$, 400 MHz

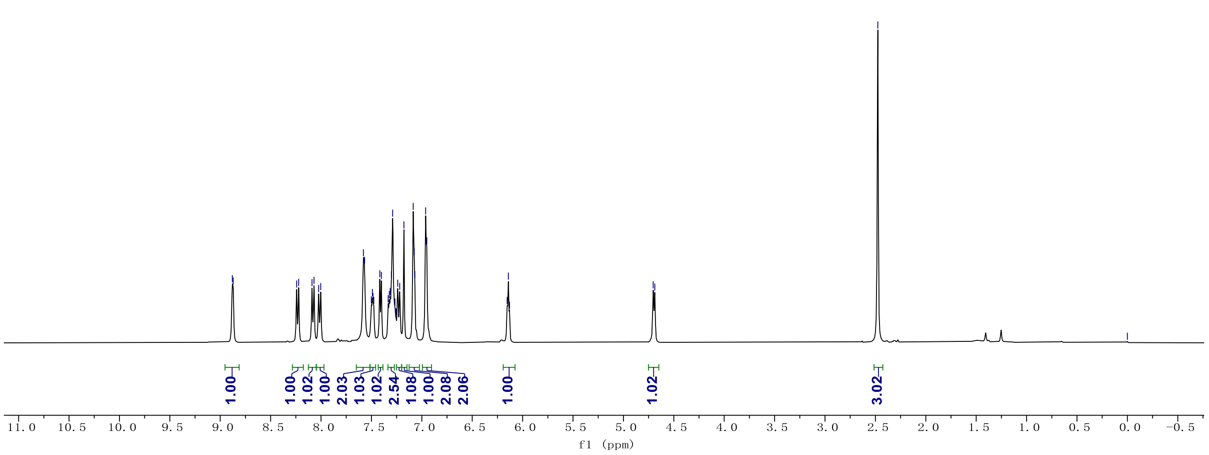


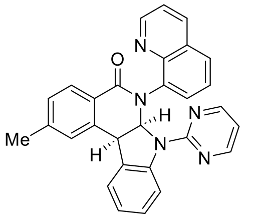

**12**

$^{13}$C NMR, $CDCl_3$, 101 MHz

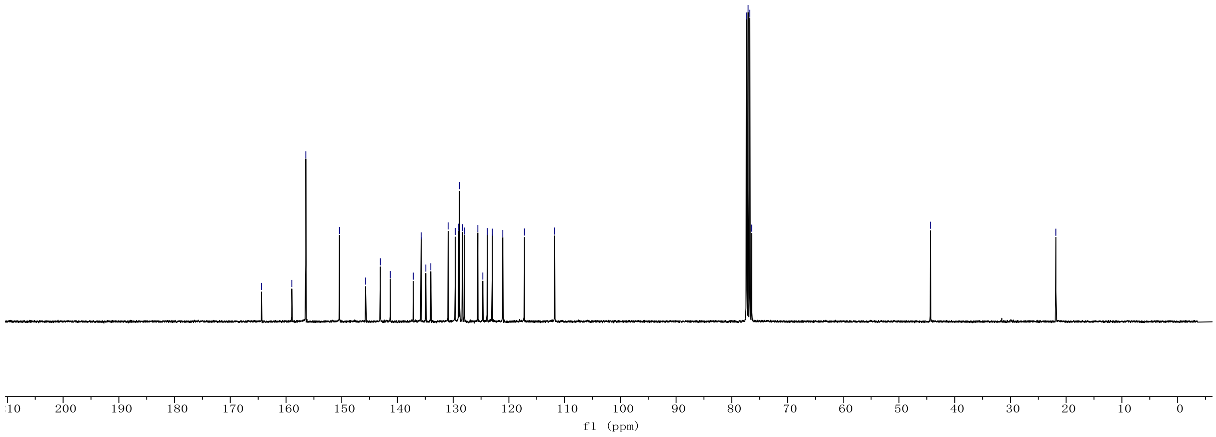

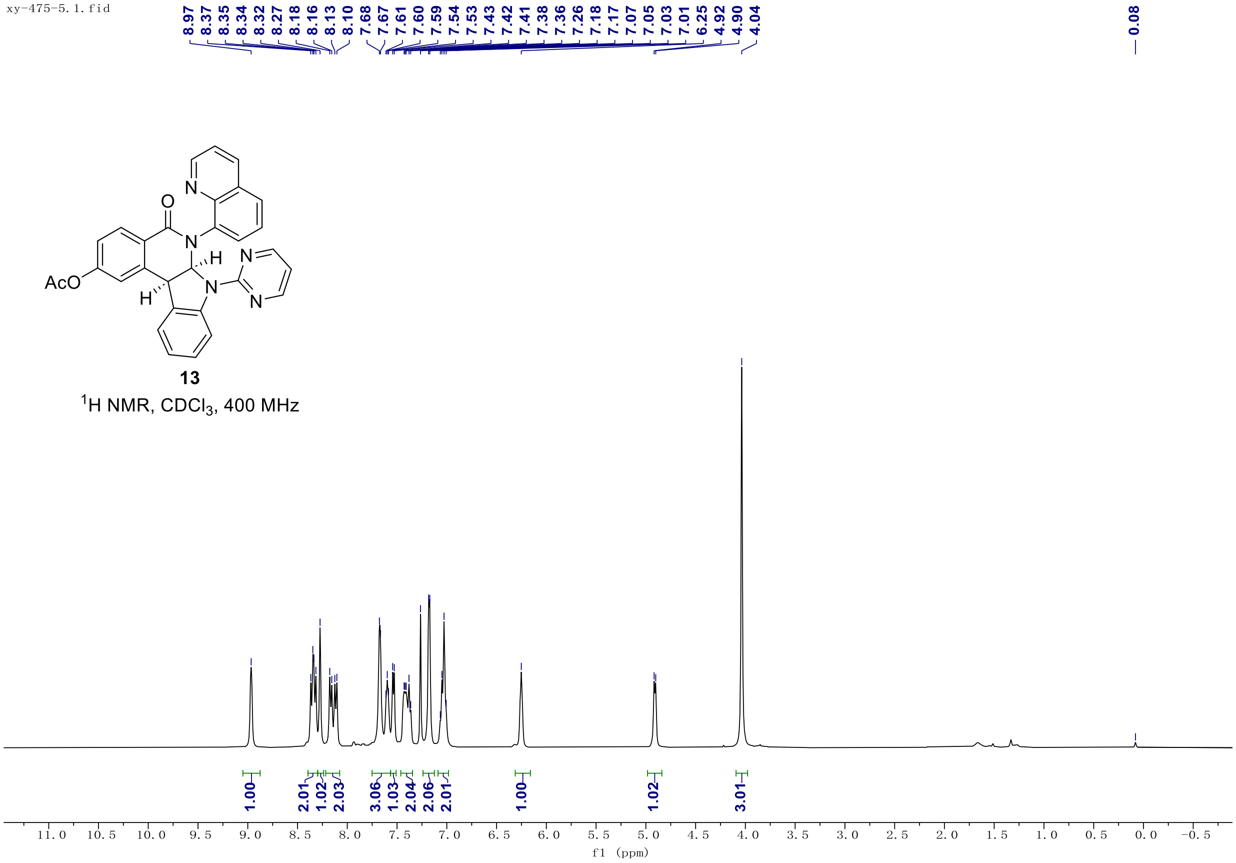

13
$^{1}H$ NMR, $CDCl_3$, 400 MHz

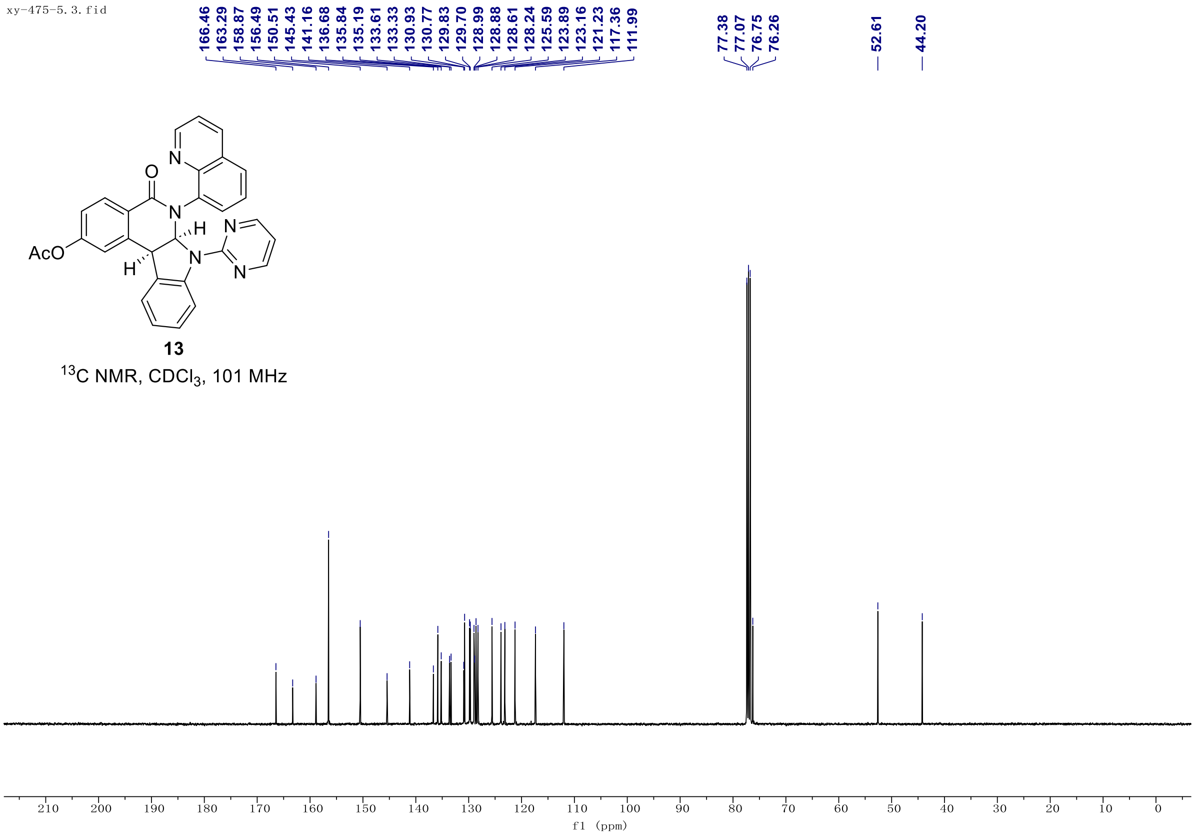

13
$^{13}C$ NMR, $CDCl_3$, 101 MHz

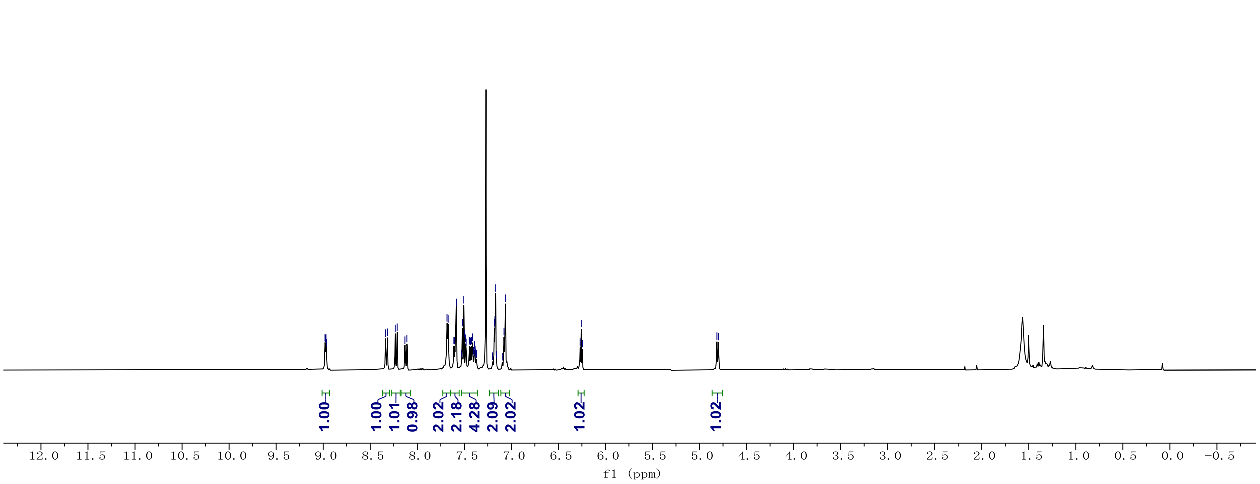


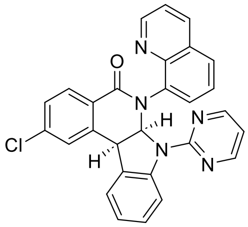


**14**

$^{1}H$ NMR, $CDCl_3$, 400 MHz

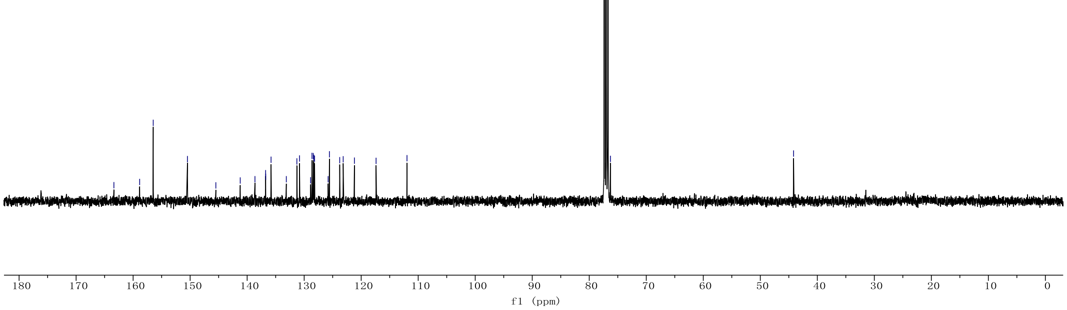


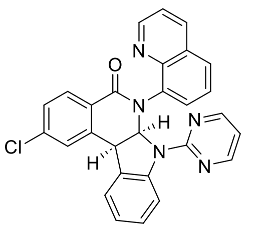


**14**

$^{13}C$ NMR, $CDCl_3$, 101 MHz

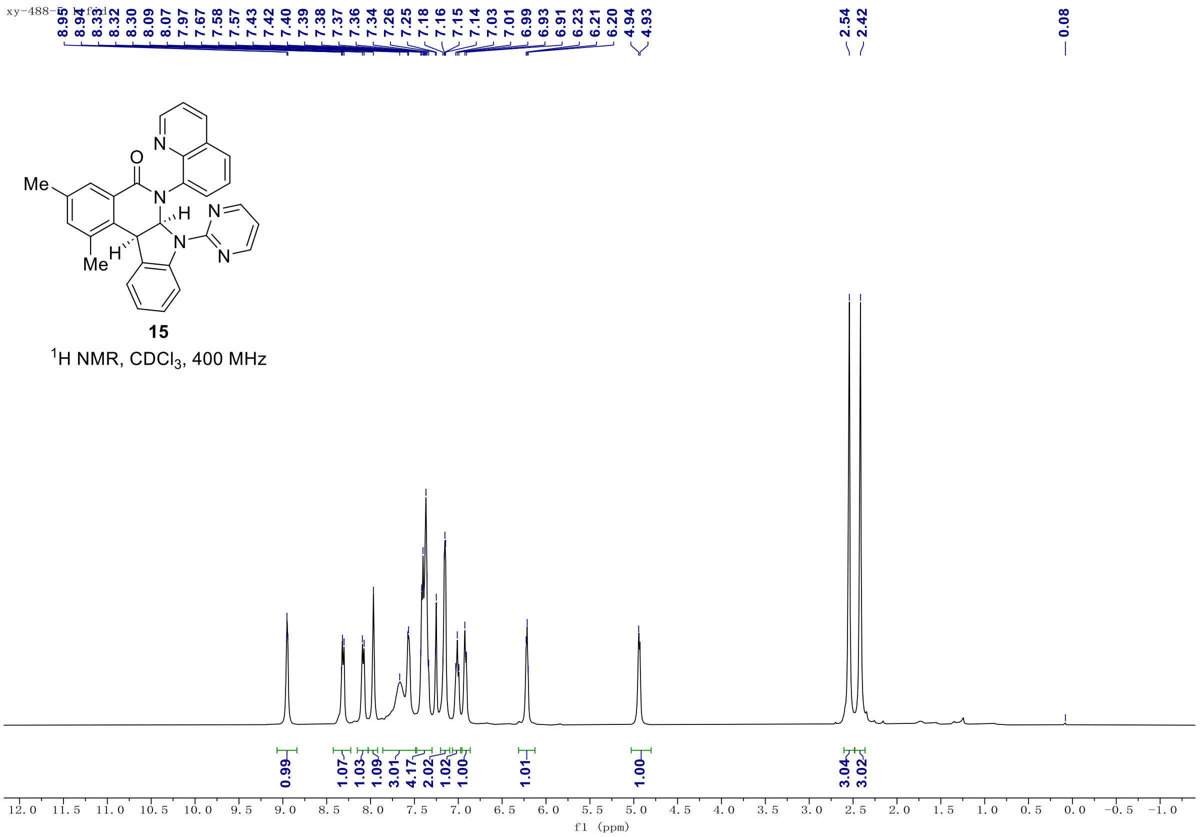
xy-488-5.1.fid
8.95 8.94 8.33 8.32 8.30 8.09 8.07 7.97 7.67 7.58 7.57 7.43 7.42 7.40 7.39 7.38 7.37 7.36 7.34 7.26 7.25 7.18 7.16 7.15 7.14 7.03 7.01 6.99 6.93 6.91 6.23 6.21 6.20 4.94 4.93
2.54 2.42
0.08
Me
Me
O
N
N
N
N
N
H
H
15
$^{1}$H NMR, $CDCl_3$, 400 MHz
0.99 1.07 1.03 1.09 3.01 4.17 2.02 1.02 1.00 1.01 1.00 3.04 3.02
12.0 11.5 11.0 10.5 10.0 9.5 9.0 8.5 8.0 7.5 7.0 6.5 6.0 5.5 5.0 4.5 4.0 3.5 3.0 2.5 2.0 1.5 1.0 0.5 0.0 -0.5 -1.0
f1 (ppm)

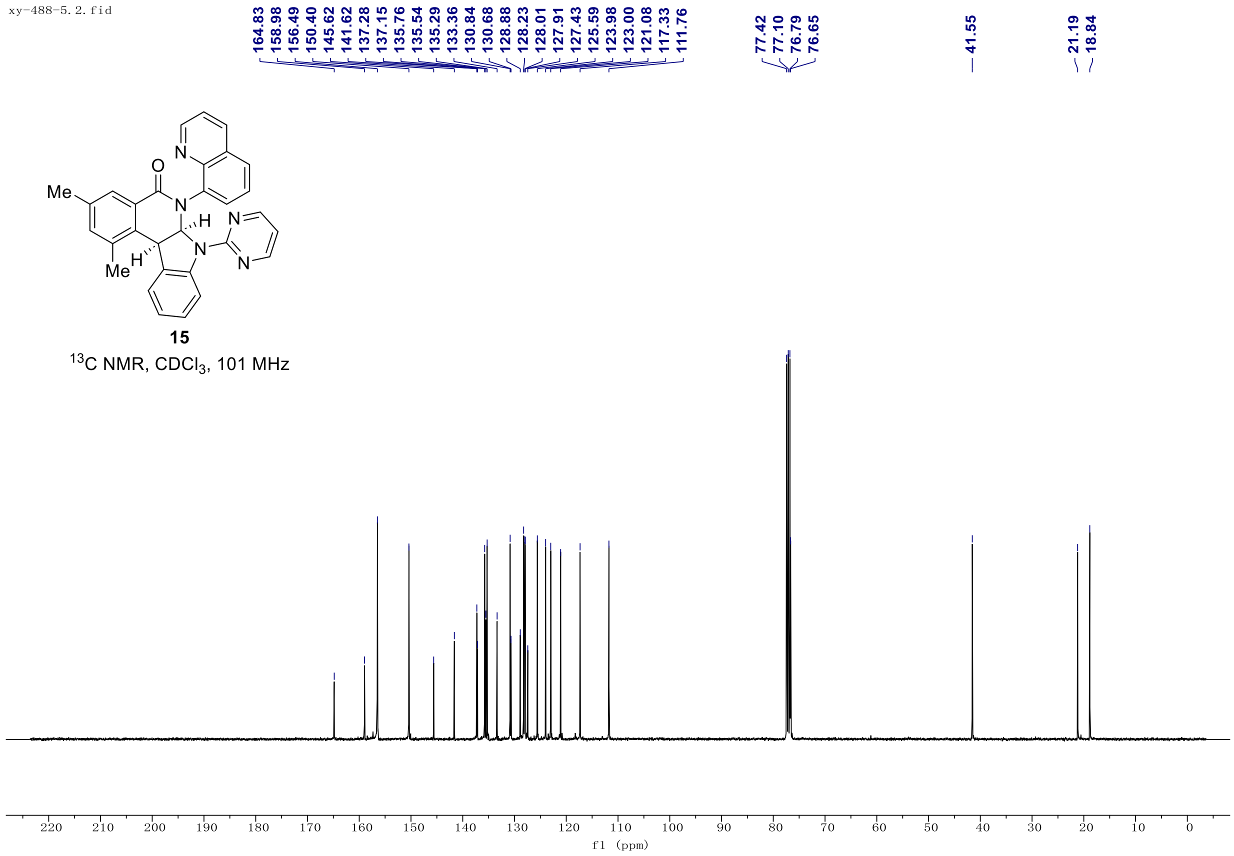
xy-488-5.2.fid
164.83 158.98 156.49 150.40 145.62 141.62 137.28 137.15 135.76 135.54 135.29 133.36 130.84 130.68 128.88 128.23 128.01 127.91 127.43 125.59 123.98 123.00 121.08 117.33 111.76
77.42 77.10 76.79 76.65
41.55
21.19 18.84
Me
Me
O
N
N
N
N
N
H
H
15
$^{13}$C NMR, $CDCl_3$, 101 MHz
220 210 200 190 180 170 160 150 140 130 120 110 100 90 80 70 60 50 40 30 20 10 0
f1 (ppm)

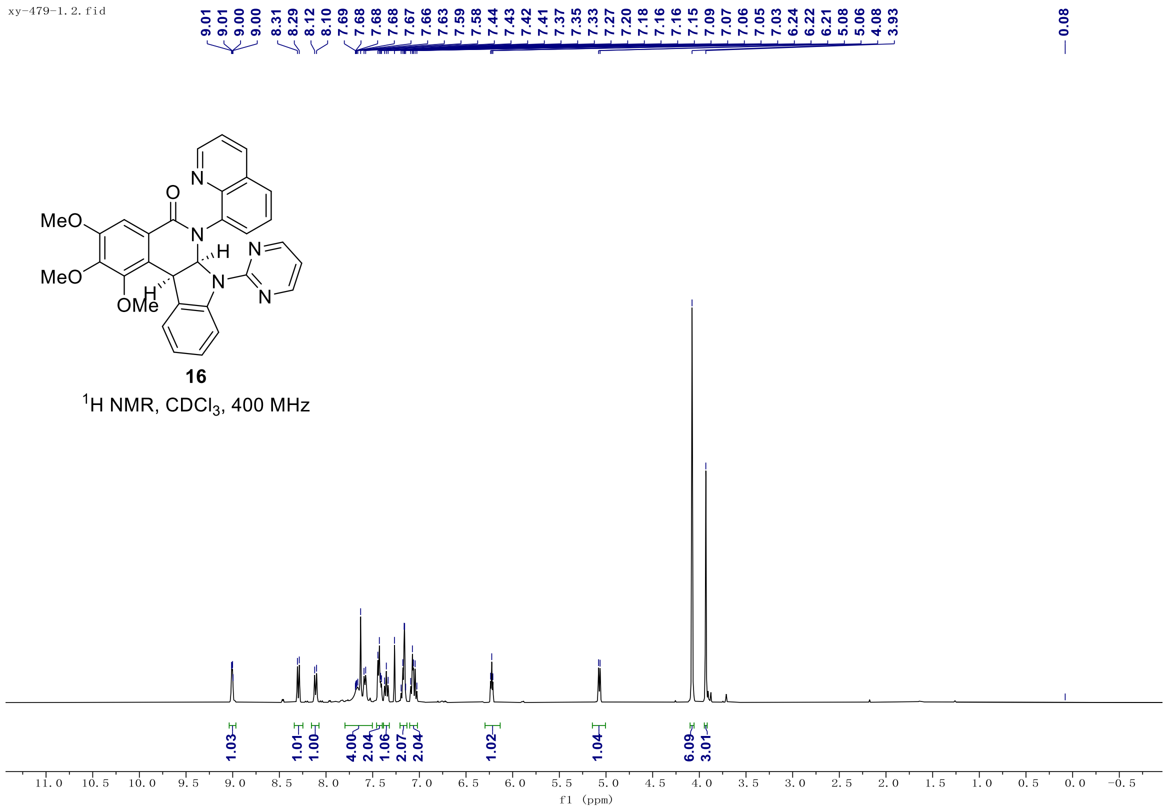
xy-479-1.2.fid
9.01 9.01 9.00 9.00 8.31 8.29 8.12 8.10 7.69 7.68 7.68 7.67 7.66 7.63 7.59 7.58 7.44 7.43 7.42 7.41 7.37 7.35 7.33 7.27 7.20 7.18 7.16 7.16 7.15 7.09 7.07 7.06 7.05 7.03 6.24 6.22 6.21 5.08 5.06 4.08 3.93
0.08
MeO
MeO
OMe
O
N
N
H
H
N
N
N
16
$^{1}H$ NMR, $CDCl_3$, 400 MHz
1.03 1.01 1.00 4.00 2.04 1.06 2.07 2.04 1.02 1.04 6.09 3.01
11.0 10.5 10.0 9.5 9.0 8.5 8.0 7.5 7.0 6.5 6.0 5.5 5.0 4.5 4.0 3.5 3.0 2.5 2.0 1.5 1.0 0.5 0.0 -0.5
f1 (ppm)

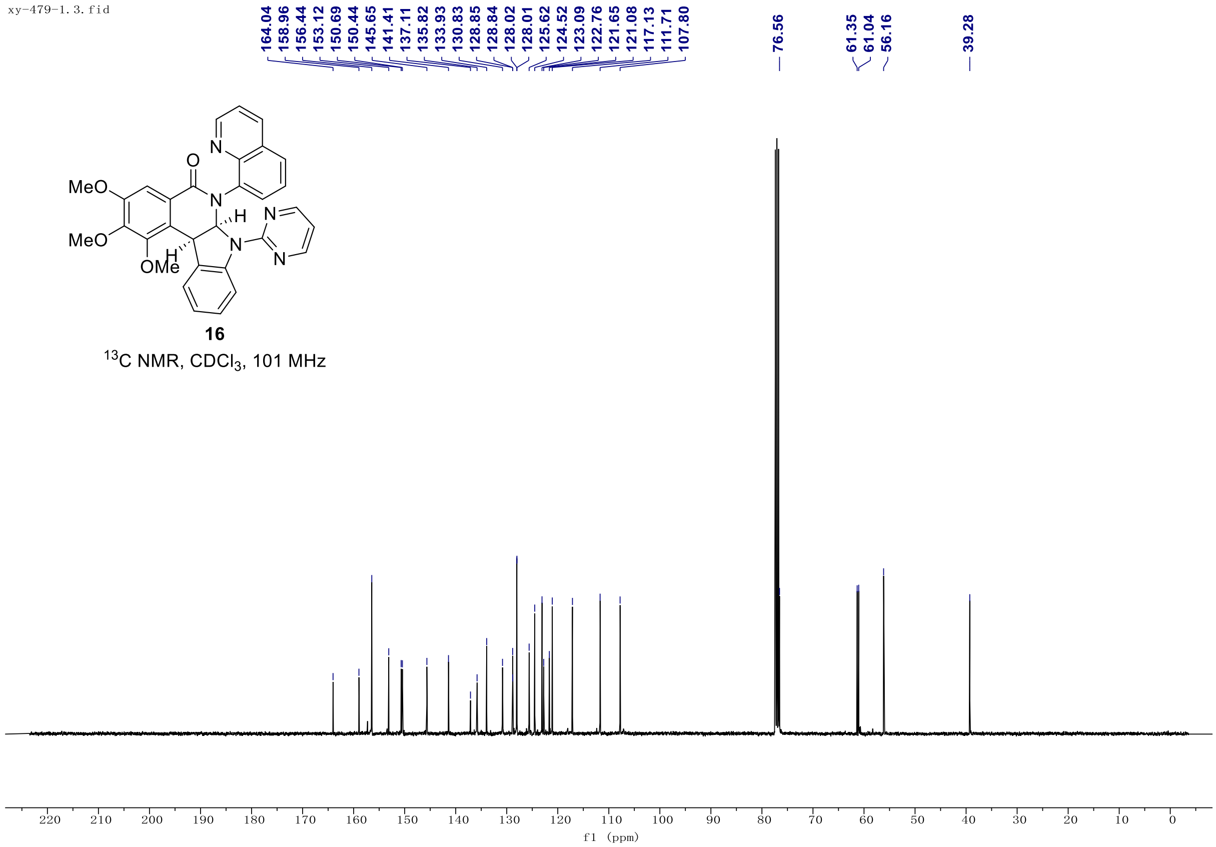
xy-479-1.3.fid
164.04 158.96 156.44 153.12 150.69 150.44 145.65 141.41 137.11 135.82 133.93 130.83 128.85 128.84 128.02 128.01 125.62 124.52 123.09 122.76 121.65 121.08 117.13 111.71 107.80
76.56
61.35 61.04 56.16
39.28
MeO
MeO
OMe
O
N
N
H
H
N
N
N
16
$^{13}C$ NMR, $CDCl_3$, 101 MHz
220 210 200 190 180 170 160 150 140 130 120 110 100 90 80 70 60 50 40 30 20 10 0
f1 (ppm)

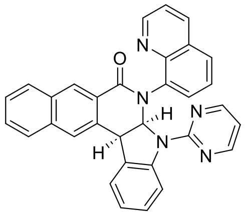

**17**

$^{1}$H NMR, $CDCl_3$, 400 MHz

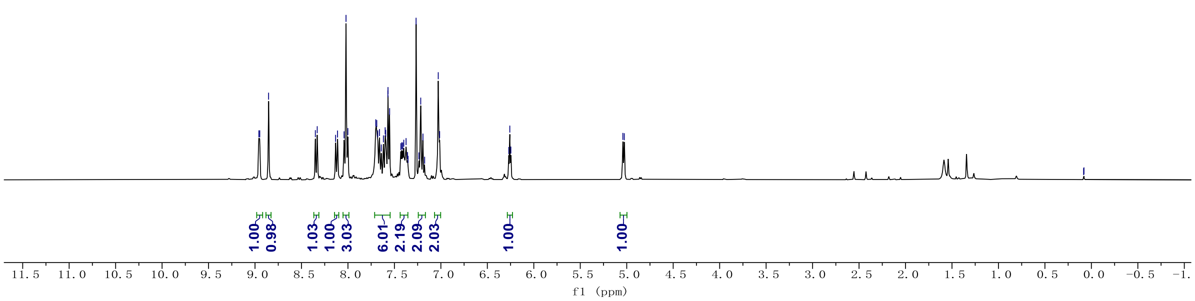


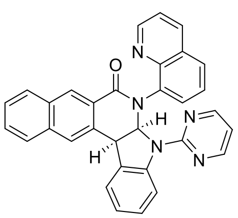

**17**

$^{13}$C NMR, $CDCl_3$, 101 MHz

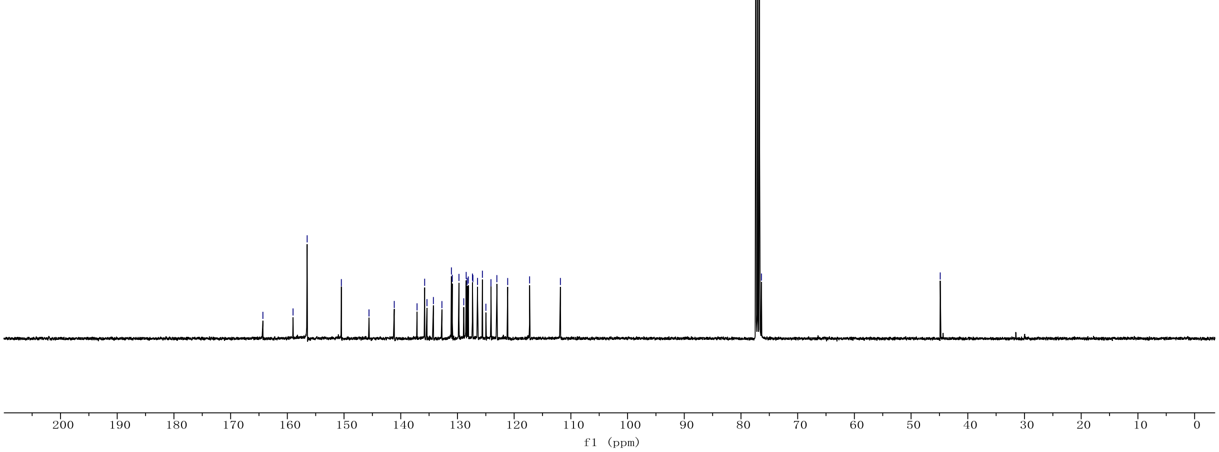

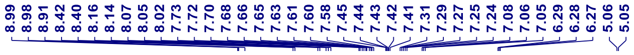


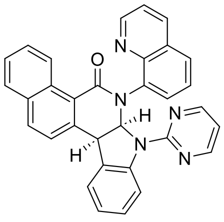


**18**

$^{1}$H NMR, $CDCl_3$, 400 MHz

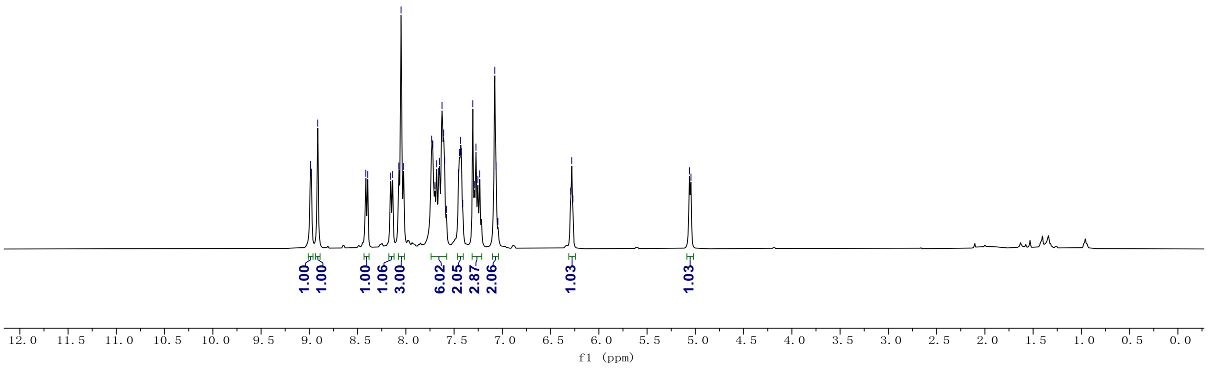


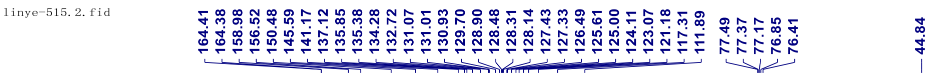


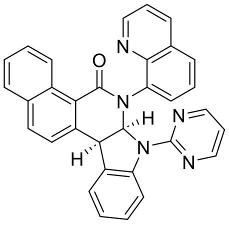


**18**

$^{13}$C NMR, $CDCl_3$, 101 MHz

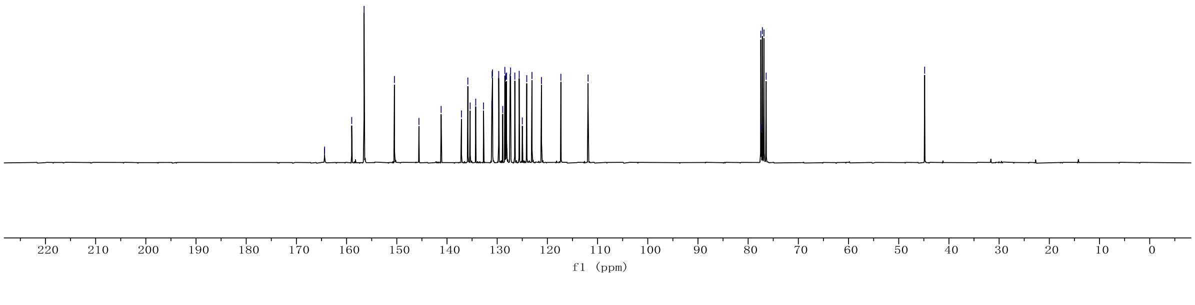

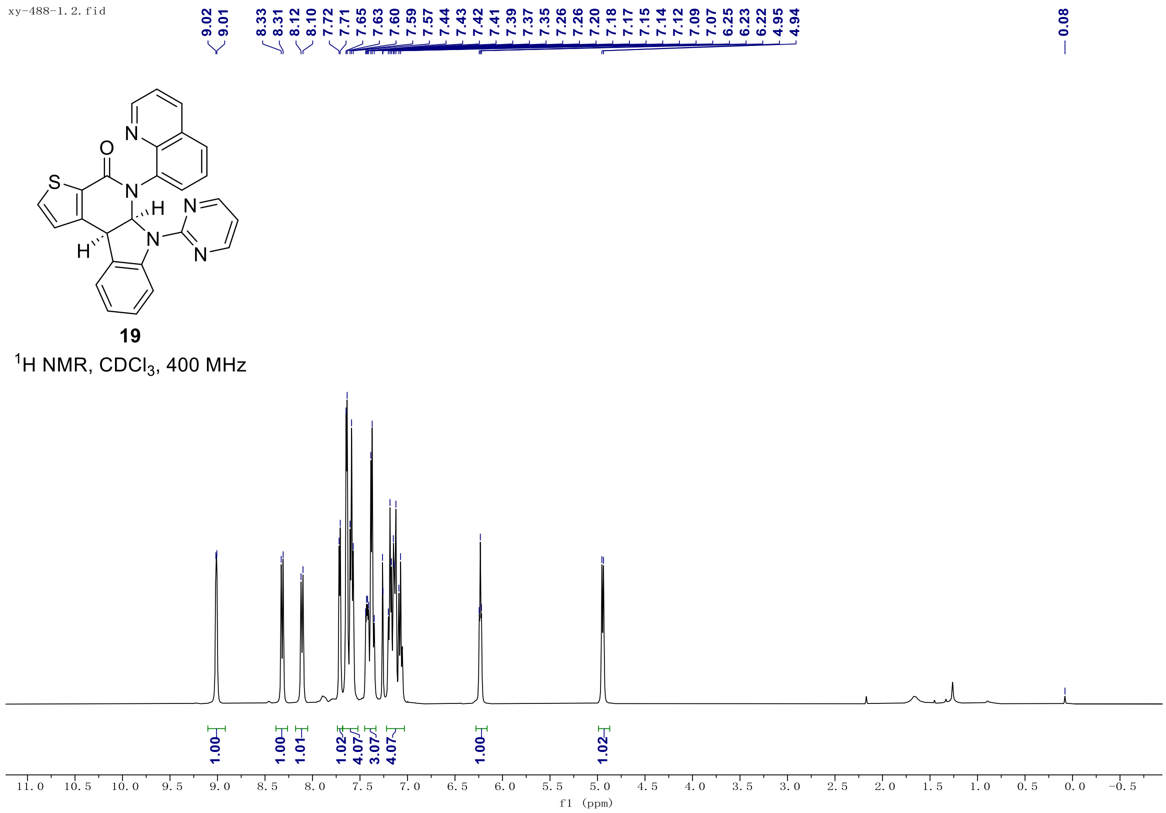


**19**

$^{1}$H NMR, $CDCl_3$, 400 MHz

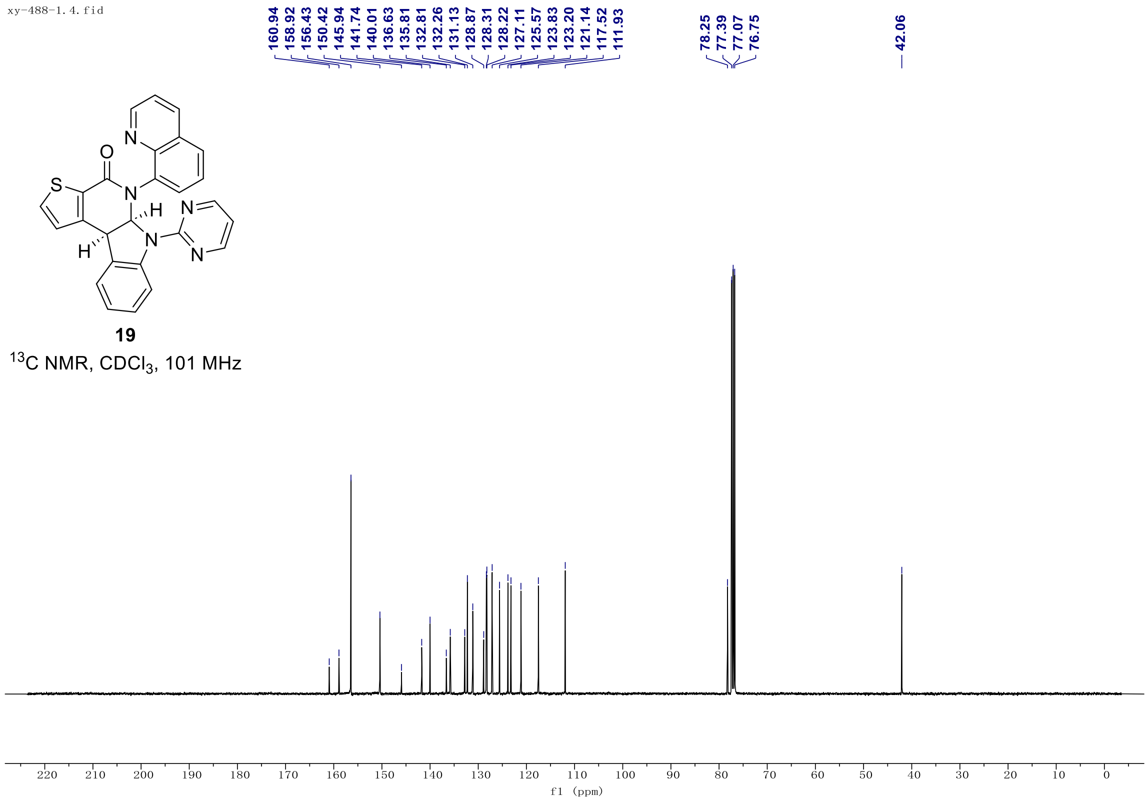


**19**

$^{13}$C NMR, $CDCl_3$, 101 MHz

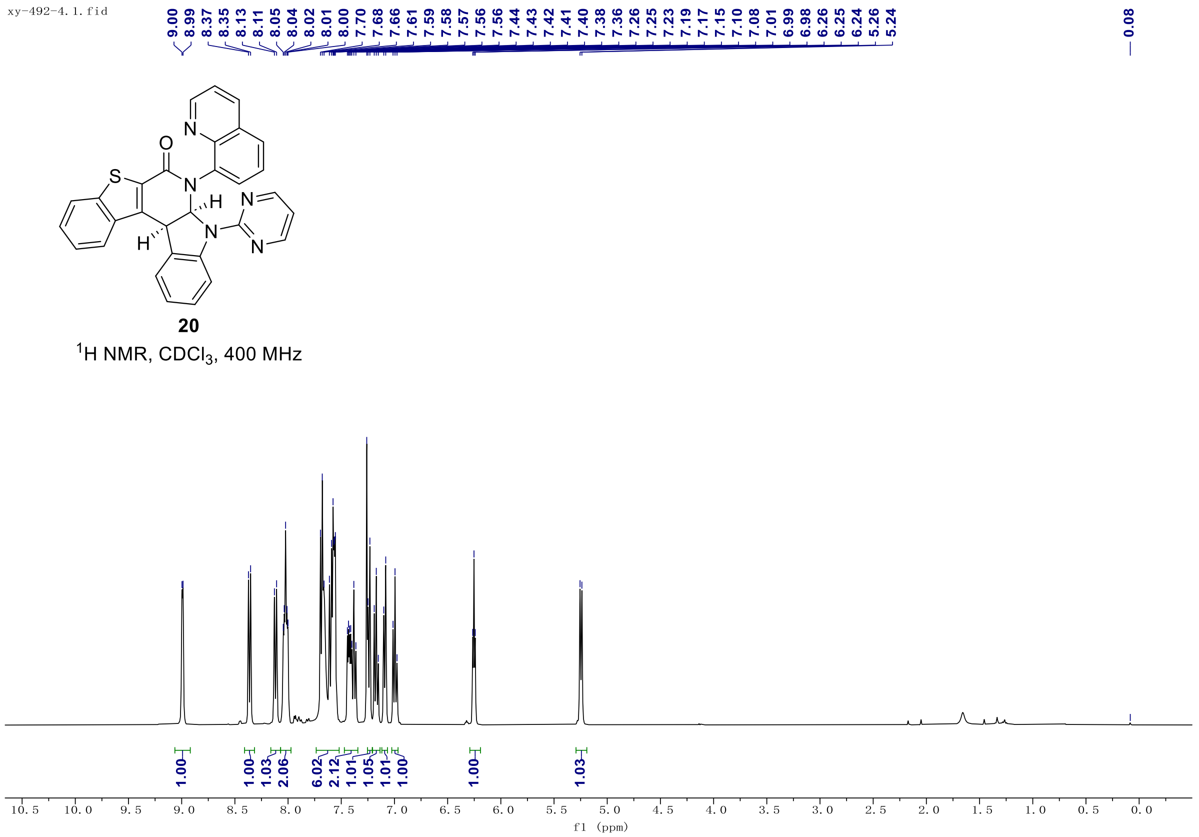


**20**

$^1$H NMR, $CDCl_3$, 400 MHz

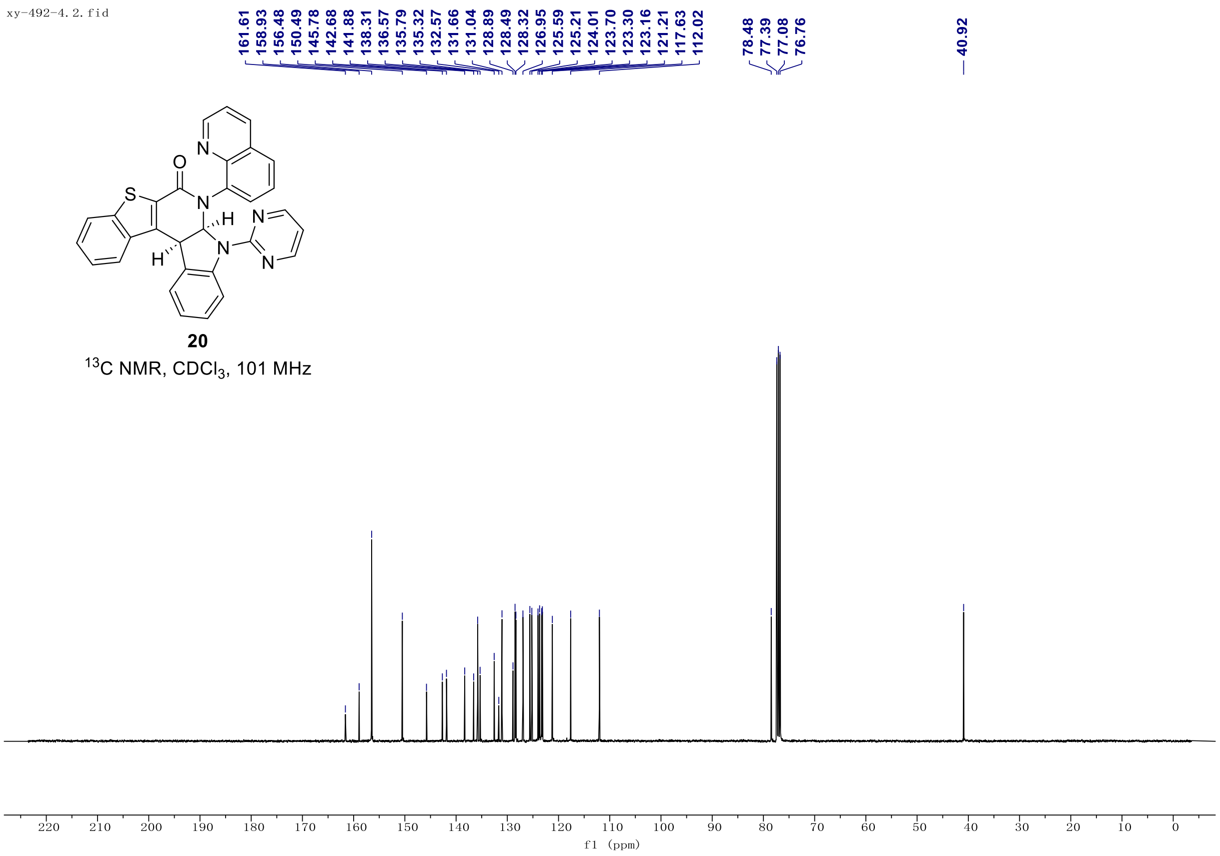


**20**

$^{13}$C NMR, $CDCl_3$, 101 MHz

xy-480-4.1.fid

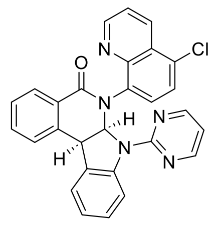

**21**

$^{1}$H NMR, $CDCl_3$, 400 MHz

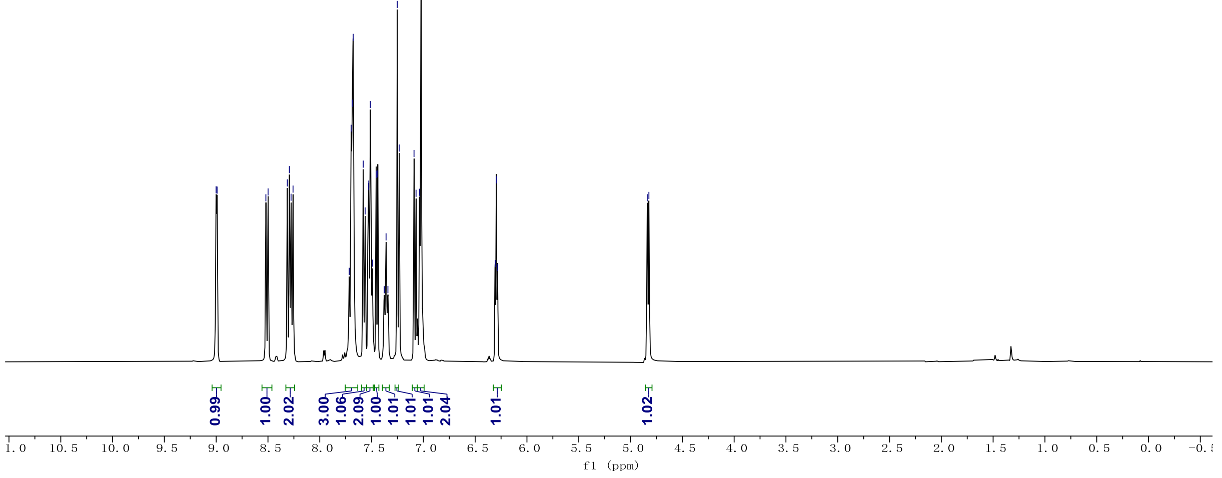


xy-480-4.2.fid

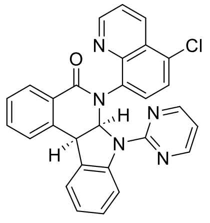

**21**

$^{13}$C NMR, $CDCl_3$, 101 MHz

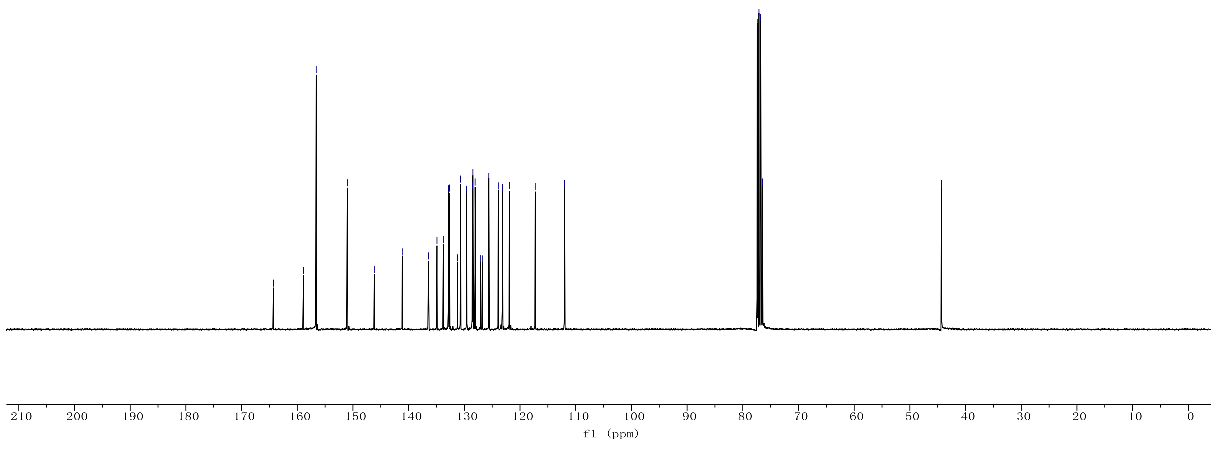

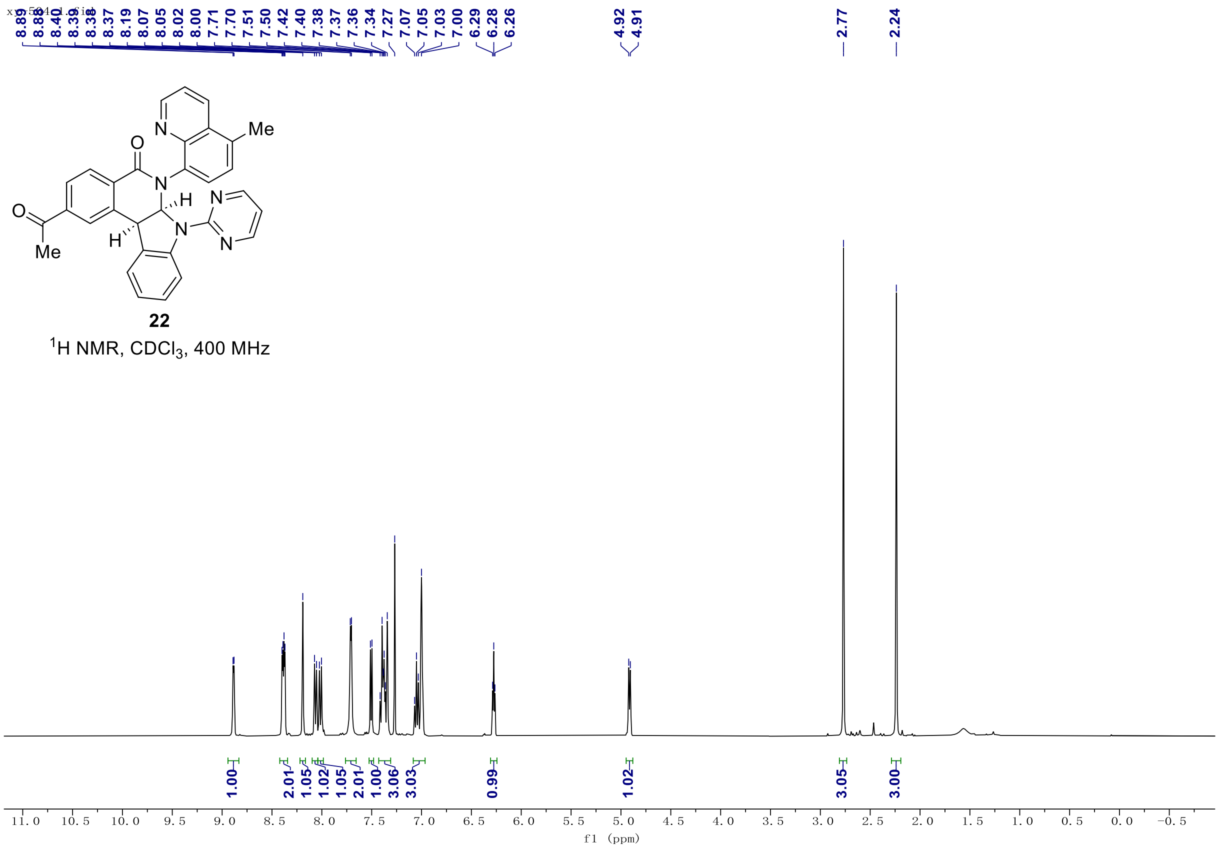

22

$^1$H NMR, $CDCl_3$, 400 MHz

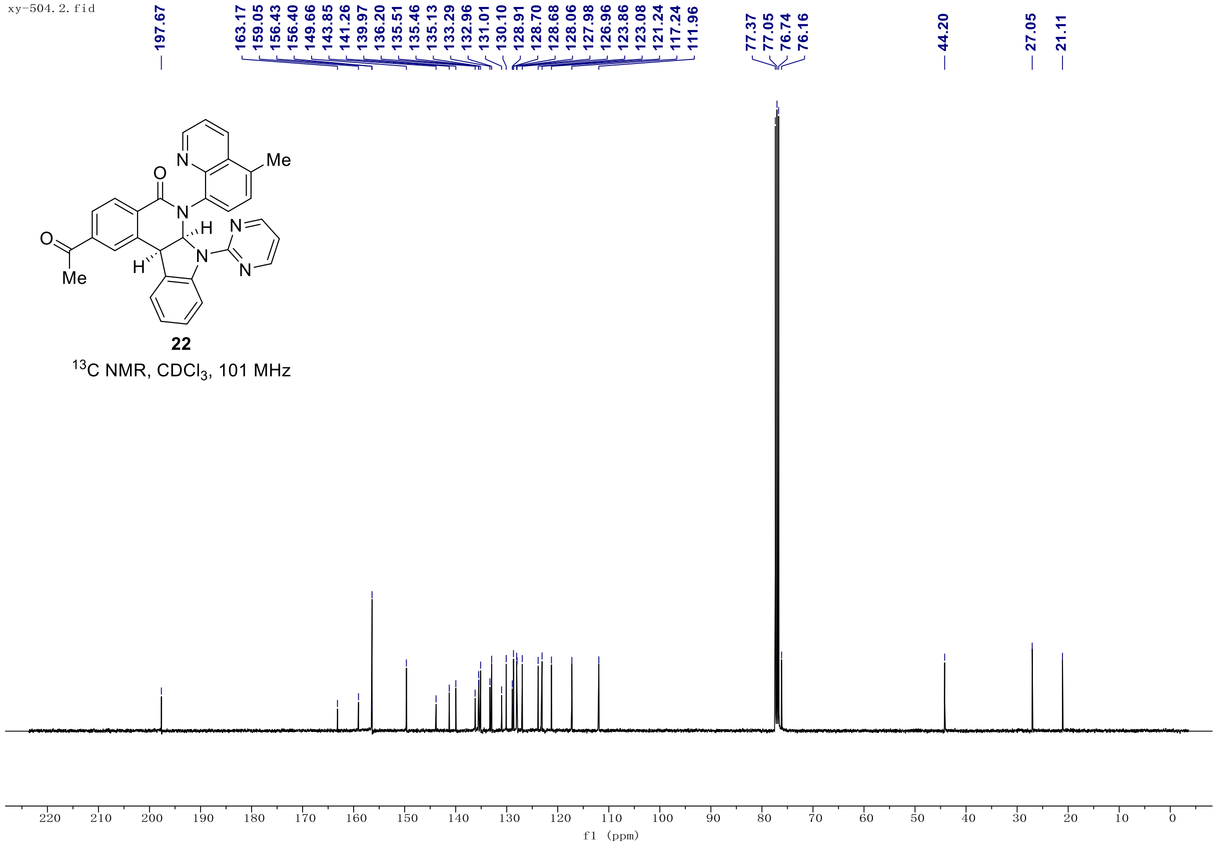

22

$^{13}$C NMR, $CDCl_3$, 101 MHz

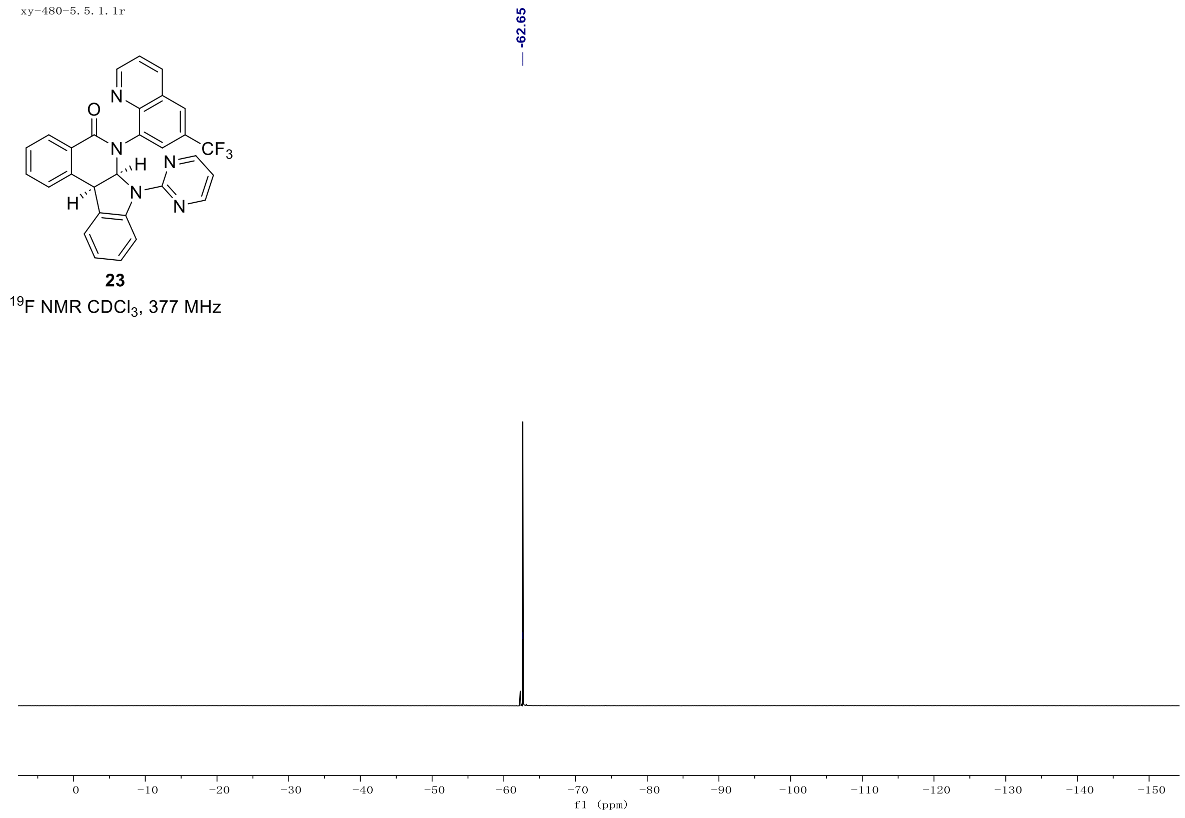
xy-480-5.5.1.1r
-62.65
23
$^{19}$F NMR CDCl$_3$, 377 MHz
f1 (ppm)

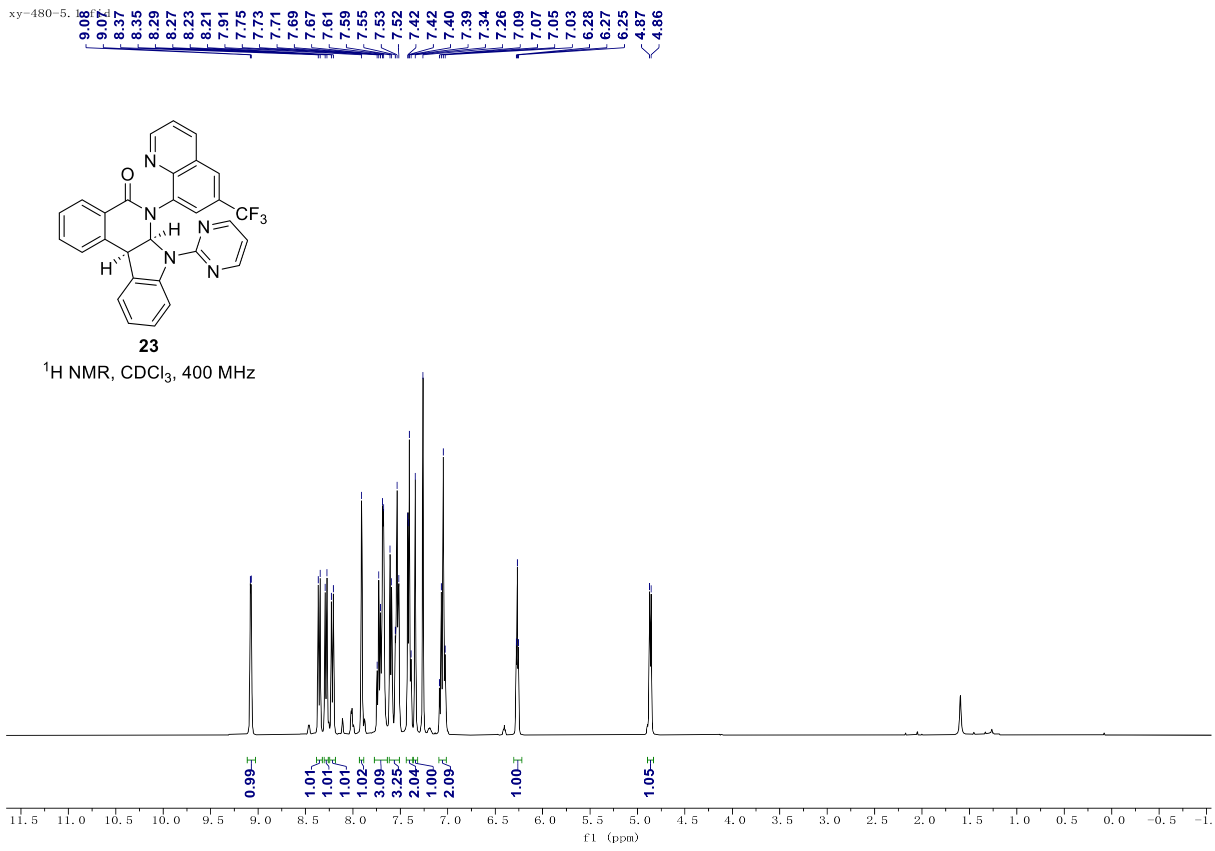
9.08
9.07
8.37
8.35
8.29
8.27
8.23
8.21
7.91
7.75
7.73
7.71
7.69
7.67
7.61
7.59
7.55
7.53
7.52
7.42
7.42
7.40
7.39
7.34
7.26
7.09
7.07
7.05
7.03
6.28
6.27
6.25
4.87
4.86
23
$^{1}$H NMR, CDCl$_3$, 400 MHz
0.99
1.01
1.01
1.01
1.02
3.09
3.25
2.04
1.00
2.09
1.00
1.05
f1 (ppm)

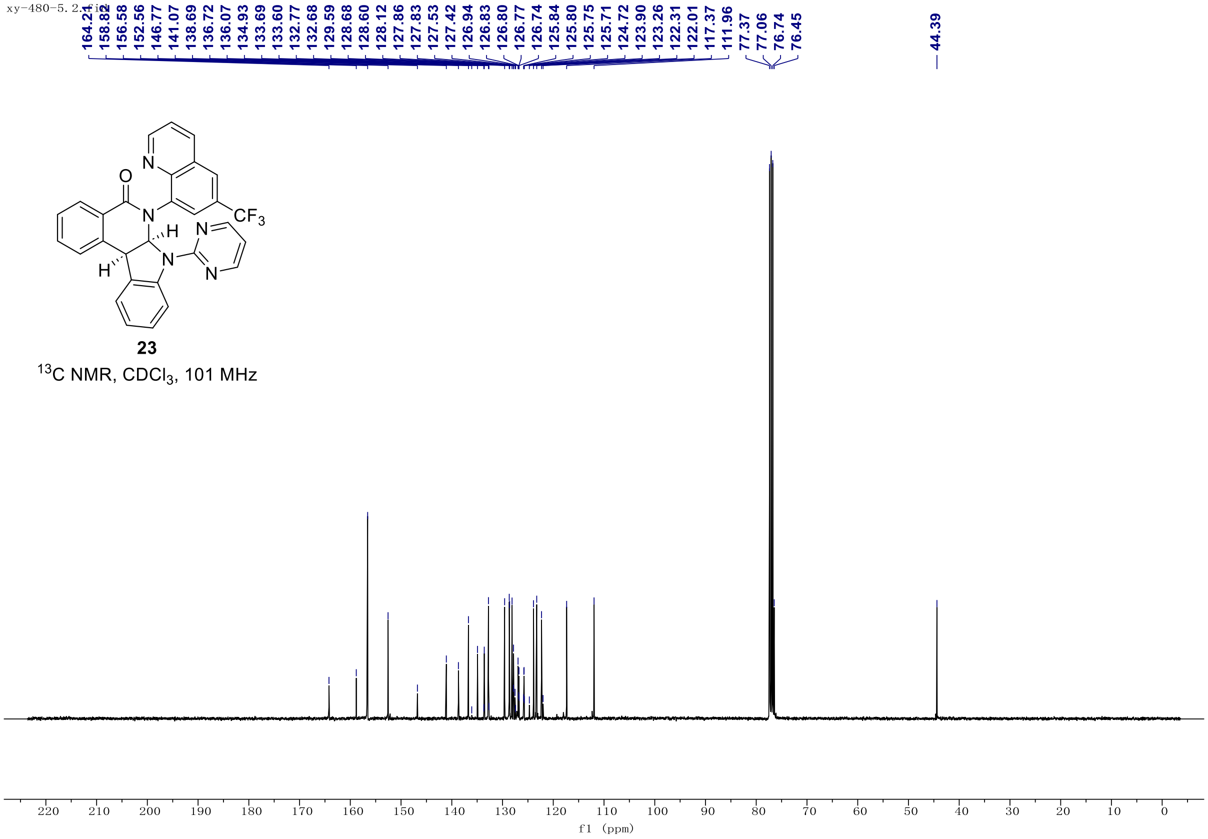


**23**

$^{13}$C NMR, $CDCl_3$, 101 MHz

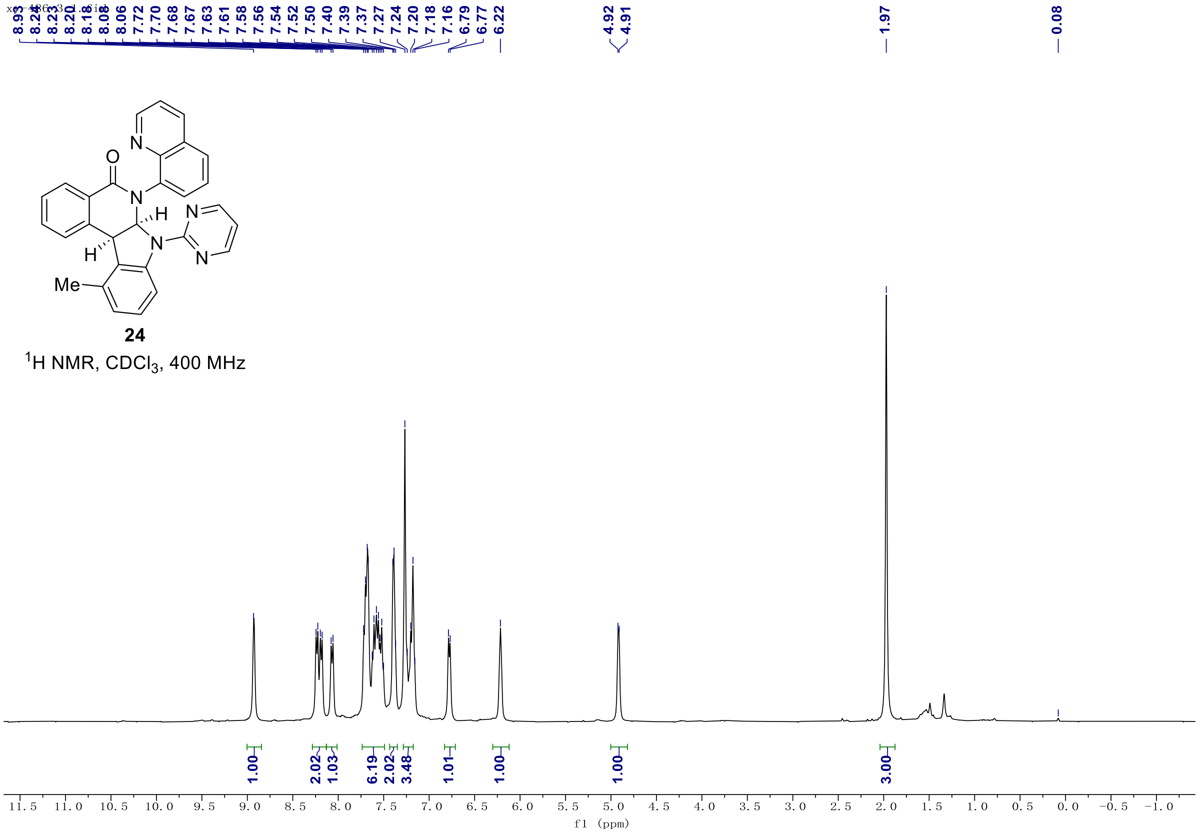


**24**

$^{1}$H NMR, $CDCl_3$, 400 MHz

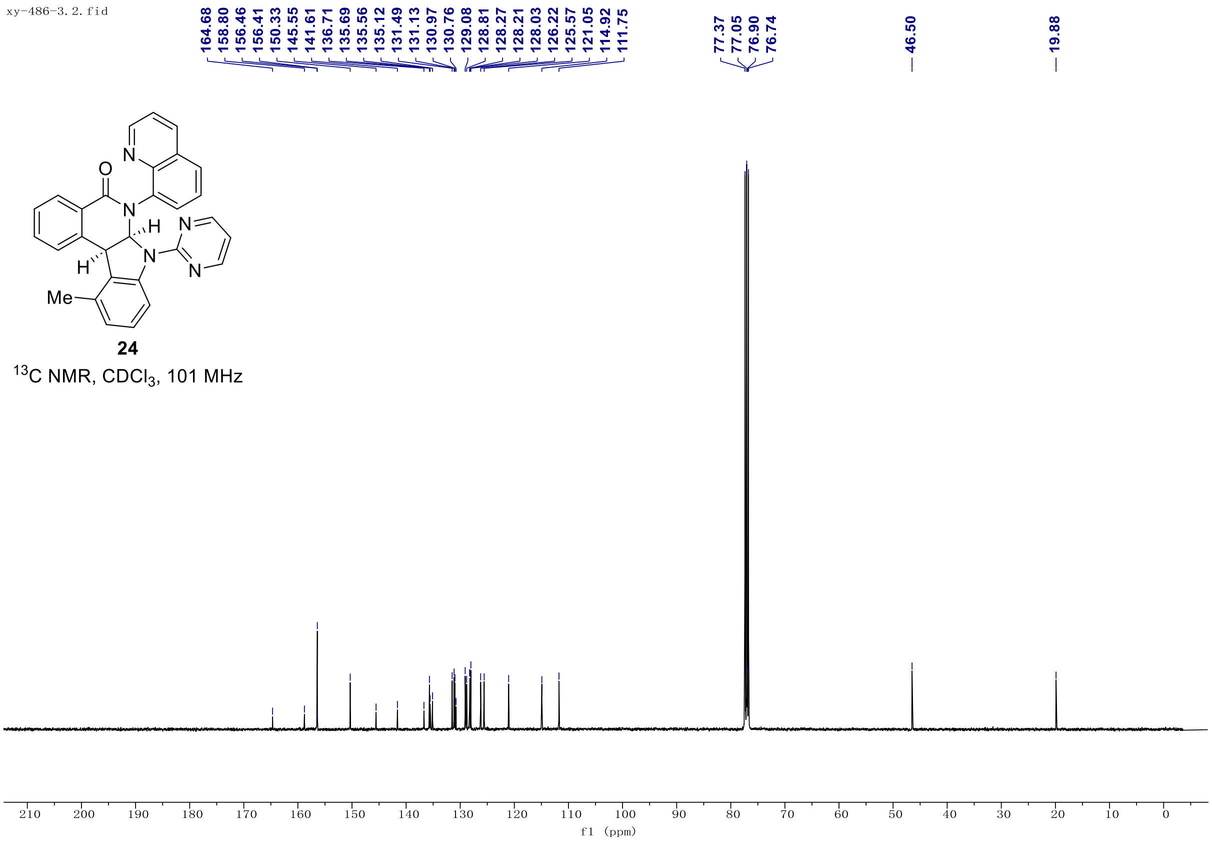


**24**

$^{13}C$ NMR, $CDCl_3$, 101 MHz

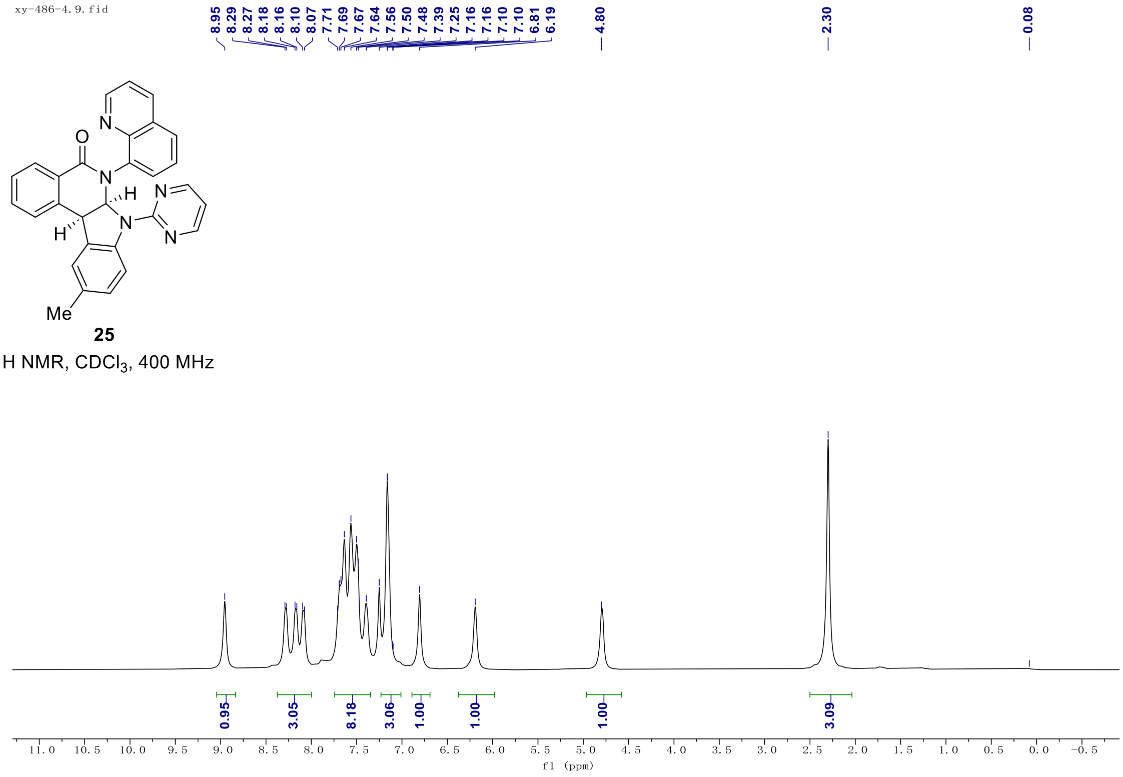


**25**

$^{1}H$ NMR, $CDCl_3$, 400 MHz

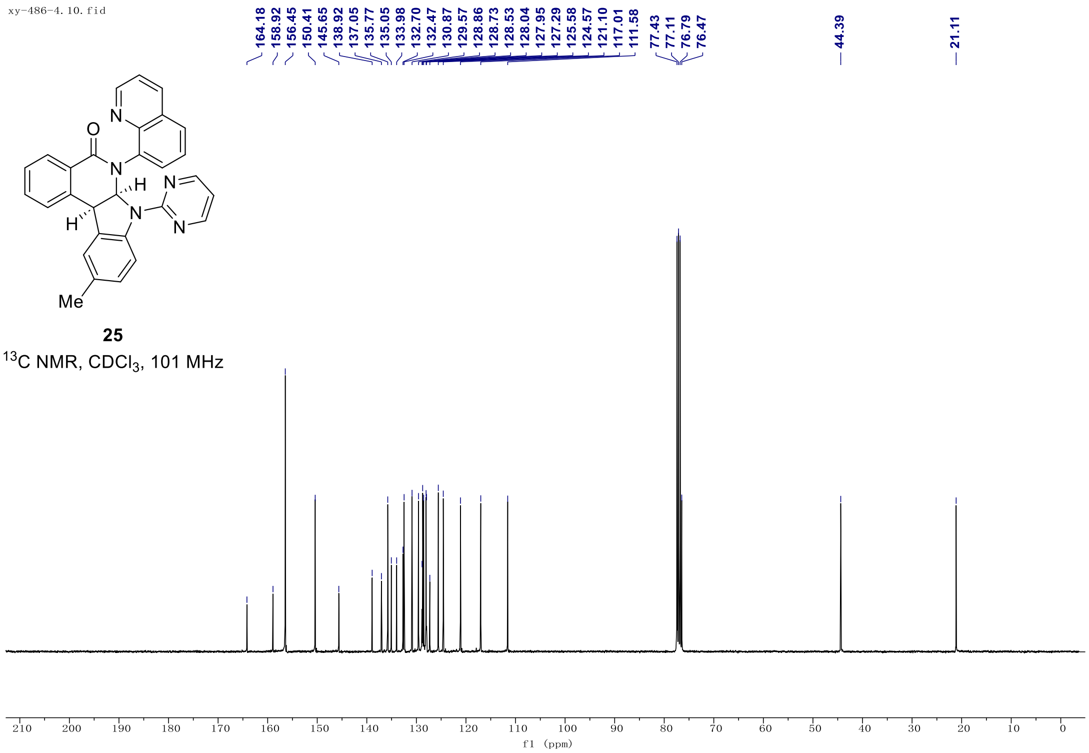
xy-486-4.10.fid
164.18
158.92
156.45
150.41
145.65
138.92
137.05
135.77
135.05
133.98
132.70
132.47
130.87
129.57
128.86
128.73
128.53
128.04
127.95
127.29
125.58
124.57
121.10
117.01
111.58
77.43
77.11
76.79
76.47
44.39
21.11
O
N
N
H
H
N
N
N
Me
25
13C NMR, CDCl3, 101 MHz
210 200 190 180 170 160 150 140 130 120 110 100 90 80 70 60 50 40 30 20 10 0
f1 (ppm)

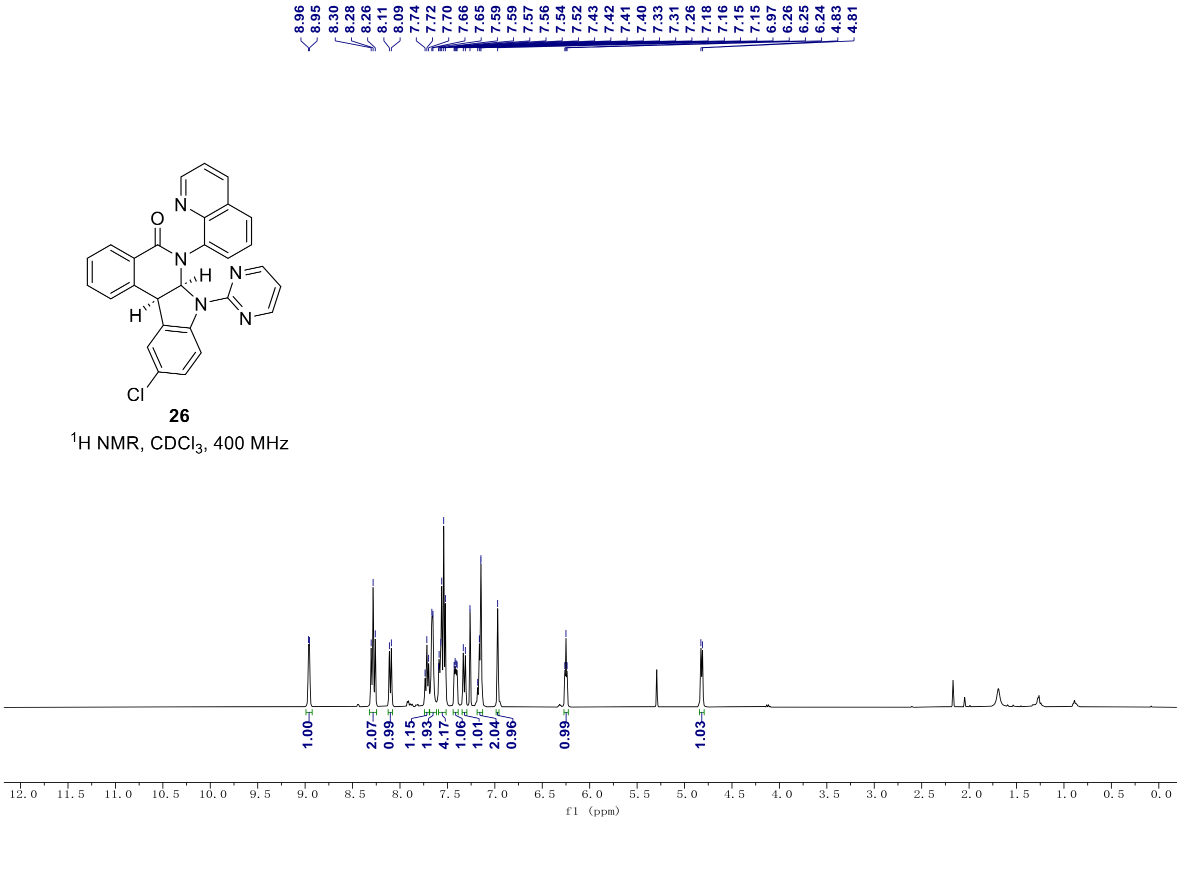
8.96
8.95
8.30
8.28
8.26
8.11
8.09
7.74
7.72
7.70
7.66
7.65
7.59
7.59
7.57
7.56
7.54
7.52
7.43
7.42
7.41
7.40
7.33
7.31
7.26
7.18
7.16
7.15
7.15
6.97
6.26
6.25
6.24
4.83
4.81
O
N
N
H
H
N
N
N
Cl
26
1H NMR, CDCl3, 400 MHz
1.00
2.07
0.99
1.15
1.93
4.17
1.06
1.01
2.04
0.96
0.99
1.03
12.0 11.5 11.0 10.5 10.0 9.5 9.0 8.5 8.0 7.5 7.0 6.5 6.0 5.5 5.0 4.5 4.0 3.5 3.0 2.5 2.0 1.5 1.0 0.5 0.0
f1 (ppm)

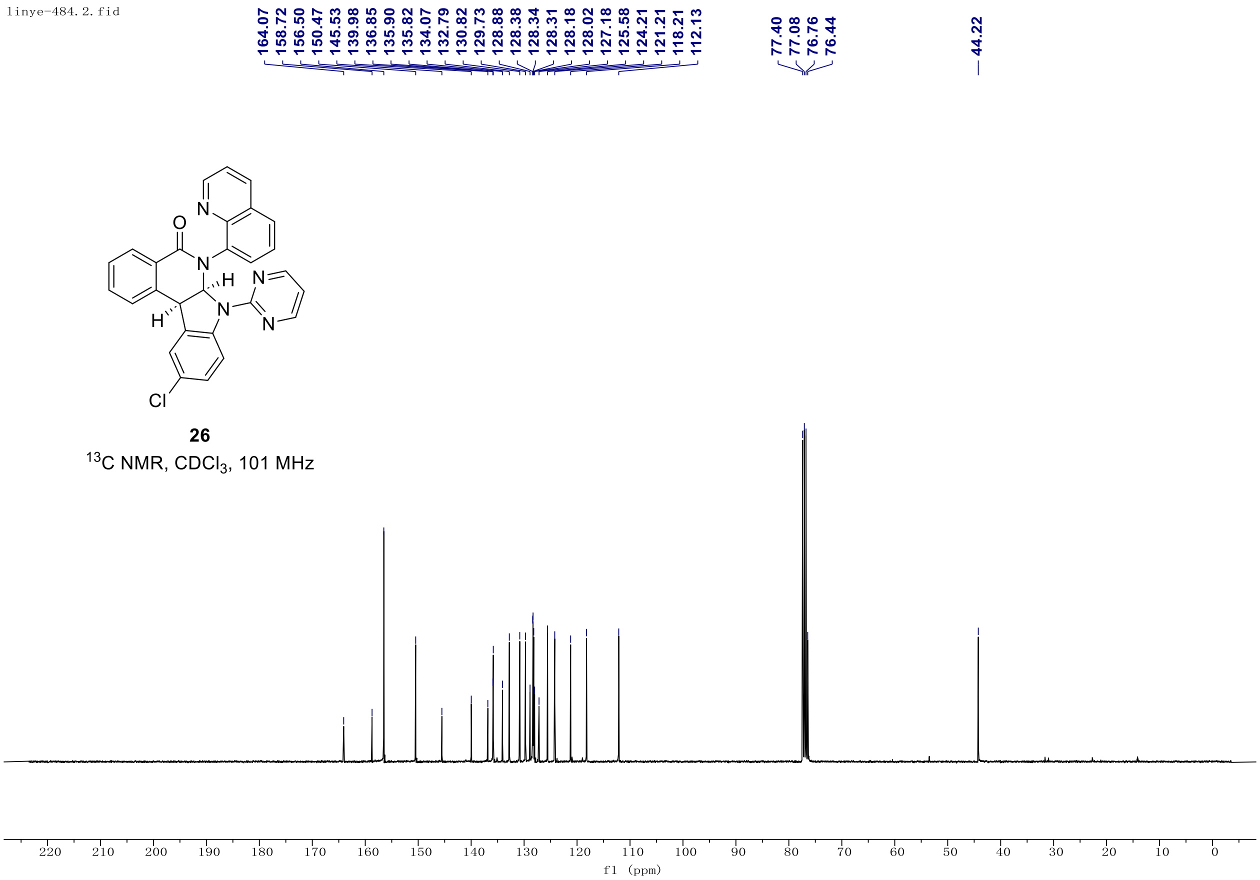


**26**

$^{13}$C NMR, $CDCl_3$, 101 MHz

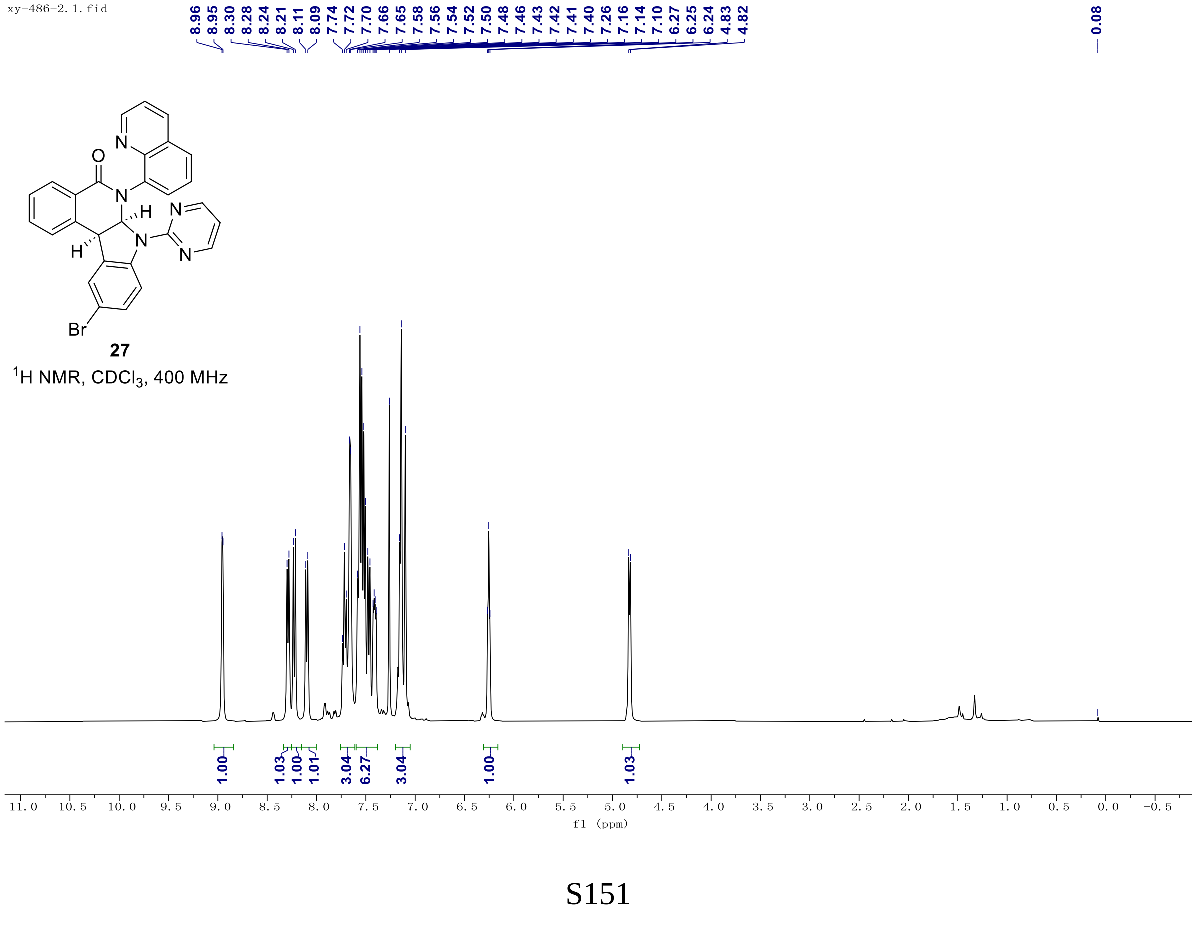


**27**

$^{1}$H NMR, $CDCl_3$, 400 MHz

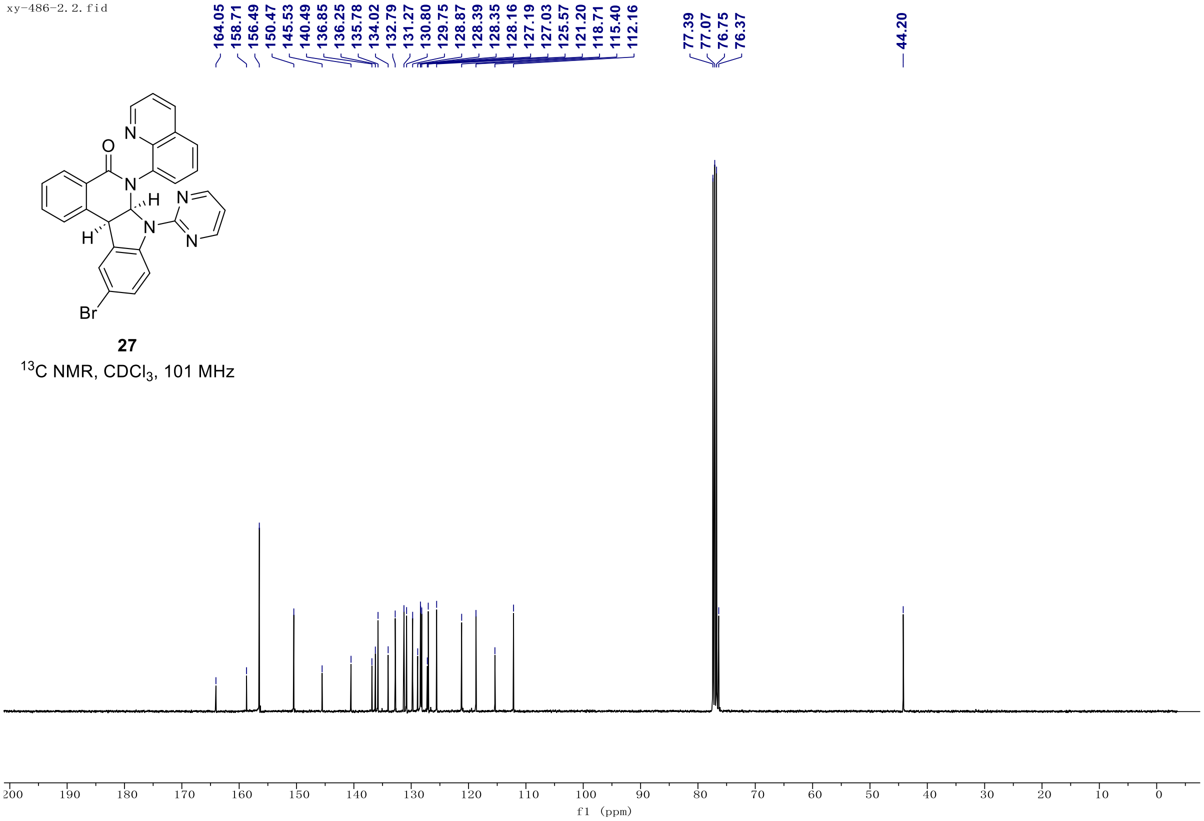


**27**

$^{13}C$ NMR, $CDCl_3$, 101 MHz

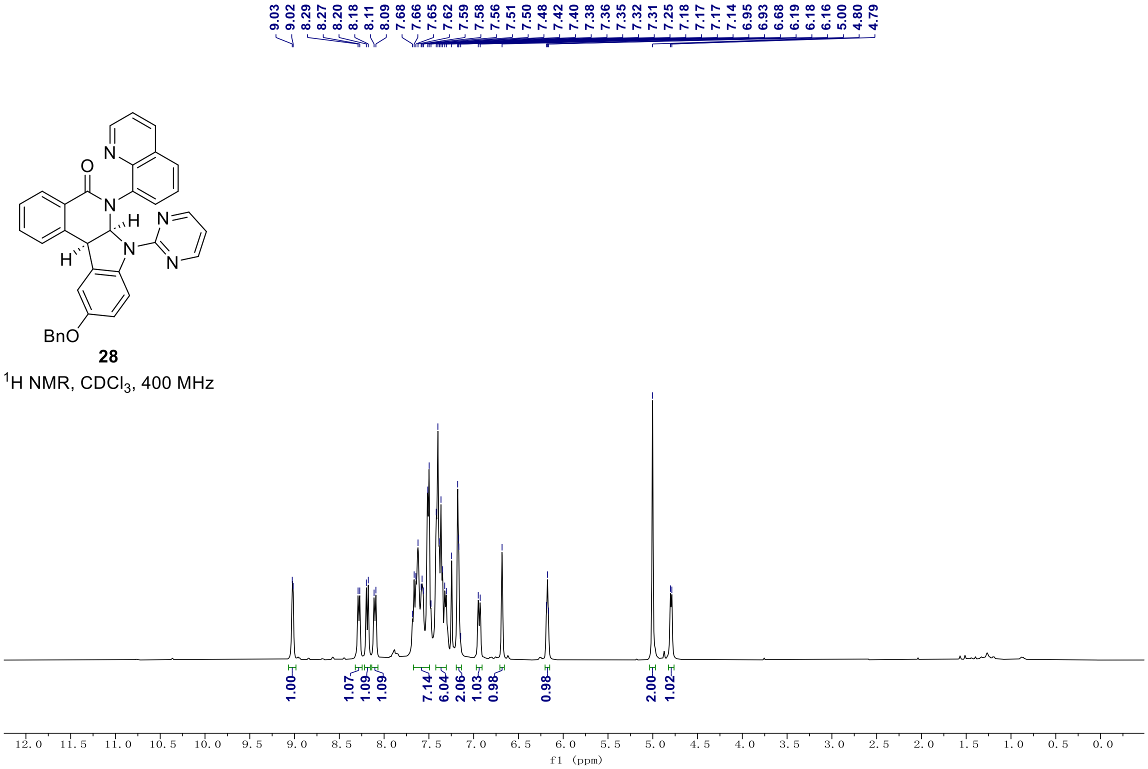


**28**

$^{1}H$ NMR, $CDCl_3$, 400 MHz

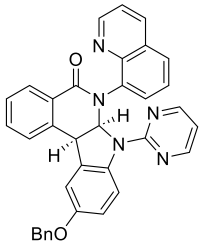

**28**

$^{13}C$ NMR, $CDCl_3$, 101 MHz

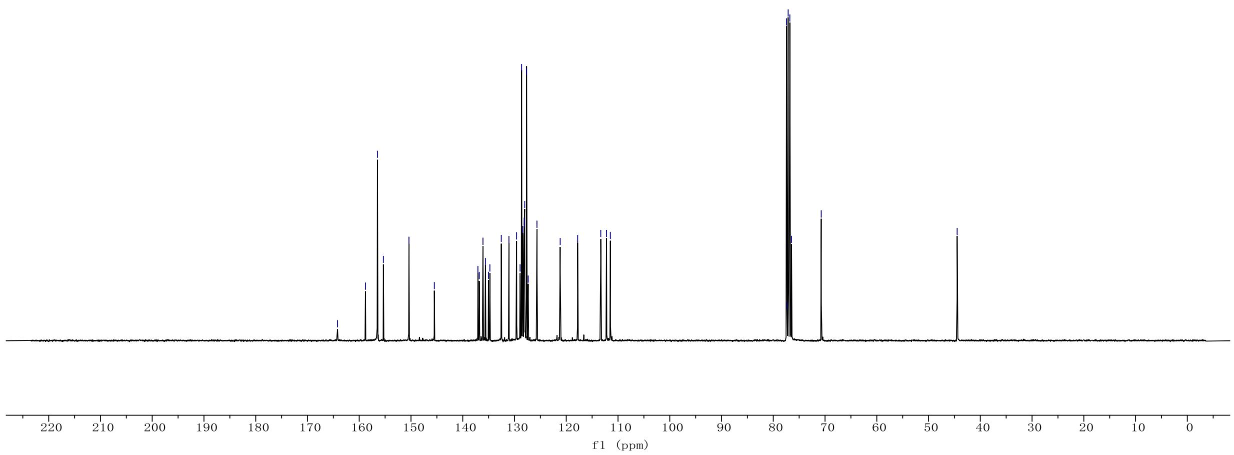


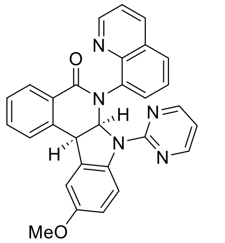

**29**

$^{1}H$ NMR, $CDCl_3$, 400 MHz

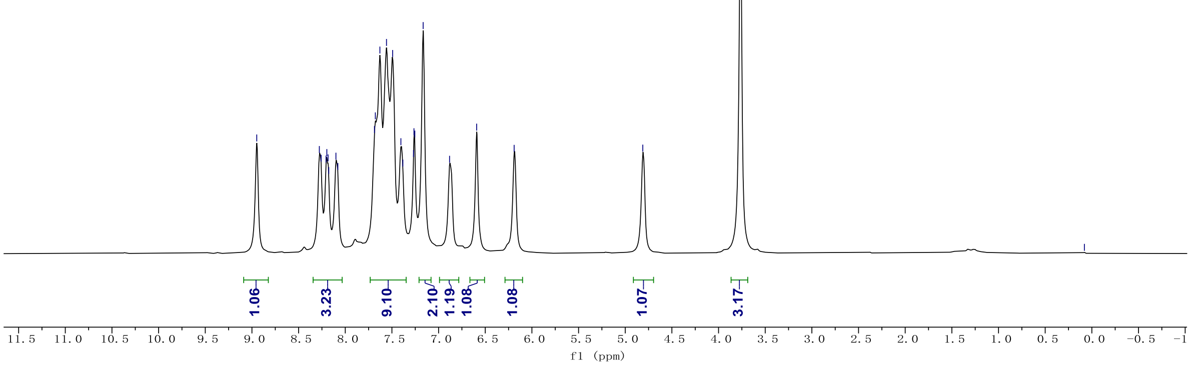

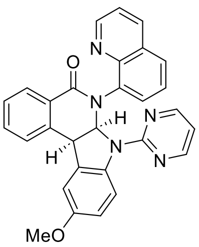

**29**

$^{13}$C NMR, $CDCl_3$, 101 MHz

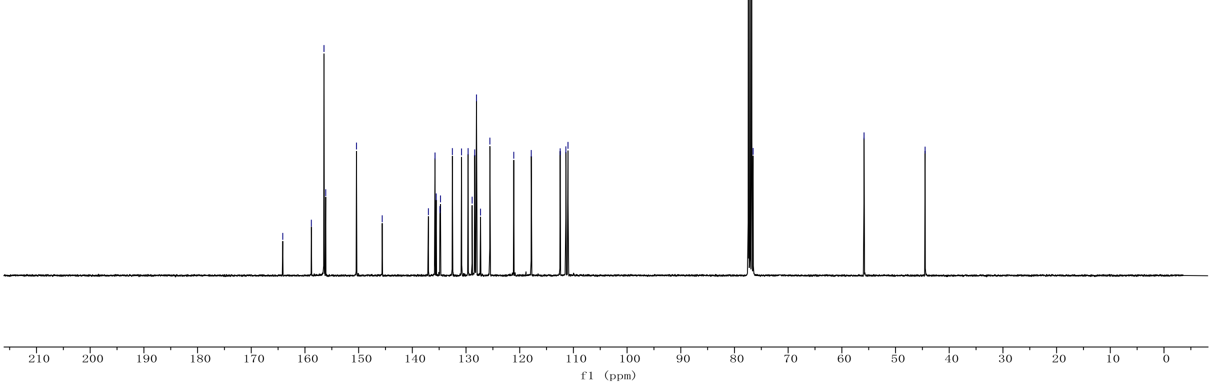


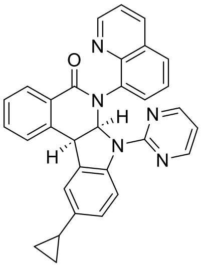

**30**

$^{1}$H NMR, $CDCl_3$, 400 MHz

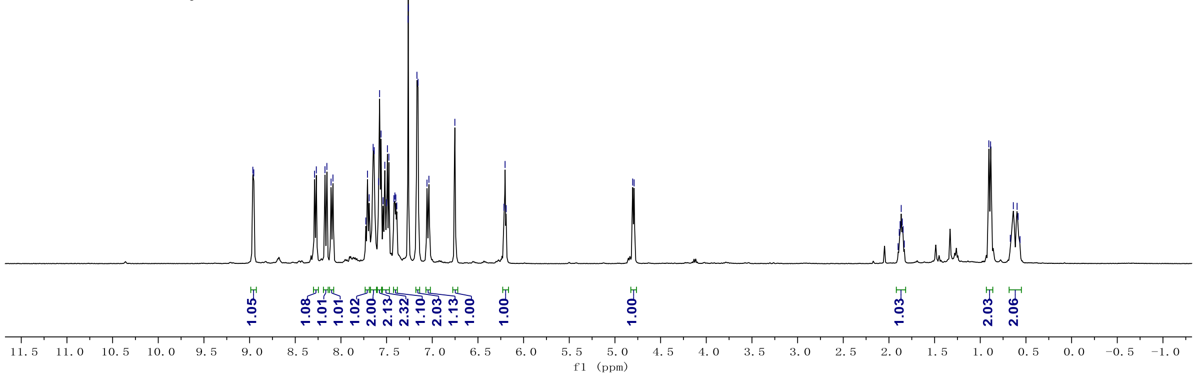

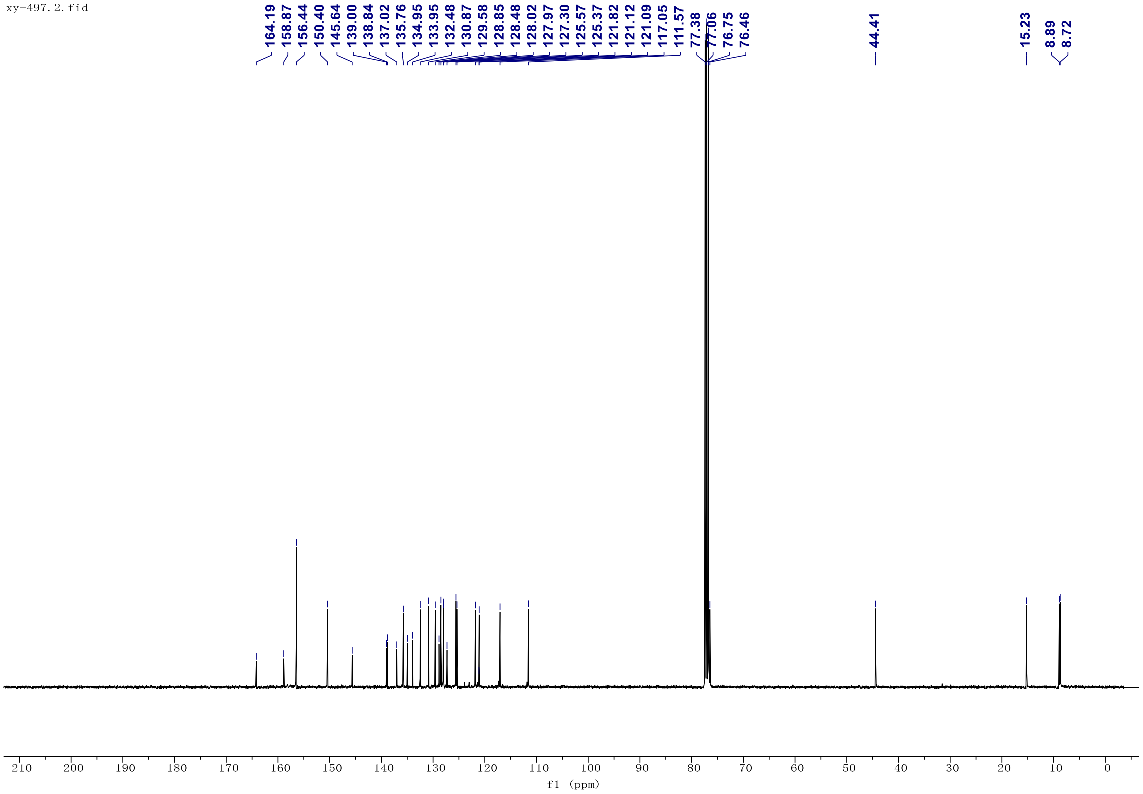


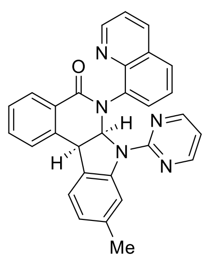


**31**

$^1$H NMR, $CDCl_3$, 400 MHz

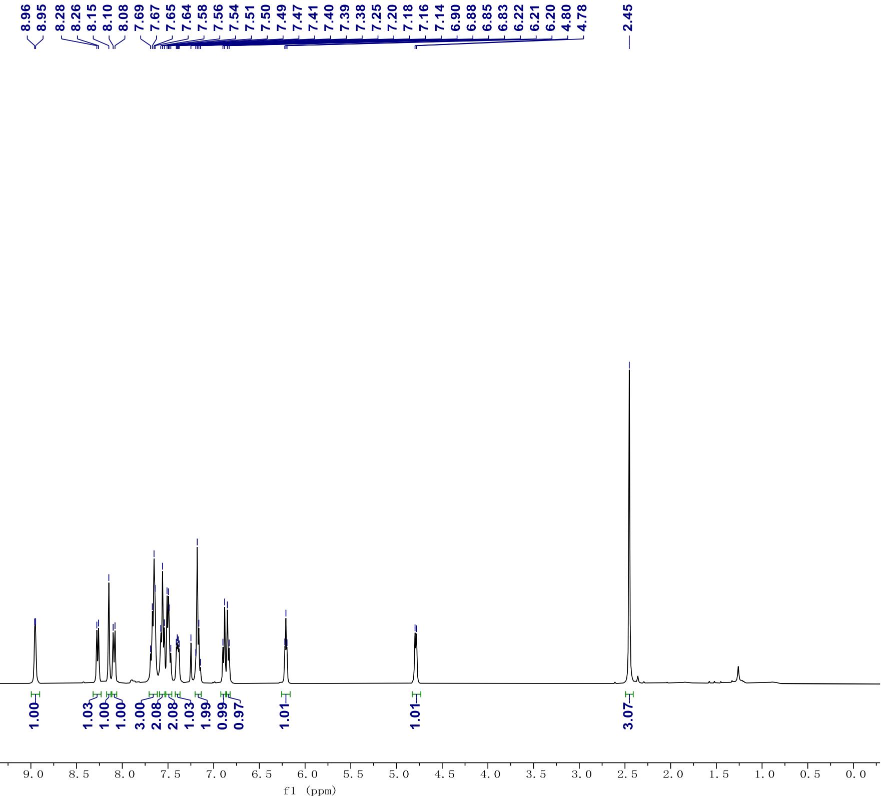

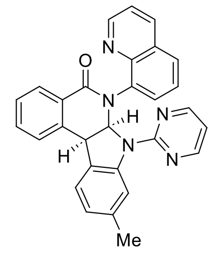

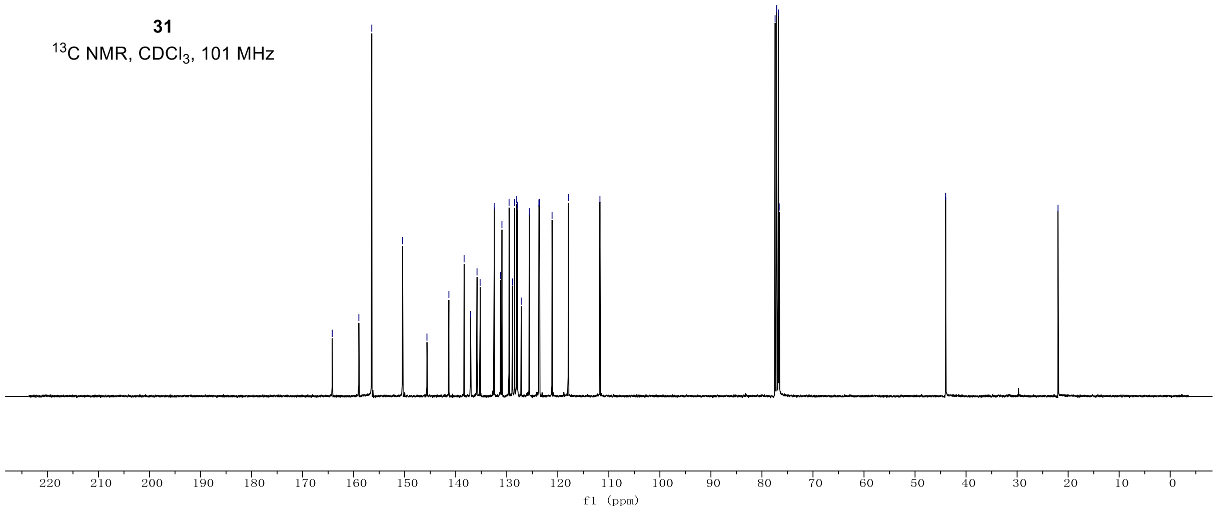


**31**

$^{13}$C NMR, $CDCl_3$, 101 MHz

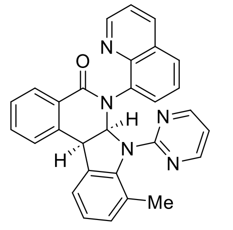

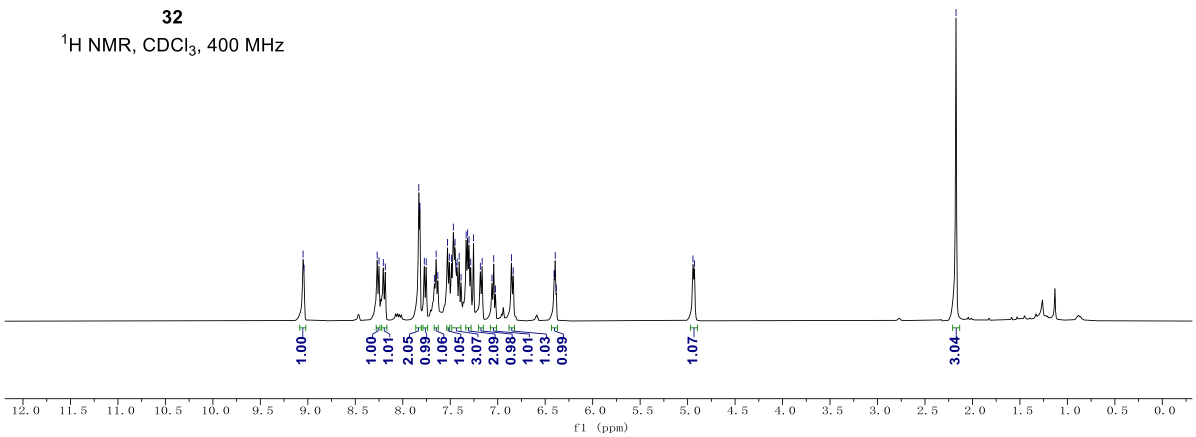


**32**

$^{1}$H NMR, $CDCl_3$, 400 MHz

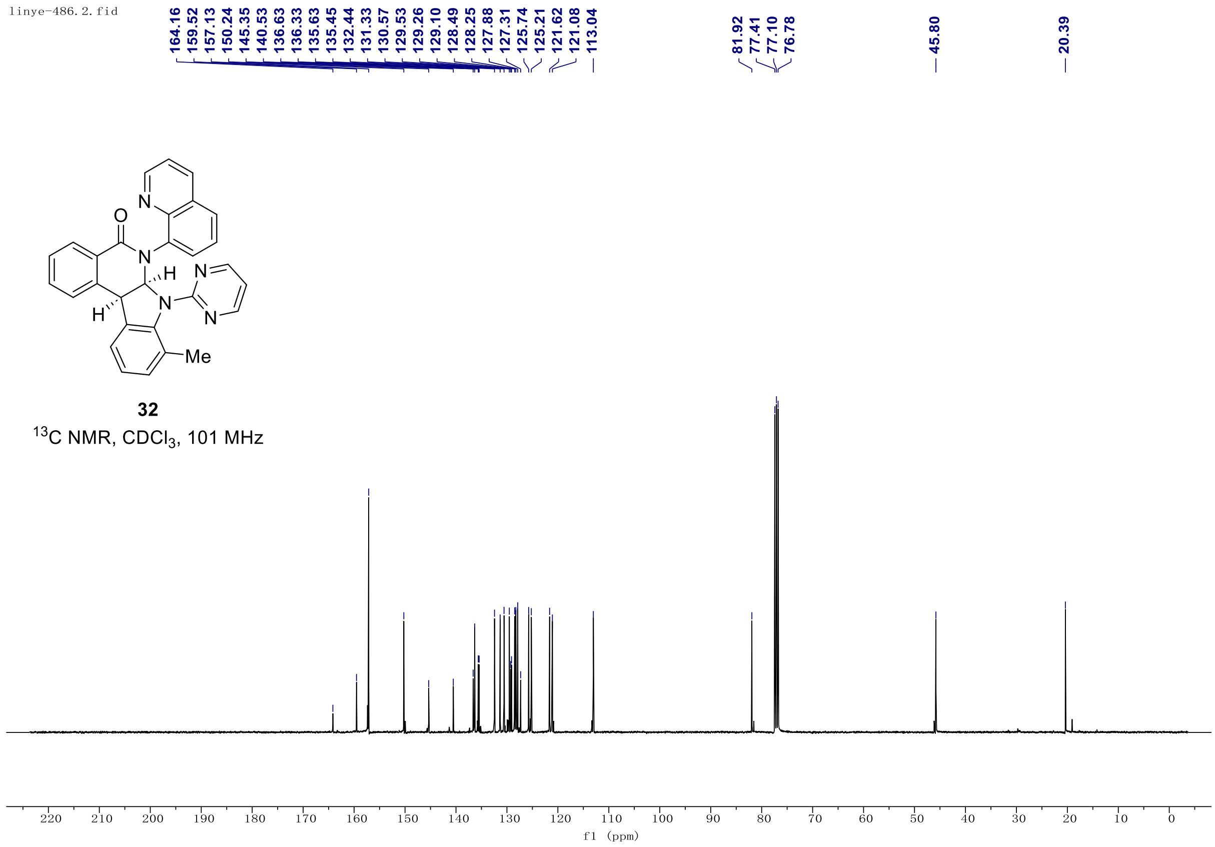


**32**

$^{13}C$ NMR, $CDCl_3$, 101 MHz

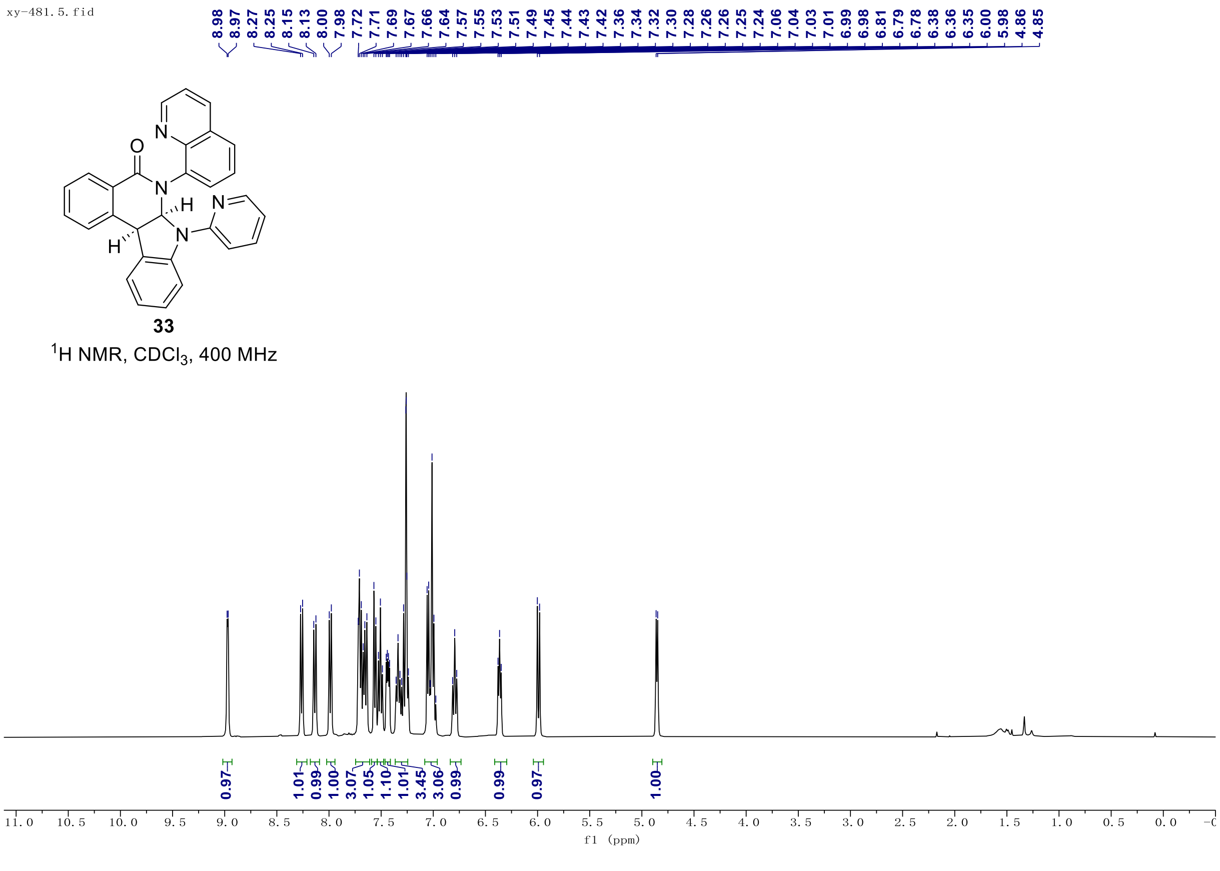


**33**

$^{1}H$ NMR, $CDCl_3$, 400 MHz

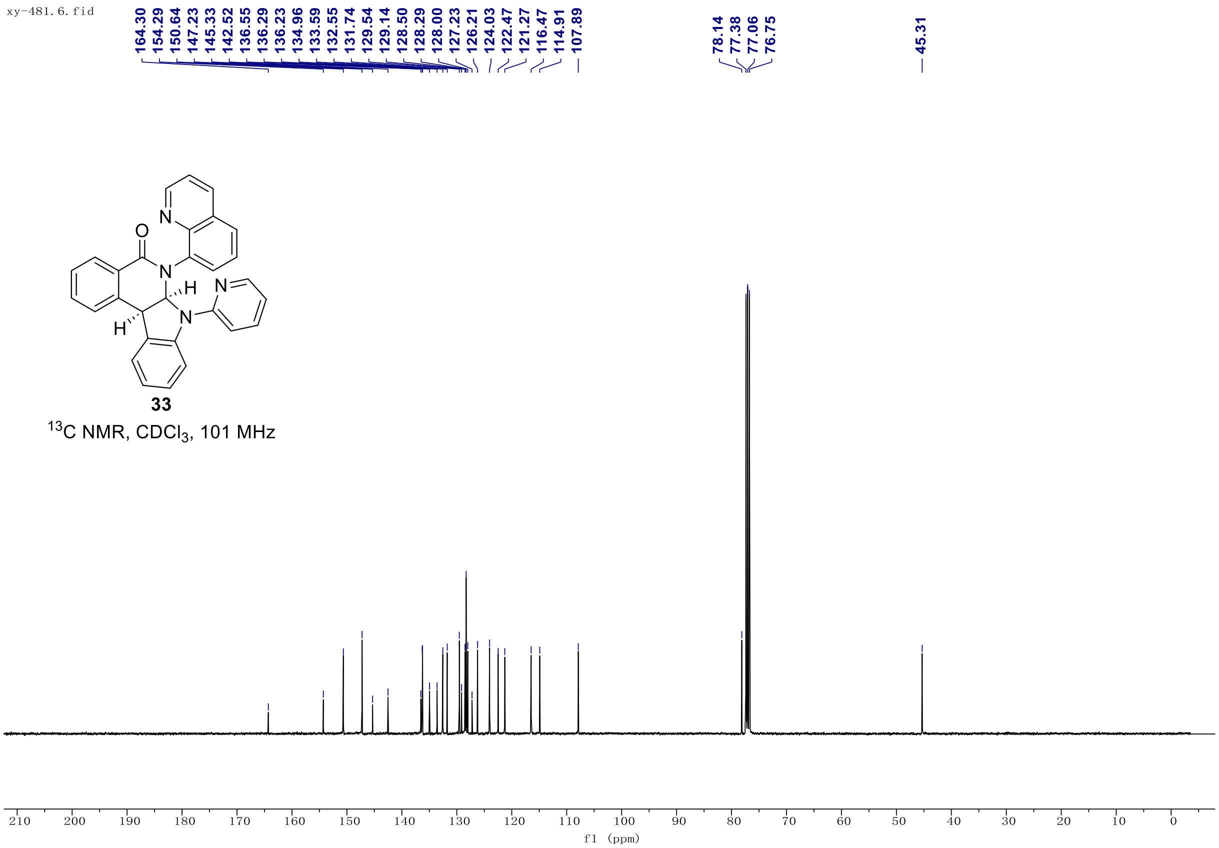


**33**

$^{13}$C NMR, $CDCl_3$, 101 MHz

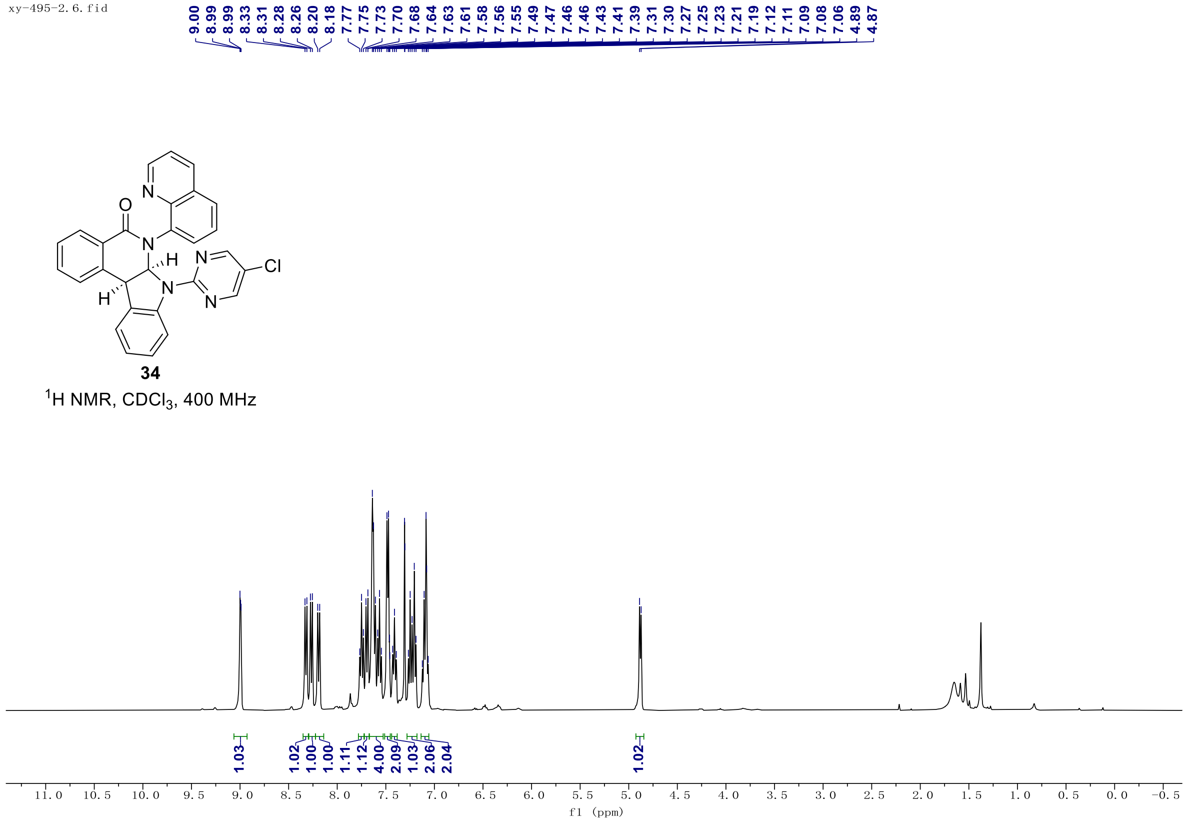


**34**

$^{1}$H NMR, $CDCl_3$, 400 MHz

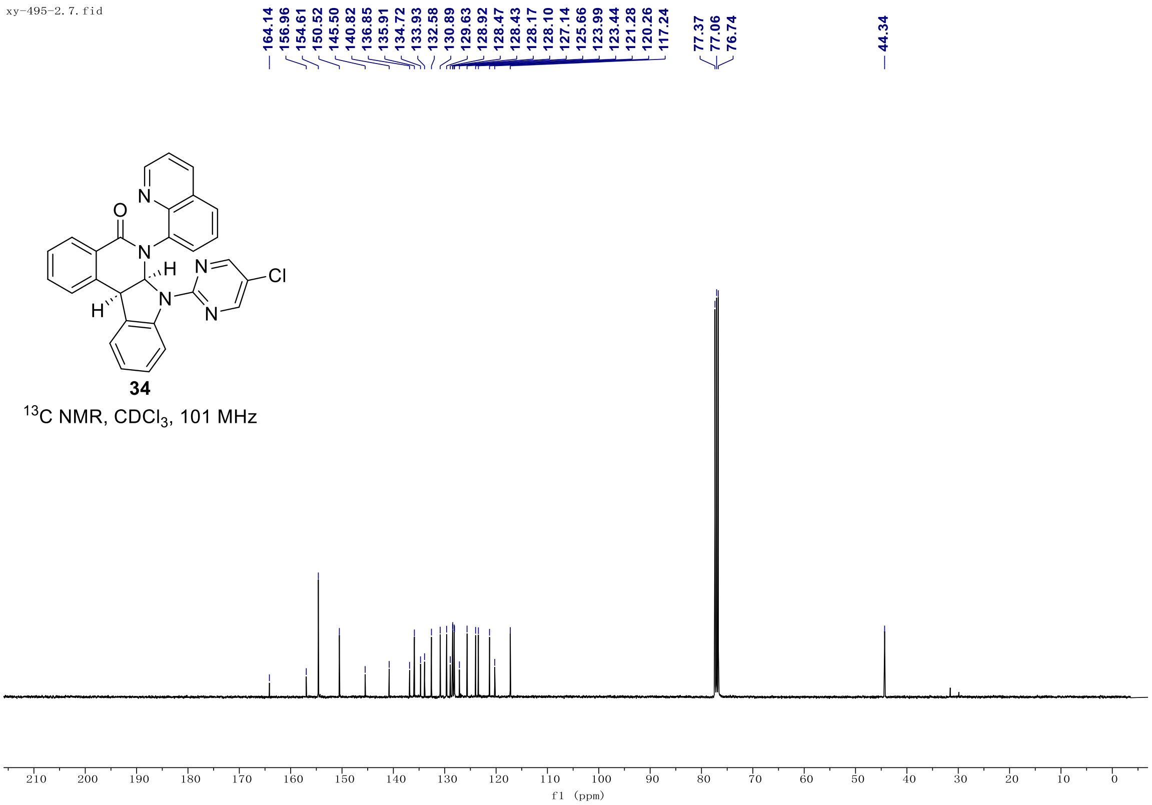


**34**

$^{13}C$ NMR, $CDCl_3$, 101 MHz

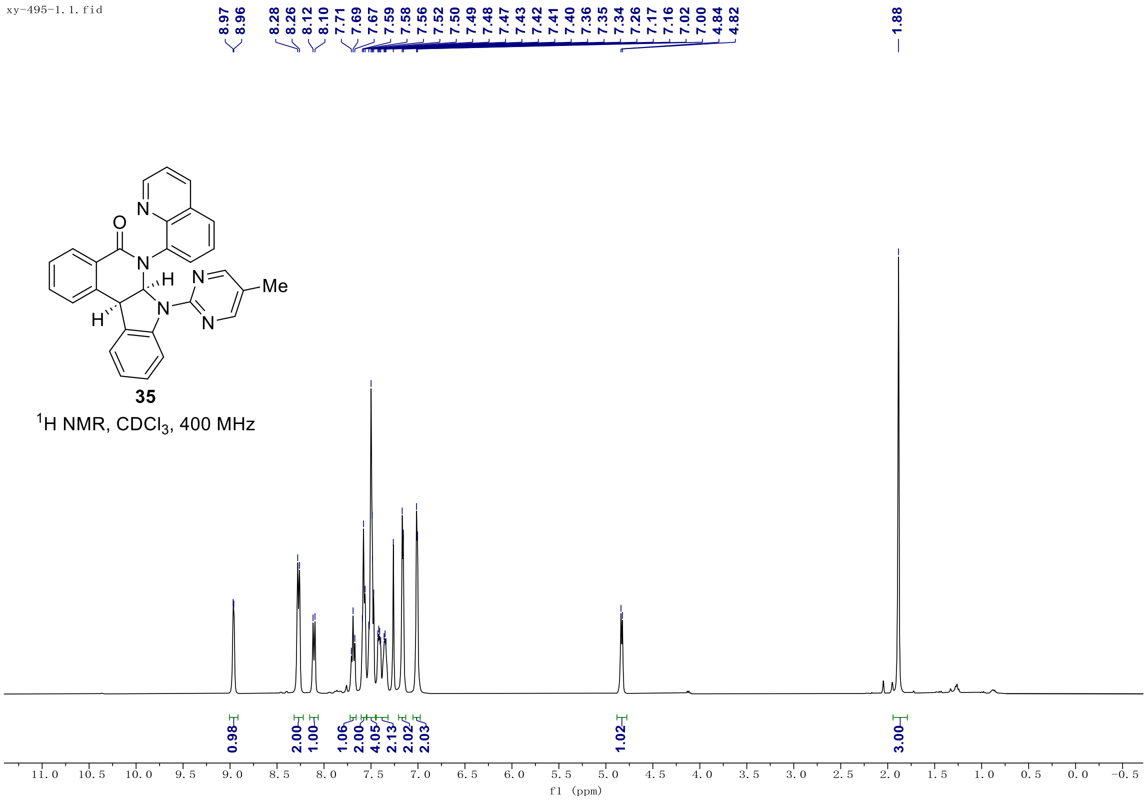


**35**

$^{1}H$ NMR, $CDCl_3$, 400 MHz

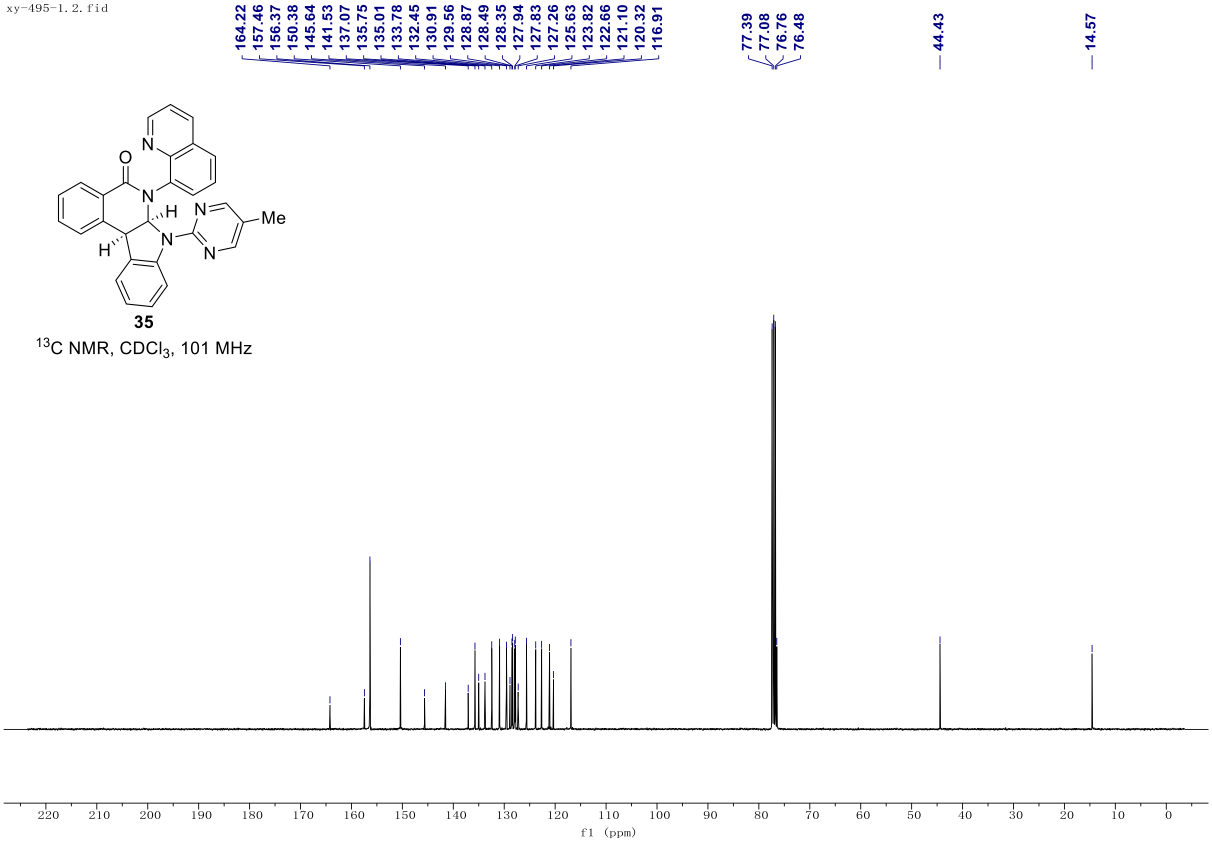


**35**

$^{13}C$ NMR, $CDCl_3$, 101 MHz

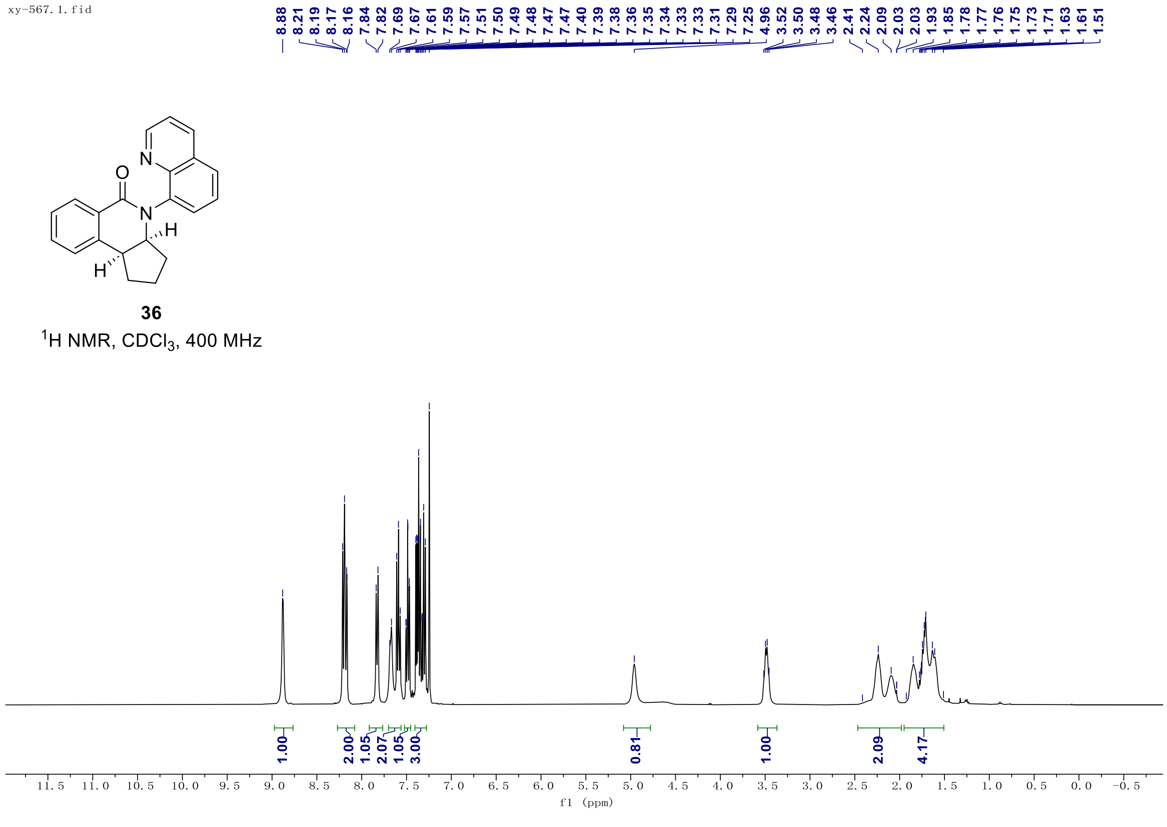


**36**

$^{1}H$ NMR, $CDCl_3$, 400 MHz

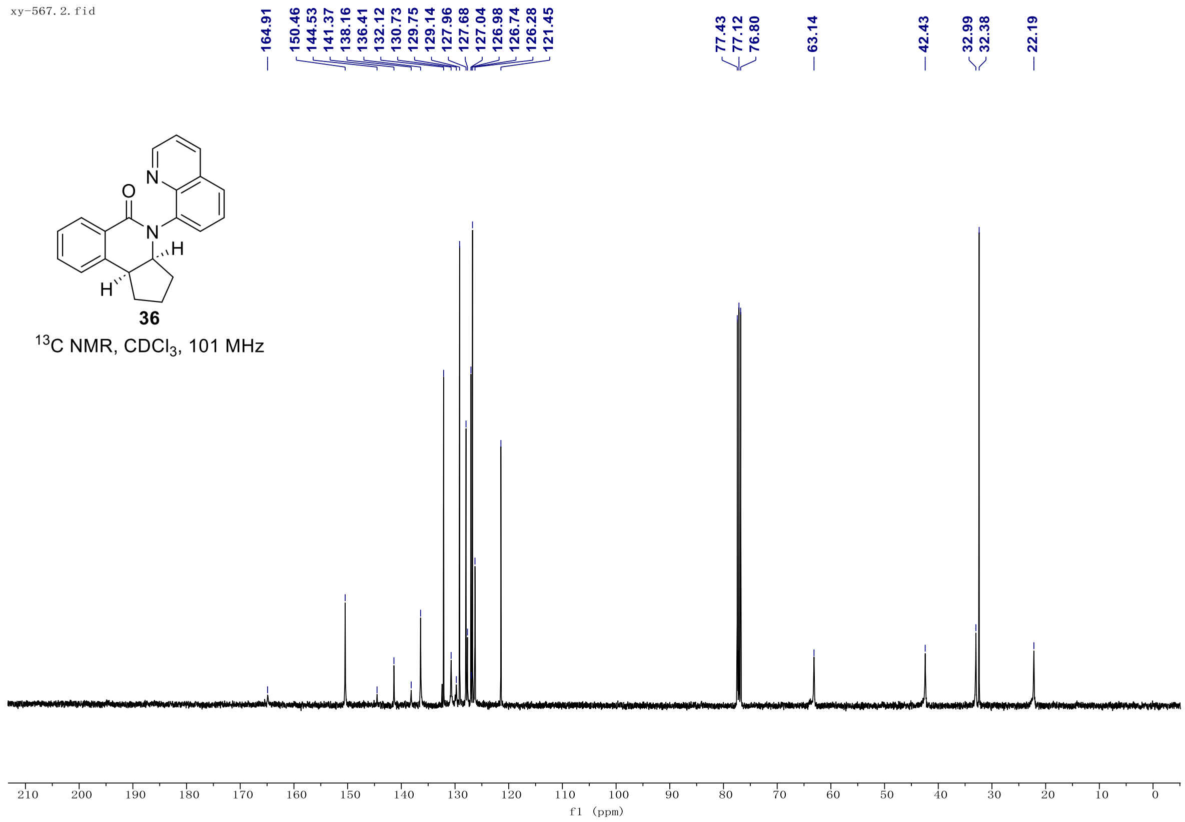
xy-567.2.fid
164.91
150.46
144.53
141.37
138.16
136.41
132.12
130.73
129.75
129.14
127.96
127.68
127.04
126.98
126.74
126.28
121.45
77.43
77.12
76.80
63.14
42.43
32.99
32.38
22.19
O
N
N
H
H
36
13C NMR, CDCl3, 101 MHz
210 200 190 180 170 160 150 140 130 120 110 100 90 80 70 60 50 40 30 20 10 0
f1 (ppm)

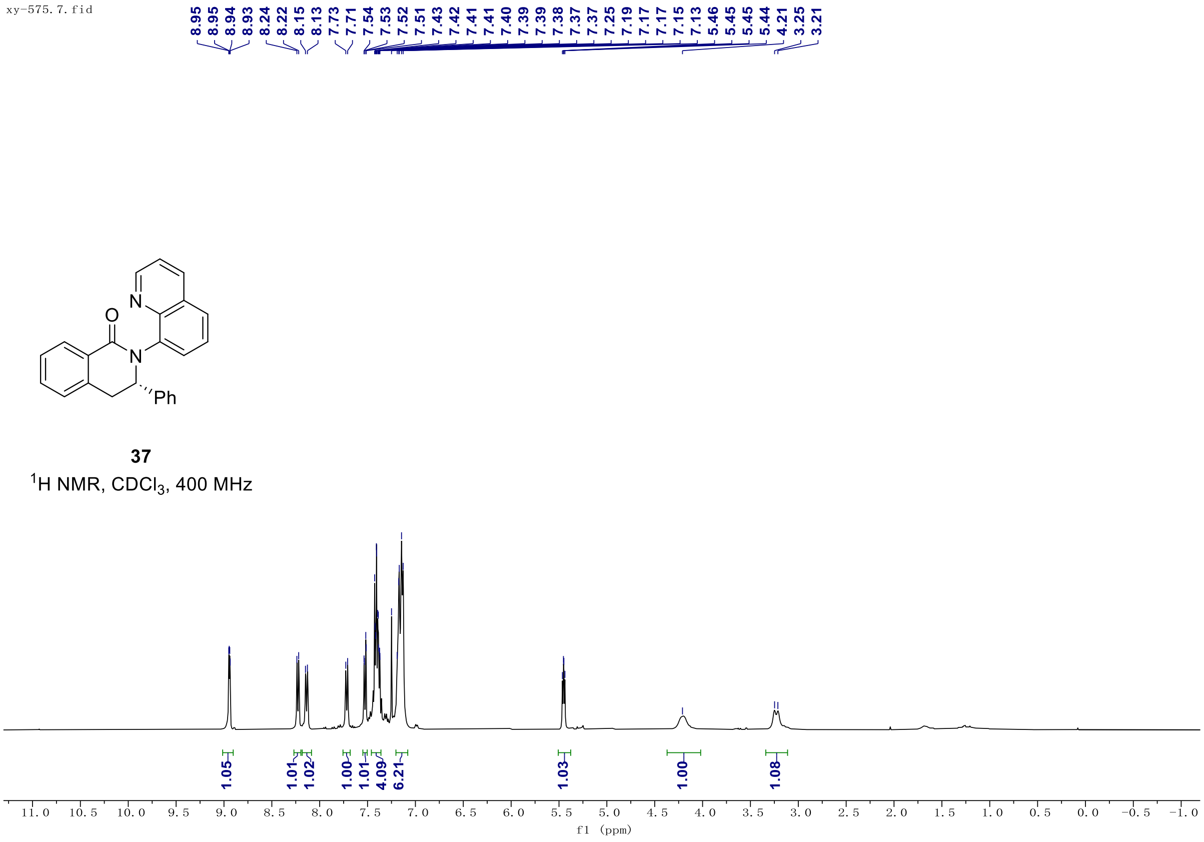
xy-575.7.fid
8.95
8.95
8.94
8.93
8.24
8.22
8.15
8.13
7.73
7.71
7.54
7.53
7.52
7.51
7.43
7.42
7.41
7.41
7.40
7.39
7.39
7.38
7.37
7.37
7.25
7.19
7.17
7.15
7.13
5.46
5.45
5.45
5.44
4.21
3.25
3.21
O
N
N
Ph
37
1H NMR, CDCl3, 400 MHz
1.05
1.01
1.02
1.00
1.01
4.09
6.21
1.03
1.00
1.08
11.0 10.5 10.0 9.5 9.0 8.5 8.0 7.5 7.0 6.5 6.0 5.5 5.0 4.5 4.0 3.5 3.0 2.5 2.0 1.5 1.0 0.5 0.0 -0.5 -1.0
f1 (ppm)

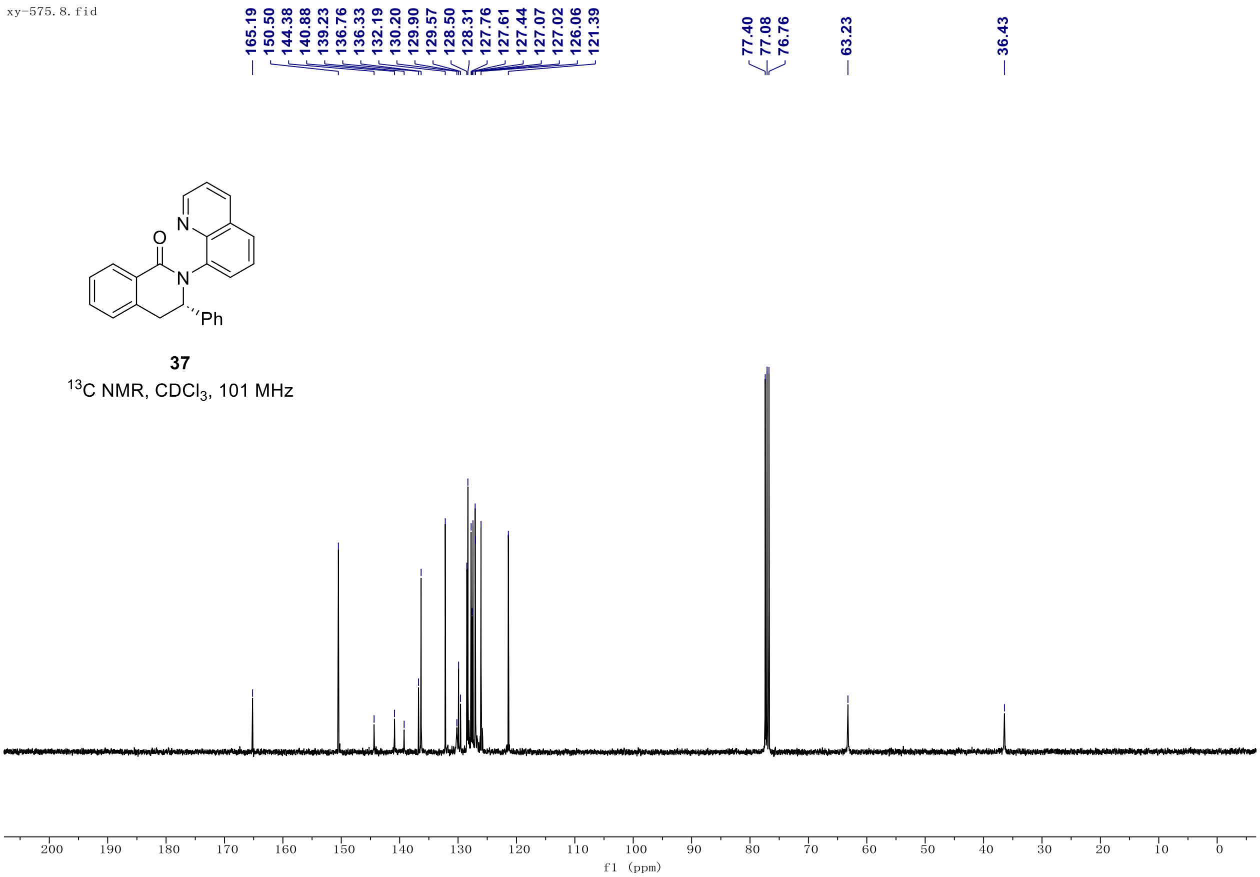
xy-575.8.fid
165.19 150.50 144.38 140.88 139.23 136.76 136.33 132.19 130.20 129.90 129.57 128.50 128.31 127.76 127.61 127.44 127.07 127.02 126.06 121.39
77.40 77.08 76.76
63.23
36.43
O
N
N
Ph
37
$^{13}$C NMR, CDCl$_3$, 101 MHz
200 190 180 170 160 150 140 130 120 110 100 90 80 70 60 50 40 30 20 10 0
f1 (ppm)

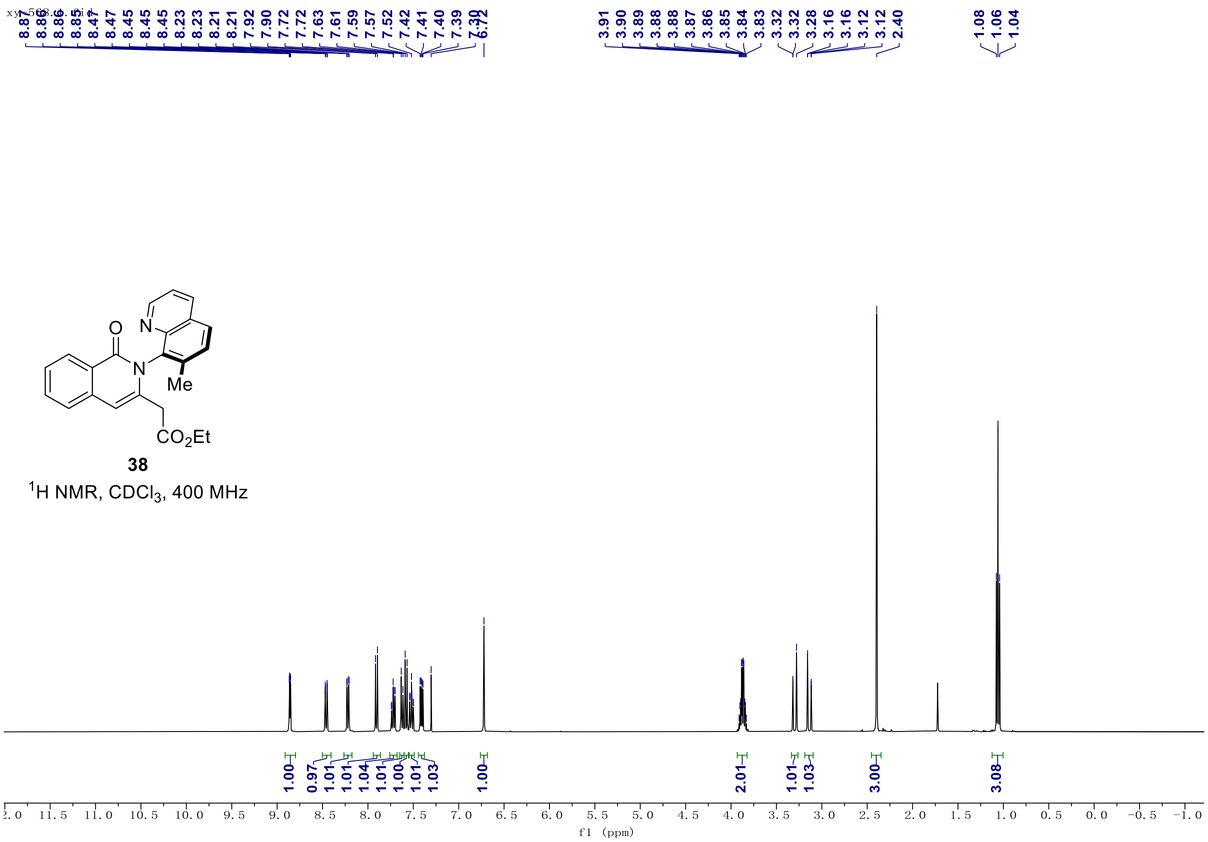
3.91 3.90 3.89 3.88 3.88 3.87 3.86 3.85 3.84 3.83 3.32 3.32 3.28 3.16 3.16 3.12 3.12 2.40
1.08 1.06 1.04
O
N
N
Me
CO$_2$Et
38
$^1$H NMR, CDCl$_3$, 400 MHz
1.00 0.97 1.01 1.01 1.04 1.01 1.00 1.01 1.03 1.00
2.01 1.01 1.03 3.00 3.08
11.5 11.0 10.5 10.0 9.5 9.0 8.5 8.0 7.5 7.0 6.5 6.0 5.5 5.0 4.5 4.0 3.5 3.0 2.5 2.0 1.5 1.0 0.5 0.0 -0.5 -1.0
f1 (ppm)

xy-568.2.fid

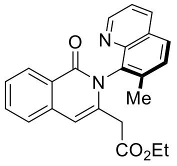

**38**

$^{13}C$ NMR, $CDCl_3$, 101 MHz

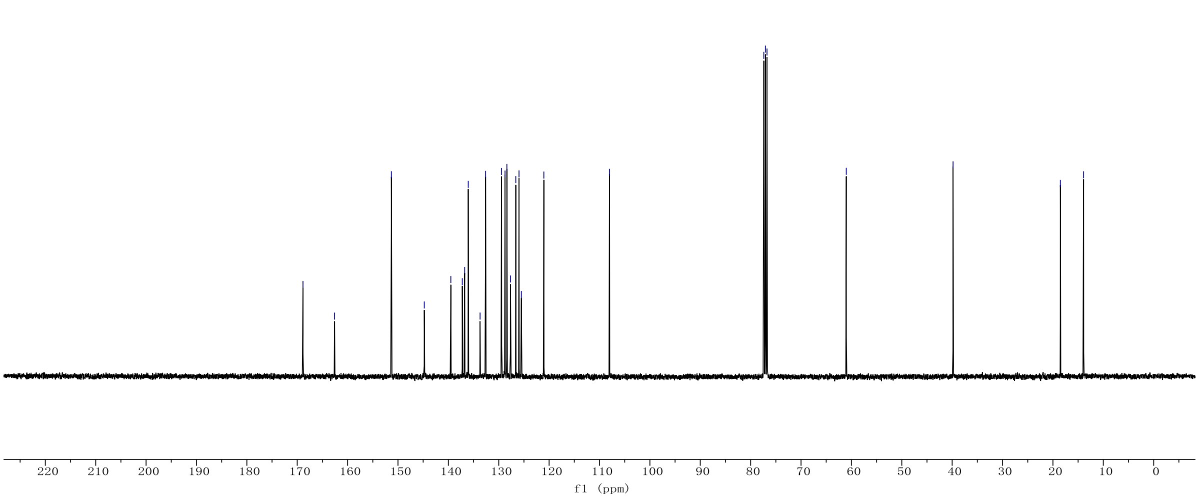


xy-573.1.fid

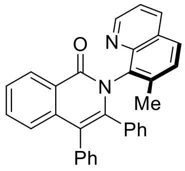

**39**

$^{1}H$ NMR, $CDCl_3$, 400 MHz

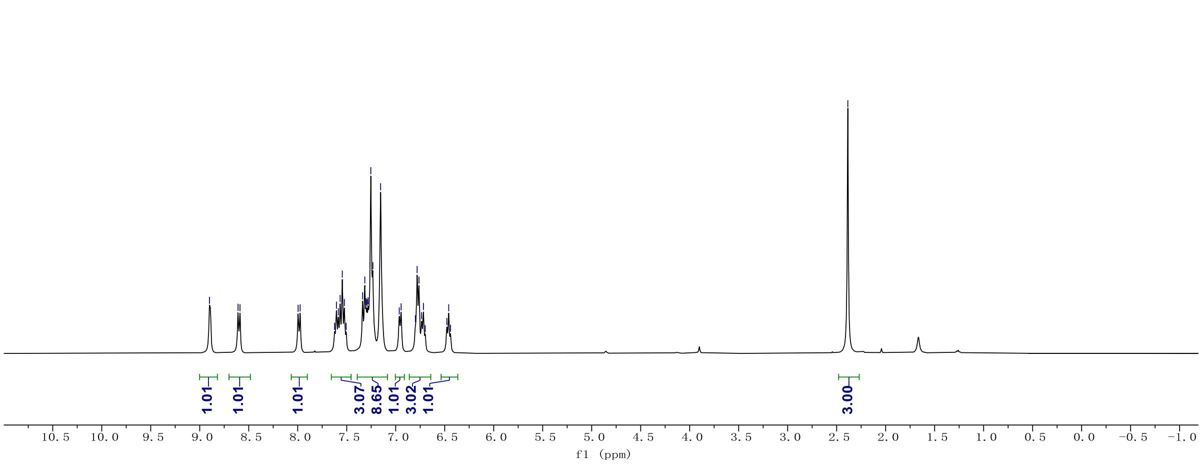

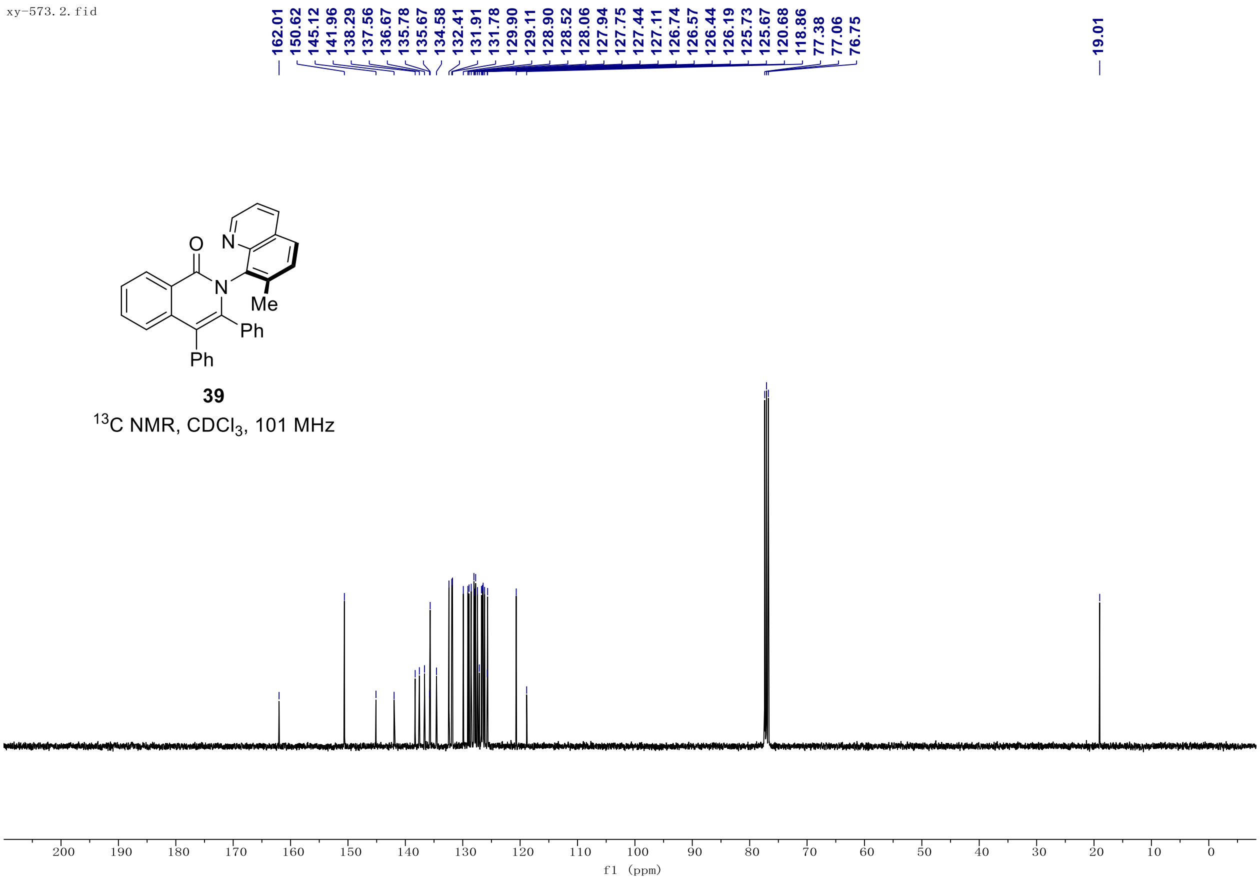
xy-573.2.fid
162.01 150.62 145.12 141.96 138.29 137.56 136.67 135.78 135.67 134.58 132.41 131.91 131.78 129.90 129.11 128.90 128.52 128.06 127.94 127.75 127.44 127.11 126.74 126.57 126.44 126.19 125.73 125.67 120.68 118.86 77.38 77.06 76.75
19.01
O
N
N
Me
Ph
Ph
39
13C NMR, CDCl3, 101 MHz
200 190 180 170 160 150 140 130 120 110 100 90 80 70 60 50 40 30 20 10 0
f1 (ppm)

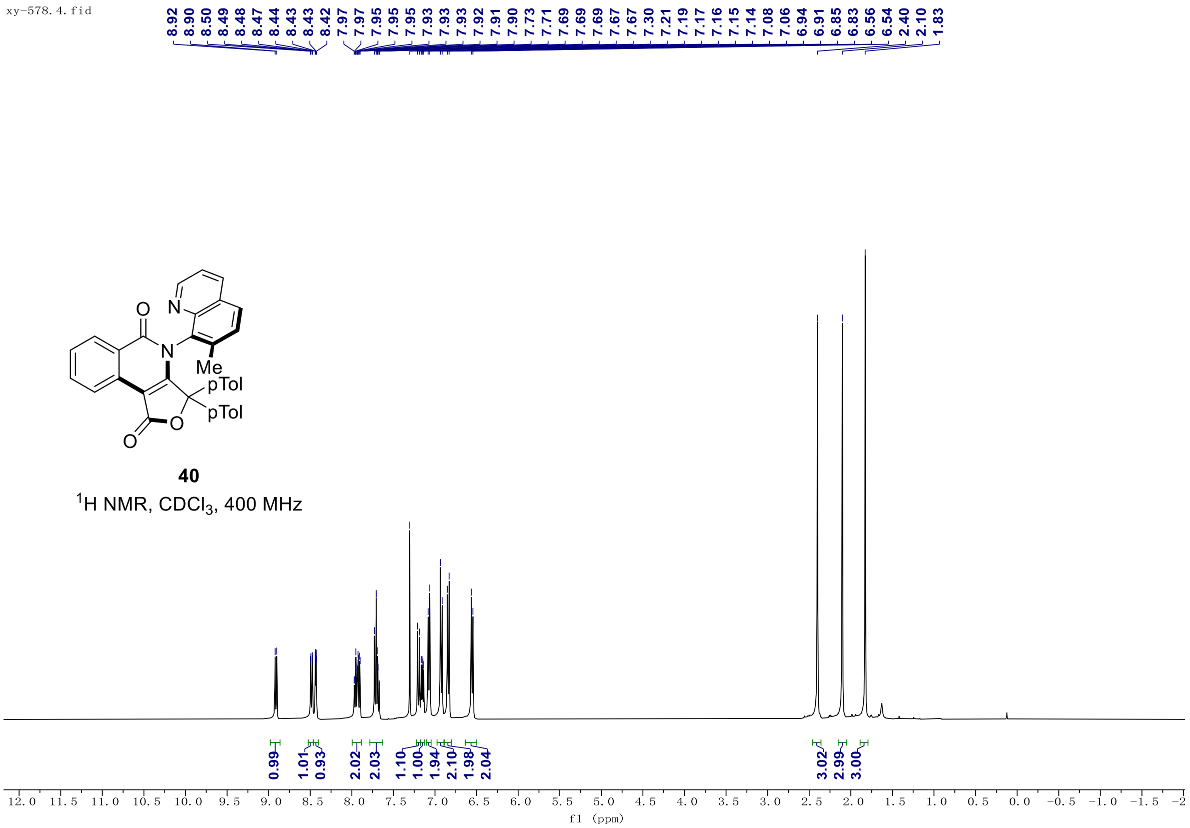
xy-578.4.fid
8.92 8.90 8.50 8.49 8.48 8.47 8.44 8.43 8.43 8.42 7.97 7.97 7.95 7.95 7.93 7.93 7.93 7.92 7.91 7.90 7.73 7.71 7.69 7.69 7.67 7.30 7.21 7.19 7.17 7.16 7.15 7.14 7.08 7.06 6.94 6.91 6.85 6.83 6.56 6.54 2.40 2.10 1.83
O
N
N
Me
pTol
pTol
O
O
40
1H NMR, CDCl3, 400 MHz
0.99 1.01 0.93 2.02 2.03 1.10 1.00 1.94 2.10 1.98 2.04 3.02 2.99 3.00
12.0 11.5 11.0 10.5 10.0 9.5 9.0 8.5 8.0 7.5 7.0 6.5 6.0 5.5 5.0 4.5 4.0 3.5 3.0 2.5 2.0 1.5 1.0 0.5 0.0 -0.5 -1.0 -1.5 -2
f1 (ppm)

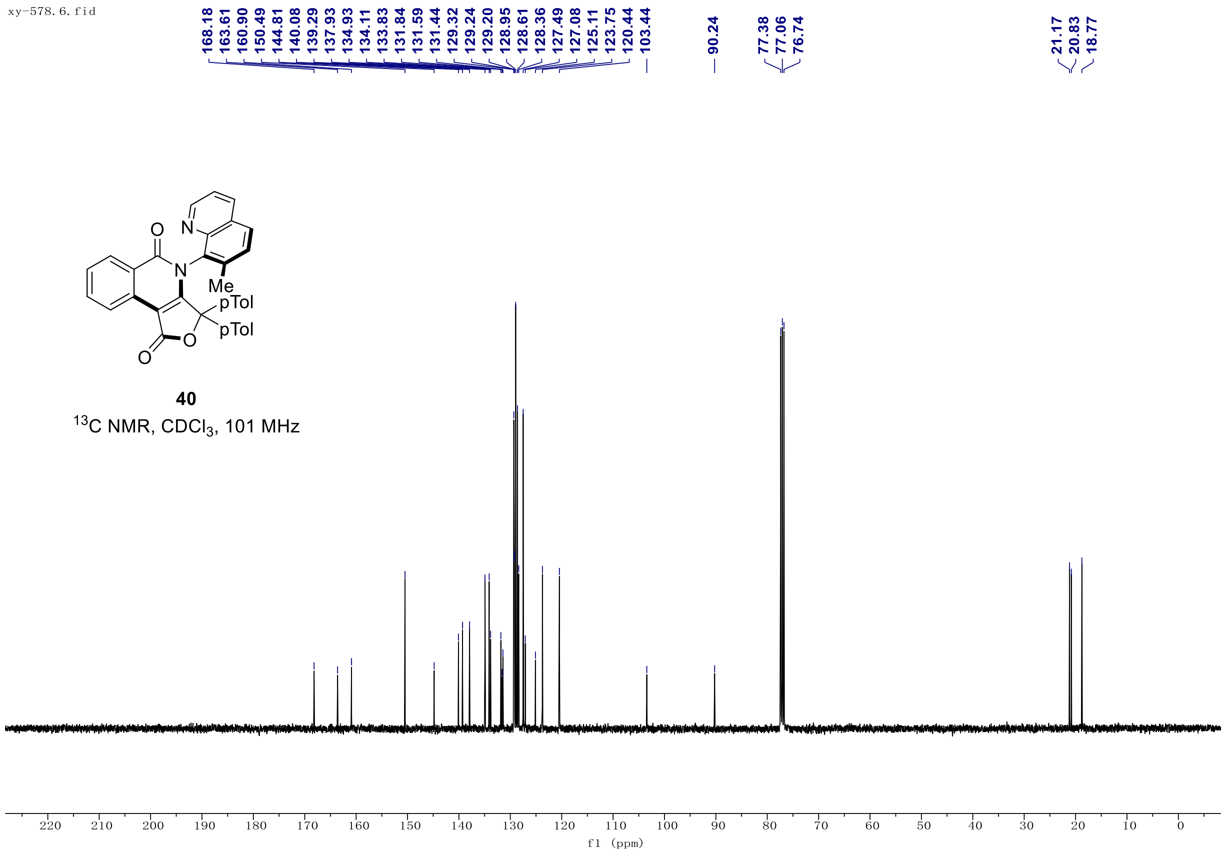
xy-578.6.fid
168.18
163.61
160.90
150.49
144.81
140.08
139.29
137.93
134.93
134.11
133.83
131.84
131.59
131.44
129.32
129.24
129.20
128.95
128.61
128.36
127.49
127.08
125.11
123.75
120.44
103.44
90.24
77.38
77.06
76.74
21.17
20.83
18.77
N
O
N
Me
pTol
pTol
O
O
40
$^{13}$C NMR, $CDCl_3$, 101 MHz
f1 (ppm)

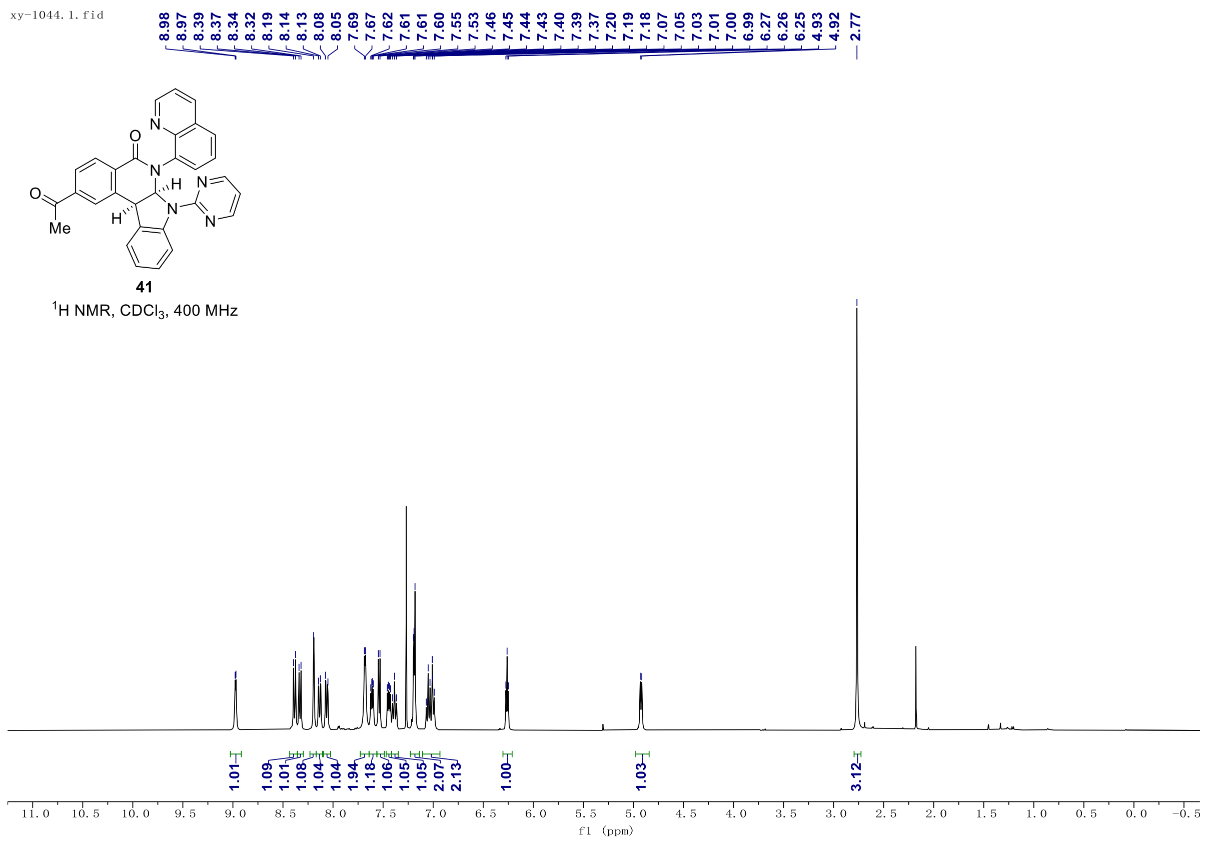
xy-1044.1.fid
8.98
8.97
8.39
8.37
8.34
8.32
8.19
8.14
8.13
8.08
8.05
7.69
7.67
7.62
7.61
7.61
7.60
7.55
7.53
7.46
7.45
7.44
7.43
7.40
7.39
7.37
7.20
7.19
7.18
7.07
7.05
7.03
7.01
7.00
6.99
6.27
6.26
6.25
4.93
4.92
2.77
N
O
N
H
N
N
N
O
H
Me
41
$^{1}$H NMR, $CDCl_3$, 400 MHz
1.01
1.09
1.01
1.08
1.04
1.04
1.94
1.18
1.06
1.05
1.05
2.07
2.13
1.00
1.03
3.12
f1 (ppm)

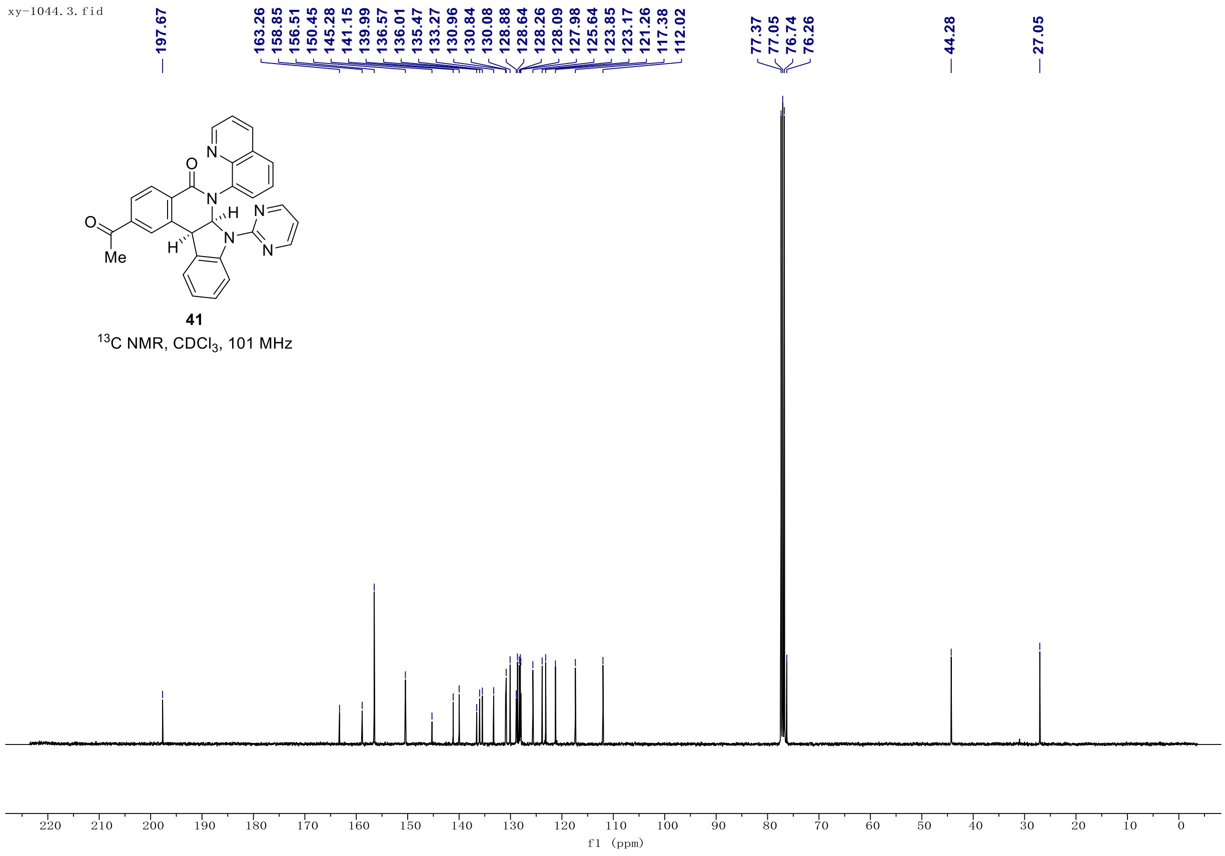
xy-1044.3.fid
197.67
163.26
158.85
156.51
150.45
145.28
141.15
139.99
136.57
136.01
135.47
133.27
130.96
130.84
130.08
128.88
128.64
128.26
128.09
127.98
125.64
123.85
123.17
121.26
117.38
112.02
77.37
77.05
76.74
76.26
44.28
27.05
O
N
N
H
H
N
N
N
O
Me
41
$^{13}C$ NMR, $CDCl_3$, 101 MHz
220 210 200 190 180 170 160 150 140 130 120 110 100 90 80 70 60 50 40 30 20 10 0
f1 (ppm)

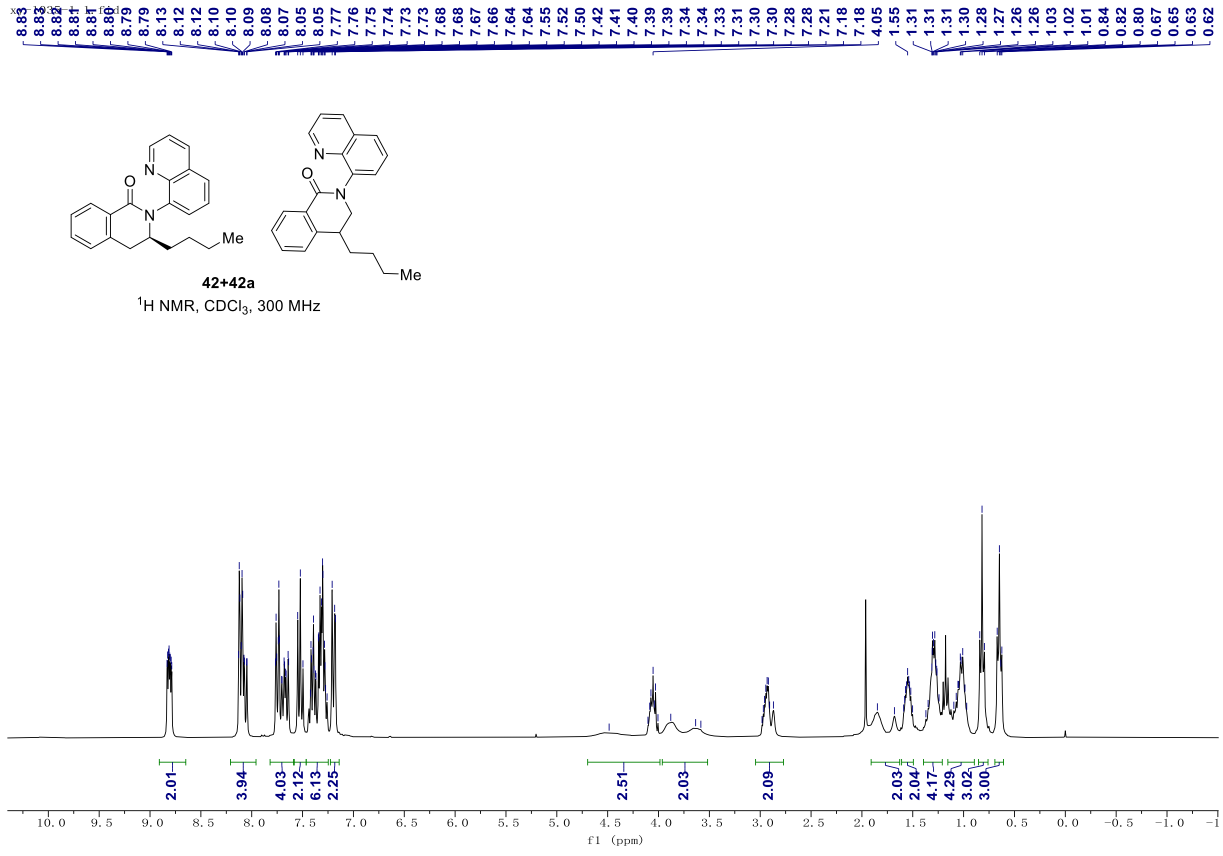
8.83 8.82 8.82 8.81 8.81 8.80 8.79 8.13 8.12 8.12 8.10 8.09 8.09 8.08 8.07 8.05 8.05 7.77 7.76 7.75 7.74 7.73 7.73 7.68 7.67 7.66 7.64 7.64 7.55 7.52 7.50 7.42 7.41 7.40 7.39 7.39 7.34 7.34 7.33 7.31 7.30 7.30 7.28 7.28 7.21 7.18 4.05 1.55 1.31 1.31 1.31 1.30 1.28 1.27 1.26 1.03 1.02 1.01 0.84 0.82 0.80 0.67 0.65 0.63 0.62
N
O
N
Me
N
O
N
Me
42+42a
$^{1}H$ NMR, $CDCl_3$, 300 MHz
2.01
3.94
4.03
2.12
6.13
2.25
2.51
2.03
2.09
2.03
2.04
4.17
4.29
3.02
3.00
10.0 9.5 9.0 8.5 8.0 7.5 7.0 6.5 6.0 5.5 5.0 4.5 4.0 3.5 3.0 2.5 2.0 1.5 1.0 0.5 0.0 −0.5 −1.0 −1
f1 (ppm)

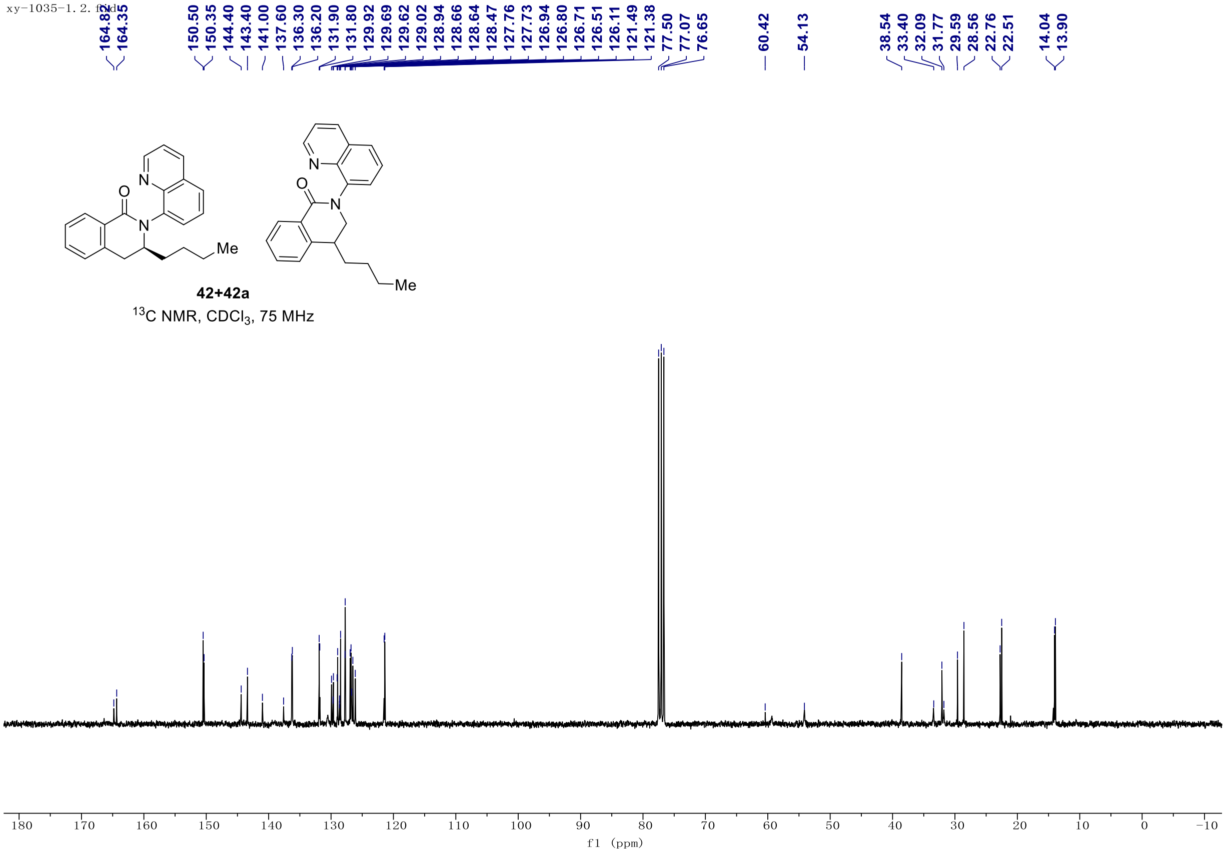
xy-1035-1.2.fid
164.82
164.35
150.50
150.35
144.40
143.40
141.00
137.60
136.30
136.20
131.90
131.80
129.92
129.69
129.62
129.02
128.94
128.66
128.64
128.47
127.76
127.73
126.94
126.80
126.71
126.51
126.11
121.49
121.38
77.50
77.07
76.65
60.42
54.13
38.54
33.40
32.09
31.77
29.59
28.56
22.76
22.51
14.04
13.90
42+42a
$^{13}$C NMR, $CDCl_3$, 75 MHz
f1 (ppm)

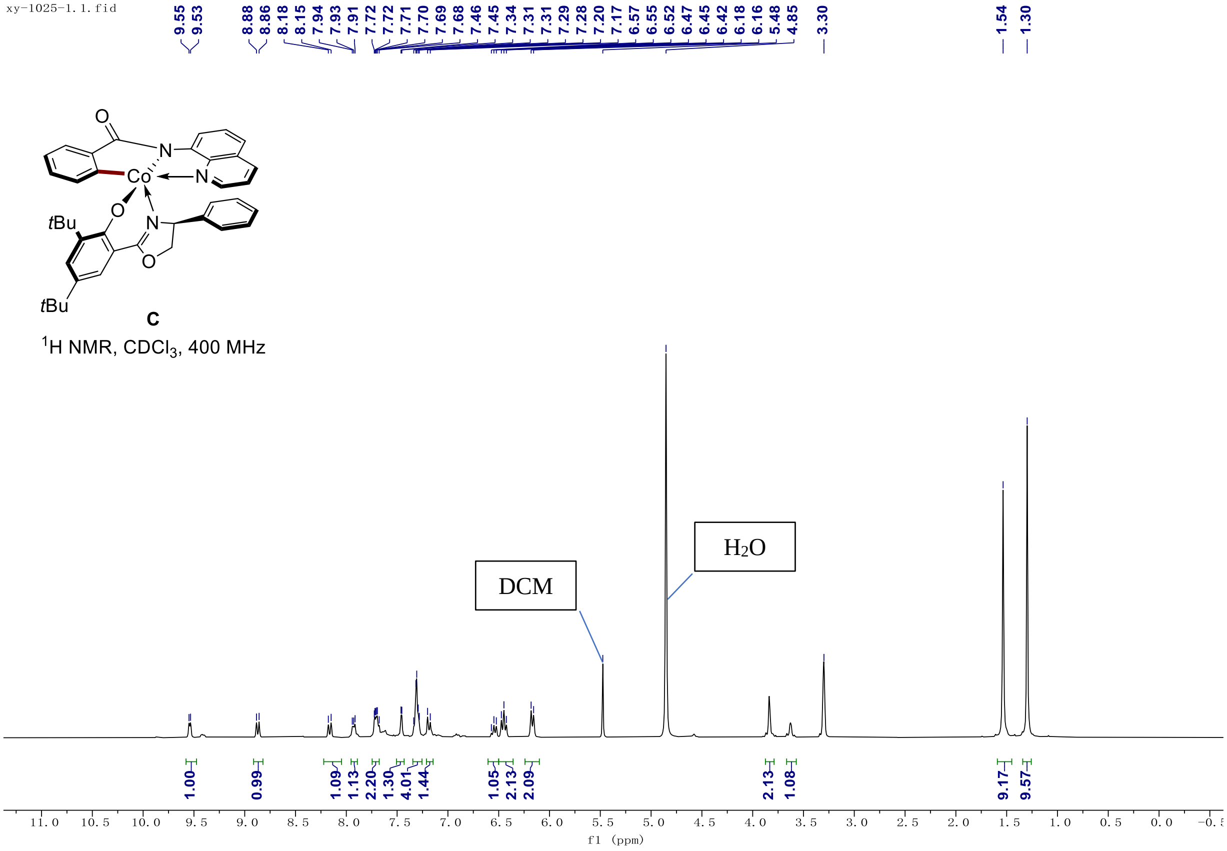
xy-1025-1.1.fid
9.55
9.53
8.88
8.86
8.18
8.15
7.94
7.93
7.91
7.72
7.72
7.71
7.70
7.69
7.68
7.46
7.45
7.34
7.31
7.29
7.28
7.20
7.17
6.57
6.55
6.52
6.47
6.45
6.42
6.18
6.16
5.48
4.85
3.30
1.54
1.30
C
$^1$H NMR, $CDCl_3$, 400 MHz
DCM
$H_2O$
1.00
0.99
1.09
1.13
2.20
1.30
4.01
1.44
1.05
2.13
2.09
2.13
1.08
9.17
9.57
f1 (ppm)

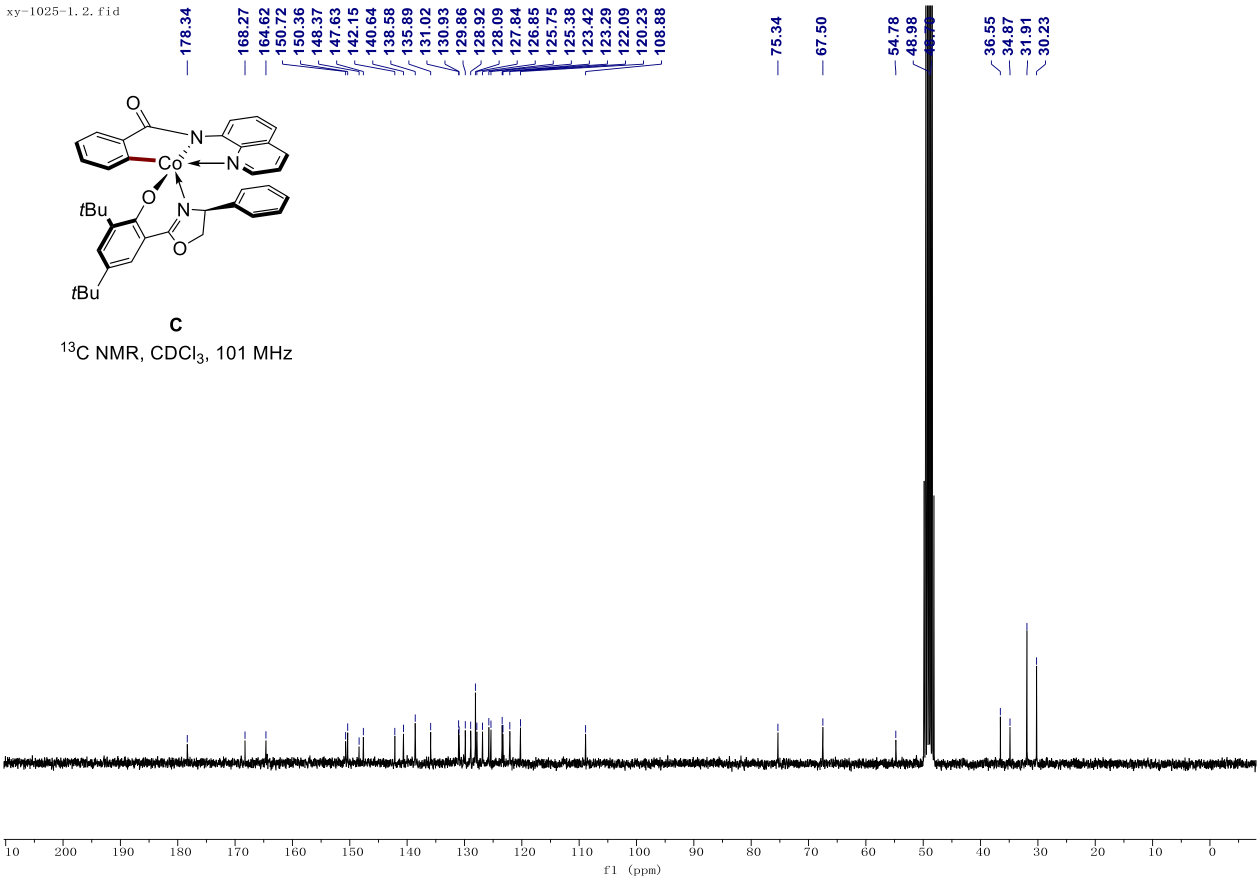
xy-1025-1.2.fid
178.34
168.27
164.62
150.72
150.36
148.37
147.63
142.15
140.64
138.58
135.89
131.02
130.93
129.86
128.92
128.09
127.84
126.85
125.75
125.38
123.42
123.29
122.09
120.23
108.88
75.34
67.50
54.78
48.98
36.55
34.87
31.91
30.23
C
$^{13}C$ NMR, $CDCl_3$, 101 MHz
f1 (ppm)

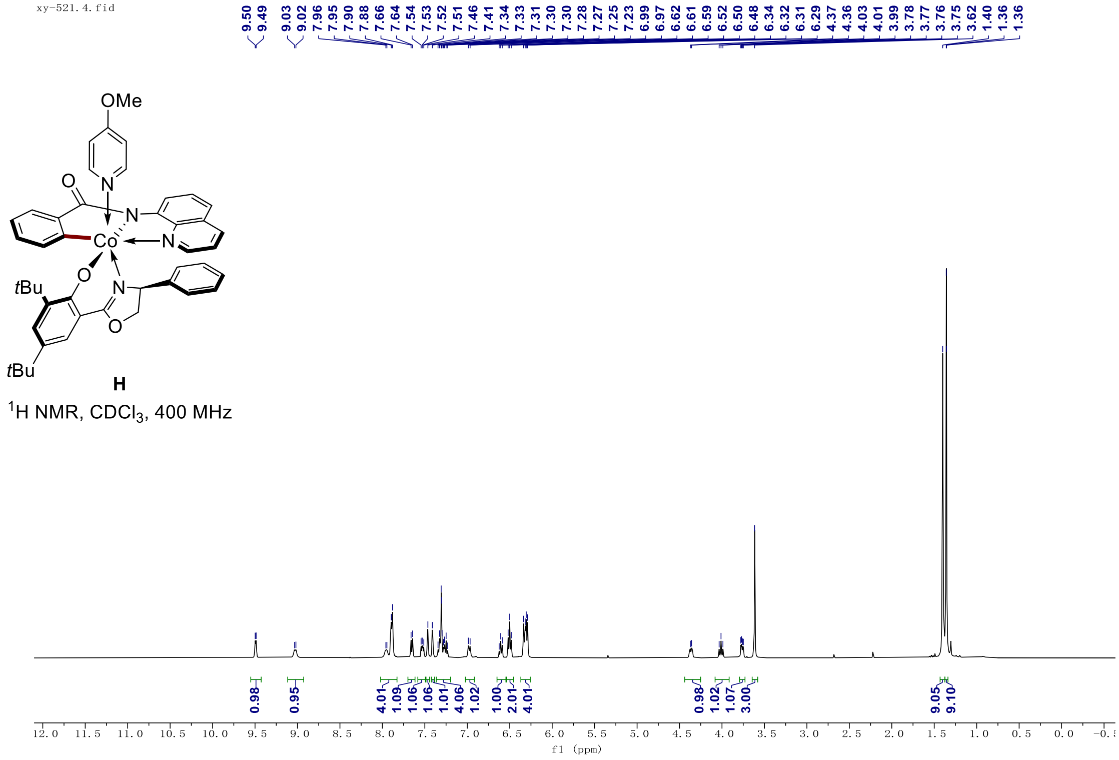
xy-521.4.fid
9.50
9.49
9.03
9.02
7.96
7.95
7.90
7.88
7.66
7.64
7.54
7.53
7.52
7.51
7.46
7.41
7.34
7.33
7.31
7.30
7.28
7.27
7.25
7.23
6.99
6.97
6.62
6.61
6.59
6.52
6.50
6.48
6.34
6.32
6.31
6.29
4.37
4.36
4.03
4.01
3.99
3.78
3.77
3.76
3.75
3.62
1.40
1.36
1.36
OMe
H
$^{1}H$ NMR, $CDCl_3$, 400 MHz
0.98
0.95
4.01
1.09
1.06
1.06
1.01
4.06
1.02
1.00
2.01
4.01
0.98
1.02
1.07
3.00
9.05
9.10
f1 (ppm)

xy-521.3.fid

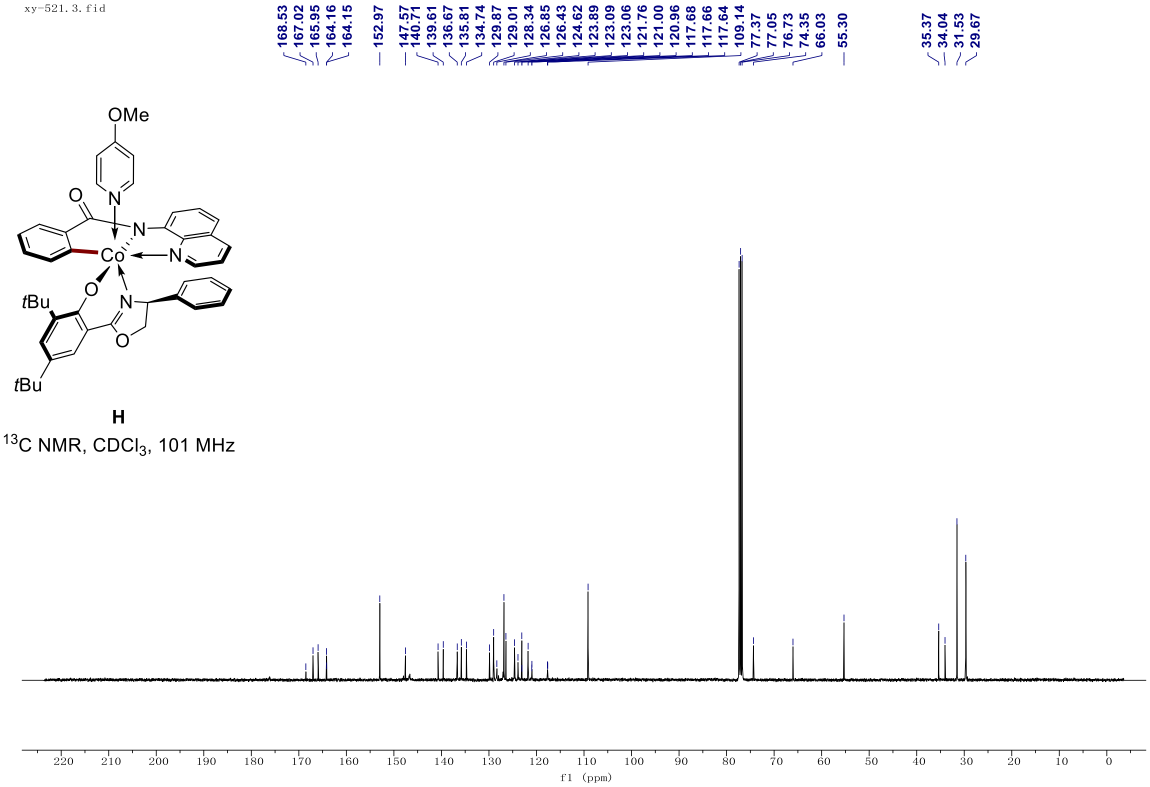


**H**

$^{13}C$ NMR, $CDCl_3$, 101 MHz
